# Mitochondrial-Related Transcriptome Feature Correlates with Prognosis, Vascular Invasion, Tumor Microenvironment, and Treatment Response in Hepatocellular Carcinoma

**DOI:** 10.1155/2022/1592905

**Published:** 2022-04-30

**Authors:** Yizhou Wang, Feihong Song, Xiaofeng Zhang, Cheng Yang

**Affiliations:** ^1^Fourth Department of Hepatic Surgery, Third Affiliated Hospital of Second Military Medical University, Shanghai 200438, China; ^2^Department of Special Treatment, Third Affiliated Hospital of Second Military Medical University, Shanghai 200438, China

## Abstract

**Background:**

Hepatocellular carcinoma (HCC) is the most common subtype of primary liver cancer, which was highly correlated with metabolic dysfunction. Nevertheless, the association between nuclear mitochondrial-related transcriptome and HCC remained unclear.

**Materials and Methods:**

A total of 147 nuclear mitochondrial-related genes (NMRGs) were downloaded from the MITOMAP: A Human Mitochondrial Genome Database. The training dataset was downloaded from The Cancer Genome Atlas (TCGA), while validation datasets were retrieved from the International Cancer Genome Consortium (ICGC) and Gene Expression Omnibus (GEO). The univariate and multivariate, and least absolute shrinkage and selection operator (LASSO) Cox regression analyses were applied to construct a NMRG signature, and the value of area under receiver operating characteristic curve (AUC) was utilized to assess the signature and nomogram. Then, data from the Genomics of Drug Sensitivity in Cancer (GDSC) were used for the evaluation of chemotherapy response in HCC.

**Results:**

Functional enrichment of differentially expressed genes (DEGs) between HCC and paired normal tissue samples demonstrated that mitochondrial dysfunction was significantly associated with HCC development. Survival analysis showed a total of 35 NMRGs were significantly correlated with overall survival (OS) of HCC, and the LASSO Cox regression analysis further identified a 25-NMRG signature and corresponding prognosis score based on their transcriptional profiling. HCC patients were divided into high- and low-risk groups according to the median prognosis score, and high-risk patients had significantly worse OS (median OS: 27.50 vs. 83.18 months, *P* < 0.0001). The AUC values for OS at 1, 3, and 5 years were 0.79, 0.77, and 0.77, respectively. The prognostic capacity of NMRG signature was verified in the GSE14520 dataset and ICGC-HCC cohort. Besides, the NMRG signature outperformed each NMRG and clinical features in prognosis prediction and could also differentiate whether patients presented with vascular invasions (VIs) or not. Subsequently, a prognostic nomogram (C-index: 0.753, 95% CI: 0.703~0.804) by the integration of age, tumor metastasis, and NMRG prognosis score was constructed with the AUC values for OS at 1, 3, and 5 years were 0.82, 0.81, and 0.82, respectively. Notably, significant enrichment of regulatory and follicular helper T cells in high-risk group indicated the potential treatment of immune checkpoint inhibitors for these patients. Interestingly, the NMRG signature could also identify the potential responders of sorafenib or transcatheter arterial chemoembolization (TACE) treatment. Additionally, HCC patients in high-risk group appeared to be more sensitive to cisplatin, vorinostat, and methotrexate, reversely, patients in low-risk group had significantly higher sensitivity to paclitaxel and bleomycin instead.

**Conclusions:**

In summary, the development of NMRG signature provided a more comprehensive understanding of mitochondrial dysfunction in HCC, helped predict prognosis and tumor microenvironment, and provided potential targeted therapies for HCC patients with different NMRG prognosis scores.

## 1. Introduction

Globally, primary liver cancer is one of the most aggressive and difficult-to-treat malignant cancers, with a 5-year survival rate of less than 21% [1]. Hepatocellular carcinoma (HCC) comprises the most common type of primary liver cancer, accounting for 90% of all liver cancer cases [2]. Besides, patients with HCC were often diagnosed in advanced stage owing to no apparent symptoms in early stage, probably leading to the poor survival. With the approval of sorafenib, lenvatinib, and other immunotherapy regimens for advanced HCC patients, the survival of metastatic or unresectable HCC patients has been improved in these years, but the therapeutic outcomes are still largely unsatisfactory [3, 5]. As is known, alpha-fetoprotein (AFP) is the most widely used serum biomarker for the HCC detection and treatment evaluation; however, it is not a robust and specific biomarker for HCC [4]. In addition, vascular invasion (VI), as a critical risk factor, is the main herald of HCC recurrence though for HCC patients receiving surgical resection [5]. Vascular invasion could be divided into two subtypes, macroscopic vascular invasion and microscopic vascular invasion, both were highly associated with tumor recurrence and poor performance status [6]. However, the limitation in the detection of VI hinders its application as a robust biomarker for determining the clinical outcomes of HCC patients. Therefore, novel prognostic models and better prognostic molecular markers are urgently required to improve the HCC management and accurately predict clinical outcomes of HCC, especially for the AFP-negative HCC.

The liver and mitochondria are the two centers of metabolism at the whole organism and cellular levels, respectively. Emerging evidences clearly suggested that mitochondrial dysfunction or maladaptation contributed to the detrimental effects on hepatocyte bioenergetics, reactive oxygen species (ROS) homeostasis, endoplasmic reticulum (ER) stress, inflammation, and cell death [7–9]. The liver mitochondria have unique features because the liver plays a central role in the regulation of a variety of metabolic functions including maintaining the homeostasis of carbohydrate, lipid, amino acid, and protein. Previous studies have revealed critical roles of mitochondrial genes in the carcinogenesis and development of HCC. For example, mitochondrial trans-2-enoyl-CoA reductase (*MECR*) had been identified as an oncogene which was significantly overexpressed in HCC cell lines [10]. Likewise, overexpression of mitofusin1 (*MFN1*) in HCC cells promoted mitochondrial fusion and inhibited cell proliferation, invasion, and migration via modulating metabolic shift from aerobic glycolysis to oxidative phosphorylation [11]. In addition, it has been proved that upregulation of aspartyl-tRNA synthetase (*DARS2*) promoted hepatocarcinogenesis through the MAPK/NFAT5 pathway [12]. However, most of these studies focused on a single gene instead of the integrated cluster of mitochondrial-related genes. Therefore, it will be of more value to evaluate the role of all the mitochondrial-related genes in the prognosis of HCC.

**Figure 1:**
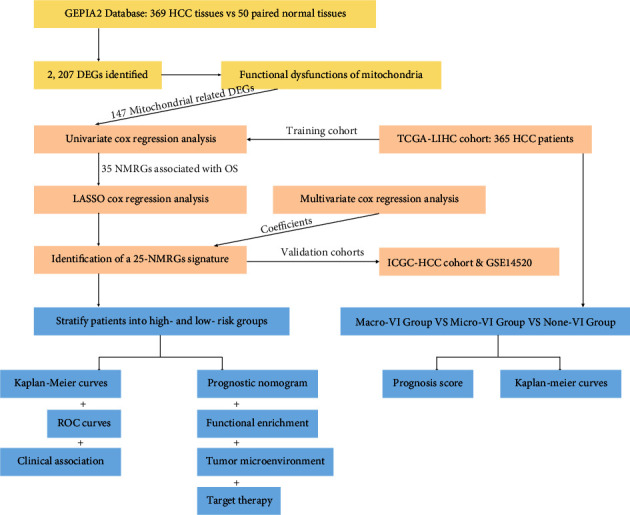
The flow-process diagram for the construction of the NMRG signature and exploration of clinicopathological association and potential targeted therapy.

In this study, we initially analyzed the transcriptome profiling of 147 NMRGs and the corresponding clinical data of patients with HCC from TCGA and then identified 35 NMRGs having significant influence on the survival of HCC patients by the univariate Cox regression analysis. Subsequently, we used the least absolute contraction and selection operator (LASSO) regression analysis and finally developed a novel 25-NMRG prognosis signature. Besides, the prediction efficacy of the established NMRG prognosis signature was verified in the validation datasets, including ICGC-HCC cohort from the ICGC and GSE14520 from the GEO. Based on the NMRG signature, a nomogram was further constructed to predict the prognosis of HCC. Moreover, the good AUC values demonstrated the reliable and stable predicting ability of the prognosis signature and nomogram. The functional differentiation, tumor microenvironment, and treatment response of precision therapy between high- and low-risk groups were further investigated to promote the precision medicine for HCC patients. The study design was mainly exhibited in a work flowchart (Figure 1).

## 2. Materials and Methods

### 2.1. Data Collection

The gene expression data and the clinical information of 365 HCC patients were collected from the Liver Hepatocellular Carcinoma (TCGA-LIHC) cohort from TCGA which was regarded as the training dataset (Table 1), while the ICGC-HCC (namely, LIRI-JP) cohort with 260 patients and GSE14520 with 242 patients from the GEO were defined as two independent validation datasets. A comprehensive list of NMRGs was downloaded from the MITOMAP: A Human Mitochondrial Genome Database (https://www.mitomap.org/MITOMAP, last updated date: January 15^th^, 2021), which comprised a total of 147 NMRGs.

### 2.2. The Analysis of Differentially Expressed Genes in HCC

The transcriptome data analysis between 369 HCC tumor tissues and 50 adjacent paired normal tissues was conducted online in the GEPIA (http://gepia2.cancer-pku.cn) for the identification of the differentially expressed genes (DEGs, ∣log2 − fold change (FC) | >1, *Q* − value < 0.01) between the HCC samples and normal samples. The visualization of the volcano plot and heatmap was performed using the “ggplot” package.

### 2.3. Signature Construction Based on Nuclear Mitochondrial-Related Genes

The univariate Cox regression was used to identify OS-associated NMRGs. Next, the LASSO regression model was selected to minimize the overfitting and identify the most significant survival-associated NMRGs in HCC via the “glmnet” package. Meanwhile, the multivariate Cox regression analysis was then used to determine the corresponding coefficients. The following formula based on a combination of coefficient and gene expression was used to calculate the prognosis score:

(1) $$\begin{array}{c} \text{Prognosis}\_\text{score}=\overset{n}{\underset{i=1}{\sum }}\text{G}\text{en}{\text{e}}_{i}*\text{coe}{\text{f}}_{i,} \end{array}$$

where *n*, Gene*_i_,* and coef*_i_* represent the number of genes involved in the signature, the level of gene expression, and the coefficient value, respectively.

To stratify patients into low- and high-risk groups, a median value of prognosis score was set for the cutoff value. The Kaplan-Meier survival curve analysis was conducted by using the “survival” and “survminer” packages, and log-rank test was performed to evaluate the survival rates between the low- and high-risk groups. The AUC values were calculated via using the “timeROC” package.

### 2.4. Establishment of a Novel Prognostic Nomogram for HCC

Several predominant prognostic factors in clinic including age, gender, AFP, vascular invasion, histological grading, clinical stages, TNM stages, alcohol consumption, and hepatitis status, together with prognosis score of NMRGs signature were investigated via the univariate and multivariate Cox regression analyses using the “rms” and “survival” packages, to find the independent prognostic factors. Next, we established a prognostic nomogram based on the independent prognostic factors.

### 2.5. Functional Enrichment Analysis

The GO (Gene Ontology) enrichment analysis was performed to determine significantly enriched GO terms for the differentially expressed genes between normal and tumor tissue samples. In order to investigate any changes in biological functions and related pathways between the high- and low-risk groups, HALLMARK gene set (including 50 gene sets from Molecular Signature Database, https://www.gsea-msigdb.org/gsea/msigdb/, [13]) enrichment analysis (GSEA), and KEGG (Kyoto Encyclopedia of Genes and Genomes) pathway enrichment analysis were performed. GSEA normalized the Enrichment Score for each gene set to account for the variation in gene set sizes, yielding a normalized enrichment score (NES). Enrichment analysis was performed by the “clusterprofiler” package and visualized using the “ggplot2”. The differentially expressed genes were defined with ∣log_2_ − fold change (FC) | >1, *P* < 0.05 in the functional enrichment analysis.

### 2.6. Tumor Microenvironment Analysis in HCC

The stromal, immune, and ESTIMATE scores were calculated using ESTIMATE [14], which could illustrate the properties of tumor infiltrated cells. Then, a heatmap of gene signature expression profiles denoting the activities of angiogenesis and immune further clarified the differentiation of tumor microenvironment between the high- and low-risk groups [15]. Finally, the CIBERSORT algorithm analysis was employed to explore 22 types of tumor-infiltrating immune cells.

### 2.7. The Evaluation of Precision Treatment and Chemotherapy Response

The GSE104580 dataset, including the transcriptomic data of 147 HCC patients (81 responders vs. 66 nonresponders) treated with TACE treatment, was enrolled in the present study to explore the predictive ability of novel prognosis score in the treatment response. Besides, GSE109211 dataset, including a total of 67 HCC patient samples treated with sorafenib (21 responders vs. 46 nonresponders) from the phase III STORM clinical trial (NCT00692770), was investigated to evaluate the capacity of prognosis score to predict sorafenib efficacy [16]. Meanwhile, the cell line data from the Genomics of Drug Sensitivity in Cancer (GDSC, https://www.cancerrxgene.org/) were downloaded to predict the treatment response of chemotherapeutic regimens between high- and low-risk groups, and the chemical drugs utilized in HCC, such as cisplatin, paclitaxel, and gemcitabine, for HCC patients were investigated. The index of half-maximal inhibitory concentration (IC50) was used for the response evaluation.

### 2.8. Statistical Analysis

All statistical analyses were conducted with the R package (v. 3.4.3, https://rstudio.com/). Fisher's test was executed for the comparison of categorical variables. The Kaplan-Meier curve analysis by using the log-rank test was used to evaluate the statistical significance of the survival rates between different risk groups. Concordance index, time-dependent ROC, and calibration were also important indicators used to assess the nomogram. *P* < 0.05 was considered statistically significant.

## 3. Results

### 3.1. Mitochondrial Dysfunction Contributed to the HCC Development

**Figure 2:**
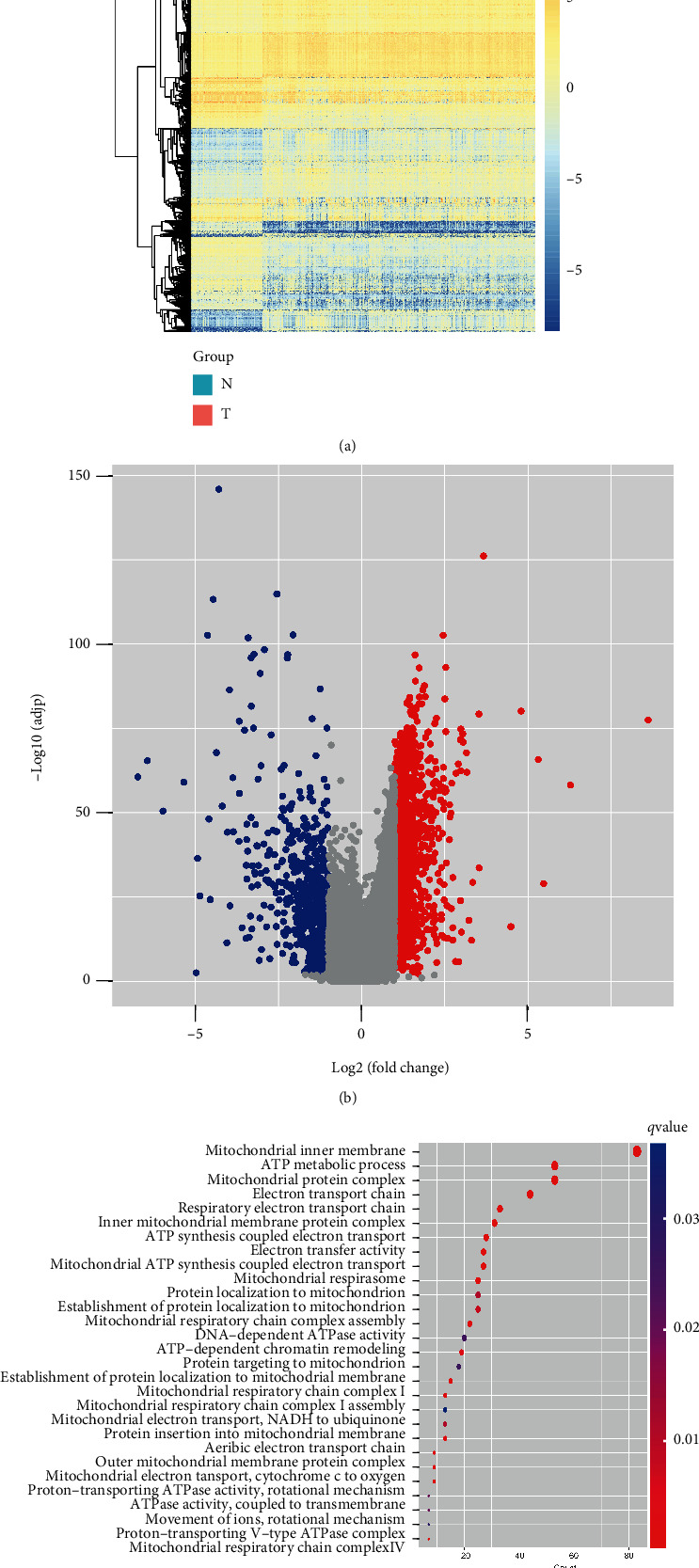
Mitochondrial dysfunction potentially promoted the hepatocarcinogenesis. (a) Transcriptional profiling of HCC and adjacent paired normal tissues. (b) Differentially expressed genes (DEGs) between HCC and adjacent paired normal tissues. Red dots represented significant upregulation and blue dots represented significant downregulation of DEGs in HCC tissues. (c) Identification of biological functions via the GO pathway enrichment analysis.

Using the ANOVA method, a total of 2,207 DEGs were identified between 369 HCC tumor tissues and 50 adjacent paired normal tissues (∣log_2_FC | >1, *Q* − value < 0.01, Supplementary Table 1), and it was demonstrated that there were 1,482 genes significantly upregulated and 725 genes significantly downregulated in the HCC tumor samples (Figures 2(a), 2(b)). In addition, biological functions and involved pathways of these identified 2,207 DEGs were analyzed by GO enrichment analysis, revealing that the DEGs were abundantly enriched in the pathways related to cell metabolisms, including mitochondrial inner membrane, ATP-dependent chromatin remodeling, and mitochondrial electron transport, NADH to ubiquinone pathways (Figure 2(c)), indicating that mitochondrial dysfunction was closely related to the carcinogenesis and development of HCC.

### 3.2. Construction of a Novel Nuclear Mitochondrial-Related Gene Prognosis Signature for HCC

**Figure 3:**
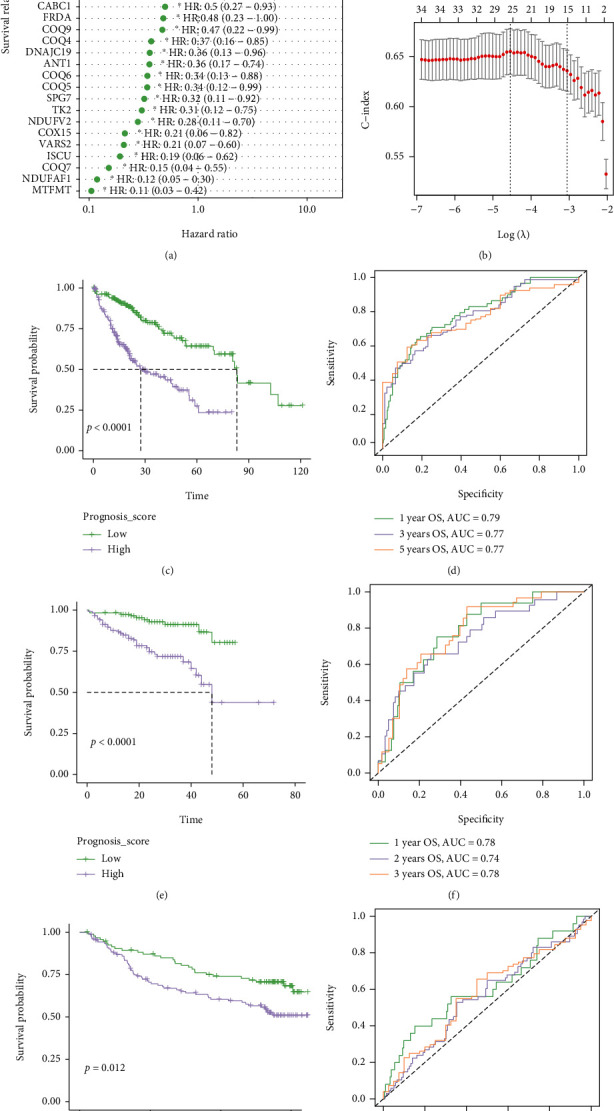
Construction and validation of the nuclear mitochondrial-related gene (NMRG) signature. (a) Univariate Cox regression analysis for selection of NMRGs correlated with overall survival of HCC patients. (b) LASSO Cox regression analysis determined a total of 25 NMRGs as the optimal combination for the NMRG signature construction. The Kaplan-Meier curves for HCC patients in high- and low-risk groups, from the TCGA cohort (c), from the ICGC-HCC cohort (e), and from the GSE14520 dataset (g). The ROC curves for OS at 1, 3, and 5 years in TCGA cohort (d), in ICGC-HCC cohort (f), and in GSE14520 dataset (h).

Univariate Cox regression analysis was performed to analyze the correlation between the transcriptional expression level of 147 NMRGs and the overall survival (OS) of HCC patients from the TCGA cohort. It was found that the elevated expression of 17 NMRGs was significantly correlated with the poorer prognosis of HCC patients, whereas the overexpression of other 18 NMRGs significantly contributed to the improved survival (*P* < 0.05, Figure 3(a)). These 35 OS-related NMRGs were then enrolled in the LASSO Cox regression analysis, finally constructing a NMRG prognosis signature for HCC patients based on the transcriptional profiling of selected 25 NMRGs (*NDUFV2, NDUFAF1, COX15, LRPPRC, MPV17, CARS2, DARS2, GARS, HARS2, LARS, PARS2, VARS2, MTFMT, TRMT10C, TRMU, C12ORF65, MRPL3, FRDA, ISCU, COQ6, COQ7, PDSS1, CABC1, SPG7,* and *ATAD3*), with the optimal value of *λ* (*λ* = 0.0106127) (Figure 3(b)). This novel prognosis score was calculated by multiplying the gene expression of each gene and its corresponding coefficient (Supplementary Table 2), which was obtained by the multivariate Cox regression analysis.

### 3.3. Survival Analysis and Validation of the NMRG Signature

According to the median prognosis score value, 365 HCC patients were divided into high-risk group and low-risk group. The analysis of the Kaplan-Meier curve showed that patients in high-risk group had significantly worse OS (median OS: 27.50 vs. 83.18 months, *P* < 0.0001, Figure 3(c)). Time-dependent ROC analysis was used to evaluate the prognostic evaluation ability of the NMRG signature (Figure 3(d)), and the AUC values at 1, 3, and 5 years for predicting OS were 0.79, 0.77, and 0.77, respectively. Furthermore, two independent cohorts were retrieved to validate the NMRG signature. The Kaplan-Meier curve analysis demonstrated that patients in high-risk group, from the ICGC cohort, had the significantly worse OS (median OS: 48.02 months vs. unreached, *P* < 0.0001, Figure 3(e)). The AUC values for predicting OS at the 1-, 2-, and 3-year timepoints were 0.78, 0.74, and 0.78, respectively (Figure 3(f)). Furthermore, the NMRG signature was verified in another independent dataset of GSE14520 from the GEO database. It could be also observed that patients in high-risk group had significantly worse OS (median OS: unreached vs. unreached, *P* = 0.012, Figure 3(g)). The AUC values for predicting OS at 1, 3, and 5 years were 0.61, 0.56, and 0.58, respectively (Figure 3(h)).

### 3.4. Comparison of Clinicopathological Features between the High- and Low-Risk Groups

**Figure 4:**
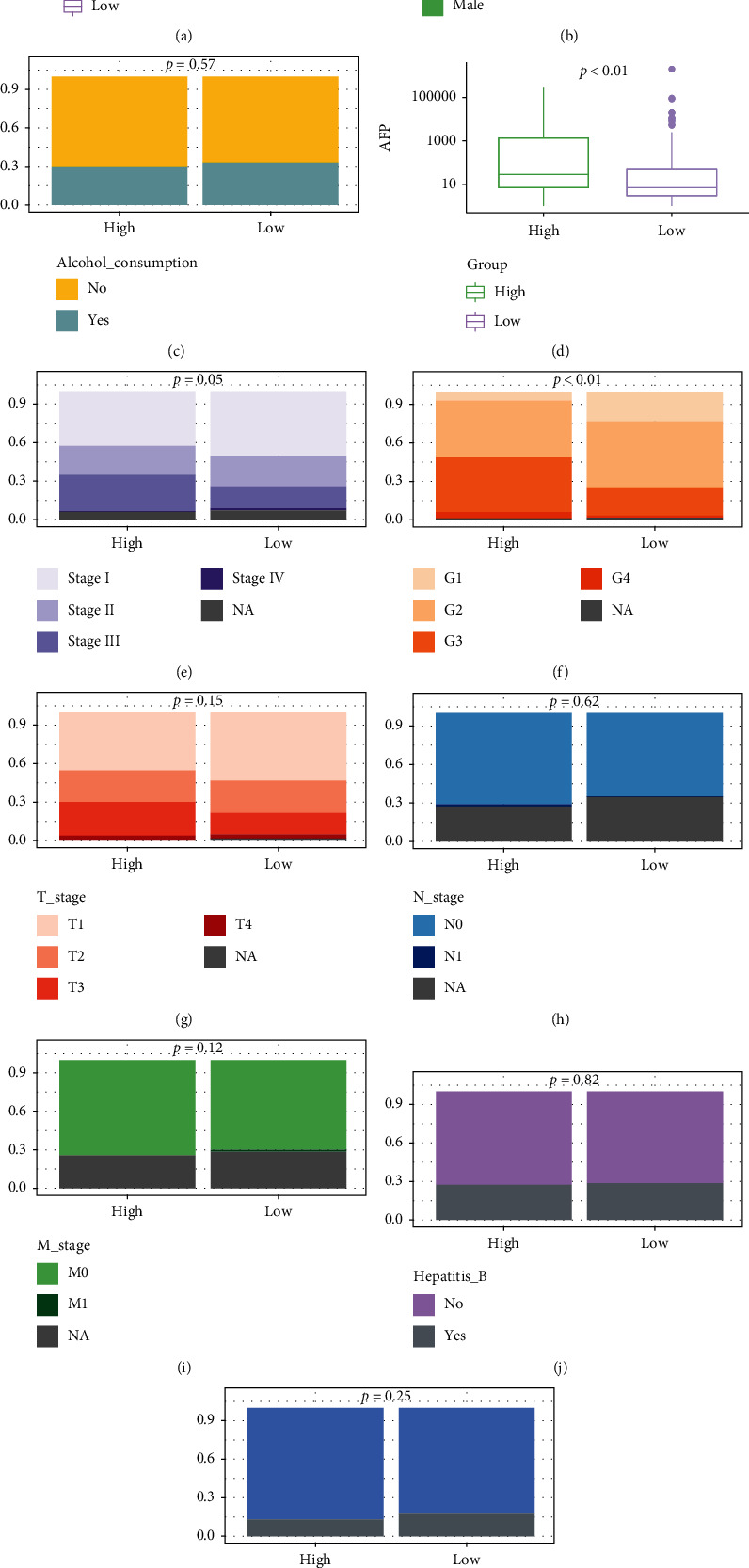
Association analysis between the NMRG signature and clinical features. (a) The boxplots showed the distribution of age at diagnosis between the high- and low-risk groups. (b) The percentage-staked bar plots for gender distribution between the high- and low-risk groups. (c) The percentage-staked bar plots for the distribution of alcohol consumption between the high- and low-risk groups. (d) The boxplots showed the distribution of AFP concentration between the high- and low-risk groups. The percentage-staked bar plots for the distribution of neoplasm cancer stages (e), histological grading (f), T stages (g), N stages (h), M stages (i), Hepatitis_B status (j), and Hepatitis_C status (k) between the high- and low-risk groups.

The differences of clinicopathological features of patients from the high- and low-risk groups, in the TCGA cohort, were subsequently analyzed. The age at diagnosis of patients in the high-risk group did not differ with that in the low-risk group (median age: 60 [18, 85] vs. 63 [16, 90] months, *P* = 0.21, Figure 4(a)). Meanwhile, there was no statistically significant difference in gender between these two groups (*P* > 0.05, Figure 4(b)). Besides, no significant difference of the alcohol consumption level was found between the high- and low-risk groups, either (*P* = 0.57, Figure 4(c)). As for the level of AFP, it demonstrated that patients in high-risk group had the significantly higher level of AFP (median level: 28 vs. 7 ng/mL, $\underset{¯}{P}<0.01$, Figure 4(d)). Moreover, there were more patients from the high-risk group having advanced neoplasm cancer stages (45.35% vs. 54.44% in stage I, 23.84% vs. 25.44% in stage II, 30.23% vs. 18.34% in stage III, and 0.58% vs. 1.78% in stage IV, *P* = 0.05, Figure 4(e)) and higher histological grading (G1: 7.18% vs. 23.46%, G2: 44.75% vs. 52.51%, G3: 43.09% vs. 22.34%, and G4: 4.97% vs. 1.68%, *P* < 0.01, Figure 4(f)). However, no statistically significant difference in the tumor stage, lymph node invasion, and metastasis (TNM stage) was observed between these two groups (*P* > 0.05, Figures 4(g)–4(i)). Finally, it was found that there was no significant difference in the ratio of patients infected with hepatitis B, nor with hepatitis C between the high- and low-risk groups (*P* > 0.05, Figures 4(j) and 4(k)).

### 3.5. Association between NMRG Prognosis Signature and VIs

**Figure 5:**
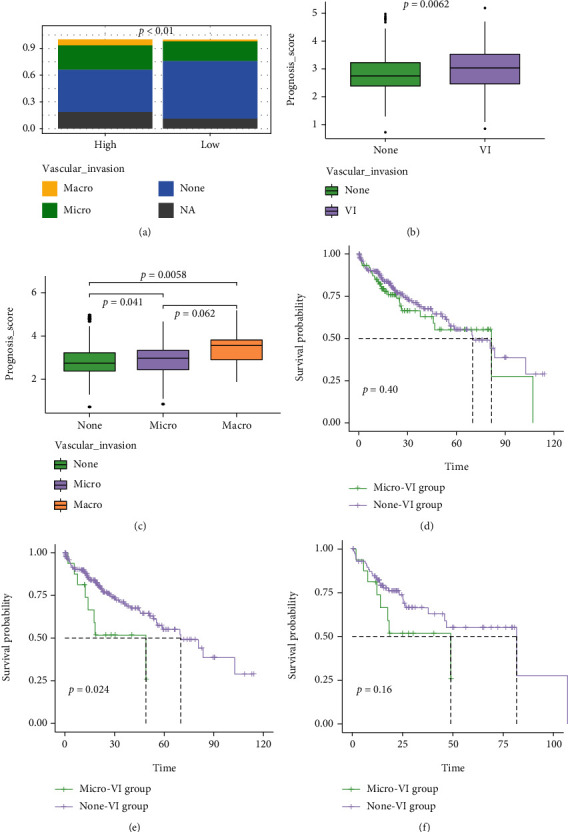
The application of the NMRGs signature in the groups with vascular invasions (VIs) or not. (a) The percentage-staked bar plots for the distribution of VIs between high- and low- risk groups. (b) Comparison of prognosis score between groups with VIs or not. (c) Comparison of prognosis score between groups with macro-VIs, micro-VIs, and without VIs. The Kaplan-Meier curves for HCC patients between micro-VI and none-VI groups (d). (e) Green and purple lines represent macro-VI group and none-VI group, respectively. (f) Green and purple lines represent macro-VI group and micro-VI group, respectively.

**Figure 6:**
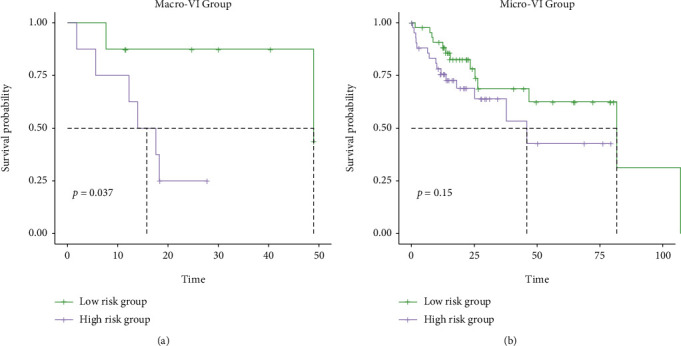
Comparison of overall survival between high- and low-risk HCC patients in the groups with macro-VIs or micro-VIs. The Kaplan-Meier curves between high- and low-risk HCC patients in the macro-VI group (a) and micro-VI group (b).

In the TCGA cohort, there were 111 patients presented with VIs (17 patients with macrovascular invasions and 94 patients with microvascular invasions), and 211 patients did not present with VIs. Further investigation for histopathological subtypes found that more HCC patients with VIs were included in the high-risk group (macro-VI: 8.05% vs. 2.47%, micro-VI: 33.56% vs. 24.69%, and none-VI: 58.39% vs. 72.84%, *P* < 0.01, Figure 5(a)). Remarkably, it was revealed that patients with VIs had the significantly higher prognosis score, compared to those without VIs (Figure 5(b)), while patients with macrovascular invasions had the highest prognosis score (Figure 5(c)). The survival analysis demonstrated that patients with macro-VI phenotype had significantly worse OS than those without VIs (median OS macro-VI vs. none-VI: 48.95 vs. 70.01 months, *P* = 0.024), while there was no significant difference in the OS between patients with micro-VI and none-VI, neither between patients with micro-VI and macro-VI (Figures 5(d)–5(f)). Nevertheless, it was shown that HCC patients having high prognosis score had worse OS, regardless of whether presenting with VI or not (median OS in VI group: 37.75 vs. 81.67 months, *P* = 0.011; none-VI group: 55.35 vs. 83.51 months, *P* = 0.0018, Supplementary Figure 1A-1B). Of note, in macro-VI group, patients with high prognosis score exhibited extremely poorer OS (15.77 vs. 48.95 months, *P* = 0.037, Figure 6(a)). Similarly, patients in micro-VI group having high prognosis score also had worse OS, however, with no statistically significant difference (45.89 vs. 81.67 months, *P* = 0.15, Figure 6(b)), mainly owing to the limited patient number.

### 3.6. Establishment of a Prognostic Nomogram

**Figure 7:**
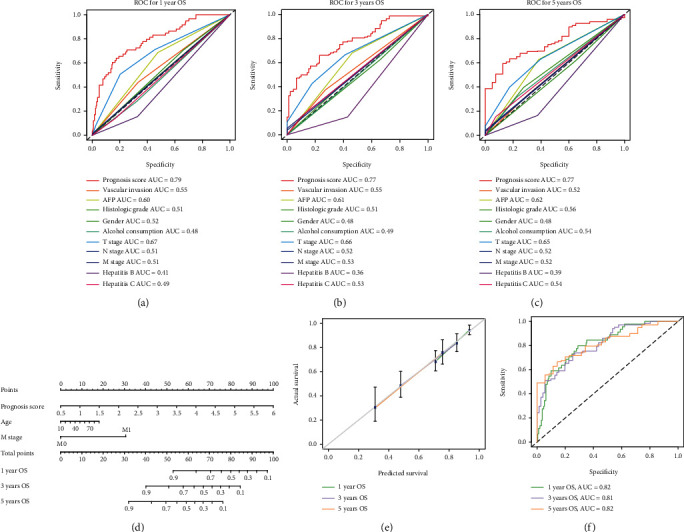
Construction of a novel nomogram for HCC patients based on the NMRG signature. The ROC curves of a variety of clinical features for overall survival (OS) at 1 (a), 3 (b), and 5 years (c). (d) The NMRG-based nomogram was constructed to predict the OS of HCC patients. (e) The calibration plots for the evaluation of predicted OS at 1, 3, and 5 years. (f) The ROC curves of the nomogram for OS at 1, 3, and 5 years in the analysis of TCGA-HCC cohort.

The multivariate Cox regression analysis exhibited that the prognosis score was an independent prognostic indicator for OS in HCC patients from the TCGA cohort (Table 2) and the ROC curve analysis revealed that the NMRG signature had the highest sensitivity and specificity in predicting the OS of HCC patients, compared with clinic-related features, including AFP, VI, histological grading, and TNM clinical stages (Figures 7(a)–7(c)). Meanwhile, the NMRG signature also had better sensitivity and specificity than each single NMRG alone in the prognosis prediction (Supplementary Figure 2A-2C). Subsequently, we combined three independent prognostic indexes, including the age, tumor metastasis status, and prognosis score to construct a nomogram to predict the OS of HCC patients (Figure 7(d)). Each patient had an integrated score according to the prognostic parameters, and the higher the total score indicated a worse outcome. The calibration chart showed that the OS probability predicted by the nomogram approximated the actual OS probability very well (Figure 7(e)). The C-index of the nomogram was 0.753 (95% CI, 0.703~0.804), and the AUC values of the nomogram were 0.82, 0.81, and 0.82 at the 1-, 3-, and 5-year timepoints, respectively (Figure 7(f)).

### 3.7. Genomic Feature Associated with the NMRG Signature

**Figure 8:**
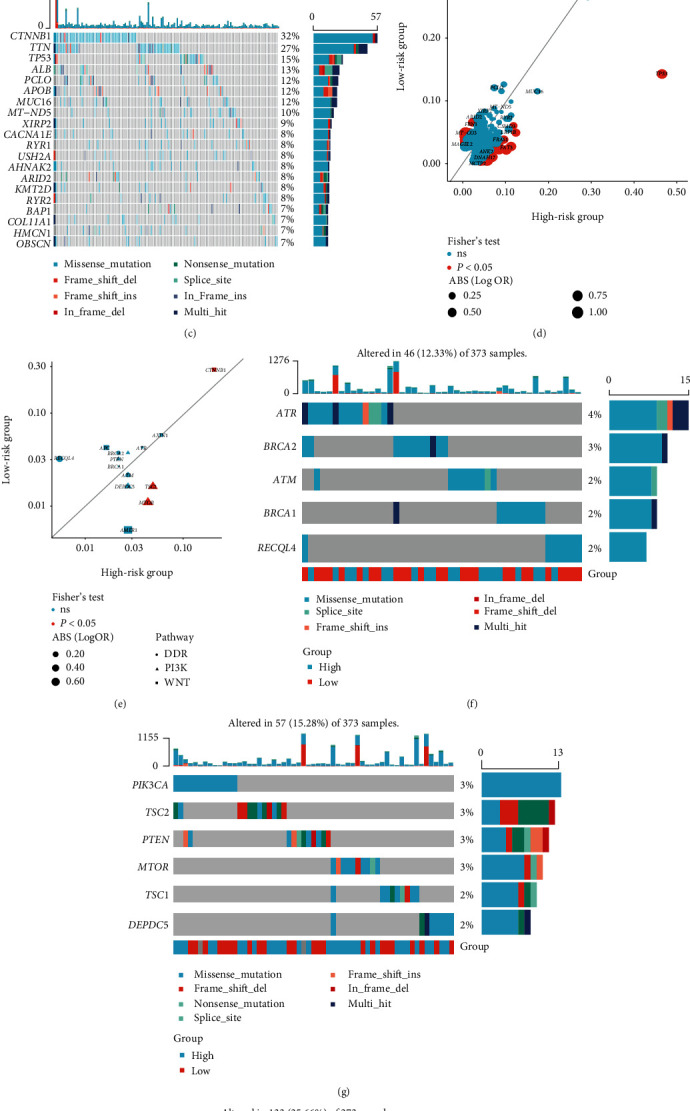
The analysis of genomic alterations between the high- and low-risk groups. (a) The boxplots showed the mutation counts between the high- and low-risk groups. The genomic profiling of the top 20 most frequently altered genes in the high-risk group (b) and in the low-risk group (c). (d) Genomic alteration enrichment of altered genes between the high- and low-risk groups. (e) Genomic alteration enrichment of altered signaling pathways between the high- and low-risk groups. The genomic profiles of altered events in DDR (f), PI3K (g), and WNT signaling pathways (h).

Statistical analysis displayed that there was no significant difference of the mutation count between the high- and low-risk groups (*P* = 0.34, Figure 8(a)), but mutation profiles revealed that the most frequently altered genes between the high- and low-risk groups were distinct (Figures 8(b) and 8(c)). HCC patients in the high-risk group had a significantly higher prevalence of *TP53* (frequency: 46% vs. 15%, *P* < 0.05, Supplementary Tables 3–4), whereas a higher prevalence of *CTNNB1* was presented in the low-risk group (frequency: 32% vs. 21%, *P* < 0.05, Supplementary Tables 3–4). Then, the altered events of patients between the high- and low-risk groups were compared (genes were excluded if their alteration event count less than 5 times happened simultaneously in both groups), demonstrating that the prevalence of a total of 61 genes was significantly different between the high- and low-risk groups (*P* < 0.05, Supplementary Table 5). The result showed that 24 altered genes, including *CTNNB1*, *FBN1*, and *MT-CO3*, were significantly prevalent in the low-risk group (*P* < 0.05, Figure 8(d), Supplementary Table 5), whereas 37 altered genes, for instance, *TP53*, *LRP1B*, and *FAT3*, were significantly prevalent in the high-risk group (*P* < 0.05, Figure 8(d), Supplementary Table 5). Subsequently, genomic alterations of the known cancer-related signaling pathways, such as DNA Damage Repair (DDR), Phosphatidylinositol-3-Kinase (PI3K), and WNT signaling pathway, were further investigated. Of note, it was found that WNT signaling-related gene *CTNNB1* was more frequently altered in the low-risk group (*P* < 0.05, Figure 8(e)), but *TSC2* and *MTOR* associated with PI3K signaling pathway were significantly enriched in the high-risk group (*P* < 0.05, Figure 8(e)). The genomic alteration profiles describing the altered events in DDR, PI3K, and WNT signaling pathways were exhibited in Figures 8(f)–8(h).

### 3.8. Identification of Differential Biological Functions

**Figure 9:**
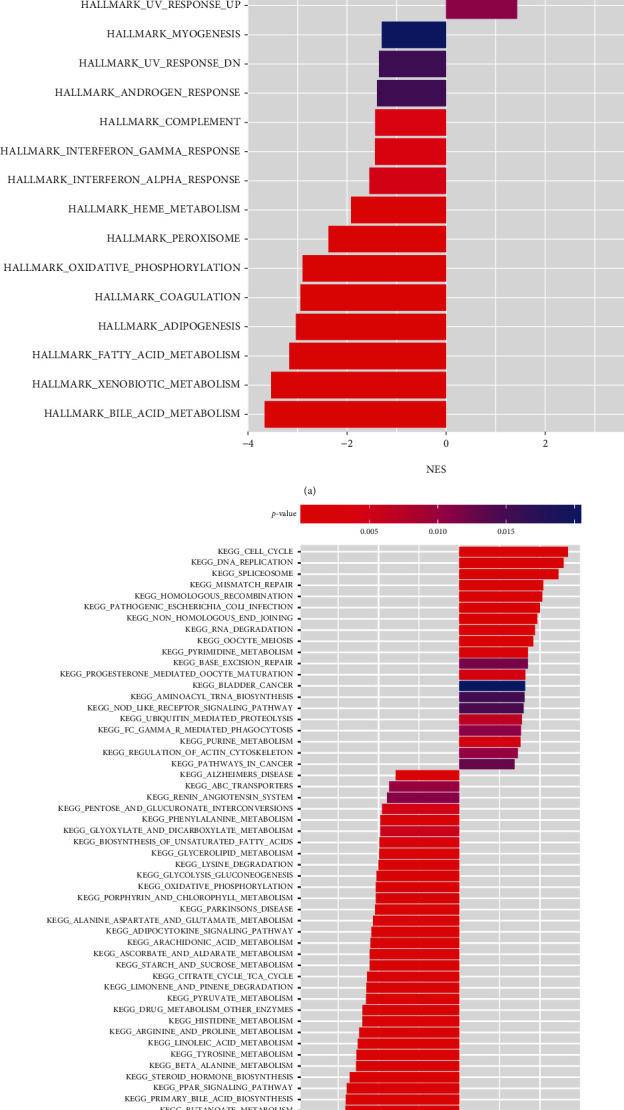
Functional enrichment analysis between the high- and low-risk groups. The HALLMARK gene set enrichment analysis (a) and the KEGG pathway enrichment analysis (b). *P* < 0.05 was considered statistically significant.

Further analysis of DEGs revealed a total of 599 genes were significantly upregulated and 487 genes were downregulated in low-risk groups (Supplementary Figure 3). Based on the identified DEGs, the differential molecular mechanisms between two groups were further elucidated via HALLMARK gene set and KEGG pathway enrichment analyses. The HALLMARK gene set enrichment analysis showed the significant enrichment of E2F targets, G2M checkpoint, and Myc targets. (Figure 9(a)), while the KEGG pathway enrichment analysis exhibited a significant abundance of cell cycle, DNA replication, and spliceosome (Figure 9(b)). In addition, both HALLMARK gene set enrichment analysis and KEGG pathway enrichment analysis showed that the metabolism-related pathways were significantly enriched, especially for fatty acid metabolism (Figures 9(a) and 9(b)).

### 3.9. Correlation between the NMRG Signature and Tumor Microenvironment

**Figure 10:**
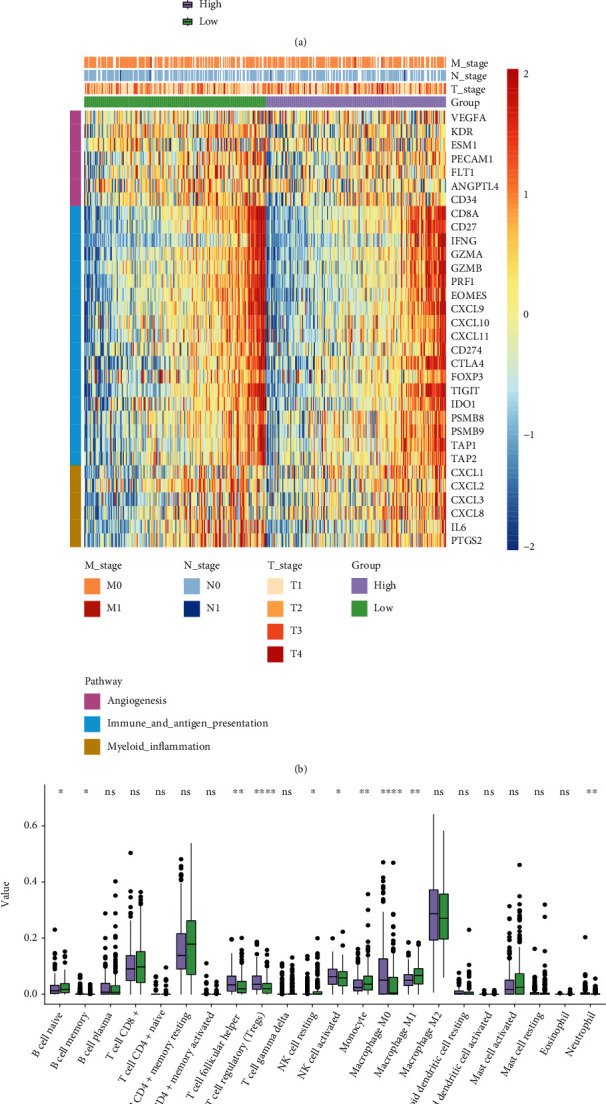
Comparison of tumor microenvironment (TME) between the high- and low-risk groups. (a) The statistical analyses of the stromal score, immune score, and ESTIMATE score between the high- and low-risk groups. (b) Heatmap demonstrated the expression of genes related to angiogenesis (purple), immune and antigen presentation (blue), and myeloid inflammation (brown). (c) The analysis of 22 immune infiltrated cells between high- and low-risk groups. ^∗∗∗∗^*P* < 0.0001, ^∗∗∗^*P* < 0.001, ^∗∗^*P* < 0.01, ^∗^*P* < 0.05.

Notably, the stromal score, immune score, and ESTIMATE score were nearly equivalent between the high- and low-risk groups (Figure 10(a)). The gene expression profiles of angiogenesis, immune and antigen presentation, and myeloid inflammation signatures between the high- and low-risk groups demonstrated that there were no distinct differences in these tumor microenvironment-related pathways (Figure 10(b)). The CIBERSORT algorithm analysis revealed that B cell memory, T cell follicular helper, regulatory T cells (Tregs), activated NK cells, macrophage M0, and neutrophils were significantly enriched in the high-risk group (*P* < 0.05, Figure 10(c)). Besides, the low-risk group had a significant abundance of naive B cells, resting NK cells, monocyte, and macrophage M1 (*P* < 0.05, Figure 10(c)).

### 3.10. The Signaling Pathways Potentially Targeted by Sorafenib Therapy

**Figure 11:**
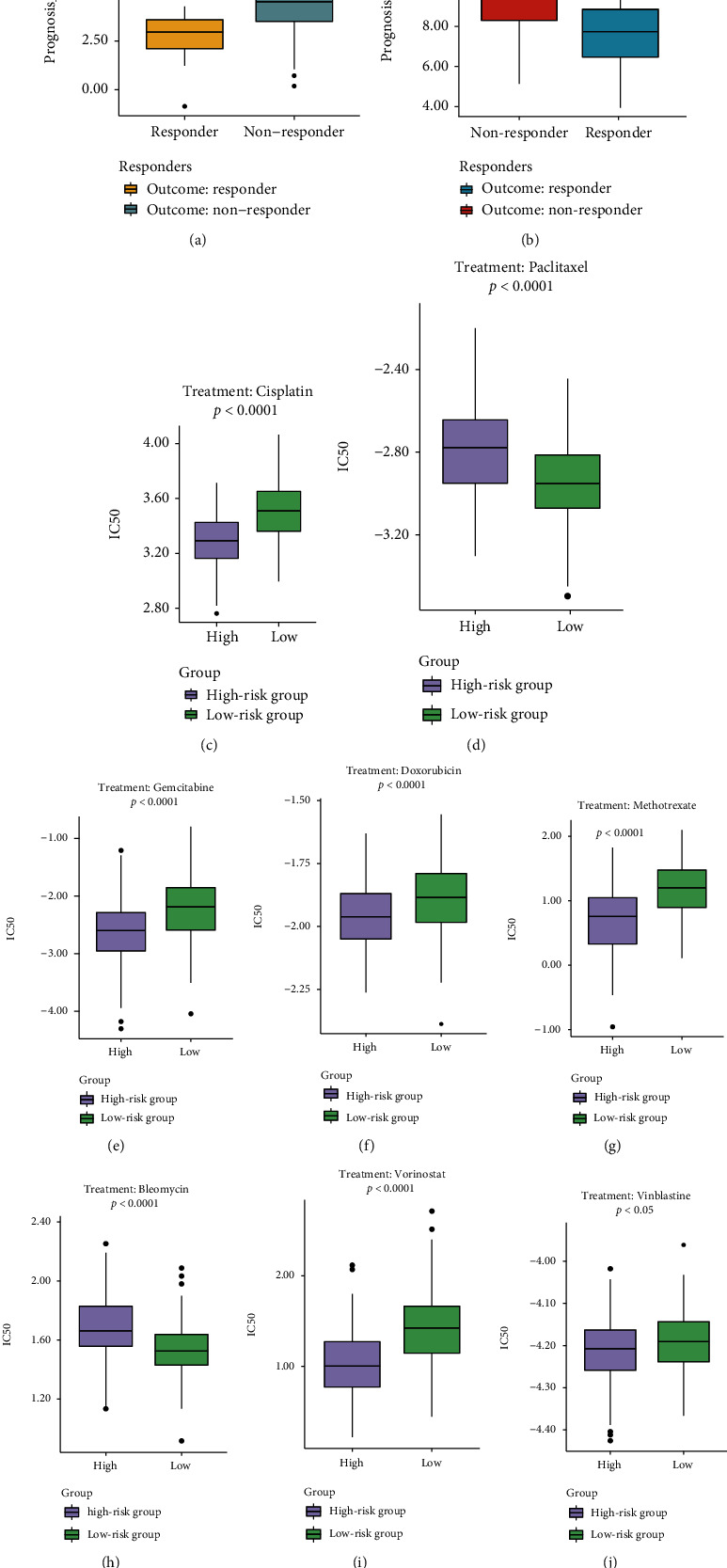
The evaluation of treatment responses by the novel prognosis score based on NMRG signature. (a) The treatment response prediction of the sorafenib therapy in the GSE109211 dataset. (b) The treatment response prediction of the transcatheter arterial chemoembolization (TACE) therapy in the GSE104580 dataset. (c–j) The boxplots of the evaluated IC50 for commonly used chemodrugs between the high- and low-risk groups by the analysis of cell line data from the GDSC database. ^∗∗∗∗^*P* < 0.0001, ^∗^*P* < 0.05.

An independent cohort (GSE109211), including 67 HCC patients treated with sorafenib, was utilized to evaluate the efficacy of sorafenib therapy in NMRG-risk groups. Notably, HCC patients who responded to sorafenib had significantly lower prognosis score (*P* = 0.0066, Figure 11(a)). Subsequently, the specific signaling pathways potentially targeted by sorafenib were further investigated. The DEG analysis showed a total of 1399 genes significantly upregulated and 1547 genes downregulated in the responders (Supplementary Figure 4). By the statistical analysis, the overlapping gene cluster between the low-risk and responder groups included 519 upregulated genes and 457 downregulated genes (Supplementary Figure 5A-5B), which might be highly correlated with the response of sorafenib therapy. Moreover, gene set enrichment analysis revealed that the upregulated pathways of xenobiotic metabolism, oxidative phosphorylation, apoptosis, and coagulation (by HALLMARK, Supplementary Figure 5C), ribosome and glycine, serine and threonine metabolism (by KEGG, Supplementary Figure 5D), besides, the downregulated pathways of KRAS signaling_DN (by HALLMARK, Supplementary Figure 5E) and olfactory transduction (by KEGG, Supplementary Figure 5F) were enriched, which was associated with treatment response of sorafenib.

### 3.11. Treatment Response Prediction of TACE Therapy and Chemotherapy

Another independent cohort (GSE104580 dataset) of 147 HCC patients who received the treatment of TACE was further employed in the present study. Of note, it was found that HCC patients responding to TACE therapy had markedly lower prognosis score (*P* < 0.0001, Figure 11(b)), further showing the robust capacity of prognosis score to predict treatment response. In addition, cell line data from the GDSC database were employed to predict the IC50 of commonly used chemodrugs for HCC patients from TCGA cohort, wherein six chemodrugs (cisplatin, gemcitabine, doxorubicin, methotrexate, vorinostat, and vinblastine) exhibited significantly lower IC50 in the high-risk group, indicating that those patients seemed to be more sensitive to the chemotherapeutic regimens containing these drugs (Figures 11(c)–11(j)). Conversely, the significantly lower estimated IC50 values in the low-risk group demonstrated that patients with lower prognosis score could benefit more from paclitaxel and bleomycin (Figures 11(d) and 11(h)).

Subsequently, the chemodrug efficacy under VI stratification (macro-VI, micro-VI, or non-VI) was further evaluated. The sensitivities to those investigated drugs were nearly equivalent between micro-VI and non-VI groups (Supplementary Figure 6). However, four chemodrugs (including cisplatin, gemcitabine, vorinostat, and methotrexate) had significantly lower IC50 in the macro-VI group (Supplementary Figure 6), while patients from micro-VI or non-VI group seemed to be more sensitive to paclitaxel (Supplementary Figure 6). Furthermore, among patients presented with the non-VI or micro-VI phenotype, lower estimated IC50 values of cisplatin, vorinostat, and methotrexate were observed in the high-risk group, whereas the low-risk group had lower estimated IC50 values of paclitaxel and bleomycin instead (Supplementary Figure 7 & 8). Besides, among non-VI patients, the lower IC50 values of gemcitabine, doxorubicin, and vinblastine were further found in the high-risk group (Supplementary Figure 7). Owing to the limited number of macro-VI patients (*N* = 17), there was no significant difference observed in the IC50 values of nearly all investigated chemodrugs between the high- and low-risk groups, except bleomycin (Supplementary Figure 9).

## 4. Discussion

A robust prognostic predictor for HCC patients is urgently needed due to the heterogeneous outcomes of HCC patients and the difficulties in the management and treatment strategy selection. Evidences from preclinical research supported mitochondrial dysfunction as a key factor in the pathogenesis of metabolic liver disease and cancer, which further suggested the development of targeting treatments for mitochondrial genes as an attractive strategy to suppress the HCC progression [17]. In the current study, functional enrichment analysis of DEGs between HCC tumors and normal tissue samples revealed that mitochondrial dysfunction was pivotal in the development of HCC, and aberrant expression of 35 NMRGs exerted notable influences on the prognosis of HCC. By the optimal combination, a 25-NMRG signature based on their transcriptional profiling was eventually constructed with the good performance in predicting prognosis and differentiating patients with or without VIs in HCC. The clinical association analysis also showed that higher NMRG prognosis score was positively correlated with advanced stages and tumor progression, which could help improve the management of patients with HCC and provide decision-making guidance on the treatment selection. Moreover, the NMRG signature had relatively better sensitivity and specificity as an independent prognostic predictor compared to the traditionally clinicopathological features. The NMRG signature-based prognostic nomogram was finally constructed, with better AUC values and great potential to be applied to clinical practices.

A pan-cancer study by Yuan et al. revealed that the coexpression networks of mitochondrial genes and their related nuclear genes were distinct across 13 cancer types, and in HCC the coexpression of mitochondrial genes was highly correlated with cancer-related signaling pathways, such as PI3K [18]. Besides, the enriched pathways were further found to be implicated with cell cycle, such as E2F targets, G2/M checkpoint, MYC targets, mitotic spindle, and DDR-related pathways in multiple cancer types [18], consistent with the results of functional enrichment of DEGs between the high- and low-risk groups in the present study. As reported previously, some certain mitochondrial-related genes have been proved to be strongly associated with prognosis in certain cancer types. For example, *NDUFV2,* known as NADH ubiquinone oxidoreductase core subunit V2, might act as a prognostic factor in uveal melanoma [19]. The aberrant expression of NADH dehydrogenase 1 alpha subcomplex assembly factor 1 (*NDUFAF1*) caused mitochondrial respiration deficiency, which was correlated with the carcinogenesis of primary pancreatic cancer [20]. Some other NMRGs, such as *LRPPRC* [21], *DARS2* [12], *GARS* [22], *ATAD3* [23], *TRMU* [24], and *PDSS1* [25] had been identified to be correlated with the carcinogenesis and progression in HCC. Moreover, the aberrant expression of *COX15* [26], *LARS* [27], *PARS2* [28], *MRPL3* [29], *ISCU* [30], *COQ7* [31], *SPG7* [32], *TRMT10C* [33], and *COQ6* [34] were found to have certain influence on the tumor invasions in many other cancer types. However, in the present study, it was the first time that the expressions of these NMRGs, including *HARS2*, *MPV17*, *MTFMT, C12ORF65*, *FRDA*, *CARS2*, *VARS2*, and *CABC1*, were found to have influence on the progression of HCC patients. Although some of them had already been identified to be associated with metabolic diseases or neurological disorders [35–42]. Further studies are merited to give deep insights on how they involve in the development of HCC and whether they could be targeted for treatment. In the present study, comprehensive transcriptomic profiling of NMRGs offered a deep insight for the role of mitochondria in HCC.

Clinical association analysis demonstrated that the high prognosis score could discriminate HCC patients with inferior outcomes. Furthermore, some known biomarkers such as AFP and des-carboxy prothrombin had very low sensitivity in detecting the HCC invasiveness [43]. VI, as an aggressive histopathological subtype of HCC, accounts for nearly 25% ~50% of HCC [5, 44]. In the present study the prognosis score of NMRG signature had the ability to differentiate HCC patients presented with or without VIs, especially for patients with macro-VIs. In addition, the higher NMRG signature prognosis score indicated the poorer OS of HCC patients no matter whether patients presented with macro-VIs, micro-VIs, or not. In short, the novel constructed NMRG signature, which was not only a prognostic biomarker but also a VI predictor, would help clinicians and/or physicians better manage the HCC patients.

In addition to the enriched pathways of cell cycle and DDR which were of importance to carcinogenesis and progression of tumor [45, 46], it could be conspicuously found that fatty acid metabolism was the top-ranked enriched pathway. The recent study revealed that *RIPK3*, playing an important role in necroptosis, could regulate fatty acid metabolism including fatty acid oxidation in hepatocarcinogenesis [47], and the abnormal regulation of fatty acid oxidation causing the large amount of ROS promoted HCC cell migration and invasion [48]. Therefore, the elimination of ROS via antioxidant drugs [49] and/or the blockade of fatty acid metabolism [47] could, as an effective treatment strategy, suppress the HCC progression to improve the HCC prognosis, simultaneously regulating the cell cycle and/or DDR-related pathway via CDK inhibitors [50]. Moreover, the accumulation of ROS could induce tumor-associated macrophage M2 polarization in the tumor microenvironment of HCC [47], which would enhance the progression of HCC [51]. Thus, the regulation of mitochondrial respiration or ROS level, as a treatment strategy for HCC, also could restrain the immunosuppressive activities of tumor-associated macrophages and improve the tumor microenvironment. In the present study, the high-risk group had the higher fraction of B cell memory, T cell follicular helper, and regulatory T cells (Tregs). These tumor-infiltrating lymphocytes (TILs) were suggested to be related to the response of immune checkpoints such as PD-1 and PD-L1 [25, 52], so that the efficacy of PD-1/PD-L1 inhibitors may be differed between high- and low-risk patients. Meanwhile, in patients from the high-risk group there was a significantly higher abundance of Tregs indicating the suppressive immunotherapy in HCC as reported before [53], while tivozanib [54] and cystathionine *β*-synthase [55] could decrease Tregs infiltration. Therefore, the combined treatment of immune checkpoint inhibitors, such as PD-1/PD-L1 inhibitors, with the antioxidant drugs and tivozanib or cystathionine *β*-synthase was highly recommended for the advanced HCC patients with high prognosis score. However, the combinational treatment of immune checkpoint inhibition, Tregs suppression, and ROS elimination needed verification in the clinical trials in the future, and the role of mitochondria in reshaping the tumor microenvironment of HCC also needed further investigation.

In the past researches, though sorafenib was of benefit to some HCC patients, most of patients had poor response to sorafenib or eventually had resistance to molecularly targeted therapies [56, 57]. Nevertheless, the estimate of the treatment strategy of classical drug sorafenib for HCC demonstrated that the prognosis score was significantly correlated with the response of sorafenib treatment. Moreover, the low prognosis score could predict the response of TACE treatment as well, revealing that the NMRG signature could serve as a response-related biomarker in the treatment of HCC. The response evaluation of commonly used chemotherapy in HCC further illustrated that high-risk patients might be more sensitive to the cisplatin, gemcitabine, doxorubicin, methotrexate, vorinostat, and vinblastine. It was reported previously that cisplatin could restrain the activity of androgen receptor to increase the efficacy of immunotherapies [58]. Gemcitabine, one of nonhepatotoxic chemotherapy drugs, could improve the prognosis of unresectable HCC patients [59]. Doxorubicin could synergize with icaritin-inducing immunogenic cell death and synergistic effects functioned to remodel the immunosuppressive tumor microenvironment in HCC [60]. While an antifolate drug of methotrexate could provoke the oxidative stress to sensitize HCC cells to sorafenib [61]. The combination of vorinostat and oxaliplatin had the ability of inhibiting HCC cell proliferation [62]. Moreover, vinblastine in combination with rapamycin was found to suppress HCC-related angiogenesis [63]. In reverse, it was more suitable for patients in the low-risk group to take the chemotherapy of paclitaxel and bleomycin. Paclitaxel could suppress the tumorigenesis of HCC via regulating cell proliferation and apoptosis [64]. While bleomycin was often utilized to induce DNA damage for cancer therapy, but which was seldomly used in the treatment of HCC except that the TACE combined with bleomycin was recommended for HCC patients [65]. In the present study, it was found that HCC patients in the low-risk group seemed to be more sensitive to chemotherapeutic treatment of paclitaxel and bleomycin, whereas HCC patients in high-risk group, cisplatin, gemcitabine, doxorubicin, methotrexate, vorinostat, and vinblastine were highly recommended if receiving the TACE treatment. Of note, all the identified drugs with more sensitivity in the high- or low-risk groups are needed to be validated in further clinical trials, though the GDSC dataset was very useful reporting drug sensitivity retrieved by experiments with cell lines, but without utilizing clinical data or tumor tissue samples. As is known, HCC is resistant to immunotherapy and chemotherapy [66]; however, chemotherapy drugs aiming at the regulation of tumor microenvironment [60], inhibiting angiogenesis [67], directly regulating the fatty acid metabolism [68], or the combinational treatment have the potential to suppress the HCC progression.

Genomic alteration enrichment analysis further identified that WNT signaling-related gene *CTNNB1* was more frequently altered in the low-risk group. The missense mutation in *CTNNB1* encoding *β*-catenin had the trunk role in the tumorigenesis of HCC and regulated tumor cell proliferation and tumor angiogenesis [69], while molecularly targeted therapy for *CTNNB1* had been found to have the potential in treatment of HCC [70], which was suggested as a promising therapy for HCC patients with low prognosis score in this study. In the high-risk group, altered genes *TSC2* and *MTOR* were highly enriched and these genes played important roles in the activation of PI3K/AKT/mTOR pathways that could lead to the HCC carcinogenesis, progression, and invasion [71]; thus, mTOR and/or PI3K inhibitors had a potential value in treating patients with HCC [72], particularly for the advanced HCC patients in the present study. But inhibitions of these signaling pathways might generate a prooncogenic tumor microenvironment and impel the recurrence of HCC [72], so an integrated effective strategy in treating HCC patients was urgently needed by the combinational treatment of molecularly targeted therapy, immunotherapy, chemotherapy, and other potential therapeutic strategies.

Overall, the novel constructed NMRG signature had robust and stable sensitivity and specificity as a prognostic predictor in HCC. In addition, the NMRG signature-based prognostic nomogram had the superior ability of predicting OS for HCC patients. But there were some limitations; in the present study, bioinformatic analyses were based on the public databases, and only nuclear mitochondrial-related genes were taken to construct the signature because of a lack of the transcription data of mitochondrial genes. We have been collecting the HCC patient samples, which would be used for the validation of the NMRG signature, and we also would take both NMRGs and mitochondrial genes together into consideration to further investigate the comprehensive role of mitochondria in the progression of HCC. Moreover, the suggested precision medicines would be verified in the lab and for clinical trials. Noteworthily, *HARS2*, *MPV17*, *MTFMT*, *C12ORF65*, *FRDA*, *CARS2*, *VARS2*, and *CABC1* were newly identified to be correlated with HCC prognosis, which also needed to be further investigated in further studies in vivo and/or in vitro.

## Figures and Tables

**Table 1:** Clinicopathological features of 365 HCC patients from the TCGA.

Variables		Number
Total		365
Age	Median (range)	61 [16, 90]
Gender	Male	246
	Female	119
Alcohol consumption	Yes	115
	No	250
AFP	Median (range)	15 [14, 203540] ng/mL
VI	Non-VI	211
	Micro-VI	94
	Macro-VI	17
Clinical stage	Stage I	170
	Stage II	84
	Stage III	83
	Stage IV	4
	NA	24
Histological grading	G1	55
	G2	175
	G3	118
	G4	12
	NA	15
T stage	T1	180
	T2	91
	T3	78
	T4	13
	NA	3
N stage	N0	248
	N1	4
	NA	113
M stage	M0	263
	M1	3
	NA	99
Hepatitis_B	Yes	102
	No	263
Hepatitis_C	Yes	56
	No	309

HCC: hepatocellular carcinoma; VI: vascular invasion; NA: not applicable.

**Table 2:** Hazard ratios for the NMRG signature and clinical features via the multivariate Cox regression analysis.

Index	Hazard ratio	95% CI	*P* value
Prognosis score	4.65	2.59-8.34	<0.0001
Age	1.04	1.01-1.07	0.01
Gender	0.99	0.47-2.11	0.99
Alcohol consumption	0.77	0.30-1.96	0.58
AFP	1.62	0.79-3.33	0.19
Histological grading	0.66	0.30-1.45	0.30
T stage	1.08	0.1-11.61	0.61
N stage	0.43	0.04-4.48	0.48
M stage	14.53	1.31-160.62	0.03
Hepatitis_B	0.69	0.31-1.54	0.36
Hepatitis_C	1.41	0.49-4.08	0.52
Vascular invasion	1.43	0.68-3.01	0.34

CI: confidence interval. Bold for “significant” in statistical analysis.

## Data Availability

The raw data used in this article were retrieved from public databases, which were exhibited in Materials and Methods.

## Supplementary Materials

Supplementary Materials Supplementary Figure 1: the Kaplan-Meier curves between high- and low-risk patients, in VI group (A) or none-VI group (B). Supplementary Figure 2: the ROC curves of each involved NMRG and prognosis_score for overall survival (OS) at 1 (A), 3 (B), and 5 years (C). Supplementary Figure 3:the differentially expressed genes between high- and low-risk groups. Supplementary Figure 4: the differentially expressed genes between nonresponder and responder groups. Supplementary Figure 5: the intersections of significantly upregulated genes (A) and downregulated genes (B) between low-risk patients and responder patients. The upregulated pathways potentially targeted by sorafenib via HALLMARK (C) and KEGG (D) enrichment analysis. The downregulated pathways potentially targeted by sorafenib via HALLMARK (E) and KEGG (F) enrichment analysis. Supplementary Figure 6: the evaluation of chemodrug treatment response between non-VI, micro-VI, and macro-VI groups. Supplementary Figure 7: The evaluation of chemodrug treatment response between high- and low-risk patients in the non-VI group. Supplementary Figure 8: the evaluation of chemodrug treatment response between high- and low-risk patients in the micro-VI group. Supplementary Figure 9: the evaluation of chemodrug treatment response between high- and low-risk patients in the macro-VI group. Supplementary Table 1: differentially expressed genes between tumor and normal tissues. Supplementary Table 2: coefficients of each NMRG involved in the NMRG signature. Supplementary Table 3: Top 20 high prevalence of altered genes in the high-risk group. Supplementary Table 4: Top 20 high prevalence of altered genes in the low-risk group. Supplementary Table: 5. the prevalence of a total of 61 genes was significantly different between high- and low-risk groups.
