# Supplementary material for: Mitochondrial-Related Transcriptome Feature Correlates with Prognosis, Vascular Invasion, Tumor Microenvironment, and Treatment Response in Hepatocellular Carcinoma
Source: Oxid Med Cell Longev. 2022 Apr 30;2022:1592905. doi: 10.1155/2022/1592905 (PMC9078845; doi:10.1155/2022/1592905)
Supplement: Supplementary Materials — Supplementary Figure 1: the Kaplan-Meier curves between high- and low-risk patients, in VI group (A) or none-VI group (B). Supplementary Figure 2: the ROC curves of each involved NMRG and prognosis_score for overall survival (OS) at 1 (A), 3 (B), and 5 years (C). Supplementary Figure 3:the differentially expressed genes between high- and low-risk groups. Supplementary Figure 4: the differentially expressed genes between nonresponder and responder groups. Supplementary Figure 5: the intersections of significantly upregulated genes (A) and downregulated genes (B) between low-risk patients and responder patients. The upregulated pathways potentially targeted by sorafenib via HALLMARK (C) and KEGG (D) enrichment analysis. The downregulated pathways potentially targeted by sorafenib via HALLMARK (E) and KEGG (F) enrichment analysis. Supplementary Figure 6: the evaluation of chemodrug treatment response between non-VI, micro-VI, and macro-VI groups. Supplementary Figure 7: The evaluation of chemodrug treatment response between high- and low-risk patients in the non-VI group. Supplementary Figure 8: the evaluation of chemodrug treatment response between high- and low-risk patients in the micro-VI group. Supplementary Figure 9: the evaluation of chemodrug treatment response between high- and low-risk patients in the macro-VI group. Supplementary Table 1: differentially expressed genes between tumor and normal tissues. Supplementary Table 2: coefficients of each NMRG involved in the NMRG signature. Supplementary Table 3: Top 20 high prevalence of altered genes in the high-risk group. Supplementary Table 4: Top 20 high prevalence of altered genes in the low-risk group. Supplementary Table: 5. the prevalence of a total of 61 genes was significantly different between high- and low-risk groups. [file 1592905.f1.zip › supplementary table 1A.pdf]

Supplementary Table 1A. Differentially expressed genes between tumor and normal tissues.

Gene Syml	Gene ID	Median (Tu	Median (N	Log2(Fold	adjp
SMIM24	ENSG0000	1.55	6.257	-1.509	9.69E-03
RSRP1	ENSG0000	16.95	36.53	-1.064	9.46E-03
ATHL1	ENSG0000	9.89	26.485	-1.336	7.22E-03
IGHV3-15	ENSG0000	0.45	1.995	-1.046	6.26E-03
PLCH2	ENSG0000	0.57	2.5	-1.157	6.00E-03
IGLC2	ENSG0000	39.769	93.769	-1.217	5.45E-03
IGKV1-27	ENSG0000	0.4	1.94	-1.07	5.39E-03
AC005943	ENSG0000	2.24	0	1.696	5.01E-03
IGKV1D-3	ENSG0000	3.94	11.967	-1.392	4.79E-03
SLC3A1	ENSG0000	0.66	2.48	-1.068	4.67E-03
IGHV3-33	ENSG0000	0.74	2.745	-1.106	4.21E-03
GOLGA8B	ENSG0000	7.7	19.393	-1.229	3.83E-03
IGKV1-5	ENSG0000	1.45	5.679	-1.447	3.65E-03
CLASRP	ENSG0000	20.34	45.469	-1.123	2.99E-03
IGHG4	ENSG0000	4.9	13.65	-1.312	2.88E-03
GSTM1	ENSG0000	1.28	70.601	-4.973	2.84E-03
CACNA1h	ENSG0000	1.51	5.3	-1.328	2.70E-03
IGKV3-20	ENSG0000	6.35	17.302	-1.316	2.63E-03
IGKC	ENSG0000	145.713	295.005	-1.013	2.58E-03
IGKV3-11	ENSG0000	8.89	19.145	-1.026	2.15E-03
IGHV5-51	ENSG0000	0.64	2.455	-1.075	1.92E-03
U91328.1	ENSG0000	1.96	0	1.566	1.88E-03
CYP2B7P	ENSG0000	5.24	14.939	-1.353	1.85E-03
UGT2B17	ENSG0000	0.75	3.04	-1.207	1.79E-03
IGKV1-9	ENSG0000	0.57	2.357	-1.097	1.74E-03
LINC0096	ENSG0000	15.97	33.294	-1.015	1.63E-03
RP11-361	ENSG0000	20.53	65.36	-1.624	1.61E-03
IGLC3	ENSG0000	24.69	52.18	-1.05	1.49E-03
IGLV1-40	ENSG0000	1.33	6.649	-1.715	1.26E-03
IGKV1-33	ENSG0000	1.17	5.51	-1.585	1.25E-03
AP003391.	ENSG0000	2.06	0	1.614	1.08E-03
IGLV3-25	ENSG0000	0.84	3.975	-1.435	9.68E-04
RP11-115	ENSG0000	1.93	7.789	-1.585	9.26E-04
APOA2	ENSG0000	31278.85	10880.07	1.523	9.24E-04
ACAP3	ENSG0000	7.86	17.3	-1.046	7.95E-04
CYP21A1F	ENSG0000	2.25	6.15	-1.137	7.39E-04
MUC3A	ENSG0000	1.62	5.13	-1.226	5.18E-04
MALAT1	ENSG0000	13.79	31.885	-1.153	4.83E-04
SERPIND1	ENSG0000	344.389	146.027	1.232	4.45E-04
IGHG2	ENSG0000	10.09	28.674	-1.42	3.72E-04
IGHV2-5	ENSG0000	0.23	1.725	-1.148	3.21E-04
FAM193B	ENSG0000	17.13	46.951	-1.403	3.06E-04
HEPACAM	ENSG0000	0.07	1.355	-1.138	2.67E-04
IGKV2-28	ENSG0000	2.52	8.89	-1.49	2.38E-04
CYP1A1	ENSG0000	0.97	4.71	-1.535	1.72E-04
RP11-345.	ENSG0000	2.12	0	1.642	1.56E-04
RP11-238	ENSG0000	0.14	2.04	-1.415	1.53E-04
ENGASE	ENSG0000	5.9	13.544	-1.076	1.47E-04
KRTCAP3	ENSG0000	1.41	6.48	-1.634	1.47E-04
IGLV1-51	ENSG0000	1.47	5.9	-1.482	1.29E-04
XXbac-BP	ENSG0000	5.75	1.324	1.538	9.66E-05
IGLV2-11	ENSG0000	1.12	4.605	-1.403	9.43E-05
AKR1C2	ENSG0000	299.123	125.148	1.25	8.98E-05
RP11-369.	ENSG0000	1.73	0.33	1.038	8.97E-05
CBS	ENSG0000	4.02	13.905	-1.57	8.68E-05
FST	ENSG0000	51.929	24.758	1.039	7.56E-05
RNF185-A	ENSG0000	3.09	0.212	1.755	6.30E-05

PRAP1	ENSG0000	676.102	259.618	1.377	4.48E-05
LY6G5B	ENSG0000	1.49	4.07	-1.026	3.60E-05
WASH5P	ENSG0000	5.2	12.53	-1.126	3.52E-05
SERPINE1	ENSG0000	20.831	46.29	-1.115	3.42E-05
CCDC84	ENSG0000	6.33	15.905	-1.206	2.85E-05
UGT1A6	ENSG0000	70.37	21.275	1.68	2.54E-05
TTTY14	ENSG0000	0.41	2.95	-1.486	2.34E-05
FNBP4	ENSG0000	9.22	20.065	-1.043	2.32E-05
RP11-119	ENSG0000	4.61	10.829	-1.076	2.11E-05
CLDN2	ENSG0000	5.48	16.855	-1.462	1.92E-05
HLA-DRB	ENSG0000	14.2	5.88	1.144	1.50E-05
BAMBI	ENSG0000	13.81	6.22	1.036	1.44E-05
CYP3A5	ENSG0000	100.148	212.607	-1.078	1.23E-05
CCL21	ENSG0000	7.73	17.895	-1.114	1.21E-05
SCD	ENSG0000	205.457	85.176	1.26	1.12E-05
FAM151A	ENSG0000	0.45	2.274	-1.175	1.09E-05
LENG8	ENSG0000	19.07	47.91	-1.285	1.03E-05
SRRM2	ENSG0000	123.442	265.349	-1.098	9.48E-06
HERC2P2	ENSG0000	4.35	13.429	-1.431	8.98E-06
CLDN10	ENSG0000	0.1	1.76	-1.327	6.13E-06
RNU4-2	ENSG0000	13.67	5.598	1.153	5.34E-06
GSDMB	ENSG0000	9.1	23.562	-1.282	5.33E-06
C2orf54	ENSG0000	6.44	2.305	1.171	4.81E-06
ARGLU1	ENSG0000	17.771	38.953	-1.09	4.10E-06
IGLV2-8	ENSG0000	0.55	2.785	-1.288	3.27E-06
ASMTL-A	ENSG0000	3.23	11.1	-1.516	3.17E-06
IGLV2-14	ENSG0000	2.14	11.483	-1.991	2.77E-06
MGP	ENSG0000	34.7	15.225	1.138	2.51E-06
LGALS4	ENSG0000	224.27	46.12	2.257	2.48E-06
EMP1	ENSG0000	7.72	3.295	1.022	2.12E-06
MME	ENSG0000	0.91	7.135	-2.091	2.01E-06
AMN	ENSG0000	9.84	21.844	-1.075	1.73E-06
CCL19	ENSG0000	2.7	8.89	-1.418	1.65E-06
NGFRAP1	ENSG0000	82.928	34.27	1.251	1.48E-06
SPINK1	ENSG0000	187.155	23.855	2.92	1.47E-06
RP5-940J	ENSG0000	20.359	2.015	2.825	1.22E-06
CNTFR	ENSG0000	0.49	3.555	-1.612	1.21E-06
SUMO2P1	ENSG0000	1.16	0	1.111	1.18E-06
WBP5	ENSG0000	20.89	9.55	1.053	1.10E-06
ARSE	ENSG0000	49.9	23	1.085	9.66E-07
EBF4	ENSG0000	2.55	6.54	-1.087	8.38E-07
GBP7	ENSG0000	6.71	14.798	-1.035	7.89E-07
CDK11A	ENSG0000	11.85	26.269	-1.086	7.63E-07
CYP21A2	ENSG0000	5.8	14.85	-1.221	6.93E-07
SIK1	ENSG0000	1.57	4.18	-1.011	6.29E-07
GSTT2B	ENSG0000	3.38	10.82	-1.432	6.07E-07
CPT1B	ENSG0000	5.21	13.954	-1.268	5.88E-07
PLA2G2A	ENSG0000	23.811	207.166	-3.069	5.77E-07
HOOK2	ENSG0000	8.22	22.89	-1.374	5.02E-07
SLC17A9	ENSG0000	29.769	90.333	-1.57	4.91E-07
FBLN1	ENSG0000	8.62	3.655	1.047	4.88E-07
AC009120	ENSG0000	1.99	5.625	-1.148	4.51E-07
VEGFA	ENSG0000	53.05	116.073	-1.115	3.43E-07
TMEM184	ENSG0000	5.88	13.27	-1.052	3.29E-07
IRF6	ENSG0000	21.24	9.51	1.081	3.04E-07
MRC1	ENSG0000	1.92	5.135	-1.071	2.95E-07
SLC10A1	ENSG0000	33.819	69.567	-1.019	2.91E-07
PNMA6A	ENSG0000	9.82	4.305	1.028	2.33E-07

APOA4	ENSG0000	3.03	26.19	-2.754	1.79E-07
RP4-631H	ENSG0000	0	1.5	-1.322	1.76E-07
JCHAIN	ENSG0000	2.83	8.089	-1.247	1.53E-07
TAF1C	ENSG0000	6.94	14.985	-1.01	1.46E-07
RP11-326	ENSG0000	0.96	7.133	-2.053	1.31E-07
FAM160B2	ENSG0000	9.28	22.195	-1.174	1.12E-07
VIL1	ENSG0000	14.91	4.055	1.654	1.06E-07
NPIPB3	ENSG0000	8.82	24.769	-1.392	1.05E-07
RP11-609	ENSG0000	1.08	3.6	-1.145	9.69E-08
RP11-496	ENSG0000	0.65	2.6	-1.125	9.34E-08
AKR1C1	ENSG0000	481.068	212.636	1.174	9.13E-08
LOXL4	ENSG0000	3.58	0.72	1.413	6.30E-08
RP11-295	ENSG0000	3.58	10.585	-1.339	6.20E-08
SYNGR1	ENSG0000	6.5	2.715	1.014	5.92E-08
FGL1	ENSG0000	714.652	1526.116	-1.093	5.42E-08
AGAP4	ENSG0000	2.28	5.63	-1.015	5.30E-08
MAPK8IP3	ENSG0000	5.62	18.72	-1.575	5.30E-08
IGKV2-24	ENSG0000	0.37	2.074	-1.166	5.29E-08
RP11-34P	ENSG0000	0.85	2.82	-1.046	5.09E-08
STAG3L5P	ENSG0000	5.22	13.125	-1.183	5.08E-08
STAG3L5P	ENSG0000	14.78	42.15	-1.451	4.67E-08
PTOV1-AS	ENSG0000	2.39	6.895	-1.22	4.51E-08
LTB4R	ENSG0000	2.01	5.365	-1.08	3.40E-08
RP11-49K	ENSG0000	3.59	1.135	1.104	3.04E-08
LGALS3BP	ENSG0000	160.385	54.264	1.546	2.76E-08
ANXA13	ENSG0000	7.52	2.45	1.304	2.16E-08
CLDN7	ENSG0000	35.24	14.474	1.228	2.16E-08
FAM83A-	ENSG0000	0.45	4.95	-2.037	2.15E-08
FASN	ENSG0000	217.896	108.112	1.004	2.11E-08
ACP5	ENSG0000	22.77	9.84	1.133	2.02E-08
PNISR	ENSG0000	11.81	31.325	-1.335	2.01E-08
HLA-DQA	ENSG0000	2.19	0.425	1.163	1.62E-08
OSGIN1	ENSG0000	89.492	40.993	1.108	1.48E-08
S100P	ENSG0000	6.05	2.36	1.069	1.30E-08
SUSD4	ENSG0000	3.92	1.295	1.1	1.23E-08
SULT1E1	ENSG0000	1.41	5.65	-1.464	1.08E-08
PLXNB1	ENSG0000	30.89	67.29	-1.099	9.51E-09
PTPRS	ENSG0000	0.58	2.84	-1.281	8.57E-09
OAT	ENSG0000	9.78	26.915	-1.373	7.28E-09
HEPN1	ENSG0000	0.21	5.295	-2.379	5.40E-09
COX6A2	ENSG0000	2.46	6.19	-1.055	4.48E-09
TRBC2	ENSG0000	11.13	5.035	1.007	3.92E-09
TM4SF5	ENSG0000	200.31	92.641	1.104	3.90E-09
VNN1	ENSG0000	17.96	39.013	-1.077	3.43E-09
PRSS8	ENSG0000	3	10.11	-1.474	3.16E-09
IP6K3	ENSG0000	0.36	2.394	-1.319	3.14E-09
AKAP17A	ENSG0000	10.16	25.474	-1.246	3.02E-09
FOLR2	ENSG0000	10.76	4.165	1.187	2.82E-09
MUC6	ENSG0000	0.02	1.58	-1.339	2.73E-09
EFHD1	ENSG0000	5.26	17.155	-1.536	2.61E-09
COL1A1	ENSG0000	28.3	10.504	1.349	2.14E-09
ITIH2	ENSG0000	623.739	212.342	1.55	1.81E-09
LINC0132	ENSG0000	0.46	2.05	-1.063	1.73E-09
COL1A2	ENSG0000	29.91	12.124	1.236	1.68E-09
INHBB	ENSG0000	10.18	4.304	1.076	1.55E-09
FGB	ENSG0000	3258.516	6850.767	-1.072	1.51E-09
SMOX	ENSG0000	4.76	1.82	1.03	1.40E-09
C1QA	ENSG0000	81.492	39.245	1.035	1.34E-09

RP11-115	ENSG0000	0.56	3.09	-1.391	1.34E-09
MT1P3	ENSG0000	0	1.41	-1.269	1.22E-09
RP4-669L	ENSG0000	3.09	8.316	-1.188	1.19E-09
HLA-DQA	ENSG0000	12.49	3.505	1.582	1.17E-09
TMED3	ENSG0000	20.71	6.7	1.495	1.13E-09
WDR27	ENSG0000	2.94	8.715	-1.302	8.39E-10
GNMT	ENSG0000	30.38	90.336	-1.541	8.24E-10
CYP7A1	ENSG0000	10.65	1.939	1.987	7.80E-10
CD36	ENSG0000	16.32	7.178	1.083	7.68E-10
OLFM2	ENSG0000	31.021	12.169	1.282	6.92E-10
SLC27A5	ENSG0000	248.413	497.947	-1	5.86E-10
CCDC80	ENSG0000	4.3	1.31	1.198	5.83E-10
AGER	ENSG0000	1.41	4.07	-1.073	5.63E-10
PHLDA3	ENSG0000	6.77	2.53	1.138	4.69E-10
HLA-DRB	ENSG0000	66.431	27.555	1.24	4.58E-10
RP11-274	ENSG0000	4.96	12.679	-1.199	4.58E-10
YJEFN3	ENSG0000	0.55	2.535	-1.189	4.56E-10
DHFRP1	ENSG0000	2.31	0.635	1.018	4.52E-10
COL27A1	ENSG0000	6.03	16.685	-1.331	4.27E-10
IGF2	ENSG0000	10.29	89.75	-3.007	3.53E-10
DDX3Y	ENSG0000	3.68	8.605	-1.037	3.27E-10
RP4-568C	ENSG0000	1.02	3.25	-1.073	3.07E-10
IFI6	ENSG0000	95.742	36.279	1.376	2.97E-10
CH17-13I	ENSG0000	1.3	0.105	1.058	2.82E-10
MLXIPL	ENSG0000	98.7	263.982	-1.41	2.76E-10
KRT23	ENSG0000	2.85	0.21	1.67	2.75E-10
FABP4	ENSG0000	7.47	2.645	1.216	2.62E-10
ALDH1A1	ENSG0000	458.284	217.994	1.068	2.39E-10
AFG3L1P	ENSG0000	3.2	8.22	-1.134	2.33E-10
CCNL2	ENSG0000	33.029	101.006	-1.584	1.99E-10
NKTR	ENSG0000	4.61	13.3	-1.35	1.96E-10
IGHM	ENSG0000	8.76	27.634	-1.553	1.81E-10
KIAA0895	ENSG0000	2.04	7.16	-1.424	1.66E-10
B3GNT3	ENSG0000	2.33	0.495	1.155	1.36E-10
ARSEP1	ENSG0000	2.5	0.09	1.683	1.24E-10
MZF1	ENSG0000	4.09	11.175	-1.258	1.24E-10
NRG1	ENSG0000	0.45	2.87	-1.416	1.20E-10
DTX4	ENSG0000	3.77	8.57	-1.005	1.15E-10
AC005943	ENSG0000	1.38	0	1.251	8.76E-11
RASD1	ENSG0000	14.23	43.902	-1.56	8.63E-11
SRSF11	ENSG0000	25.31	68.614	-1.404	8.39E-11
TMEM45B	ENSG0000	29.9	8.1	1.764	7.97E-11
PODN	ENSG0000	1.39	3.865	-1.025	7.70E-11
FAM150B	ENSG0000	0.13	1.28	-1.013	5.92E-11
CTSS	ENSG0000	37.281	13.98	1.354	5.87E-11
GLUL	ENSG0000	323.024	144.037	1.16	5.79E-11
LPIN3	ENSG0000	4.75	13.924	-1.376	5.67E-11
CLGN	ENSG0000	2.2	0.6	1	5.64E-11
B3GAT1	ENSG0000	0.11	1.63	-1.245	5.52E-11
SCPEP1	ENSG0000	25.849	11.89	1.059	5.18E-11
ADH1B	ENSG0000	360.135	884.396	-1.294	5.15E-11
PNN	ENSG0000	14.91	32.62	-1.079	5.10E-11
PYCR1	ENSG0000	6.32	2.035	1.27	4.77E-11
HSD11B1	ENSG0000	76.531	189.827	-1.299	4.75E-11
ASPDH	ENSG0000	33.751	71.634	-1.064	4.55E-11
SLX1B-SU	ENSG0000	0.99	3.245	-1.093	4.32E-11
RP11-203	ENSG0000	1.67	4.945	-1.155	4.29E-11
FAM3B	ENSG0000	2.72	0.6	1.217	4.04E-11

CYP2A6	ENSG0000	132.862	592.285	-2.148	3.83E-11
PILRB	ENSG0000	7.6	26.129	-1.657	3.78E-11
SLC39A5	ENSG0000	52.399	108.891	-1.041	3.77E-11
FOSB	ENSG0000	1.62	4.38	-1.038	3.59E-11
DCDC2	ENSG0000	2.38	0.62	1.061	3.39E-11
HPX	ENSG0000	1194.589	2403.131	-1.008	3.35E-11
AGAP5	ENSG0000	1.11	3.235	-1.005	3.29E-11
LAPTM5	ENSG0000	28.489	13.69	1.005	3.00E-11
HLA-DPA1	ENSG0000	49.969	17.045	1.498	2.45E-11
ATF3	ENSG0000	14.94	36.796	-1.246	2.39E-11
ST14	ENSG0000	8.05	2.715	1.285	2.33E-11
AFP	ENSG0000	11.46	4.685	1.132	2.19E-11
CD5L	ENSG0000	1.35	3.725	-1.008	2.12E-11
ADAT2	ENSG0000	1.23	3.685	-1.071	1.97E-11
EGR1	ENSG0000	18.64	44.085	-1.199	1.97E-11
CTC-505C	ENSG0000	2.9	11.22	-1.648	1.94E-11
NEIL1	ENSG0000	8.76	20.924	-1.168	1.85E-11
LINC0100	ENSG0000	3.89	14.567	-1.671	1.84E-11
CCL18	ENSG0000	2.04	0.51	1.01	1.72E-11
RP11-977	ENSG0000	1.01	0	1.007	1.70E-11
SAA2-SAA	ENSG0000	2.75	17.052	-2.267	1.56E-11
BHMT	ENSG0000	58.582	197.176	-1.734	1.51E-11
BBOX1	ENSG0000	2.69	11.375	-1.746	1.48E-11
BEX2	ENSG0000	2.48	0.685	1.046	1.26E-11
NEAT1	ENSG0000	124.507	481.719	-1.943	1.13E-11
SDS	ENSG0000	58.318	343.496	-2.538	1.03E-11
AKR1D1	ENSG0000	10.96	40.685	-1.801	1.01E-11
S100A4	ENSG0000	29.25	13.083	1.103	9.94E-12
SKAP1	ENSG0000	7.37	17.729	-1.162	9.64E-12
FAM127B	ENSG0000	33.109	15.435	1.053	8.59E-12
RP11-159	ENSG0000	1	3.94	-1.305	8.45E-12
TRNP1	ENSG0000	5.14	1.415	1.346	8.41E-12
LINC0101	ENSG0000	5.58	19.171	-1.616	8.20E-12
ALDH3A1	ENSG0000	2.17	0.46	1.119	7.82E-12
CGREF1	ENSG0000	9.8	4.105	1.081	7.12E-12
SLX1A-SU	ENSG0000	2.04	6.45	-1.293	6.72E-12
HPGD	ENSG0000	14.55	40.456	-1.415	6.59E-12
RP11-175	ENSG0000	1.41	4.86	-1.282	6.52E-12
C1RL-AS1	ENSG0000	1.81	5.113	-1.121	6.51E-12
HULC	ENSG0000	127.221	34.625	1.848	6.45E-12
FAM229A	ENSG0000	1.52	4.93	-1.235	6.43E-12
ACCS	ENSG0000	6.22	14.189	-1.073	6.11E-12
AC104534	ENSG0000	5.81	0.42	2.262	6.03E-12
IGHA2	ENSG0000	2.83	10.167	-1.544	5.71E-12
TRPM8	ENSG0000	4.06	10.085	-1.131	5.06E-12
NEU4	ENSG0000	9.57	24.385	-1.264	4.71E-12
SLC6A8	ENSG0000	3.29	1.05	1.065	4.62E-12
TGM2	ENSG0000	49.931	107.727	-1.094	3.91E-12
TRAC	ENSG0000	7.94	2.665	1.286	3.38E-12
AC132217	ENSG0000	21.11	365.062	-4.049	3.36E-12
GABBR1	ENSG0000	0.68	2.665	-1.125	3.18E-12
FXYD1	ENSG0000	79.34	237.14	-1.568	2.63E-12
S100A6	ENSG0000	69.07	28.38	1.254	2.49E-12
MTND2P2	ENSG0000	211.541	441.922	-1.059	2.46E-12
PHLDA2	ENSG0000	4.54	1.015	1.459	2.33E-12
ACOT12	ENSG0000	13.96	29.518	-1.029	2.17E-12
RP11-458	ENSG0000	1.31	3.715	-1.029	1.74E-12
HIST2H2A	ENSG0000	29.59	9.48	1.545	1.62E-12

CLRN3	ENSG0000	4	14.575	-1.639	1.60E-12
CYP2D7	ENSG0000	19.04	65.65	-1.734	1.53E-12
RBBP4	ENSG0000	30.969	14.58	1.037	1.25E-12
TPT1-AS1	ENSG0000	3.42	8.05	-1.034	1.23E-12
MAGED1	ENSG0000	64.44	31.6	1.005	1.20E-12
LINC0010(	ENSG0000	0.56	2.26	-1.063	1.09E-12
SRC	ENSG0000	8.85	3.365	1.174	1.05E-12
SERPINA3	ENSG0000	370.748	888.913	-1.259	9.53E-13
G0S2	ENSG0000	46.051	133.107	-1.511	9.21E-13
UCP2	ENSG0000	14.85	5.555	1.274	9.18E-13
ISG15	ENSG0000	71.55	30.819	1.189	9.07E-13
BCHE	ENSG0000	17.321	38.95	-1.125	8.65E-13
RP11-510	ENSG0000	0.27	1.56	-1.011	8.48E-13
F5	ENSG0000	206.614	92.446	1.152	8.08E-13
SOCS3	ENSG0000	6.84	20.916	-1.483	7.50E-13
PLA2G4B	ENSG0000	1.46	4.7	-1.212	7.46E-13
S100A11	ENSG0000	77.321	36.376	1.067	7.15E-13
RP11-42C	ENSG0000	4.66	11.395	-1.131	6.70E-13
PIGY	ENSG0000	8.94	0.005	3.306	6.41E-13
COX7CP1	ENSG0000	0	1.532	-1.34	6.12E-13
BCL6	ENSG0000	10.78	24.681	-1.124	5.88E-13
APOA5	ENSG0000	97.267	211.834	-1.115	5.78E-13
CD24	ENSG0000	50.85	6.745	2.743	5.43E-13
CTB-89H1	ENSG0000	1.4	3.94	-1.041	5.23E-13
TNFRSF21	ENSG0000	5.45	2.02	1.095	5.04E-13
AP006285.	ENSG0000	0.47	2.525	-1.262	4.77E-13
CKB	ENSG0000	26.379	10.115	1.301	4.73E-13
NPIPB4	ENSG0000	4.31	16.34	-1.707	4.23E-13
LRP5L	ENSG0000	2.81	7.535	-1.164	3.77E-13
PARVB	ENSG0000	16.31	6.394	1.227	3.72E-13
AF064858.	ENSG0000	0.81	3.285	-1.243	3.50E-13
TPPP3	ENSG0000	4.09	1.46	1.049	3.36E-13
PEG10	ENSG0000	5.21	0.71	1.861	3.12E-13
APOC2	ENSG0000	2260.615	475.779	2.246	3.11E-13
SYT7	ENSG0000	6.73	21.18	-1.521	2.42E-13
ADH4	ENSG0000	154.032	549.147	-1.827	2.35E-13
MYO7A	ENSG0000	4.79	10.68	-1.012	2.15E-13
AGAP6	ENSG0000	3.38	12.338	-1.607	1.98E-13
KDELR3	ENSG0000	9.84	3.98	1.122	1.90E-13
UNC13D	ENSG0000	1.63	5.01	-1.192	1.65E-13
CRP	ENSG0000	251.671	2792.323	-3.467	1.62E-13
LCN2	ENSG0000	38.101	5.525	2.583	1.58E-13
SLC25A18	ENSG0000	21.47	46.775	-1.088	1.48E-13
TEAD2	ENSG0000	7.46	3.145	1.029	1.47E-13
BGN	ENSG0000	56.79	133.176	-1.215	1.42E-13
CCDC64	ENSG0000	4.25	1.51	1.065	1.30E-13
CES3	ENSG0000	7.32	16.224	-1.05	1.23E-13
CYP3A43	ENSG0000	0.75	2.875	-1.147	1.02E-13
EEF1A2	ENSG0000	7.09	0.45	2.48	9.75E-14
IGF1	ENSG0000	4.77	12.475	-1.224	9.57E-14
SELM	ENSG0000	33.981	12.185	1.408	9.10E-14
APOE	ENSG0000	5880.405	2771.689	1.085	8.27E-14
ORM2	ENSG0000	1430.198	3502.573	-1.292	8.12E-14
CYP2A7	ENSG0000	1.97	29.83	-3.376	7.48E-14
LCN12	ENSG0000	6.83	17.9	-1.271	7.33E-14
LY96	ENSG0000	8.45	3.375	1.111	7.04E-14
RP11-66N	ENSG0000	1.31	5.55	-1.504	6.60E-14
CXCL9	ENSG0000	4.18	0.765	1.553	5.77E-14

TMEM45A	ENSG0000	11.38	32.179	-1.422	4.51E-14
HAL	ENSG0000	20.48	54.784	-1.377	3.89E-14
OLFML3	ENSG0000	2.83	8.29	-1.278	3.70E-14
IFITM10	ENSG0000	1.82	8.635	-1.773	3.62E-14
RP11-452	ENSG0000	7.38	2.75	1.16	3.59E-14
KCNK5	ENSG0000	1.67	4.375	-1.009	3.47E-14
SMIM11	ENSG0000	4.77	1.32	1.314	3.18E-14
C8orf46	ENSG0000	1.62	5.51	-1.313	2.86E-14
SULT1B1	ENSG0000	1.05	3.175	-1.026	2.81E-14
HLA-DPB1	ENSG0000	59.821	17.4	1.725	2.74E-14
CDA	ENSG0000	15.42	32.819	-1.042	2.27E-14
LINC0065	ENSG0000	1.39	4.703	-1.255	2.20E-14
RP5-890E	ENSG0000	4.32	0	2.411	2.20E-14
EEF1A1P5	ENSG0000	63.341	26.253	1.239	2.14E-14
TRPV4	ENSG0000	0.77	2.83	-1.114	2.01E-14
LINC0137	ENSG0000	0.53	6.555	-2.304	1.99E-14
USP9Y	ENSG0000	0.4	2.55	-1.342	1.78E-14
POLR2J2	ENSG0000	5.5	2.205	1.02	1.64E-14
HLA-DRB	ENSG0000	4.28	0.965	1.426	1.57E-14
FXYD5	ENSG0000	29.699	13.652	1.067	1.43E-14
REC8	ENSG0000	2.28	7.14	-1.311	1.43E-14
ERICH5	ENSG0000	18.92	3.41	2.175	1.43E-14
NAMPT	ENSG0000	38.001	86.288	-1.162	1.32E-14
S100A9	ENSG0000	11.29	46.889	-1.962	1.32E-14
TBX15	ENSG0000	2.27	7.885	-1.442	1.26E-14
LINC0159	ENSG0000	0.99	6.505	-1.915	1.21E-14
*Metazoa_*	ENSG0000	343.769	129.247	1.404	1.20E-14
ARRDC2	ENSG0000	15.5	7.085	1.029	1.11E-14
SEC14L4	ENSG0000	2.04	5.92	-1.187	1.11E-14
NDUFA6-	ENSG0000	5.28	12.819	-1.138	9.18E-15
RP1-241P	ENSG0000	3.77	0.74	1.455	9.14E-15
EVL	ENSG0000	25.86	54.749	-1.054	8.84E-15
TNFRSF12	ENSG0000	46.019	15.247	1.533	8.77E-15
ELOVL2	ENSG0000	17.6	4.335	1.802	7.73E-15
RUFY3	ENSG0000	6.88	14.95	-1.017	7.58E-15
AMDHD1	ENSG0000	23.48	51.479	-1.1	6.39E-15
ANK3	ENSG0000	1.01	3.435	-1.142	6.39E-15
GSN	ENSG0000	85.882	37.655	1.168	6.32E-15
AP1M2	ENSG0000	2.83	0.56	1.296	4.23E-15
CITED2	ENSG0000	16.549	36.75	-1.105	3.51E-15
CFHR3	ENSG0000	33.541	174.664	-2.346	3.33E-15
SNRPGP1	ENSG0000	1.81	5.694	-1.252	3.22E-15
RP11-423	ENSG0000	0.19	1.485	-1.062	2.96E-15
IGF2BP2	ENSG0000	2.68	0.68	1.131	2.89E-15
BCL3	ENSG0000	31.12	75.011	-1.243	2.34E-15
SPP1	ENSG0000	75.378	8.524	3.004	2.34E-15
GDF15	ENSG0000	44.51	15.795	1.438	1.80E-15
RAMP1	ENSG0000	103.679	31.471	1.689	1.78E-15
NPC1L1	ENSG0000	4.68	13.435	-1.346	1.66E-15
UBE2Q2P	ENSG0000	655.975	307.128	1.092	1.39E-15
PPP1R3C	ENSG0000	10.36	28.32	-1.368	1.33E-15
LRG1	ENSG0000	121.229	339.166	-1.477	1.29E-15
CACNB2	ENSG0000	0.74	2.575	-1.039	1.27E-15
LTB	ENSG0000	7.99	2.18	1.499	1.25E-15
RPL39L	ENSG0000	5.42	1.46	1.384	1.23E-15
CYP4A11	ENSG0000	108.217	299.559	-1.46	1.21E-15
SLC46A3	ENSG0000	11.35	23.705	-1	1.20E-15
MMP9	ENSG0000	3.01	0.8	1.156	1.18E-15

WASH7P	ENSG0000	1.2	4.41	-1.298	1.11E-15
CMTM7	ENSG0000	8.1	3.55	1	1.06E-15
ZNF160	ENSG0000	1.91	4.945	-1.031	1.05E-15
ADAMTS1	ENSG0000	3.15	7.81	-1.086	8.91E-16
TUBB2A	ENSG0000	60.17	28.979	1.029	8.62E-16
NPIPB5	ENSG0000	9.71	48.205	-2.2	8.53E-16
HLA-C	ENSG0000	499.658	223.828	1.155	7.73E-16
ISG20	ENSG0000	19.96	9.17	1.043	7.63E-16
TMEM97	ENSG0000	38.62	18.5	1.023	7.48E-16
GRAMD1A	ENSG0000	18.69	8.595	1.037	7.23E-16
C15orf48	ENSG0000	3.02	0.55	1.375	7.15E-16
EPO	ENSG0000	0.19	3.185	-1.814	6.95E-16
AZGP1	ENSG0000	569.518	1221.805	-1.1	5.97E-16
TNFSF14	ENSG0000	2.84	14.71	-2.032	5.52E-16
GGT5	ENSG0000	8.17	19.87	-1.186	5.36E-16
TESC	ENSG0000	7.24	2.13	1.397	5.28E-16
SOX4	ENSG0000	3.07	0.71	1.251	5.22E-16
TM4SF1	ENSG0000	41.469	14.024	1.499	5.03E-16
PRKDC	ENSG0000	22.389	9.685	1.13	4.97E-16
H3F3AP4	ENSG0000	144.256	34.374	2.038	4.70E-16
GREM2	ENSG0000	0.93	6.41	-1.941	4.38E-16
PRELP	ENSG0000	1.25	4.32	-1.241	4.30E-16
MAMDC4	ENSG0000	2.66	11.405	-1.761	4.27E-16
TAT	ENSG0000	98.25	345.525	-1.804	4.06E-16
RP11-231	ENSG0000	6.94	27.937	-1.866	3.68E-16
DPP4	ENSG0000	19.36	5.345	1.682	3.61E-16
HLA-DQB	ENSG0000	22.92	5.189	1.95	3.59E-16
TM7SF2	ENSG0000	103.407	41.026	1.313	3.57E-16
HLA-DRA	ENSG0000	171.468	42.669	1.982	3.47E-16
RP11-499	ENSG0000	2.1	7.765	-1.499	3.20E-16
IGHA1	ENSG0000	21.08	103.5	-2.243	3.01E-16
EXOC3L4	ENSG0000	17.59	43.365	-1.255	2.97E-16
ADAMTS2	ENSG0000	1.37	4.445	-1.2	2.95E-16
HSD17B1	ENSG0000	6.73	74.827	-3.294	2.68E-16
ACSM1	ENSG0000	8.56	1.085	2.197	2.43E-16
MFAP4	ENSG0000	3.46	14.005	-1.75	2.29E-16
AUTS2	ENSG0000	3.86	9.375	-1.094	2.28E-16
SPTBN2	ENSG0000	8.04	18.735	-1.126	2.26E-16
SMO	ENSG0000	16.5	7.11	1.11	2.25E-16
CD3D	ENSG0000	4.12	1.295	1.158	2.24E-16
PTGIS	ENSG0000	0.37	1.815	-1.039	1.91E-16
CLDN15	ENSG0000	24.411	8.805	1.374	1.73E-16
AP000355	ENSG0000	0.63	2.28	-1.009	1.62E-16
PPDPF	ENSG0000	190.982	72.223	1.391	1.61E-16
STAT1	ENSG0000	38.62	17.54	1.096	1.35E-16
CYP3A4	ENSG0000	31.139	386.278	-3.591	1.22E-16
HLA-B	ENSG0000	788.298	281.004	1.485	1.15E-16
RP11-680	ENSG0000	3.77	1.305	1.049	1.13E-16
PDZK1IP1	ENSG0000	15.74	1.505	2.74	1.12E-16
PDGFRB	ENSG0000	10.1	4.26	1.077	1.10E-16
MT-ND5	ENSG0000	1410.997	3065.91	-1.119	9.28E-17
MAP2	ENSG0000	3.05	0.965	1.043	8.47E-17
TRIM16L	ENSG0000	7.1	2.925	1.045	6.52E-17
MEG3	ENSG0000	2.21	22.203	-2.854	6.21E-17
HLA-DMB	ENSG0000	14.22	4.74	1.407	5.56E-17
MTND4P1	ENSG0000	39.65	0.81	4.489	5.49E-17
PAN2	ENSG0000	13.25	46.264	-1.73	5.37E-17
LINC0084	ENSG0000	5.59	37.984	-2.565	5.09E-17

GPNMB	ENSG0000	12.74	3.525	1.602	4.77E-17
UGT1A10	ENSG0000	1.21	0.1	1.007	4.13E-17
SOX9	ENSG0000	4.95	1	1.573	3.90E-17
HP	ENSG0000	8497.954	26421.96	-1.636	2.98E-17
RP11-328	ENSG0000	3.02	13.645	-1.865	2.86E-17
IL4R	ENSG0000	17.09	36.155	-1.038	2.59E-17
HCST	ENSG0000	12.42	5.039	1.152	2.51E-17
TAX1BP3	ENSG0000	26.981	11.99	1.107	2.37E-17
EPHX1	ENSG0000	1532.848	505.37	1.599	2.23E-17
PALM3	ENSG0000	7.61	18.025	-1.144	2.18E-17
GGH	ENSG0000	121.852	51.186	1.235	1.99E-17
SPON2	ENSG0000	94.851	45.069	1.057	1.97E-17
PLP2	ENSG0000	24.319	8.734	1.379	1.90E-17
DPH1	ENSG0000	10.36	22.265	-1.034	1.87E-17
LGALS9	ENSG0000	20.79	8.12	1.257	1.77E-17
OGDHL	ENSG0000	18.45	41.286	-1.12	1.77E-17
TDO2	ENSG0000	76.478	213.996	-1.472	1.71E-17
NR4A1	ENSG0000	8.57	25.707	-1.481	1.62E-17
RP11-166	ENSG0000	0.87	3.453	-1.252	1.57E-17
FAM134B	ENSG0000	2.32	9.124	-1.609	1.44E-17
DNHD1	ENSG0000	1.45	6.529	-1.62	1.38E-17
CYP2C9	ENSG0000	134.596	470.368	-1.798	1.33E-17
EPOR	ENSG0000	2.57	7.27	-1.212	1.33E-17
TMEM132	ENSG0000	2.42	0.575	1.119	1.31E-17
PIK3IP1	ENSG0000	7	2.74	1.097	1.30E-17
TRIM66	ENSG0000	0.84	3.18	-1.184	1.30E-17
RP11-394	ENSG0000	0.48	2.335	-1.172	1.21E-17
GPD1	ENSG0000	15.39	46.359	-1.531	1.03E-17
6-Sep	ENSG0000	22.06	9.535	1.13	9.56E-18
FNDC4	ENSG0000	22.841	53.096	-1.182	9.26E-18
TUBA1A	ENSG0000	15.45	6.205	1.191	8.89E-18
RP11-386	ENSG0000	69.622	32.095	1.094	7.95E-18
AC009963	ENSG0000	12.75	43.499	-1.694	7.81E-18
FPR1	ENSG0000	0.53	2.29	-1.105	7.81E-18
GOLM1	ENSG0000	22.4	5.695	1.805	7.37E-18
PTGFRN	ENSG0000	10.95	4.845	1.032	4.96E-18
RP3-508I1	ENSG0000	0	1.745	-1.457	4.80E-18
MBL2	ENSG0000	12.81	37.715	-1.487	4.36E-18
EPHX2	ENSG0000	47.891	106.244	-1.133	3.89E-18
GLYAT	ENSG0000	35.37	138.764	-1.942	3.66E-18
MARCKSL	ENSG0000	29.07	13.709	1.032	3.51E-18
LINC0066	ENSG0000	2.16	0.485	1.089	3.09E-18
GJA1	ENSG0000	4.23	1.415	1.115	3.08E-18
RPL41P2	ENSG0000	37.139	17.225	1.065	2.90E-18
C12orf75	ENSG0000	6.37	1.335	1.658	2.87E-18
TM4SF4	ENSG0000	430.001	95.683	2.156	2.86E-18
MT1DP	ENSG0000	0.59	4.235	-1.719	2.76E-18
KCTD17	ENSG0000	4.51	1.625	1.07	2.73E-18
ANXA10	ENSG0000	10.59	34.34	-1.608	2.59E-18
CTD-254	ENSG0000	125.4	57.014	1.124	2.20E-18
RPL39P3	ENSG0000	319.151	110.798	1.518	2.15E-18
RP11-419	ENSG0000	2.46	11.16	-1.813	2.07E-18
HMGN4	ENSG0000	13.39	5.36	1.178	1.88E-18
C10orf35	ENSG0000	3.84	1.375	1.027	1.80E-18
GOLGA6L	ENSG0000	0.93	3.465	-1.21	1.80E-18
MT1A	ENSG0000	15.19	81.693	-2.353	1.74E-18
GPSM1	ENSG0000	3.67	1.165	1.109	1.63E-18
GBA3	ENSG0000	8.22	31.277	-1.808	1.37E-18

IGSF9	ENSG0000	1.64	7.91	-1.755	1.31E-18
ATP1B3	ENSG0000	21.14	8.2	1.267	1.30E-18
CHST4	ENSG0000	0.03	1.585	-1.327	1.28E-18
SH3BP5-A	ENSG0000	0.9	3.05	-1.092	1.24E-18
DNAJC12	ENSG0000	6.66	21.085	-1.528	1.18E-18
RP11-715	ENSG0000	0	1.02	-1.014	1.02E-18
PHYHD1	ENSG0000	14.71	34.515	-1.177	9.58E-19
PPP1R3B	ENSG0000	10.71	24.785	-1.139	9.18E-19
SOX9-AS1	ENSG0000	2.87	0.72	1.17	9.13E-19
ABCB6	ENSG0000	39.371	18.615	1.041	8.28E-19
CTD-2619	ENSG0000	1.95	6.439	-1.334	7.86E-19
ERRFI1	ENSG0000	67.672	160.379	-1.233	7.82E-19
MAT1A	ENSG0000	220.907	484.649	-1.13	7.57E-19
CCL20	ENSG0000	22.42	1.5	3.228	7.05E-19
PBLD	ENSG0000	29.59	69.384	-1.202	6.28E-19
S100A13	ENSG0000	75.29	27.839	1.403	6.17E-19
DOCK5	ENSG0000	2.04	5.98	-1.199	5.82E-19
SCARNA1	ENSG0000	0.37	1.865	-1.064	5.67E-19
TUBA1C	ENSG0000	72.028	27.1	1.378	5.52E-19
FTL	ENSG0000	11550.76	5723.571	1.013	5.51E-19
CTSH	ENSG0000	163.46	77.63	1.065	5.46E-19
SLC29A1	ENSG0000	46.571	22.445	1.021	4.68E-19
SNRPGP1	ENSG0000	0.25	1.765	-1.145	4.66E-19
LINC0017	ENSG0000	1.05	3.635	-1.177	4.56E-19
SECTM1	ENSG0000	5.34	2.085	1.039	4.35E-19
C8A	ENSG0000	73.338	173.446	-1.231	4.02E-19
RP11-114	ENSG0000	17.64	3.005	2.219	4.02E-19
AGAP9	ENSG0000	2.16	10.75	-1.895	3.89E-19
IFI27	ENSG0000	145.209	26.64	2.403	3.73E-19
SULT1C2	ENSG0000	4.09	0.35	1.915	3.66E-19
PSMC1P1	ENSG0000	13.25	5.405	1.154	3.47E-19
NREP	ENSG0000	17.25	7.315	1.134	3.45E-19
PDGFRA	ENSG0000	0.92	3.855	-1.338	3.43E-19
ORM1	ENSG0000	4575.773	15121.36	-1.724	3.35E-19
RTN2	ENSG0000	5.1	1.68	1.187	3.26E-19
RP11-316	ENSG0000	78.699	24.778	1.628	2.61E-19
C1QTNF5	ENSG0000	11.43	5.08	1.032	2.00E-19
VIM	ENSG0000	130.273	52.559	1.293	1.76E-19
C1orf106	ENSG0000	1.81	0.26	1.157	1.60E-19
RNA5SP2	ENSG0000	0	7.302	-3.054	1.59E-19
CYP4A22	ENSG0000	23.29	77.329	-1.689	1.47E-19
B3GNT5	ENSG0000	2.3	0.45	1.186	1.39E-19
GRAMD1	ENSG0000	2.24	5.925	-1.096	1.36E-19
TSPAN15	ENSG0000	5.6	2.11	1.086	1.23E-19
RP11-61N	ENSG0000	6.12	1.49	1.516	1.21E-19
AFM	ENSG0000	50.38	143.334	-1.49	1.15E-19
CTD-2537	ENSG0000	1.46	8.73	-1.984	1.04E-19
RP4-763G	ENSG0000	5.4	19.574	-1.685	9.76E-20
SLC43A2	ENSG0000	4.48	1.57	1.092	9.52E-20
AASS	ENSG0000	3.24	11.16	-1.52	9.03E-20
PLIN5	ENSG0000	18.75	68.379	-1.813	8.12E-20
PVT1	ENSG0000	6.01	1.035	1.784	7.25E-20
SLC13A5	ENSG0000	60.4	161.954	-1.408	6.58E-20
TSTD1	ENSG0000	79.181	35.715	1.127	6.37E-20
DNAJC19	ENSG0000	2.02	0.425	1.084	5.55E-20
C6	ENSG0000	47.422	120.137	-1.323	5.41E-20
C14orf105	ENSG0000	5.67	19.307	-1.606	5.36E-20
RP11-256	ENSG0000	0.83	3.32	-1.239	5.22E-20

SERPINH1	ENSG0000	37.121	15.61	1.199	5.16E-20
SLC11A1	ENSG0000	0.59	2.335	-1.069	5.08E-20
RP11-69E.	ENSG0000	0.83	3.935	-1.431	4.93E-20
LAMP2	ENSG0000	125.756	60.959	1.033	4.88E-20
UGT2B11	ENSG0000	3.8	0.615	1.572	4.59E-20
DTD1	ENSG0000	10.72	4.72	1.035	4.28E-20
FBP1	ENSG0000	116.403	296.902	-1.343	4.11E-20
THRSP	ENSG0000	6.27	72.531	-3.338	4.00E-20
RPL22L1	ENSG0000	61.469	28.814	1.067	3.95E-20
CPVL	ENSG0000	14.5	2.755	2.045	3.69E-20
FAT1	ENSG0000	33.42	11.8	1.427	3.61E-20
ID1	ENSG0000	15.63	47.12	-1.533	3.47E-20
PDXDC2P	ENSG0000	2.91	11.375	-1.662	3.44E-20
ADM2	ENSG0000	2.8	0.75	1.119	3.37E-20
SLAMF8	ENSG0000	2.05	0.415	1.108	2.94E-20
GADD45B	ENSG0000	66.472	152.783	-1.189	2.93E-20
AKR1C8P	ENSG0000	0.26	1.74	-1.121	2.75E-20
GOLGA8N	ENSG0000	1.07	3.665	-1.172	2.69E-20
RP11-756.	ENSG0000	2.13	0	1.646	2.60E-20
COLEC11	ENSG0000	18.71	53.509	-1.468	2.49E-20
LIPG	ENSG0000	8.01	21.904	-1.346	2.07E-20
SCARA3	ENSG0000	3.69	1.065	1.183	2.01E-20
CTD-2619	ENSG0000	1.41	6.11	-1.561	1.97E-20
HLA-DMA	ENSG0000	37.32	12.255	1.532	1.92E-20
SPAG4	ENSG0000	6.14	2.21	1.154	1.70E-20
SRD5A2	ENSG0000	1.17	6.605	-1.809	1.59E-20
CTB-63M2	ENSG0000	136.882	29.105	2.195	1.57E-20
HULC	ENSG0000	307.319	57.466	2.399	1.54E-20
CTSC	ENSG0000	35.961	14.13	1.289	1.43E-20
IFI27L2	ENSG0000	12.91	4.625	1.306	1.37E-20
TAP2	ENSG0000	17.289	7.24	1.15	1.36E-20
ZNF28	ENSG0000	3.54	0.65	1.46	1.30E-20
HK3	ENSG0000	1.21	4.105	-1.208	1.17E-20
AC006128	ENSG0000	1.15	7.035	-1.902	1.11E-20
PLGLB1	ENSG0000	29.8	74.586	-1.295	1.09E-20
CADM1	ENSG0000	40.07	14.41	1.414	1.01E-20
TRIM31	ENSG0000	2.55	0.295	1.455	7.90E-21
SAA4	ENSG0000	251.479	770.846	-1.612	7.75E-21
PKM	ENSG0000	38.791	13.675	1.439	7.36E-21
CAV1	ENSG0000	11.49	4.81	1.104	7.21E-21
MMP14	ENSG0000	15	5	1.415	6.88E-21
RP11-138.	ENSG0000	198.074	98.4	1.002	6.64E-21
DUSP1	ENSG0000	76.452	213.619	-1.47	6.33E-21
CROCCP2	ENSG0000	2.91	7.045	-1.041	5.78E-21
HLA-F	ENSG0000	95.709	30.939	1.598	5.53E-21
MST1L	ENSG0000	10.36	64.444	-2.526	5.43E-21
CTC-524C	ENSG0000	0.71	3.425	-1.372	4.47E-21
AQP3	ENSG0000	24.599	60.915	-1.274	4.29E-21
ASPH	ENSG0000	41.04	16.18	1.291	4.24E-21
SPG20	ENSG0000	1.55	5.41	-1.33	4.11E-21
CYP2E1	ENSG0000	500.525	2526.544	-2.333	3.98E-21
RP11-817.	ENSG0000	0.04	1.375	-1.191	3.70E-21
TTC39A	ENSG0000	2.76	0.555	1.274	3.15E-21
CTB-79E8.	ENSG0000	0	2.14	-1.651	3.13E-21
AC099668	ENSG0000	2.33	10.819	-1.828	3.09E-21
ZFP36	ENSG0000	53.742	127.304	-1.229	2.91E-21
TSPAN4	ENSG0000	40.52	18.425	1.096	2.62E-21
CYP8B1	ENSG0000	34.489	127.326	-1.854	2.61E-21

TMEM98	ENSG0000	30.38	7.44	1.895	2.53E-21
TMEM82	ENSG0000	5.08	21.11	-1.863	2.33E-21
STAP2	ENSG0000	66.68	31.891	1.041	2.07E-21
LMCD1	ENSG0000	10.45	3.68	1.291	2.04E-21
SHISA4	ENSG0000	11.06	3.83	1.32	1.95E-21
UBE2Q2	ENSG0000	5.68	2.01	1.15	1.86E-21
DBH-AS1	ENSG0000	5.1	20.855	-1.841	1.48E-21
RASSF3	ENSG0000	6.27	2.21	1.179	1.48E-21
CBR1	ENSG0000	181.056	75.123	1.258	1.46E-21
RP11-73M	ENSG0000	1.31	0	1.208	1.45E-21
TMEM25	ENSG0000	2.85	7.29	-1.107	1.37E-21
IL32	ENSG0000	748.417	295.25	1.339	1.34E-21
SPTSSA	ENSG0000	24.609	11.13	1.078	1.28E-21
RP11-35N	ENSG0000	3.71	1.305	1.031	1.27E-21
DTX1	ENSG0000	4.03	11.335	-1.294	1.17E-21
MAN1C1	ENSG0000	4.64	13.085	-1.32	1.14E-21
PYCARD	ENSG0000	16.68	4.764	1.617	1.09E-21
COCH	ENSG0000	1.42	0.18	1.036	1.03E-21
CCNL1	ENSG0000	21.84	60.633	-1.432	8.69E-22
SLC28A1	ENSG0000	8.97	30.939	-1.68	7.70E-22
NUDT14	ENSG0000	19.94	9.41	1.008	7.68E-22
FCGR2B	ENSG0000	1.64	9.1	-1.936	7.25E-22
AC138035	ENSG0000	0.6	3.465	-1.481	7.14E-22
SLAIN1	ENSG0000	0.63	2.575	-1.133	7.08E-22
TRIM16	ENSG0000	6.27	2.35	1.118	7.03E-22
CASC5	ENSG0000	3.48	1.025	1.146	7.01E-22
KMO	ENSG0000	7.17	20.115	-1.37	7.00E-22
HIST2H4A	ENSG0000	8.54	3.165	1.196	6.77E-22
EHD4	ENSG0000	9.79	3.77	1.178	6.04E-22
TGFBI	ENSG0000	114.213	51.15	1.144	5.76E-22
DBN1	ENSG0000	4.76	1.49	1.21	5.55E-22
LSMEM1	ENSG0000	0.64	3.055	-1.306	5.49E-22
DHRS7	ENSG0000	80.309	37.9	1.064	5.29E-22
RP11-667	ENSG0000	6.33	0.655	2.147	5.25E-22
FKBP11	ENSG0000	70.438	29.725	1.217	5.20E-22
NME2	ENSG0000	15.899	5.455	1.389	5.15E-22
GSTM2	ENSG0000	4.74	20.76	-1.923	4.67E-22
ARHGAP1	ENSG0000	1.86	5.19	-1.114	4.07E-22
RTP4	ENSG0000	5.18	1.725	1.181	3.68E-22
ENPP2	ENSG0000	14.99	3.555	1.812	3.56E-22
TGM3	ENSG0000	1.68	0.19	1.171	3.54E-22
AL161668.	ENSG0000	1.63	5.785	-1.367	3.53E-22
RAC3	ENSG0000	14.47	6.25	1.093	3.17E-22
RP11-465	ENSG0000	4.77	1.63	1.134	3.17E-22
AC068535	ENSG0000	1.62	9.54	-2.008	3.12E-22
ALDH8A1	ENSG0000	27.029	64.679	-1.229	3.10E-22
BCAM	ENSG0000	57.46	21.915	1.351	2.50E-22
SATB1	ENSG0000	1.65	4.965	-1.17	2.49E-22
ACADVL	ENSG0000	359.313	861.913	-1.26	2.35E-22
TSPAN8	ENSG0000	52.008	7.16	2.7	2.31E-22
STC1	ENSG0000	2.25	0.325	1.294	2.19E-22
RP11-164	ENSG0000	0.77	2.985	-1.171	1.98E-22
LAMA3	ENSG0000	3.81	0.88	1.355	1.87E-22
SPARCL1	ENSG0000	19.1	4.845	1.782	1.87E-22
TTC9	ENSG0000	2.24	0.43	1.18	1.69E-22
RP11-43N	ENSG0000	0.69	3.215	-1.318	1.67E-22
PRSS2	ENSG0000	0.03	1.25	-1.127	1.60E-22
NIPAL2	ENSG0000	5.82	2.35	1.026	1.57E-22

C1R	ENSG0000	414.692	877.009	-1.079	1.34E-22
CES4A	ENSG0000	1.93	10.081	-1.919	1.34E-22
CRTAP	ENSG0000	47.31	22.299	1.052	1.27E-22
TP53BP2	ENSG0000	12.89	4.945	1.224	1.25E-22
RP11-556	ENSG0000	1.14	0	1.098	1.21E-22
RP11-404	ENSG0000	0.35	2.57	-1.403	1.16E-22
TXNRD1	ENSG0000	30.751	11.718	1.32	1.14E-22
ACSM5	ENSG0000	28.829	79.263	-1.428	1.12E-22
XAF1	ENSG0000	7.13	29.705	-1.917	1.11E-22
TAP1	ENSG0000	22.079	9.69	1.11	1.05E-22
RP11-575	ENSG0000	0.44	2.52	-1.289	9.72E-23
NNMT	ENSG0000	269.342	1779.553	-2.719	8.31E-23
CPD	ENSG0000	16.511	6.295	1.263	8.17E-23
SLC25A27	ENSG0000	2.23	11.03	-1.897	7.15E-23
SWAP70	ENSG0000	5.09	1.985	1.029	7.00E-23
HOGA1	ENSG0000	7.79	21.02	-1.325	6.56E-23
SSR1	ENSG0000	43.609	20.554	1.049	4.69E-23
SEC31B	ENSG0000	1.22	5.16	-1.472	4.60E-23
AC239868	ENSG0000	22.92	4.91	2.017	4.33E-23
AC239868	ENSG0000	22.92	4.91	2.017	4.33E-23
PLGLB2	ENSG0000	65.348	195.78	-1.568	4.11E-23
NDRG2	ENSG0000	83.239	176.349	-1.074	4.03E-23
HIST1H2B	ENSG0000	106.042	36.82	1.501	4.03E-23
SAA1	ENSG0000	287.157	4468.423	-3.955	3.44E-23
CXCL10	ENSG0000	11.72	1.145	2.568	3.36E-23
HKDC1	ENSG0000	8.11	1.01	2.18	3.33E-23
ACADL	ENSG0000	2.9	8.555	-1.293	3.27E-23
SEZ6L2	ENSG0000	2.88	0.205	1.687	3.02E-23
CPED1	ENSG0000	3.01	7.33	-1.055	2.93E-23
TPP1	ENSG0000	67.568	32.47	1.035	2.90E-23
RP11-384	ENSG0000	1.46	4.205	-1.081	2.87E-23
HOMER3	ENSG0000	7.3	2.49	1.25	2.80E-23
AC016292	ENSG0000	2.15	0.485	1.085	2.75E-23
TNS2	ENSG0000	24.931	67.367	-1.399	2.42E-23
TPRG1	ENSG0000	1.7	4.69	-1.075	2.30E-23
LINC0051	ENSG0000	1.86	0.105	1.372	2.19E-23
GLS2	ENSG0000	7.55	64.115	-2.929	2.16E-23
MT-ND2	ENSG0000	6531.958	14680.36	-1.168	2.13E-23
F9	ENSG0000	42.659	138.457	-1.675	2.08E-23
RBM3	ENSG0000	79.539	36.115	1.118	1.99E-23
SORL1	ENSG0000	6.55	15	-1.084	1.74E-23
WDR72	ENSG0000	1.77	8.21	-1.733	1.72E-23
SMG1P7	ENSG0000	1.05	4.145	-1.328	1.69E-23
PCK1	ENSG0000	84.799	405.426	-2.244	1.54E-23
AGXT2	ENSG0000	13.63	35.03	-1.3	1.27E-23
CIDEB	ENSG0000	62.721	137.997	-1.125	1.22E-23
SNCG	ENSG0000	6.78	1.72	1.516	1.21E-23
CCL15	ENSG0000	29.421	9.305	1.562	1.09E-23
PLAU	ENSG0000	2.82	0.77	1.11	1.07E-23
CRIP1	ENSG0000	26.359	7.01	1.772	1.06E-23
MTATP6P	ENSG0000	1387.526	3715.008	-1.42	1.04E-23
HSPG2	ENSG0000	22.611	8.685	1.286	9.63E-24
ADGRA3	ENSG0000	8.9	21.525	-1.186	8.54E-24
MST1	ENSG0000	205.486	472.132	-1.196	7.93E-24
STK39	ENSG0000	2.72	0.525	1.286	7.44E-24
CTB-50L1	ENSG0000	1.12	5.205	-1.549	6.55E-24
SLC12A5	ENSG0000	2.37	0.515	1.153	6.52E-24
PAMR1	ENSG0000	0.7	2.855	-1.181	6.04E-24

UBAP1L	ENSG0000	0.45	1.93	-1.015	5.99E-24
MT-ND3	ENSG0000	9254.247	19245.72	-1.056	5.56E-24
SERPINB1	ENSG0000	26.379	10.953	1.196	4.83E-24
SLC7A2	ENSG0000	14.44	46.174	-1.611	3.88E-24
C1orf54	ENSG0000	7.23	2.655	1.171	3.55E-24
RND3	ENSG0000	9.27	21.98	-1.162	3.53E-24
MT-ND1	ENSG0000	5574.782	11534.36	-1.049	3.52E-24
PRR34-AS	ENSG0000	7.78	3.39	1	3.51E-24
LYPD1	ENSG0000	2.34	0.225	1.447	3.30E-24
CTHRC1	ENSG0000	2.35	0.245	1.428	3.20E-24
ATRN	ENSG0000	27.481	10.28	1.336	3.10E-24
HIST1H1C	ENSG0000	128.818	48.314	1.396	2.82E-24
SCAMP5	ENSG0000	3.87	0.69	1.527	2.60E-24
C4A-AS1	ENSG0000	2.72	10.738	-1.658	2.36E-24
C4B-AS1	ENSG0000	2.72	10.738	-1.658	2.36E-24
CPE	ENSG0000	15.74	4.685	1.558	2.12E-24
DBNDD2	ENSG0000	8.35	3.465	1.066	1.79E-24
SPAG5-AS	ENSG0000	0.5	2.125	-1.059	1.73E-24
ACSM3	ENSG0000	18.45	58.215	-1.606	1.69E-24
MAGI2-AS	ENSG0000	1.53	8.76	-1.948	1.65E-24
IGFBP3	ENSG0000	49.428	164.471	-1.714	1.52E-24
DUSP9	ENSG0000	2.14	0.27	1.306	1.47E-24
AVPR1A	ENSG0000	0.47	5.405	-2.123	1.46E-24
SDC2	ENSG0000	176.557	68.859	1.346	1.43E-24
MT-CO2	ENSG0000	13276.62	27014.57	-1.025	1.28E-24
TMCO3	ENSG0000	15.33	5.335	1.366	1.16E-24
LYZ	ENSG0000	81.413	9.505	2.972	1.14E-24
FANCC	ENSG0000	2.86	7.004	-1.052	1.14E-24
AC007318	ENSG0000	4.63	1.58	1.126	1.13E-24
AC093673	ENSG0000	5.26	2.085	1.021	1.12E-24
CLEC14A	ENSG0000	7.72	3.055	1.105	1.10E-24
C11orf96	ENSG0000	6.79	26.472	-1.818	1.04E-24
SFRP5	ENSG0000	0.05	4.03	-2.26	1.01E-24
GLYATL1	ENSG0000	44.609	140.035	-1.629	8.95E-25
RASGEF1B	ENSG0000	4.63	11.45	-1.145	8.14E-25
FN3K	ENSG0000	54.059	25.804	1.039	7.59E-25
MUC13	ENSG0000	5.82	0.22	2.483	6.99E-25
PRSS1	ENSG0000	0	1.17	-1.118	6.84E-25
RIPK2	ENSG0000	6.29	2.515	1.052	6.24E-25
OXT	ENSG0000	0.13	5.293	-2.477	6.22E-25
C1QTNF1	ENSG0000	4.54	17.865	-1.768	5.91E-25
DPT	ENSG0000	0.55	6.09	-2.194	5.77E-25
SAA2	ENSG0000	105.36	2489.516	-4.549	4.99E-25
RP11-347	ENSG0000	0.37	1.89	-1.077	4.93E-25
SEC11C	ENSG0000	180.33	89.39	1.004	4.71E-25
HAUS4	ENSG0000	21.44	9.115	1.15	4.55E-25
ADAMTSL	ENSG0000	3.15	11.359	-1.574	3.48E-25
CYP2B6	ENSG0000	15.26	76.219	-2.248	3.21E-25
RP11-480	ENSG0000	3.39	0.955	1.167	3.16E-25
ACACB	ENSG0000	8.47	23.61	-1.378	2.85E-25
ALOX12P2	ENSG0000	1.39	6.128	-1.577	2.52E-25
EMCN	ENSG0000	3.89	1.22	1.139	2.32E-25
CTC-246B	ENSG0000	5.76	1.865	1.238	2.29E-25
WBSCR27	ENSG0000	3.28	0.965	1.123	2.17E-25
SQLE	ENSG0000	36.309	9.635	1.811	2.09E-25
CAPN2	ENSG0000	26.33	11.385	1.142	1.95E-25
AC004540	ENSG0000	0.17	1.654	-1.182	1.94E-25
HAPLN4	ENSG0000	0.31	1.81	-1.101	1.90E-25

LGALS3	ENSG0000	30.231	6.845	1.993	1.84E-25
LOXL2	ENSG0000	3.68	1.03	1.205	1.84E-25
AP000349.	ENSG0000	1.28	0.03	1.146	1.78E-25
GYS2	ENSG0000	7.64	28.294	-1.761	1.57E-25
ICAM2	ENSG0000	12.49	5.214	1.118	1.55E-25
PLIN4	ENSG0000	4.27	22.554	-2.16	1.51E-25
RP13-516.	ENSG0000	0.38	1.96	-1.101	1.50E-25
CD248	ENSG0000	3.79	1.195	1.126	1.40E-25
RP11-228.	ENSG0000	0.38	2.354	-1.281	1.39E-25
PAPSS1	ENSG0000	6.5	2.495	1.102	1.33E-25
CD74	ENSG0000	753.727	185.354	2.018	1.25E-25
APOLD1	ENSG0000	4.3	1.215	1.259	1.19E-25
CTD-2619	ENSG0000	1.39	8.145	-1.936	1.05E-25
ETV4	ENSG0000	3.97	0.46	1.767	9.97E-26
PTMAP5	ENSG0000	2.79	0.765	1.103	9.23E-26
ITGB4	ENSG0000	5.13	1.67	1.199	9.14E-26
SH3BGRL3	ENSG0000	70.629	32.565	1.094	7.00E-26
CUEDC1	ENSG0000	7.88	3.19	1.084	6.86E-26
TBC1D16	ENSG0000	5.07	1.81	1.111	6.32E-26
RRP15	ENSG0000	4.32	1.655	1.003	5.88E-26
SAMD5	ENSG0000	0.48	2.47	-1.229	5.79E-26
TENM1	ENSG0000	0.12	1.3	-1.038	5.58E-26
MPPED1	ENSG0000	1.34	5.769	-1.532	5.30E-26
RNF157	ENSG0000	3.3	0.58	1.444	5.21E-26
PEMT	ENSG0000	50.53	119.908	-1.23	5.18E-26
LINC0155	ENSG0000	1.42	69.249	-4.859	4.99E-26
NT5DC2	ENSG0000	11.39	3.31	1.523	4.62E-26
SLC44A3	ENSG0000	6.79	1.63	1.567	4.44E-26
CSTB	ENSG0000	62.012	30.3	1.009	3.64E-26
ABCC9	ENSG0000	2.25	6.825	-1.268	3.07E-26
CCDC28B	ENSG0000	3.8	1.31	1.055	2.88E-26
RPL23AP6	ENSG0000	2.83	0.525	1.329	2.76E-26
DIO3OS	ENSG0000	0.41	4.344	-1.922	2.75E-26
CAPN3	ENSG0000	3.25	13.15	-1.735	2.72E-26
UCHL5	ENSG0000	15.85	7.27	1.027	2.61E-26
RP11-632.	ENSG0000	1.68	5.815	-1.346	2.31E-26
RP11-125.	ENSG0000	0.32	2.325	-1.333	1.81E-26
ALPL	ENSG0000	10.67	40.58	-1.833	1.71E-26
HPN-AS1	ENSG0000	0.59	3.69	-1.561	1.59E-26
IGSF8	ENSG0000	46.529	21.859	1.056	1.58E-26
HTATIP2	ENSG0000	77.831	32.464	1.236	1.48E-26
CASK	ENSG0000	8.9	3.435	1.159	1.41E-26
CAMK2B	ENSG0000	0.24	2.31	-1.416	1.36E-26
GCH1	ENSG0000	11.76	29.19	-1.242	1.20E-26
GSTZ1	ENSG0000	29.67	81.055	-1.42	1.10E-26
LL22NC03	ENSG0000	1.96	0.29	1.198	1.07E-26
ALDOA	ENSG0000	289.595	130.929	1.139	1.02E-26
DCK	ENSG0000	3.95	1.415	1.035	8.38E-27
TBCAP1	ENSG0000	1.64	0.26	1.067	8.15E-27
C1orf198	ENSG0000	12.46	4.765	1.223	7.64E-27
SLC51B	ENSG0000	3.87	0.415	1.783	6.74E-27
SEMA3G	ENSG0000	1.93	0.435	1.03	6.60E-27
MT-ND4L	ENSG0000	5341.391	15483.03	-1.535	6.04E-27
LILRB5	ENSG0000	1.45	4.175	-1.079	5.91E-27
CYP2C8	ENSG0000	85.017	509.168	-2.568	5.79E-27
DNASE2	ENSG0000	32.07	13.445	1.195	5.48E-27
SHC1	ENSG0000	58.189	27.29	1.065	5.31E-27
FKBP9	ENSG0000	17.64	8.315	1.001	5.14E-27

DTNA	ENSG0000	6.14	0.99	1.843	4.13E-27
uc_338	ENSG0000	0	1.365	-1.242	4.08E-27
C1orf228	ENSG0000	1.79	6.188	-1.365	4.05E-27
MTND4P2	ENSG0000	4	25.358	-2.398	3.83E-27
CSF3R	ENSG0000	1.43	6.905	-1.702	3.81E-27
FLJ22763	ENSG0000	0.76	6.25	-2.042	3.65E-27
PDIA6	ENSG0000	159.155	77.753	1.024	3.64E-27
ST8SIA6-A	ENSG0000	3.81	0	2.266	2.87E-27
NPM3	ENSG0000	17.14	5.595	1.46	2.79E-27
CTD-3092	ENSG0000	2.47	9.255	-1.563	2.53E-27
CRNDE	ENSG0000	4.62	0.5	1.906	2.52E-27
LRRC1	ENSG0000	2.35	0.34	1.322	2.45E-27
LMNB1	ENSG0000	7.73	2.21	1.443	2.22E-27
NR1I2	ENSG0000	9.26	30.295	-1.609	2.22E-27
GAS5	ENSG0000	169.388	80.573	1.063	1.93E-27
FBLN7	ENSG0000	6.2	2.045	1.242	1.89E-27
DUXAP8	ENSG0000	1.89	0.225	1.238	1.59E-27
MTHFD1L	ENSG0000	4.12	1.275	1.17	1.52E-27
PIR	ENSG0000	23.02	9.2	1.236	1.28E-27
ARPC1B	ENSG0000	99.181	38.33	1.349	1.13E-27
PHYKPL	ENSG0000	20.569	47.765	-1.177	1.10E-27
RPS3AP6	ENSG0000	12.82	5.885	1.005	1.09E-27
H2BFS	ENSG0000	7.35	2.365	1.311	1.07E-27
THEM6	ENSG0000	35.921	16.975	1.038	1.03E-27
RP11-443	ENSG0000	1.78	0.02	1.447	1.02E-27
TOR3A	ENSG0000	17.179	7.93	1.026	1.02E-27
PDGFA	ENSG0000	9.08	1.965	1.765	1.02E-27
MFAP3L	ENSG0000	1.98	7.005	-1.426	9.41E-28
AZGP1P1	ENSG0000	6.43	19.275	-1.448	8.77E-28
GGTA1P	ENSG0000	3.88	1.105	1.213	7.57E-28
KCND3	ENSG0000	0.84	3.235	-1.203	7.35E-28
SOX12	ENSG0000	4.26	1.51	1.067	6.01E-28
ACLY	ENSG0000	20.91	8.174	1.256	5.83E-28
RP11-390	ENSG0000	1.26	4.439	-1.267	5.45E-28
RP11-579	ENSG0000	1.95	0.33	1.149	5.42E-28
MAPRE1	ENSG0000	14.98	6.11	1.168	5.40E-28
MARCKS	ENSG0000	15.05	5.1	1.396	5.08E-28
TMEM164	ENSG0000	2.59	0.78	1.012	5.00E-28
H1F0	ENSG0000	142.438	60.62	1.219	4.46E-28
PLAC8	ENSG0000	1.03	5.975	-1.781	4.43E-28
NFKBIE	ENSG0000	11.85	4.04	1.35	4.40E-28
DEPTOR	ENSG0000	9.3	3.985	1.047	4.12E-28
CYP39A1	ENSG0000	3.25	17.415	-2.115	3.87E-28
NCAPG2	ENSG0000	2.93	0.895	1.052	3.71E-28
FAM103A	ENSG0000	6.95	2.265	1.284	2.92E-28
MT1XP1	ENSG0000	0	2.03	-1.599	2.90E-28
GSTA4	ENSG0000	18.01	6.855	1.275	2.86E-28
FOLH1B	ENSG0000	0.43	4.13	-1.843	2.53E-28
MT-CYB	ENSG0000	6751.535	15334.04	-1.183	2.38E-28
RP11-475	ENSG0000	15.42	7.145	1.011	2.34E-28
GTF2IP4	ENSG0000	23.86	8.225	1.43	1.57E-28
TMEM14A	ENSG0000	50.499	22.89	1.108	1.53E-28
FAM49B	ENSG0000	17.579	7.295	1.163	1.50E-28
RP11-622	ENSG0000	5.56	23.667	-1.911	1.49E-28
BMS1P8	ENSG0000	1.92	0.11	1.395	1.45E-28
RRAGD	ENSG0000	8.76	3.255	1.198	1.36E-28
G6PD	ENSG0000	9.9	2.875	1.492	1.33E-28
SLC6A13	ENSG0000	2.29	11.89	-1.97	1.19E-28

AKR7L	ENSG0000	4.43	14.072	-1.473	1.15E-28
SRXN1	ENSG0000	30.141	10.395	1.45	1.06E-28
CDHR2	ENSG0000	1.82	16.165	-2.606	1.02E-28
CHRNA4	ENSG0000	0.33	12.095	-3.3	9.74E-29
SPARC	ENSG0000	214.881	65.26	1.704	8.74E-29
CDC6	ENSG0000	2.44	0.365	1.334	8.64E-29
BTG3	ENSG0000	8.55	3.095	1.222	8.12E-29
LEF1	ENSG0000	2.67	0.29	1.508	8.05E-29
PLIN1	ENSG0000	1.84	8.99	-1.815	7.43E-29
ANKRD29	ENSG0000	3.48	0.61	1.476	6.70E-29
RP11-676.	ENSG0000	0.11	1.625	-1.242	6.65E-29
LY6E	ENSG0000	54.53	259.294	-2.229	6.44E-29
PSMB9	ENSG0000	50.011	17.194	1.487	6.35E-29
CANX	ENSG0000	179.83	85.843	1.058	6.23E-29
CD151	ENSG0000	152.704	70.965	1.095	6.23E-29
SOX18	ENSG0000	4.61	1.78	1.013	5.70E-29
ADCY1	ENSG0000	0.4	4.115	-1.869	5.43E-29
CSAD	ENSG0000	14.78	44.387	-1.524	5.27E-29
RP3-417G	ENSG0000	2.94	0.94	1.022	5.27E-29
RP11-286	ENSG0000	0.06	1.165	-1.03	5.25E-29
SPRR3	ENSG0000	0	1.315	-1.211	5.13E-29
RNASE1	ENSG0000	56.531	18.8	1.539	4.55E-29
ENO3	ENSG0000	11.36	58.1	-2.258	4.36E-29
PSMD2	ENSG0000	81.48	38.55	1.06	4.01E-29
FBXO32	ENSG0000	4.23	1.22	1.236	3.93E-29
PROZ	ENSG0000	8.74	32.433	-1.779	3.48E-29
P3H4	ENSG0000	3.91	1.29	1.1	3.48E-29
ABCC6P2	ENSG0000	8.95	3.735	1.071	3.34E-29
RP11-284	ENSG0000	1.99	0.06	1.496	3.11E-29
ABLIM3	ENSG0000	12.69	37.135	-1.478	3.05E-29
SLC22A1	ENSG0000	56.102	495.588	-3.12	2.77E-29
ENAH	ENSG0000	10.65	2.805	1.614	2.54E-29
APOBEC3	ENSG0000	2.34	0.22	1.453	2.35E-29
PLXND1	ENSG0000	36.579	15.975	1.147	2.23E-29
LRCOL1	ENSG0000	1.04	7.855	-2.118	2.13E-29
NQO1	ENSG0000	11.72	1.285	2.477	2.02E-29
MT-ATP8	ENSG0000	11020.48	37358.53	-1.761	1.62E-29
NEDD4L	ENSG0000	25.941	11.405	1.119	1.58E-29
CTD-308C	ENSG0000	0.44	3.79	-1.734	1.40E-29
FOXRED2	ENSG0000	6.45	2.045	1.291	1.30E-29
FUCA2	ENSG0000	34.951	16.73	1.02	1.28E-29
HELLS	ENSG0000	1.83	0.405	1.01	1.27E-29
DCN	ENSG0000	14.87	101.289	-2.688	1.21E-29
ZP3	ENSG0000	2.71	0.66	1.16	1.18E-29
RAD21	ENSG0000	33.499	14.025	1.199	1.17E-29
PSMB10	ENSG0000	59.112	28.645	1.02	1.14E-29
ATP1B1	ENSG0000	97.951	37.295	1.37	1.12E-29
GHR	ENSG0000	7.3	26.178	-1.711	1.09E-29
TPPP2	ENSG0000	0.76	3.525	-1.362	1.01E-29
MANEAL	ENSG0000	9.16	3.035	1.332	1.01E-29
AKR1B10	ENSG0000	177.158	3.015	5.472	9.84E-30
TUBA1B	ENSG0000	234.996	72.433	1.684	8.76E-30
CYR61	ENSG0000	14.11	53.815	-1.859	8.70E-30
AC012146	ENSG0000	5.99	2.17	1.141	8.25E-30
EZH2	ENSG0000	3.65	1.275	1.031	7.56E-30
RNF152	ENSG0000	2.87	7.37	-1.113	7.43E-30
COPA	ENSG0000	47.009	19.744	1.211	6.92E-30
ATF5	ENSG0000	145.946	767.753	-2.387	6.51E-30

CCNE1	ENSG0000	1.55	0.22	1.064	5.34E-30
FOS	ENSG0000	15.56	80.208	-2.294	5.31E-30
ATP6V1C1	ENSG0000	16.8	6.985	1.157	5.23E-30
MST1P2	ENSG0000	9.95	74.23	-2.78	5.13E-30
NOTCH3	ENSG0000	4.32	0.925	1.467	5.02E-30
ACSL4	ENSG0000	33.02	2.355	3.342	4.63E-30
RP11-161	ENSG0000	58.602	23.37	1.29	4.29E-30
PLA2G4C	ENSG0000	16.41	5.25	1.478	3.54E-30
DLGAP1-	ENSG0000	7.94	2.765	1.248	3.17E-30
BX842568.	ENSG0000	0.4	2.485	-1.316	3.16E-30
C19orf66	ENSG0000	42.679	100.513	-1.217	3.13E-30
ITPKA	ENSG0000	3.46	0.555	1.52	3.00E-30
HACD3	ENSG0000	47.261	19.23	1.254	2.71E-30
RP11-434	ENSG0000	1.32	11.964	-2.482	2.64E-30
RP4-706A	ENSG0000	22.42	9.355	1.177	2.60E-30
TPGS2	ENSG0000	20.93	8.2	1.253	2.54E-30
ATOH8	ENSG0000	4.42	18.95	-1.88	2.47E-30
CENPK	ENSG0000	1.51	0.24	1.017	2.37E-30
CDKN2B	ENSG0000	2.03	0.37	1.145	2.24E-30
EPS8L3	ENSG0000	4.76	0.13	2.35	2.06E-30
ZC3H13	ENSG0000	4.81	15.4	-1.497	1.80E-30
NAP1L1	ENSG0000	87.462	40.215	1.102	1.73E-30
ARF3	ENSG0000	30.759	14.79	1.008	1.65E-30
CD163L1	ENSG0000	2.63	0.49	1.285	1.64E-30
IFI30	ENSG0000	140.769	51.639	1.429	1.31E-30
MPZL1	ENSG0000	21.41	8.445	1.247	1.17E-30
CHRD	ENSG0000	9.55	72.098	-2.793	1.16E-30
EEF1A1P6	ENSG0000	11.7	3.45	1.513	1.01E-30
AC009065	ENSG0000	1.34	0	1.227	9.02E-31
ADRA1B	ENSG0000	1.21	3.855	-1.135	8.39E-31
PLSCR4	ENSG0000	5.39	13.155	-1.147	8.18E-31
KIF23	ENSG0000	1.48	0.19	1.059	7.83E-31
TPR	ENSG0000	20	9.455	1.006	7.29E-31
C7	ENSG0000	5.88	49.485	-2.875	7.22E-31
LAPTM4B	ENSG0000	34.179	7.56	2.039	7.12E-31
SERPINI1	ENSG0000	3.56	0.94	1.233	6.41E-31
SLCO1B3	ENSG0000	1.89	30.546	-3.448	4.57E-31
ACSL1	ENSG0000	129.984	406.58	-1.638	4.55E-31
RGS10	ENSG0000	7.6	1.915	1.561	4.08E-31
ATP5J2-P	ENSG0000	3.96	1.075	1.257	3.69E-31
CSRNP1	ENSG0000	7.19	23.779	-1.597	3.50E-31
DDAH2	ENSG0000	45.041	19.78	1.148	3.31E-31
RP11-415	ENSG0000	0.74	3.8	-1.464	3.31E-31
RELB	ENSG0000	13.58	4.209	1.485	2.92E-31
PPIC	ENSG0000	23.64	11.08	1.028	2.60E-31
ZIC2	ENSG0000	1.42	0.01	1.261	2.17E-31
DEFA3	ENSG0000	0	1.345	-1.23	2.14E-31
BCAS4	ENSG0000	1.85	0.41	1.015	2.00E-31
ACTN4	ENSG0000	102.749	47.2	1.106	1.82E-31
RAP2A	ENSG0000	5.38	1.86	1.158	1.82E-31
ATP1A1	ENSG0000	136.342	51.872	1.377	1.61E-31
SFN	ENSG0000	9.74	0.605	2.742	1.61E-31
LRP11	ENSG0000	7.82	3.315	1.032	1.44E-31
BPNT1	ENSG0000	13.67	6.01	1.065	1.24E-31
RAD51AP	ENSG0000	1.72	0.29	1.076	1.24E-31
PSEN2	ENSG0000	14.1	6.11	1.087	1.08E-31
PPT1	ENSG0000	19.291	7.325	1.285	1.05E-31
CTSD	ENSG0000	769.138	382.084	1.007	1.02E-31

GJA5	ENSG0000	2.24	0.385	1.226	1.02E-31
NUDCD2	ENSG0000	15.3	6.895	1.046	8.99E-32
IMMP2L	ENSG0000	17.52	8.165	1.015	8.77E-32
NMB	ENSG0000	3.43	0.835	1.272	8.27E-32
PODXL	ENSG0000	5.7	1.355	1.508	8.20E-32
PAQR4	ENSG0000	3.82	0.8	1.421	7.08E-32
CD1D	ENSG0000	1.31	5.54	-1.501	6.84E-32
C1orf168	ENSG0000	3.31	11.729	-1.562	6.25E-32
ACP6	ENSG0000	18.6	8.69	1.016	6.14E-32
PTENP1	ENSG0000	1.98	0.39	1.1	5.57E-32
RFC3	ENSG0000	3.4	1.165	1.023	5.19E-32
C15orf39	ENSG0000	4.46	1.5	1.127	5.00E-32
SLC25A6	ENSG0000	110.063	52.654	1.05	5.00E-32
RDH16	ENSG0000	39.609	193.762	-2.262	4.68E-32
IFI35	ENSG0000	35.901	15.2	1.188	3.78E-32
RP11-713	ENSG0000	10.17	3.25	1.394	3.38E-32
TUBB	ENSG0000	207.015	88.763	1.212	3.10E-32
SPA17	ENSG0000	2.84	0.75	1.134	2.96E-32
PLOD1	ENSG0000	76.388	36.346	1.051	2.95E-32
F11-AS1	ENSG0000	2.75	8.87	-1.396	2.92E-32
RPS6	ENSG0000	1117.066	553.925	1.011	2.75E-32
RP11-394	ENSG0000	0.23	1.495	-1.02	2.61E-32
SUCO	ENSG0000	6.03	2.25	1.113	2.38E-32
FLVCR1-A	ENSG0000	2.93	0.71	1.201	1.73E-32
MT1E	ENSG0000	86.228	797.532	-3.194	1.59E-32
DTNBP1	ENSG0000	8.48	3.67	1.021	1.58E-32
PTP4A3	ENSG0000	8.63	2.31	1.541	1.56E-32
FABP5P7	ENSG0000	8.22	1.645	1.801	1.41E-32
AIMP2	ENSG0000	27.651	13.26	1.007	1.33E-32
ECSCR	ENSG0000	6.8	2.27	1.254	1.22E-32
ROBO1	ENSG0000	9.19	1.07	2.299	1.09E-32
RAMP2	ENSG0000	17.27	5.77	1.432	1.02E-32
RP4-583P	ENSG0000	2.09	7.12	-1.394	1.02E-32
UROC1	ENSG0000	3.89	38.638	-3.019	9.88E-33
CAPRIN1	ENSG0000	35.17	17.019	1.005	9.85E-33
RNASET2	ENSG0000	42.86	17.46	1.248	9.02E-33
TREM2	ENSG0000	3.51	0.245	1.857	9.01E-33
NES	ENSG0000	5.32	1.63	1.265	6.86E-33
UBALD2	ENSG0000	27.02	12.254	1.08	6.24E-33
FAM111B	ENSG0000	1.57	0.09	1.237	5.95E-33
TMEM27	ENSG0000	1.03	7.24	-2.021	5.51E-33
ITIH4-AS1	ENSG0000	0.58	3.97	-1.653	4.22E-33
AL158801.	ENSG0000	2.19	0.275	1.323	4.13E-33
HAO2	ENSG0000	11.13	111.712	-3.216	4.03E-33
DUSP23	ENSG0000	131.497	63.76	1.033	3.93E-33
LHX2	ENSG0000	0.36	2.33	-1.292	3.44E-33
IDO2	ENSG0000	0.11	2.54	-1.673	3.37E-33
TRIP13	ENSG0000	1.55	0.21	1.075	3.15E-33
TKT	ENSG0000	113.921	35.77	1.644	3.14E-33
ETFDH	ENSG0000	27.81	58.846	-1.055	2.86E-33
STIP1	ENSG0000	52.57	22.525	1.187	2.85E-33
RUSC1	ENSG0000	10.76	4.85	1.007	2.44E-33
CTNNBL1	ENSG0000	45.379	20.99	1.077	2.39E-33
CKAP2	ENSG0000	3.09	0.855	1.141	2.09E-33
PIK3R2	ENSG0000	16.35	6.97	1.122	1.80E-33
SMYD2	ENSG0000	30.199	12.82	1.175	1.71E-33
NBPF8	ENSG0000	2.65	12.719	-1.91	1.69E-33
MT1L	ENSG0000	5.34	45.263	-2.867	1.56E-33

KAZN	ENSG0000	0.34	1.91	-1.119	1.53E-33
AIM1L	ENSG0000	1.81	0.17	1.264	1.32E-33
GTF2IRD1	ENSG0000	7.07	2.745	1.108	1.25E-33
RRN3P1	ENSG0000	1.24	5.655	-1.571	1.25E-33
ABCA9	ENSG0000	0.63	3.27	-1.389	1.21E-33
HNRNPA1	ENSG0000	7.86	3.015	1.142	1.06E-33
FAM13A	ENSG0000	3.1	15.24	-1.986	1.03E-33
COPG2	ENSG0000	6	2.18	1.138	8.90E-34
SLC25A37	ENSG0000	4.4	12.924	-1.367	8.04E-34
ZNF585A	ENSG0000	5.72	2.155	1.091	7.45E-34
CHCHD3	ENSG0000	24.45	10.515	1.144	7.26E-34
DCTN2	ENSG0000	55.341	26.62	1.028	6.03E-34
N4BP2L1	ENSG0000	12.22	28.05	-1.136	5.56E-34
FAM198A	ENSG0000	0.36	3.95	-1.864	4.73E-34
ECT2	ENSG0000	3	0.61	1.313	4.23E-34
LINC0160	ENSG0000	8.22	3.345	1.085	3.98E-34
BZW2	ENSG0000	15.3	6.63	1.095	3.77E-34
RP3-461F	ENSG0000	7.65	1.78	1.638	3.70E-34
DES	ENSG0000	0.19	2.725	-1.646	3.52E-34
APLN	ENSG0000	1.59	0.1	1.235	3.06E-34
LINC0129	ENSG0000	2.28	0.15	1.512	2.92E-34
IL1RL1	ENSG0000	0.08	1.33	-1.109	2.80E-34
DBP	ENSG0000	18.81	6.13	1.474	2.74E-34
GPATCH4	ENSG0000	18.181	7.33	1.203	2.74E-34
SORT1	ENSG0000	7.08	1.66	1.603	2.69E-34
TMEM209	ENSG0000	5.27	2.015	1.056	2.42E-34
LINC0023	ENSG0000	0.19	3.295	-1.852	2.37E-34
HMGN2P5	ENSG0000	42.659	13.404	1.6	2.32E-34
OR2I1P	ENSG0000	25.71	1.305	3.535	2.25E-34
RGS5	ENSG0000	18.01	4.01	1.924	2.20E-34
NCSTN	ENSG0000	62.552	26.736	1.196	1.96E-34
HLA-H	ENSG0000	36.931	6.175	2.402	1.66E-34
BUB1B	ENSG0000	1.6	0.16	1.164	1.37E-34
CSTF2	ENSG0000	4.47	1.56	1.095	1.37E-34
CALM3	ENSG0000	104.213	50.399	1.033	1.35E-34
ATIC	ENSG0000	66.201	32.119	1.021	1.34E-34
RRS1	ENSG0000	16	4.47	1.636	1.32E-34
RP1-232P	ENSG0000	0.06	1.595	-1.292	1.26E-34
SOWAHA	ENSG0000	2.79	0.565	1.276	1.14E-34
PARP1	ENSG0000	36.99	15.685	1.187	1.12E-34
AC104809	ENSG0000	0.04	3.375	-2.073	1.11E-34
MT-ND4	ENSG0000	9684.084	23104.72	-1.254	1.08E-34
NFKBIZ	ENSG0000	3.85	18.334	-1.995	1.07E-34
NSL1	ENSG0000	13.84	6.095	1.065	1.05E-34
CCL14	ENSG0000	7.25	46.812	-2.535	1.04E-34
ZNF775	ENSG0000	8.49	3.35	1.125	1.01E-34
YBEY	ENSG0000	17.97	8.19	1.046	9.18E-35
FANCD2	ENSG0000	2.68	0.595	1.206	8.55E-35
SPATS2	ENSG0000	5.64	2.025	1.134	7.78E-35
HGF	ENSG0000	0.79	6.615	-2.089	6.68E-35
DLL4	ENSG0000	3.79	1.165	1.146	6.49E-35
UHRF1	ENSG0000	1.79	0.115	1.323	6.25E-35
TRAPPC2E	ENSG0000	10.94	3.775	1.322	5.49E-35
HGFAC	ENSG0000	11.26	117.783	-3.276	5.22E-35
NPM1P27	ENSG0000	5.95	1.935	1.244	5.04E-35
YIF1B	ENSG0000	47.891	22.32	1.068	4.91E-35
ABCA8	ENSG0000	3.33	13.07	-1.7	4.52E-35
RP5-966N	ENSG0000	6.86	66.16	-3.095	4.32E-35

C6orf47	ENSG0000	10.63	4.24	1.15	4.29E-35
AC016739	ENSG0000	12.83	4.745	1.267	4.16E-35
C9	ENSG0000	23.051	263.854	-3.461	3.80E-35
PHF19	ENSG0000	6.2	1.995	1.265	3.53E-35
ESR1	ENSG0000	0.53	4.47	-1.838	3.35E-35
PRR11	ENSG0000	1.37	0.14	1.056	2.78E-35
ZNF544	ENSG0000	9.53	3.565	1.206	2.66E-35
TP53I3	ENSG0000	20.09	5.15	1.778	2.13E-35
PSPH	ENSG0000	8.69	2.705	1.387	2.07E-35
CBX1	ENSG0000	14.33	5.675	1.2	2.02E-35
TAF9	ENSG0000	33.171	15.97	1.01	1.93E-35
P4HA2	ENSG0000	15.899	5.81	1.311	1.88E-35
SRPX	ENSG0000	0.84	5.53	-1.827	1.85E-35
KRT17P4	ENSG0000	0.05	1.34	-1.156	1.80E-35
GNAZ	ENSG0000	2.38	0.15	1.555	1.68E-35
TDRKH	ENSG0000	4.09	1.165	1.233	1.56E-35
DDOST	ENSG0000	104.22	48.816	1.079	1.42E-35
TMEM38B	ENSG0000	8.39	3.005	1.229	1.28E-35
RIC3	ENSG0000	0.07	1.775	-1.375	1.08E-35
RPL12	ENSG0000	922.752	451.379	1.03	9.95E-36
FAM222A	ENSG0000	4.25	1.125	1.305	9.68E-36
RPL6P27	ENSG0000	12.27	4.93	1.162	9.28E-36
DBNDD1	ENSG0000	11.41	2.12	1.992	7.68E-36
CAPG	ENSG0000	26.6	3.725	2.546	7.53E-36
RPL24P4	ENSG0000	9.02	3.245	1.239	7.20E-36
KRT13	ENSG0000	0	2.74	-1.903	6.91E-36
INMT	ENSG0000	1.57	7.73	-1.764	6.84E-36
PLK1	ENSG0000	3.77	0.765	1.434	6.79E-36
GLA	ENSG0000	19.061	7.165	1.297	6.62E-36
PYGB	ENSG0000	14.47	5.205	1.318	6.43E-36
TARBP1	ENSG0000	14.1	5.62	1.19	5.14E-36
RFX5	ENSG0000	10.55	3.49	1.363	4.80E-36
KIAA1522	ENSG0000	11.99	3.85	1.421	4.79E-36
PDGFB	ENSG0000	4.05	1.06	1.294	4.44E-36
STEAP4	ENSG0000	0.77	4.24	-1.566	4.38E-36
MCAM	ENSG0000	14.76	3.97	1.665	3.84E-36
TMEM150	ENSG0000	6.44	0.645	2.177	3.44E-36
GBP2	ENSG0000	45.972	15.32	1.525	3.30E-36
HMGN1	ENSG0000	69.42	32.281	1.081	3.30E-36
DNASE1L3	ENSG0000	6.64	39.306	-2.399	3.21E-36
CFP	ENSG0000	2.32	8.934	-1.581	3.12E-36
RAB11FIP4	ENSG0000	5.22	1.225	1.483	3.08E-36
SAMM50	ENSG0000	30.89	14.73	1.02	2.94E-36
LAMA4	ENSG0000	4.83	1.38	1.293	2.58E-36
OPTN	ENSG0000	50.569	22.641	1.125	2.32E-36
SNHG6	ENSG0000	110.921	47.349	1.211	2.29E-36
HRCT1	ENSG0000	4.34	0.48	1.851	2.27E-36
RPP40	ENSG0000	7.11	3.025	1.011	2.19E-36
AC092171	ENSG0000	2.23	0.56	1.05	2.10E-36
NGFR	ENSG0000	1.25	7.695	-1.95	2.04E-36
CCNA2	ENSG0000	2.96	0.455	1.445	1.85E-36
ARHGEF39	ENSG0000	1.81	0.4	1.005	1.83E-36
PDAP1	ENSG0000	72.978	35.554	1.017	1.75E-36
FAM83D	ENSG0000	4.5	0.855	1.568	1.65E-36
C8orf4	ENSG0000	12.81	58.63	-2.11	1.62E-36
RP11-290	ENSG0000	3.19	20.87	-2.384	1.44E-36
RGCC	ENSG0000	11.91	2.12	2.049	1.35E-36
APIP	ENSG0000	18.439	8.705	1.002	1.29E-36

YWHAZ	ENSG0000	107.38	40.699	1.378	1.29E-36
TYMSOS	ENSG0000	2.58	0.44	1.314	1.16E-36
TWF2	ENSG0000	30.83	12.465	1.241	1.10E-36
RP11-295	ENSG0000	20.34	5.555	1.703	9.96E-37
EIF5AP4	ENSG0000	3.95	0.16	2.093	9.78E-37
HTATSF1	ENSG0000	19.28	8.46	1.1	9.73E-37
AC016747	ENSG0000	4.32	1.61	1.027	9.53E-37
RPL37A	ENSG0000	2021.064	985.047	1.036	9.48E-37
RP11-258	ENSG0000	0.23	1.655	-1.11	9.13E-37
MTHFD2L	ENSG0000	3.57	9.69	-1.226	7.44E-37
AC005255	ENSG0000	3.45	0	2.154	6.77E-37
DPP3	ENSG0000	21.58	10.23	1.008	6.32E-37
SLC39A14	ENSG0000	54.681	160.457	-1.536	6.14E-37
PITPNA-A	ENSG0000	8.52	3.245	1.165	5.67E-37
DERL1	ENSG0000	56.191	26.628	1.05	5.19E-37
MKKS	ENSG0000	22.899	10.375	1.071	4.69E-37
RP11-111	ENSG0000	4.52	1.645	1.061	4.45E-37
HAUS1	ENSG0000	8.63	3.675	1.043	4.37E-37
RP11-128	ENSG0000	1.61	0.24	1.074	4.26E-37
ALKBH2	ENSG0000	25.259	11.915	1.024	4.09E-37
CYP1A2	ENSG0000	0.48	44.13	-4.93	3.50E-37
B9D1	ENSG0000	10.26	3.88	1.206	3.33E-37
DPCD	ENSG0000	7.67	3.15	1.063	3.19E-37
SMS	ENSG0000	25.701	11.905	1.049	2.98E-37
MYOM2	ENSG0000	0.86	4.635	-1.599	2.62E-37
CTC-529I	ENSG0000	0.66	3.14	-1.318	2.44E-37
GINS1	ENSG0000	1.72	0.2	1.181	2.40E-37
IFT52	ENSG0000	7.39	3.06	1.047	2.12E-37
NPY1R	ENSG0000	0.4	2.24	-1.211	1.79E-37
KIFAP3	ENSG0000	9.63	3.71	1.174	1.70E-37
STYXL1	ENSG0000	31.679	14.21	1.103	1.62E-37
ACADS	ENSG0000	39.17	97.48	-1.294	1.55E-37
TMSB10	ENSG0000	859.646	208.556	2.038	1.53E-37
MCM4	ENSG0000	11.24	2.725	1.716	1.41E-37
GCKR	ENSG0000	22.119	98.499	-2.106	1.31E-37
RPL13AP5	ENSG0000	68.802	31.064	1.122	1.31E-37
COL4A2	ENSG0000	30.359	7.015	1.968	1.19E-37
RUVBL1	ENSG0000	17.349	7.765	1.066	1.18E-37
ANXA2P2	ENSG0000	4.42	0.755	1.627	1.14E-37
PSMD14	ENSG0000	27.46	11.975	1.133	1.07E-37
EIF3CL	ENSG0000	33.16	14.54	1.136	9.35E-38
SEC61G	ENSG0000	167.195	79.423	1.064	8.75E-38
C19orf48	ENSG0000	26.67	11.515	1.145	7.86E-38
OR10J6P	ENSG0000	0.62	5.62	-2.031	6.99E-38
GOLPH3L	ENSG0000	9.31	3.605	1.163	6.65E-38
RPL15P3	ENSG0000	8.69	3.015	1.271	6.17E-38
HMGN1P3	ENSG0000	1.59	0	1.373	5.96E-38
SNRPGP2	ENSG0000	9.32	2.87	1.415	5.66E-38
RPL23A	ENSG0000	690.213	342.33	1.01	5.01E-38
CENPN	ENSG0000	6.1	2.08	1.205	4.90E-38
LASP1	ENSG0000	64.408	28.585	1.145	4.22E-38
HBA2	ENSG0000	22.15	162.612	-2.821	4.20E-38
SSR3	ENSG0000	89.059	40.774	1.108	4.02E-38
GNS	ENSG0000	30.759	13.59	1.122	3.96E-38
DLGAP5	ENSG0000	1.41	0.07	1.171	3.76E-38
PTK2	ENSG0000	22.721	9.61	1.161	3.37E-38
SPATC1L	ENSG0000	3.81	0.41	1.77	3.21E-38
FDPS	ENSG0000	291.63	108.698	1.416	2.85E-38

MCC	ENSG0000	1.22	3.825	-1.12	2.64E-38
C9orf16	ENSG0000	50.801	23.865	1.059	2.63E-38
APOF	ENSG0000	17.701	113.668	-2.616	2.62E-38
SYNGR2	ENSG0000	83.169	40.321	1.026	2.46E-38
HGH1	ENSG0000	22.409	10.215	1.062	1.99E-38
CCDC86	ENSG0000	12.32	4.795	1.201	1.91E-38
NRM	ENSG0000	8.47	2.63	1.383	1.91E-38
CHEK1	ENSG0000	2.38	0.48	1.191	1.77E-38
KNSTRN	ENSG0000	7.78	2.795	1.21	1.60E-38
MIS18A	ENSG0000	4.36	1.595	1.047	1.36E-38
RPS24	ENSG0000	1646.514	799.358	1.042	1.33E-38
DIRAS3	ENSG0000	0.25	2.305	-1.403	1.29E-38
C6orf89	ENSG0000	20.281	9.085	1.077	1.24E-38
ATAD2	ENSG0000	6.91	1.855	1.47	1.16E-38
CKAP4	ENSG0000	32.45	11.11	1.466	9.61E-39
MMP11	ENSG0000	5.24	0.35	2.209	9.60E-39
ARL2	ENSG0000	24.729	8.94	1.372	6.90E-39
CACYBP	ENSG0000	43.419	15.51	1.428	6.64E-39
RAD51	ENSG0000	1.75	0.235	1.155	6.48E-39
PSMD10	ENSG0000	20.941	8.67	1.182	5.29E-39
FHIT	ENSG0000	11.09	4.22	1.212	4.78E-39
PARPBP	ENSG0000	1.5	0.19	1.071	4.70E-39
TECRP1	ENSG0000	6.04	1.785	1.338	4.63E-39
LPAL2	ENSG0000	1.31	6.39	-1.678	4.47E-39
GLB1	ENSG0000	33.079	15.252	1.068	3.66E-39
ATP6V1D	ENSG0000	38.409	18.475	1.017	3.53E-39
VBP1	ENSG0000	18.29	8.53	1.017	3.50E-39
BRIX1	ENSG0000	13	5.58	1.089	2.97E-39
PECAM1	ENSG0000	11.39	3.285	1.532	2.92E-39
CANT1	ENSG0000	14.81	6.46	1.084	2.58E-39
AGBL5	ENSG0000	8.21	3.445	1.051	2.57E-39
RPSA	ENSG0000	680.38	312.941	1.118	2.42E-39
GCGR	ENSG0000	4.47	57.661	-3.423	2.38E-39
IMPDH2	ENSG0000	67.662	29.13	1.188	2.28E-39
TIAF1	ENSG0000	0.48	2.72	-1.33	2.07E-39
FLYWCH2	ENSG0000	14.31	4.85	1.388	1.74E-39
ELOVL1	ENSG0000	37.6	17.105	1.092	1.59E-39
FAM83H	ENSG0000	14.96	5.055	1.398	1.52E-39
S100A8	ENSG0000	3.42	29.025	-2.764	1.36E-39
C16orf59	ENSG0000	2.19	0.37	1.219	1.35E-39
SKA3	ENSG0000	1.16	0.07	1.013	1.35E-39
CBFA2T3	ENSG0000	0.57	2.535	-1.171	1.29E-39
IRAK1	ENSG0000	36.75	13.79	1.352	1.22E-39
STXBP6	ENSG0000	6.17	1.34	1.615	9.91E-40
MORN2	ENSG0000	12.46	5.615	1.025	9.02E-40
ATP6V0B	ENSG0000	142.339	68.669	1.041	8.52E-40
DHX9	ENSG0000	41.55	19.855	1.029	8.48E-40
H2AFX	ENSG0000	14.94	4.97	1.417	8.39E-40
RPL37P6	ENSG0000	2.03	0	1.599	8.18E-40
CXCL12	ENSG0000	11.66	60.325	-2.276	7.53E-40
SKA1	ENSG0000	1.33	0.04	1.164	7.50E-40
ZFAS1	ENSG0000	81.002	27.78	1.511	7.16E-40
CPEB3	ENSG0000	1.11	3.71	-1.158	7.09E-40
THBS4	ENSG0000	3.68	0.15	2.025	6.22E-40
MIF4GD	ENSG0000	16.58	7.17	1.106	5.98E-40
DAXX	ENSG0000	23.62	10.495	1.099	5.71E-40
RPS19	ENSG0000	1703.043	813.895	1.064	4.40E-40
C6orf48	ENSG0000	78.378	31.529	1.287	4.33E-40

SNX29P2	ENSG0000	0.59	3.925	-1.631	4.19E-40
UBE2A	ENSG0000	25.029	11.63	1.043	4.00E-40
UBE2Q1	ENSG0000	39.719	15.774	1.279	3.97E-40
BPGM	ENSG0000	8.15	3.535	1.013	3.91E-40
RPL14P1	ENSG0000	9.55	3.85	1.121	3.84E-40
FAM195B	ENSG0000	68.721	30.14	1.163	3.78E-40
BUB1	ENSG0000	1.72	0.11	1.293	3.44E-40
RPL5	ENSG0000	560.899	273.187	1.035	3.44E-40
EIF4BP7	ENSG0000	1.68	0.34	1	3.21E-40
VPS33A	ENSG0000	7.39	3.075	1.042	3.20E-40
STEAP3	ENSG0000	19.849	62.63	-1.61	3.11E-40
RP11-250	ENSG0000	0.5	2.48	-1.214	3.11E-40
CNTN3	ENSG0000	0.08	2.035	-1.491	2.99E-40
LRPAP1	ENSG0000	95.259	43.646	1.108	2.91E-40
HCFC1R1	ENSG0000	44.622	21.699	1.007	2.88E-40
RPL31	ENSG0000	1529.028	743.015	1.04	2.85E-40
DAPK2	ENSG0000	4.37	1.105	1.351	2.77E-40
ANLN	ENSG0000	2.26	0.175	1.472	2.75E-40
NUSAP1	ENSG0000	8.31	1.675	1.799	2.72E-40
TACC3	ENSG0000	10.26	3.13	1.447	2.48E-40
MRPL15	ENSG0000	50.699	24.155	1.039	2.07E-40
HJURP	ENSG0000	2.22	0.245	1.371	2.04E-40
RPL4	ENSG0000	1141.566	557.933	1.032	2.02E-40
SQSTM1	ENSG0000	460.225	152.9	1.583	1.68E-40
SAMD1	ENSG0000	17.89	7.82	1.099	1.55E-40
CD320	ENSG0000	20.33	9.475	1.026	1.42E-40
TRAIP	ENSG0000	1.87	0.385	1.051	1.16E-40
DPM3	ENSG0000	133.61	60.894	1.121	1.08E-40
ASPG	ENSG0000	7.77	75.475	-3.124	8.33E-41
PBK	ENSG0000	2.1	0.1	1.495	8.04E-41
CENPL	ENSG0000	1.82	0.34	1.073	8.02E-41
CCHCR1	ENSG0000	11.72	4.52	1.204	6.93E-41
MCM7	ENSG0000	32.919	12.145	1.368	6.89E-41
EMC2	ENSG0000	31.99	14.01	1.136	6.04E-41
MT2A	ENSG0000	261.977	2324.572	-3.145	5.54E-41
NDUFA4L2	ENSG0000	14.62	3.17	1.905	4.81E-41
ZSCAN16-	ENSG0000	8.45	3.54	1.058	4.19E-41
SPCS2P4	ENSG0000	30.96	12.14	1.282	2.97E-41
PVALB	ENSG0000	0.11	1.22	-1	2.77E-41
FOXM1	ENSG0000	5.72	0.91	1.815	2.70E-41
RAN	ENSG0000	208.441	81.905	1.337	2.40E-41
WDR76	ENSG0000	2.02	0.295	1.222	2.31E-41
PHPT1	ENSG0000	99.787	48.509	1.026	2.25E-41
SKA2	ENSG0000	14.76	6.375	1.096	2.07E-41
S100A10	ENSG0000	241.168	54.345	2.129	2.01E-41
ITGA6	ENSG0000	10.25	2.74	1.589	1.72E-41
MOGAT2	ENSG0000	5.55	49.036	-2.933	1.71E-41
RP11-524	ENSG0000	0.37	2.685	-1.427	1.64E-41
PTMA	ENSG0000	508.498	243.57	1.059	1.61E-41
OLFML2A	ENSG0000	1.48	0.175	1.078	1.57E-41
RAB4A	ENSG0000	37.219	17.995	1.009	1.56E-41
RPL23AP8	ENSG0000	9.59	4.1	1.054	1.44E-41
RMI2	ENSG0000	3.39	0.47	1.578	1.38E-41
CDK4	ENSG0000	47.33	19.38	1.246	1.22E-41
TSEN15	ENSG0000	11.54	5.17	1.023	1.19E-41
NDRG3	ENSG0000	7.09	2.73	1.117	1.16E-41
MT-ATP6	ENSG0000	11214.67	31942.73	-1.51	1.16E-41
RHOA	ENSG0000	154.396	75.585	1.021	1.12E-41

LYVE1	ENSG0000	0.83	7.055	-2.138	1.12E-41
DTL	ENSG0000	2.23	0.145	1.496	1.04E-41
CH17-340	ENSG0000	9.38	4.11	1.022	8.62E-42
PTGES3	ENSG0000	137.758	65.443	1.062	8.02E-42
C3P1	ENSG0000	11.36	51.089	-2.075	5.67E-42
TIPRL	ENSG0000	13.95	6.325	1.029	5.64E-42
MYL12B	ENSG0000	310.295	142.938	1.113	5.47E-42
LINC0134	ENSG0000	1.2	8.645	-2.132	5.14E-42
CYP26A1	ENSG0000	0.28	4.96	-2.219	5.10E-42
TBCB	ENSG0000	58.351	26.444	1.113	4.50E-42
CENPU	ENSG0000	4.69	0.885	1.594	4.48E-42
TAX1BP1	ENSG0000	42.499	18.625	1.148	4.00E-42
OIP5	ENSG0000	1.88	0.17	1.3	3.71E-42
ARF1	ENSG0000	269.025	132.775	1.013	3.51E-42
HMGN2	ENSG0000	225.736	103.877	1.112	3.38E-42
CCT5	ENSG0000	82.509	34.429	1.237	3.35E-42
STARD5	ENSG0000	6.45	19.359	-1.45	3.13E-42
TTC36	ENSG0000	4	63.26	-3.684	2.66E-42
BYSL	ENSG0000	12.53	4.635	1.264	2.49E-42
TSLP	ENSG0000	0.33	2.1	-1.221	2.45E-42
WDYHV1	ENSG0000	8.96	3.605	1.113	2.23E-42
LCAT	ENSG0000	32.421	153.649	-2.21	2.18E-42
ECHDC2	ENSG0000	148.549	458.109	-1.618	1.73E-42
PRIM1	ENSG0000	5.97	1.56	1.445	1.73E-42
C17orf49	ENSG0000	55.241	23.97	1.171	1.71E-42
CORO1B	ENSG0000	47.55	23.209	1.004	1.49E-42
CHMP4B	ENSG0000	57.181	27.285	1.041	1.47E-42
STT3A	ENSG0000	76.882	32.829	1.203	1.44E-42
RPS10	ENSG0000	973.069	427.846	1.184	1.26E-42
ANO10	ENSG0000	10.95	4.33	1.165	1.20E-42
LINC0046	ENSG0000	10.37	4.585	1.026	1.15E-42
AC004538	ENSG0000	0.14	2.04	-1.415	9.74E-43
ALG1L	ENSG0000	7.27	0.325	2.642	9.46E-43
MT1X	ENSG0000	145.29	1597.997	-3.45	8.62E-43
TOMM40	ENSG0000	36.99	16.13	1.149	8.57E-43
SNX15	ENSG0000	9.93	4.415	1.013	8.06E-43
TMED9	ENSG0000	183.304	91.034	1.002	6.92E-43
KIF2C	ENSG0000	2.21	0.17	1.456	6.33E-43
NDUFAF2	ENSG0000	25.06	11.515	1.058	6.33E-43
H2AFJ	ENSG0000	110.208	42.105	1.367	5.74E-43
POLR3C	ENSG0000	10.38	4.26	1.113	5.62E-43
RCAN1	ENSG0000	17.179	48.371	-1.441	5.50E-43
RP4-564F	ENSG0000	0.54	5.865	-2.156	5.41E-43
RPL28	ENSG0000	1238.089	565.741	1.129	5.32E-43
IL1RAP	ENSG0000	6.41	25.615	-1.845	5.13E-43
RPL7	ENSG0000	1178.47	467.589	1.332	4.79E-43
KIF4A	ENSG0000	1.87	0.07	1.423	4.49E-43
SLC35B2	ENSG0000	32.739	13.99	1.17	4.34E-43
HMMR	ENSG0000	2.54	0.2	1.561	4.19E-43
ANXA11	ENSG0000	86.99	38.454	1.157	3.81E-43
COL4A1	ENSG0000	26.299	4.465	2.321	3.62E-43
PGA4	ENSG0000	0.05	1.7	-1.362	3.57E-43
MYBL2	ENSG0000	4.07	0.19	2.091	3.56E-43
TAGLN2	ENSG0000	252.545	76.164	1.716	3.34E-43
C6orf1	ENSG0000	20.87	9.29	1.088	3.18E-43
TRAF2	ENSG0000	10.5	4.355	1.103	2.86E-43
RSPO3	ENSG0000	0.06	1.57	-1.278	2.81E-43
SLC39A7	ENSG0000	107.157	45.144	1.229	2.79E-43

PAFAH1B3	ENSG0000	14.81	3.225	1.904	2.54E-43
LAMC1	ENSG0000	26.829	5.775	2.038	2.21E-43
HHIP	ENSG0000	0.06	1.475	-1.223	2.21E-43
EEF1D	ENSG0000	621.107	280.43	1.144	2.20E-43
HSPB1	ENSG0000	644.572	168.996	1.925	2.04E-43
HMGB2	ENSG0000	34.871	11.52	1.519	1.95E-43
TUBG1	ENSG0000	24.47	8.475	1.427	1.90E-43
ADIPOR1	ENSG0000	66.791	32.8	1.004	1.88E-43
VAMP5	ENSG0000	90.88	33.767	1.402	1.87E-43
UGGT1	ENSG0000	14.17	5.615	1.197	1.62E-43
RP11-464	ENSG0000	2.61	0.35	1.419	1.55E-43
FAM50A	ENSG0000	61.922	25.359	1.255	1.46E-43
NUDT2	ENSG0000	20.569	9.42	1.05	1.35E-43
CTNNA1	ENSG0000	88.812	42.721	1.039	1.10E-43
CBX8	ENSG0000	4.54	1.57	1.108	1.07E-43
SMARCE1	ENSG0000	49.05	20.464	1.221	1.03E-43
SYT9	ENSG0000	0.01	1.26	-1.162	8.39E-44
DEFA1	ENSG0000	0.09	2.755	-1.784	7.86E-44
CDCA8	ENSG0000	2.69	0.29	1.516	7.21E-44
RPLP0P6	ENSG0000	13.89	4.415	1.459	7.08E-44
ACAD11	ENSG0000	12.74	40.844	-1.607	7.07E-44
CENPA	ENSG0000	2.09	0.12	1.464	6.70E-44
LPA	ENSG0000	1.21	8.365	-2.083	6.63E-44
HNRNPA1	ENSG0000	363.698	146.569	1.305	6.58E-44
LIG1	ENSG0000	16.18	6.24	1.247	6.01E-44
SLC35F6	ENSG0000	16.66	7.18	1.11	4.73E-44
SNRPF	ENSG0000	27.971	13.225	1.026	4.38E-44
POC1A	ENSG0000	4.81	1.72	1.095	4.36E-44
DHODH	ENSG0000	14.04	49.561	-1.749	3.99E-44
CETN2	ENSG0000	20.93	9.26	1.096	3.54E-44
UTP14A	ENSG0000	7.05	2.75	1.102	2.94E-44
SLC52A2	ENSG0000	14.78	5.38	1.307	2.94E-44
MAP1S	ENSG0000	14.04	5.995	1.104	2.83E-44
COX20	ENSG0000	40.969	18.425	1.111	2.80E-44
SRP9	ENSG0000	168.685	79.5	1.076	2.63E-44
HEXA	ENSG0000	76.68	33.93	1.153	2.55E-44
OLA1	ENSG0000	40.941	16.146	1.291	2.43E-44
PDIA3	ENSG0000	370.671	173.452	1.091	2.37E-44
FAM99B	ENSG0000	0.23	3.835	-1.975	1.97E-44
ANXA2	ENSG0000	254.866	63.065	1.998	1.96E-44
RPL39	ENSG0000	1924.809	846.93	1.183	1.93E-44
RPL15	ENSG0000	755.139	377.046	1	1.90E-44
RPLP0	ENSG0000	1398.049	655.112	1.092	1.85E-44
ADAM15	ENSG0000	42.241	16.735	1.286	1.78E-44
FABP5	ENSG0000	16.66	2.88	2.186	1.76E-44
ADSL	ENSG0000	31.91	14.58	1.079	1.69E-44
VAT1	ENSG0000	49.37	16.41	1.533	1.56E-44
H2AFY	ENSG0000	56.539	24.299	1.185	1.46E-44
ZNF581	ENSG0000	9.69	4.23	1.031	1.32E-44
RP11-334	ENSG0000	6.87	0.46	2.43	1.24E-44
FANCI	ENSG0000	3.08	0.565	1.382	1.22E-44
EEF1G	ENSG0000	721.421	356.435	1.015	1.20E-44
GINS2	ENSG0000	2.4	0.41	1.27	1.15E-44
ATP6V1H	ENSG0000	23.62	11.17	1.016	1.12E-44
MELK	ENSG0000	2.23	0.1	1.554	1.08E-44
RP11-51C	ENSG0000	21.64	8.325	1.28	1.06E-44
GPM6A	ENSG0000	0.16	1.64	-1.186	9.84E-45
KIF20A	ENSG0000	2.79	0.13	1.746	9.12E-45

KDM8	ENSG0000	5.66	45.724	-2.811	9.08E-45
SLC39A1	ENSG0000	72.098	29.34	1.269	8.80E-45
CDC45	ENSG0000	3.07	0.32	1.624	7.92E-45
RPL35P5	ENSG0000	5.17	1.38	1.374	7.81E-45
NSA2	ENSG0000	56.629	26.857	1.049	7.30E-45
PNPLA7	ENSG0000	1.7	5.1	-1.176	7.03E-45
CDC123	ENSG0000	33.16	16.075	1	6.77E-45
PSME2	ENSG0000	253.755	120.229	1.071	5.98E-45
MRPS17	ENSG0000	16.48	7.29	1.076	5.73E-45
TSNAX	ENSG0000	14.6	6.555	1.046	5.62E-45
TPX2	ENSG0000	6	0.765	1.988	5.60E-45
MFSD2A	ENSG0000	3.81	77.691	-4.032	5.52E-45
MRPL21	ENSG0000	61.23	29.245	1.041	5.43E-45
RPS23	ENSG0000	1376.605	648.044	1.086	5.30E-45
NPC2	ENSG0000	121.456	52.019	1.208	5.03E-45
TRIM28	ENSG0000	143.419	66.495	1.097	4.58E-45
IQGAP3	ENSG0000	2.35	0.12	1.581	4.48E-45
DCAF13	ENSG0000	17.179	6.265	1.323	4.36E-45
HSP90AB1	ENSG0000	400.622	139.516	1.515	4.29E-45
NDUFS4	ENSG0000	82.081	38.374	1.077	4.28E-45
SLC25A47	ENSG0000	13.85	213.567	-3.853	3.91E-45
COA3	ENSG0000	130.119	62.989	1.035	3.77E-45
COX7A2	ENSG0000	317.364	149.805	1.078	3.75E-45
RRP9	ENSG0000	12.34	5.515	1.034	3.72E-45
CKS2	ENSG0000	31.349	8.62	1.75	3.56E-45
CYP2C19	ENSG0000	0.33	6.81	-2.554	3.42E-45
ARV1	ENSG0000	17.67	8.02	1.049	3.26E-45
TBCC	ENSG0000	11.02	4.93	1.019	3.26E-45
OLFML2B	ENSG0000	2.78	0.275	1.568	3.12E-45
TATDN3	ENSG0000	7.83	3.265	1.05	3.12E-45
ZNF687	ENSG0000	11.88	4.36	1.265	3.11E-45
GARS	ENSG0000	59.231	24.455	1.243	2.68E-45
NCAPH	ENSG0000	2.55	0.17	1.601	2.29E-45
RP11-132	ENSG0000	1.1	6.998	-1.929	2.20E-45
AP006285.	ENSG0000	0.06	1.265	-1.095	2.02E-45
PES1	ENSG0000	40.87	19.35	1.041	2.02E-45
PKN1	ENSG0000	44.609	13.205	1.683	1.92E-45
BROX	ENSG0000	10.95	4.735	1.059	1.51E-45
CXCL2	ENSG0000	18.181	119.784	-2.655	1.51E-45
RPL7P1	ENSG0000	20.549	5	1.845	1.49E-45
C17orf89	ENSG0000	74.47	35.49	1.048	1.48E-45
EXO1	ENSG0000	1.81	0.1	1.353	1.41E-45
HAND2	ENSG0000	0.4	5.02	-2.104	1.16E-45
HM13	ENSG0000	160.162	70.709	1.168	1.15E-45
EIF3E	ENSG0000	194.253	69.545	1.469	1.00E-45
RPN1	ENSG0000	160.362	76.362	1.061	9.65E-46
PDE6D	ENSG0000	11.73	5.08	1.066	9.55E-46
MCM3	ENSG0000	20.53	5.435	1.742	9.00E-46
BTF3	ENSG0000	338.66	168.376	1.004	8.92E-46
NUF2	ENSG0000	2.48	0.13	1.623	8.54E-46
PPP1R11	ENSG0000	42.579	19.67	1.076	8.00E-46
ITGB3BP	ENSG0000	8.56	3.155	1.202	6.97E-46
PA2G4	ENSG0000	52.57	23.56	1.125	6.70E-46
PLGLA	ENSG0000	4.32	22.95	-2.171	6.68E-46
MKI67	ENSG0000	2.9	0.17	1.737	6.18E-46
ADGRG7	ENSG0000	0.72	4.425	-1.657	4.94E-46
DNPH1	ENSG0000	125.6	57.579	1.112	3.92E-46
BLVRA	ENSG0000	19.46	3.145	2.303	3.46E-46

HLA-A	ENSG0000	714.504	165.214	2.106	3.22E-46
AKR1C3	ENSG0000	351.236	89.588	1.959	3.03E-46
LSM7	ENSG0000	71.848	34.91	1.021	2.95E-46
RPS12P21	ENSG0000	0.07	1.17	-1.02	2.74E-46
MSH2	ENSG0000	5.18	1.415	1.356	2.42E-46
RP1-102E	ENSG0000	0.27	2.495	-1.46	2.12E-46
EFNA4	ENSG0000	8.27	2.885	1.255	2.00E-46
NCAPG	ENSG0000	2.59	0.115	1.687	1.63E-46
RPL36	ENSG0000	739.803	336.869	1.133	1.54E-46
ENY2	ENSG0000	127.009	62.88	1.003	1.36E-46
AIDA	ENSG0000	24.519	10.875	1.104	1.27E-46
RPL23	ENSG0000	944.625	439.386	1.102	1.17E-46
CCT6A	ENSG0000	83.169	34.536	1.244	1.14E-46
RRM1	ENSG0000	13.68	4.735	1.356	1.14E-46
NDUFS8	ENSG0000	131.06	64.625	1.009	9.08E-47
DEFA1B	ENSG0000	0	2.35	-1.744	8.92E-47
TCF19	ENSG0000	4.27	0.44	1.872	6.76E-47
RP3-342P	ENSG0000	0.1	3.96	-2.173	5.87E-47
CMSS1	ENSG0000	13.65	5.695	1.13	5.50E-47
RPS16	ENSG0000	1328.515	622.443	1.093	5.50E-47
TLCD1	ENSG0000	16.11	4.87	1.543	4.12E-47
GNB2L1	ENSG0000	891.937	427.994	1.058	4.11E-47
NOMO1	ENSG0000	31.299	13.774	1.128	4.10E-47
VARS	ENSG0000	36.521	13.985	1.324	3.20E-47
SPC25	ENSG0000	2.11	0.16	1.423	3.18E-47
HBA1	ENSG0000	10	98.975	-3.184	2.99E-47
RP11-196	ENSG0000	0.57	2.975	-1.34	2.77E-47
CALR	ENSG0000	813.782	337.302	1.268	2.50E-47
NPM1	ENSG0000	485.355	170.378	1.505	2.36E-47
IGFALS	ENSG0000	2.83	40.84	-3.449	2.19E-47
FAM163B	ENSG0000	0.09	2.415	-1.648	2.16E-47
IGBP1	ENSG0000	20.49	8.45	1.185	2.05E-47
PDCD2L	ENSG0000	6.35	2.57	1.042	1.95E-47
CLIC1	ENSG0000	143.867	43.331	1.708	1.91E-47
UXS1	ENSG0000	15.49	5.405	1.364	1.89E-47
RP11-452	ENSG0000	11.78	4.12	1.32	1.81E-47
FAM189B	ENSG0000	12.53	4.445	1.313	1.79E-47
RPS27	ENSG0000	3269.149	1428.86	1.193	1.73E-47
MAD2L1	ENSG0000	3.9	0.535	1.675	1.61E-47
SNRPD3	ENSG0000	99.401	46.269	1.087	1.58E-47
ZMAT2	ENSG0000	48.509	23.279	1.028	1.50E-47
PYGO2	ENSG0000	15.31	6.875	1.05	1.25E-47
MRPS23	ENSG0000	28.859	9.985	1.443	1.07E-47
GRK6	ENSG0000	10.62	4.71	1.025	1.04E-47
CCDC167	ENSG0000	49.179	20.844	1.2	8.38E-48
ASF1B	ENSG0000	3.27	0.28	1.738	7.91E-48
EIF3H	ENSG0000	142.754	54.924	1.362	7.81E-48
THOC3	ENSG0000	22.43	9.25	1.193	6.88E-48
RPS13	ENSG0000	649.415	322.018	1.01	6.76E-48
RPS4X	ENSG0000	486.365	194.576	1.317	6.51E-48
EXOC4	ENSG0000	11.34	5.17	1	6.07E-48
MESP1	ENSG0000	3.86	0.96	1.31	6.00E-48
NOMO2	ENSG0000	83.609	35.69	1.205	5.96E-48
CYC1	ENSG0000	157.324	75.334	1.052	5.84E-48
FKBP1A	ENSG0000	88.56	40.05	1.125	5.76E-48
RPS18	ENSG0000	2144.461	874.278	1.293	5.72E-48
CHCHD6	ENSG0000	8.61	3.59	1.066	5.59E-48
UCK2	ENSG0000	18.22	6	1.457	5.06E-48

C8orf33	ENSG0000	18.379	7.305	1.223	4.89E-48
PMS2P1	ENSG0000	5.08	2.01	1.014	4.84E-48
XRCC6	ENSG0000	110.598	53.725	1.028	4.22E-48
HRAS	ENSG0000	32.271	15.19	1.039	3.00E-48
EIF3M	ENSG0000	133.963	61.922	1.101	2.64E-48
CYTH2	ENSG0000	23.18	9.76	1.168	2.22E-48
TYMS	ENSG0000	16.4	3.05	2.103	2.21E-48
RP11-6B4.	ENSG0000	0.17	4.765	-2.301	2.21E-48
LYPLAL1	ENSG0000	36.221	14.405	1.273	2.19E-48
ZCRB1	ENSG0000	33.481	15.205	1.089	1.97E-48
C1orf35	ENSG0000	12.7	5.775	1.016	1.91E-48
RPL7P9	ENSG0000	28.391	9.16	1.533	1.79E-48
DSN1	ENSG0000	7.32	2.66	1.185	1.72E-48
ITGB1BP1	ENSG0000	32.839	12.855	1.288	1.65E-48
C11orf98	ENSG0000	63.592	30.65	1.029	1.59E-48
NCAPH2	ENSG0000	23.71	10.6	1.091	1.53E-48
RFC4	ENSG0000	12.42	3.89	1.457	1.42E-48
PYCR2	ENSG0000	40.649	18.06	1.128	1.26E-48
TOPORS-/	ENSG0000	9.65	3.685	1.185	1.22E-48
NDUFA4	ENSG0000	120.508	59.309	1.011	1.15E-48
PGA3	ENSG0000	0.06	2.469	-1.71	8.52E-49
MRPL47	ENSG0000	30.3	14.075	1.054	8.16E-49
AGPAT1	ENSG0000	46.821	21.794	1.069	7.90E-49
EFCAB11	ENSG0000	4.05	1.51	1.009	6.94E-49
MT1F	ENSG0000	7	191.386	-4.588	6.81E-49
FAM20B	ENSG0000	8.97	3.605	1.114	6.50E-49
PTRH2	ENSG0000	27.851	13.168	1.026	6.31E-49
POLD1	ENSG0000	13.28	4.905	1.274	6.07E-49
HSPB11	ENSG0000	21.91	10.425	1.004	5.79E-49
LIFR	ENSG0000	0.71	3.515	-1.401	4.82E-49
C20orf27	ENSG0000	22.11	9.443	1.146	4.31E-49
C15orf40	ENSG0000	15.5	7.065	1.033	4.08E-49
FTH1	ENSG0000	3615.3	1496.627	1.272	3.64E-49
CEP131	ENSG0000	5.98	2.185	1.132	3.42E-49
MBL1P	ENSG0000	0.22	3.06	-1.735	3.31E-49
FTH1P23	ENSG0000	2.4	0.63	1.061	2.57E-49
PDCD5	ENSG0000	85.241	41.33	1.027	2.56E-49
HBB	ENSG0000	15.86	167.277	-3.319	2.55E-49
MCM6	ENSG0000	7.88	1.485	1.837	2.31E-49
CTB-25B1.	ENSG0000	9.2	3.654	1.132	2.22E-49
RP11-641.	ENSG0000	174.126	60.058	1.52	2.02E-49
COPZ1	ENSG0000	81.882	40.161	1.01	1.86E-49
RPL29	ENSG0000	1049.145	487.919	1.103	1.66E-49
HSPB1P1	ENSG0000	5.31	0	2.658	1.66E-49
MT1JP	ENSG0000	0.05	4.62	-2.42	1.61E-49
RPL14	ENSG0000	706.917	314.714	1.165	1.55E-49
CDT1	ENSG0000	3.68	0.265	1.887	1.30E-49
MCM2	ENSG0000	6.75	0.735	2.159	1.21E-49
KIAA0101	ENSG0000	9.86	1.51	2.113	9.70E-50
BCL2L12	ENSG0000	19.401	8.03	1.176	9.33E-50
AKIP1	ENSG0000	12.78	5.335	1.121	7.69E-50
BOP1	ENSG0000	28.041	9.04	1.532	7.46E-50
SPCS1	ENSG0000	167.846	78.948	1.079	7.46E-50
FBL	ENSG0000	123.391	47.738	1.352	7.33E-50
TRIM52-A	ENSG0000	9.2	3.35	1.229	7.07E-50
HNRNPA3	ENSG0000	124.077	57.412	1.098	4.79E-50
GGPS1	ENSG0000	12.93	5.865	1.021	4.77E-50
CDCA3	ENSG0000	5.67	0.685	1.985	4.65E-50

ADRA2B	ENSG0000	0.41	1.885	-1.033	4.15E-50
TIMM10	ENSG0000	60.48	29.36	1.018	4.07E-50
AURKB	ENSG0000	5.25	0.53	2.03	3.98E-50
C7orf50	ENSG0000	47.619	20.535	1.175	3.79E-50
DARS2	ENSG0000	10.48	3.105	1.484	3.52E-50
TPM3	ENSG0000	124.154	46.16	1.408	3.43E-50
SMARCA4	ENSG0000	29.769	13.425	1.093	2.20E-50
CLTA	ENSG0000	122.402	57.029	1.089	1.99E-50
RPL32	ENSG0000	1338.312	560.083	1.255	1.99E-50
SRD5A3	ENSG0000	15.24	6.015	1.211	1.74E-50
RACGAP1	ENSG0000	4.61	0.705	1.718	1.73E-50
POLA2	ENSG0000	7.17	2.465	1.238	1.69E-50
SEMA3F	ENSG0000	5.44	1.655	1.278	1.43E-50
SLC50A1	ENSG0000	65.289	24.135	1.399	1.38E-50
RRM2	ENSG0000	9.26	0.595	2.685	1.36E-50
TXN	ENSG0000	347.458	121.873	1.504	1.17E-50
TMEM147	ENSG0000	69.05	28.196	1.263	1.04E-50
GNPAT	ENSG0000	27.72	11.88	1.157	9.80E-51
FAM103A1	ENSG0000	15.7	7.285	1.011	9.60E-51
KIAA1462	ENSG0000	3.31	0.52	1.504	9.40E-51
HMGA1	ENSG0000	36.319	6.9	2.24	8.96E-51
TALDO1	ENSG0000	149.955	65.534	1.182	8.91E-51
RAC1	ENSG0000	116.994	54.913	1.077	8.53E-51
PPIH	ENSG0000	16.36	6.63	1.186	8.15E-51
CENPH	ENSG0000	3.04	0.59	1.345	7.84E-51
POLR2L	ENSG0000	155.448	74.78	1.046	6.25E-51
LSM8	ENSG0000	21.201	8.135	1.281	6.01E-51
RECQL4	ENSG0000	8.65	1.435	1.987	6.01E-51
RPL27A	ENSG0000	1599.05	693.57	1.204	5.94E-51
RHOC	ENSG0000	174.755	63.284	1.451	4.75E-51
FLVCR1	ENSG0000	3.43	0.705	1.378	3.91E-51
ALG3	ENSG0000	57.289	26.005	1.11	3.84E-51
MT1G	ENSG0000	40.209	2587.601	-5.973	3.31E-51
CLN6	ENSG0000	19.69	9.18	1.023	2.95E-51
RPS2P55	ENSG0000	2.26	0.33	1.293	2.92E-51
RP11-830	ENSG0000	0.04	1.39	-1.2	2.41E-51
NEK2	ENSG0000	3.21	0.11	1.923	2.38E-51
TCEB2	ENSG0000	295.517	138.164	1.091	2.37E-51
AC005077	ENSG0000	0.52	6.74	-2.348	2.36E-51
RPLP2	ENSG0000	879.597	343.329	1.355	2.02E-51
CKS1BP3	ENSG0000	5.44	0.475	2.126	1.92E-51
TBCE	ENSG0000	16.95	7.4	1.096	1.50E-51
NUTF2	ENSG0000	55.861	25.134	1.121	1.43E-51
NFKBIL1	ENSG0000	18.87	8.68	1.038	1.09E-51
CCDC34	ENSG0000	5.74	1.22	1.602	1.03E-51
RP11-452	ENSG0000	37.569	5.96	2.47	9.36E-52
GMPS	ENSG0000	19.41	6.88	1.373	9.06E-52
AADAT	ENSG0000	1.83	11.5	-2.143	8.81E-52
NDUFB6	ENSG0000	61.601	29.35	1.044	8.17E-52
RPL41	ENSG0000	2353.512	831.227	1.5	7.63E-52
VRK1	ENSG0000	4.99	1.65	1.177	7.51E-52
BUD31	ENSG0000	64.382	29.16	1.116	6.44E-52
RPL24	ENSG0000	884.733	404.767	1.126	5.84E-52
RAD51C	ENSG0000	13.68	5.425	1.192	5.70E-52
TMEM183	ENSG0000	27.02	12.51	1.052	5.56E-52
COL15A1	ENSG0000	3.03	0.17	1.784	4.85E-52
GNAO1	ENSG0000	0.68	7.76	-2.382	4.76E-52
NDC80	ENSG0000	2.91	0.18	1.728	3.06E-52

CAP2	ENSG0000	8.86	1.345	2.072	3.05E-52
CYB5R1	ENSG0000	47.698	20.285	1.194	2.90E-52
RHNO1	ENSG0000	6.63	1.77	1.462	2.76E-52
PHB	ENSG0000	120.25	54.451	1.129	2.57E-52
PRSS53	ENSG0000	0.23	4.55	-2.174	2.46E-52
KIAA0196	ENSG0000	15.47	6.095	1.215	2.18E-52
OIT3	ENSG0000	2.66	15.075	-2.135	1.80E-52
ESM1	ENSG0000	3.22	0.09	1.953	1.59E-52
MYL6B	ENSG0000	27.219	9.079	1.485	1.42E-52
RPL23AP4	ENSG0000	644.751	238.359	1.432	1.41E-52
SNRPB2	ENSG0000	28.699	12.59	1.128	1.39E-52
LYRM4	ENSG0000	16	6.435	1.193	1.35E-52
SAE1	ENSG0000	33.261	12.505	1.343	1.26E-52
ALYREF	ENSG0000	37.749	13.9	1.379	1.14E-52
HSPA4	ENSG0000	68.029	27.19	1.292	1.13E-52
FAM99A	ENSG0000	1.1	37.31	-4.189	1.13E-52
C8orf76	ENSG0000	19.81	8.225	1.174	8.54E-53
MYL6	ENSG0000	1006.338	476.208	1.078	8.54E-53
RP11-620	ENSG0000	5.08	1.055	1.565	8.54E-53
UQCR10	ENSG0000	135.532	66.201	1.023	7.94E-53
MAGOHB	ENSG0000	17.95	7.98	1.077	7.20E-53
PPM1G	ENSG0000	39.769	17.025	1.177	6.71E-53
DCTPP1	ENSG0000	22.201	9.24	1.18	5.91E-53
TOMM22	ENSG0000	38.84	17.85	1.08	5.91E-53
PBXIP1	ENSG0000	38.369	13.51	1.44	5.51E-53
LCMT1	ENSG0000	12.62	5.335	1.104	5.15E-53
MIR4435-	ENSG0000	33.1	4.45	2.645	4.40E-53
CHAF1A	ENSG0000	5.12	1.39	1.357	4.33E-53
H2AFZ	ENSG0000	99.808	32.785	1.577	4.21E-53
CDC25C	ENSG0000	1.84	0.055	1.429	3.83E-53
RPL6	ENSG0000	779.064	371.739	1.065	3.44E-53
BOLA2	ENSG0000	10.8	2.74	1.658	3.31E-53
DYNC1H1	ENSG0000	65.922	29.81	1.119	3.19E-53
ATP6V1E1	ENSG0000	67.568	28.939	1.196	3.07E-53
RPL35A	ENSG0000	803.914	353.091	1.185	3.04E-53
ATP6V0E1	ENSG0000	207.691	99.105	1.06	2.92E-53
MAPK3	ENSG0000	24.54	9.99	1.217	2.16E-53
C14orf18C	ENSG0000	0.01	1.21	-1.13	2.10E-53
KCNN2	ENSG0000	0.25	4.35	-2.098	1.91E-53
PLOD3	ENSG0000	60.601	23.364	1.338	1.88E-53
MYEOV2	ENSG0000	94.693	41.148	1.183	1.77E-53
PDCL3	ENSG0000	11.29	4.515	1.156	1.68E-53
CENPF	ENSG0000	6.14	0.295	2.463	1.12E-53
TRAPPC2L	ENSG0000	54.651	23.875	1.162	1.09E-53
NECAB3	ENSG0000	21.341	8.84	1.183	1.03E-53
RPS20	ENSG0000	1550.372	610.077	1.344	9.99E-54
ASPM	ENSG0000	2.96	0.125	1.816	8.72E-54
HN1	ENSG0000	64.208	19.065	1.7	8.00E-54
TATDN1	ENSG0000	22.869	8.72	1.296	7.80E-54
KLHDC3	ENSG0000	61.12	29.215	1.04	5.23E-54
SPSB2	ENSG0000	6.75	2.24	1.258	4.55E-54
CDKN2C	ENSG0000	7.91	1.415	1.883	4.49E-54
HDAC11	ENSG0000	8.49	2.1	1.614	4.48E-54
CTD-224C	ENSG0000	0.3	2.59	-1.465	3.83E-54
RPS11	ENSG0000	1591.31	734.489	1.114	3.62E-54
CCBE1	ENSG0000	0.06	1.185	-1.044	3.49E-54
PFDN4	ENSG0000	21.78	8.525	1.258	3.44E-54
PUF60	ENSG0000	112.259	47.77	1.216	3.24E-54

TFPT	ENSG0000	11.74	4.665	1.169	2.46E-54
COX8A	ENSG0000	396.56	187.065	1.08	2.35E-54
CCT4	ENSG0000	57.519	25.355	1.151	2.01E-54
RPL27	ENSG0000	1270.164	553.58	1.197	2.01E-54
TMEM50A	ENSG0000	50.779	20.175	1.29	2.01E-54
CD63	ENSG0000	685.588	273.832	1.321	1.84E-54
RPS14	ENSG0000	955.822	419.331	1.187	1.83E-54
THOC7	ENSG0000	41.271	19.789	1.024	1.66E-54
CFL1	ENSG0000	437.093	184.272	1.242	1.62E-54
BIRC5	ENSG0000	6.81	0.505	2.376	1.57E-54
NME1-NM	ENSG0000	398.434	162.714	1.287	1.52E-54
RPS7	ENSG0000	895.903	394.121	1.183	1.42E-54
SNAPIN	ENSG0000	32.479	15.445	1.026	1.25E-54
EIF3D	ENSG0000	84.829	35.248	1.244	9.48E-55
ARPC3	ENSG0000	174.634	81.089	1.097	8.44E-55
GEMIN6	ENSG0000	11.22	5.08	1.007	7.82E-55
TXNDC12	ENSG0000	31.04	14.5	1.048	4.23E-55
FITM1	ENSG0000	1.31	7.18	-1.824	3.99E-55
DTYMK	ENSG0000	19.68	7.14	1.345	3.89E-55
YIPF3	ENSG0000	100.002	47.349	1.063	3.77E-55
SNX8	ENSG0000	17.73	5.43	1.542	3.64E-55
NDUFA8	ENSG0000	57.432	27.36	1.043	3.50E-55
CSE1L	ENSG0000	21.24	8.83	1.178	3.31E-55
TUBE1	ENSG0000	4.17	14.245	-1.56	2.56E-55
TCEB1	ENSG0000	67.911	26.575	1.321	2.54E-55
KIFC1	ENSG0000	4.37	0.205	2.156	2.31E-55
ATRAID	ENSG0000	74.109	34.835	1.068	2.28E-55
EMC3	ENSG0000	74.279	28.66	1.344	1.83E-55
BAG6	ENSG0000	179.805	88.398	1.016	1.81E-55
MAF1	ENSG0000	56.469	23.51	1.229	1.43E-55
DYNLL1	ENSG0000	246.304	98.267	1.317	1.33E-55
EXOSC4	ENSG0000	41.63	15.195	1.396	1.23E-55
PZP	ENSG0000	0.18	5.14	-2.379	1.20E-55
RPS21	ENSG0000	1270.692	486.517	1.383	9.69E-56
GGCT	ENSG0000	31.26	13.475	1.156	8.64E-56
CDCA5	ENSG0000	3.89	0.275	1.939	6.90E-56
PSME1	ENSG0000	221.797	100.384	1.136	5.05E-56
CD2BP2	ENSG0000	24.97	9.89	1.254	4.12E-56
COX7B	ENSG0000	168.51	73.211	1.192	4.09E-56
ARPC5	ENSG0000	86.762	29.82	1.51	3.85E-56
RNF187	ENSG0000	55.749	23.695	1.2	3.40E-56
MCM5	ENSG0000	21.45	4.025	2.16	2.99E-56
MRPL55	ENSG0000	113.739	49.114	1.195	2.74E-56
GPKOW	ENSG0000	14.82	6.73	1.033	2.64E-56
AURKA	ENSG0000	7.35	0.99	2.069	2.38E-56
NSMCE1	ENSG0000	51.61	24.375	1.052	2.32E-56
NDUFC2	ENSG0000	323.853	140.872	1.195	2.15E-56
PKMYT1	ENSG0000	5.72	0.695	1.987	1.95E-56
CNDP1	ENSG0000	0.49	17.989	-3.672	1.82E-56
ASNA1	ENSG0000	43.761	17.964	1.239	1.73E-56
RP11-215	ENSG0000	2.32	0.17	1.505	1.69E-56
UBE2M	ENSG0000	40.19	17.905	1.123	1.37E-56
FIBP	ENSG0000	36.801	17.76	1.011	8.92E-57
DOLK	ENSG0000	10.08	4.42	1.032	8.50E-57
MRPL13	ENSG0000	47.431	19.64	1.23	8.48E-57
UBA52	ENSG0000	852.821	414.275	1.04	8.44E-57
PSMB3	ENSG0000	225.627	100.068	1.165	7.70E-57
KBTBD11	ENSG0000	0.26	2.645	-1.532	7.57E-57

SUMO2	ENSG0000	146.859	49.28	1.556	7.57E-57
DRG1	ENSG0000	40.159	18.46	1.081	7.30E-57
VWF	ENSG0000	14.41	1.715	2.505	7.16E-57
XRCC1	ENSG0000	13.98	4.955	1.331	6.31E-57
METTL13	ENSG0000	18	7.735	1.121	6.04E-57
NABP2	ENSG0000	19.38	7.77	1.217	5.42E-57
FAM180A	ENSG0000	0.22	3.43	-1.86	4.66E-57
ALG1	ENSG0000	19.83	8.77	1.092	3.96E-57
CCNB2	ENSG0000	4.81	0.27	2.194	3.46E-57
TOMM7	ENSG0000	327.397	128.181	1.346	3.43E-57
TROAP	ENSG0000	6.69	0.325	2.537	3.39E-57
RBM8A	ENSG0000	83.152	34.049	1.264	3.29E-57
PFDN2	ENSG0000	104.959	42.964	1.269	3.23E-57
LAMTOR4	ENSG0000	128.079	55.479	1.192	3.09E-57
E2F1	ENSG0000	5.49	0.28	2.342	2.90E-57
PPIB	ENSG0000	710.208	316.299	1.164	2.58E-57
SND1	ENSG0000	82.521	38.05	1.097	2.44E-57
PRIM2	ENSG0000	4.04	1.06	1.291	2.40E-57
EFTUD2	ENSG0000	32.139	14.38	1.107	2.23E-57
PPP2R1A	ENSG0000	101.412	44.099	1.183	2.18E-57
PCNA	ENSG0000	48.09	13.71	1.739	1.99E-57
CCT7	ENSG0000	105.829	49.213	1.089	1.59E-57
ZCCHC10	ENSG0000	18.15	8.21	1.056	1.41E-57
ALMS1	ENSG0000	5	1.79	1.105	1.28E-57
MAGED2	ENSG0000	63.711	22.045	1.49	1.27E-57
UQCRH	ENSG0000	217.594	83.829	1.366	1.14E-57
RALY	ENSG0000	63.039	26.163	1.237	1.08E-57
EIF2D	ENSG0000	30.789	13.13	1.17	1.04E-57
CENPM	ENSG0000	5.97	0.33	2.39	8.75E-58
ZWINT	ENSG0000	9.69	0.865	2.519	7.03E-58
SLC39A3	ENSG0000	29.3	11.91	1.231	6.69E-58
MFSD5	ENSG0000	14.31	5.98	1.133	6.51E-58
CYSTM1	ENSG0000	72.781	21.57	1.709	6.22E-58
ATP5E	ENSG0000	77.471	33.549	1.184	5.86E-58
RP11-345.	ENSG0000	18.04	5.385	1.576	5.76E-58
DNAJB11	ENSG0000	145.411	63.964	1.172	4.51E-58
CPQ	ENSG0000	47.94	14.525	1.656	4.21E-58
SUPT4H1	ENSG0000	66.192	30.304	1.102	4.01E-58
UFD1L	ENSG0000	57.039	23.485	1.245	3.98E-58
PEA15	ENSG0000	45.819	12.42	1.803	3.81E-58
BOLA1	ENSG0000	26.79	11.355	1.169	3.78E-58
PEX11B	ENSG0000	23.079	10.54	1.061	3.73E-58
PXMP4	ENSG0000	10.38	3.335	1.392	3.71E-58
HAND2-A	ENSG0000	0.12	2.285	-1.552	3.44E-58
C12orf45	ENSG0000	19.5	8.885	1.052	3.14E-58
TK1	ENSG0000	17.25	2.335	2.452	2.99E-58
PMVK	ENSG0000	77.262	35.51	1.1	2.50E-58
RFXANK	ENSG0000	44.099	17.625	1.276	1.95E-58
SMUG1P1	ENSG0000	0	1.055	-1.039	1.88E-58
SNF8	ENSG0000	88.101	39.81	1.127	1.69E-58
NAA20	ENSG0000	71.858	29.014	1.279	1.48E-58
CPSF3	ENSG0000	13.29	5.94	1.042	1.43E-58
EHMT2	ENSG0000	23.36	8.135	1.415	1.27E-58
MRPL14	ENSG0000	79.71	35.33	1.152	9.91E-59
PABPC1	ENSG0000	653.57	181.313	1.844	9.41E-59
C12orf73	ENSG0000	7.32	2.955	1.073	9.38E-59
ARPC1A	ENSG0000	87.772	39.045	1.148	8.56E-59
JMJD4	ENSG0000	14.75	6.215	1.126	8.15E-59

IL3RA	ENSG0000	2.95	0.6	1.304	7.64E-59
TMEM106	ENSG0000	44.05	11.765	1.819	6.54E-59
GPC3	ENSG0000	148.158	0.92	6.28	6.38E-59
RPL8	ENSG0000	1876.722	569.775	1.718	6.14E-59
RPL36A	ENSG0000	883.691	296.337	1.573	5.82E-59
ZNF706	ENSG0000	102.992	38.45	1.398	4.72E-59
ATP5L	ENSG0000	304.183	126.381	1.261	4.57E-59
NSUN5	ENSG0000	17.259	7.33	1.132	4.49E-59
C17orf58	ENSG0000	10.87	4.22	1.185	4.39E-59
GPANK1	ENSG0000	16.02	6.145	1.252	4.25E-59
RBP7	ENSG0000	17.259	2.635	2.329	3.32E-59
GMNN	ENSG0000	31.111	6.19	2.159	2.77E-59
TMEM14C	ENSG0000	101.159	43.904	1.186	2.44E-59
SMYD3	ENSG0000	13.12	3.06	1.798	2.36E-59
ACBD6	ENSG0000	24.71	9.845	1.245	2.08E-59
PSENEN	ENSG0000	80.968	34.785	1.196	1.95E-59
SMIM4	ENSG0000	34.9	9.335	1.796	1.83E-59
LINC0015	ENSG0000	35.26	4.54	2.711	1.71E-59
NUP37	ENSG0000	10.3	3.445	1.346	1.67E-59
NHP2L1	ENSG0000	81.379	35.463	1.176	1.60E-59
NOL7	ENSG0000	38.55	15.44	1.266	1.50E-59
BAX	ENSG0000	78.71	24.245	1.659	1.38E-59
PSMC3	ENSG0000	107.507	52.736	1.014	1.17E-59
ZNRD1	ENSG0000	25.93	10.03	1.288	1.05E-59
MT1M	ENSG0000	1.78	112.018	-5.345	8.93E-60
SNRPD2	ENSG0000	193.165	70.121	1.449	8.88E-60
TBC1D7	ENSG0000	15.92	6.43	1.187	8.27E-60
PSMC4	ENSG0000	71.401	32.394	1.116	7.36E-60
NDUFA13	ENSG0000	306.702	131.338	1.217	6.06E-60
FKBPL	ENSG0000	5.26	2.025	1.049	4.76E-60
FTH1P8	ENSG0000	9.88	1.33	2.223	4.15E-60
NME1	ENSG0000	100.099	29.344	1.736	3.98E-60
COX6C	ENSG0000	582.17	237.502	1.29	3.06E-60
TMEM14B	ENSG0000	75.572	32.725	1.183	2.85E-60
SLC25A39	ENSG0000	125.382	59.779	1.056	2.03E-60
MAN2B1	ENSG0000	55.591	17.704	1.597	1.74E-60
SERF1B	ENSG0000	18.65	6.19	1.45	1.62E-60
TMCO1	ENSG0000	124.94	41.106	1.581	1.55E-60
IFI27L1	ENSG0000	14.55	4.515	1.495	1.39E-60
RNF165	ENSG0000	0.16	1.53	-1.125	1.29E-60
ENSA	ENSG0000	109.949	44.47	1.287	1.18E-60
EXOSC5	ENSG0000	22.461	8.788	1.261	1.15E-60
SNRPD1	ENSG0000	42.22	14.7	1.461	1.13E-60
NAT2	ENSG0000	2.28	27.324	-3.11	1.07E-60
CDK1	ENSG0000	6.32	0.33	2.46	7.38E-61
QARS	ENSG0000	101.447	45.099	1.152	6.28E-61
SLC38A6	ENSG0000	6.79	2.24	1.266	5.55E-61
MRPL17	ENSG0000	33.91	14.065	1.212	5.09E-61
LSM4	ENSG0000	102.757	45.219	1.167	4.90E-61
AP1S1	ENSG0000	45.54	17.135	1.36	4.79E-61
TIMM9	ENSG0000	33.88	14.115	1.206	4.79E-61
COX6A1	ENSG0000	639.1	304.795	1.066	4.56E-61
SHARPIN	ENSG0000	59.272	24.24	1.256	4.45E-61
RPL19	ENSG0000	1450.263	637.353	1.185	4.15E-61
METTL5	ENSG0000	34.82	15.849	1.088	4.15E-61
MPC2	ENSG0000	279.053	110.929	1.323	4.12E-61
MARCO	ENSG0000	0.39	19.3	-3.868	4.03E-61
CKS1B	ENSG0000	71.878	24.695	1.504	3.76E-61

CAPNS1	ENSG0000	328.51	144.006	1.184	3.25E-61
C8orf59	ENSG0000	87.069	34.17	1.324	3.10E-61
TIMM17B	ENSG0000	46.659	22.065	1.047	2.67E-61
SUB1	ENSG0000	184.144	73.124	1.321	2.45E-61
MT1H	ENSG0000	2.16	332.67	-6.722	2.39E-61
EPRS	ENSG0000	37.111	13.56	1.388	2.11E-61
PMF1	ENSG0000	61.158	26.235	1.191	2.11E-61
DAD1	ENSG0000	207.245	96.907	1.089	1.94E-61
CCDC107	ENSG0000	40.179	15.745	1.298	1.88E-61
MANBAL	ENSG0000	32.901	15.21	1.064	1.33E-61
GTF2A2	ENSG0000	61.158	26.395	1.182	1.28E-61
PSMA6	ENSG0000	123.974	57.615	1.092	8.22E-62
TMEM101	ENSG0000	23.46	9.185	1.264	7.51E-62
FEN1	ENSG0000	12.59	2.9	1.801	7.26E-62
PSMG3	ENSG0000	31.519	11.92	1.332	7.20E-62
ADCK2	ENSG0000	14.49	5.585	1.234	6.84E-62
TMEM258	ENSG0000	160.841	73.84	1.113	4.55E-62
UBXN1	ENSG0000	75.378	36.076	1.043	4.46E-62
C1orf43	ENSG0000	182.214	81.021	1.159	4.33E-62
CENPW	ENSG0000	8.1	1.1	2.115	3.78E-62
HDGFRP2	ENSG0000	27.739	12.485	1.092	3.29E-62
NACA	ENSG0000	953.903	425.79	1.162	3.21E-62
RPP21	ENSG0000	44.901	18.17	1.26	2.39E-62
S100A12	ENSG0000	0.19	3.425	-1.895	2.35E-62
CKLF	ENSG0000	29.829	9.115	1.608	2.26E-62
FAU	ENSG0000	796.206	363.206	1.13	2.00E-62
THY1	ENSG0000	20.42	1.995	2.838	1.97E-62
POLR2G	ENSG0000	53.772	23.625	1.153	1.79E-62
GABRD	ENSG0000	1.66	0.06	1.327	1.49E-62
KPNA2	ENSG0000	28.43	6.31	2.009	1.03E-62
CDKN2A	ENSG0000	10.79	0.315	3.164	9.92E-63
PET100	ENSG0000	68.322	29.18	1.2	8.36E-63
ATOX1	ENSG0000	347.29	114.178	1.596	6.63E-63
ZMAT5	ENSG0000	25.2	10.63	1.172	6.35E-63
CDK5	ENSG0000	12.8	4.67	1.283	5.06E-63
CRNKL1	ENSG0000	13.85	5.9	1.106	4.78E-63
CDC20	ENSG0000	9.32	0.31	2.978	3.93E-63
NDUFA2	ENSG0000	139.111	64.451	1.098	2.33E-63
COX7C	ENSG0000	750.183	309.093	1.276	2.01E-63
NDUFA12	ENSG0000	82.681	39.89	1.033	1.74E-63
ECM1	ENSG0000	2.51	17.755	-2.418	1.30E-63
HNRNPCF	ENSG0000	7.96	0.895	2.241	1.00E-63
TMEM9	ENSG0000	78.102	28.145	1.44	6.19E-64
MND1	ENSG0000	2.58	0.17	1.613	4.58E-64
WBSCR22	ENSG0000	56.441	25.895	1.095	4.57E-64
ERGIC3	ENSG0000	192.324	94.039	1.024	4.39E-64
ANKRD39	ENSG0000	14.36	6.27	1.079	4.26E-64
SPNS1	ENSG0000	31.6	13.335	1.185	4.26E-64
PRC1	ENSG0000	10.68	1.12	2.462	3.32E-64
SNRPEP2	ENSG0000	7.97	1.61	1.781	2.82E-64
LSM2	ENSG0000	38.869	13.895	1.42	2.80E-64
LAMTOR2	ENSG0000	162.263	71.28	1.176	2.33E-64
NDUFB9	ENSG0000	377.752	144.862	1.377	2.15E-64
RPL30	ENSG0000	1964.708	654.091	1.585	1.70E-64
GM2A	ENSG0000	16	4.965	1.511	1.23E-64
BSG	ENSG0000	301.329	101.335	1.563	1.20E-64
CNIH4	ENSG0000	32.81	10.014	1.618	1.20E-64
MT2P1	ENSG0000	0	7.11	-3.02	1.10E-64

INS-IGF2	ENSG0000	0	4.03	-2.331	9.78E-65
RUVBL2	ENSG0000	50.822	22.285	1.154	9.46E-65
COMMD5	ENSG0000	24.38	9.165	1.32	5.36E-65
EIF6	ENSG0000	180.305	77.275	1.212	5.07E-65
LMAN2	ENSG0000	226.866	97.544	1.209	4.84E-65
BOLA3	ENSG0000	36.339	13.22	1.393	4.46E-65
COX6B1	ENSG0000	407.285	148.543	1.449	4.41E-65
TNFRSF4	ENSG0000	2.97	0.32	1.589	4.05E-65
TOP2A	ENSG0000	9.54	0.405	2.907	3.35E-65
ATP5G1	ENSG0000	239.967	89.216	1.417	2.90E-65
C14orf166	ENSG0000	107.94	52.815	1.017	2.38E-65
ANAPC11	ENSG0000	192.35	76.061	1.327	1.35E-65
RPL26L1	ENSG0000	43.989	19.929	1.104	1.27E-65
RPL38	ENSG0000	1230.73	457.934	1.424	8.63E-66
GPAA1	ENSG0000	115.511	39.639	1.52	7.85E-66
IK	ENSG0000	56.141	25.39	1.115	5.67E-66
RBM42	ENSG0000	52.83	21.78	1.241	5.56E-66
HEXB	ENSG0000	156.096	62.003	1.318	5.25E-66
SPC24	ENSG0000	5.15	0.33	2.209	5.18E-66
SLC41A3	ENSG0000	18.379	6.215	1.425	5.16E-66
KIAA1429	ENSG0000	32.019	13.155	1.222	4.60E-66
HAMP	ENSG0000	6.75	669.968	-6.436	3.63E-66
ALG8	ENSG0000	45.711	19.841	1.164	3.48E-66
RP11-10G	ENSG0000	2.99	0	1.996	3.34E-66
VPS28	ENSG0000	192.071	76.699	1.313	3.18E-66
CDH13	ENSG0000	3.25	0.32	1.687	2.95E-66
PRDX5	ENSG0000	278.165	128.23	1.111	2.65E-66
RPL37	ENSG0000	1566.576	594.465	1.396	2.08E-66
SRP14	ENSG0000	253.474	113.165	1.156	1.94E-66
MRPL9	ENSG0000	49.261	19.215	1.314	1.94E-66
CXorf36	ENSG0000	2.25	0.32	1.3	1.83E-66
UBD	ENSG0000	334.949	7.468	5.31	1.60E-66
SSR2	ENSG0000	259.267	82.939	1.633	6.01E-67
RNF181	ENSG0000	192.417	83.178	1.2	5.57E-67
C19orf53	ENSG0000	130.861	56.883	1.188	5.48E-67
ATP6V1F	ENSG0000	102.047	36.464	1.46	5.33E-67
UBE2S	ENSG0000	19.39	3.845	2.073	5.29E-67
EMC4	ENSG0000	77.299	36.715	1.054	5.22E-67
CSNK2B	ENSG0000	182.771	79.873	1.184	4.67E-67
GLMP	ENSG0000	123.023	36.071	1.742	4.49E-67
TEX264	ENSG0000	62.651	29.615	1.056	3.68E-67
ILF2	ENSG0000	80.991	27.02	1.549	3.65E-67
LAGE3	ENSG0000	36.59	11.005	1.647	2.33E-67
CCL23	ENSG0000	0.22	2.155	-1.371	1.11E-67
ORMDL2	ENSG0000	48.691	19.455	1.281	9.75E-68
RFWD2	ENSG0000	30.789	12.06	1.283	9.26E-68
NDUFS6	ENSG0000	89.12	35.605	1.3	6.13E-68
RPLP1	ENSG0000	1491.242	599.659	1.313	5.50E-68
UBL7	ENSG0000	29.531	12.445	1.183	4.75E-68
NEU1	ENSG0000	55.299	15.57	1.765	3.02E-68
CLN3	ENSG0000	58.809	19.355	1.555	2.78E-68
DAP3	ENSG0000	95.378	39.351	1.256	2.69E-68
H2AFV	ENSG0000	78.302	31.871	1.271	2.69E-68
NTPCR	ENSG0000	54.161	16.505	1.656	2.67E-68
MAZ	ENSG0000	114.332	43.406	1.377	1.78E-68
UBE2C	ENSG0000	13.24	0.6	3.154	1.56E-68
HIGD2A	ENSG0000	145.804	67.002	1.11	1.48E-68
SF3B4	ENSG0000	35.839	10.79	1.644	1.44E-68

FCN3	ENSG0000	2.32	67.3	-4.363	1.37E-68
BANF1	ENSG0000	152.165	69.61	1.117	1.06E-68
COPS6	ENSG0000	83.707	38.084	1.116	9.69E-69
OST4	ENSG0000	273.879	129.4	1.076	7.70E-69
HIGD1B	ENSG0000	2.2	0.19	1.427	6.54E-69
RABIF	ENSG0000	6.21	2.225	1.161	5.55E-69
UQCRB	ENSG0000	349.269	129.912	1.42	4.17E-69
MRPS16	ENSG0000	81.452	35.44	1.178	4.09E-69
ROMO1	ENSG0000	249.154	86.933	1.508	3.79E-69
BRMS1	ENSG0000	30.751	11.85	1.305	3.54E-69
TOMM20	ENSG0000	65.871	21.944	1.543	3.13E-69
CHMP2A	ENSG0000	166.352	74.886	1.141	3.08E-69
DDX41	ENSG0000	59.511	26.05	1.162	1.64E-69
NDUFA7	ENSG0000	96.837	42.579	1.167	9.36E-70
HAX1	ENSG0000	107.328	46.864	1.178	6.03E-70
ATP5H	ENSG0000	378.119	168.709	1.16	5.68E-70
EIF3K	ENSG0000	205.429	85.005	1.263	5.18E-70
RBM34	ENSG0000	44.711	15.919	1.434	4.00E-70
CTSA	ENSG0000	181.974	53.563	1.746	1.71E-70
SAC3D1	ENSG0000	13.78	4.47	1.434	1.33E-70
UBL5	ENSG0000	409.522	183.329	1.155	1.28E-70
SNRPB	ENSG0000	126.473	35.895	1.789	1.23E-70
NHP2	ENSG0000	99.332	37.635	1.377	6.68E-71
GRN	ENSG0000	187.857	59.719	1.637	6.44E-71
PSMB5	ENSG0000	103.421	47.67	1.101	6.32E-71
CDKN2AIF	ENSG0000	16.91	6.3	1.295	5.74E-71
NEDD8	ENSG0000	160.385	77.409	1.041	5.01E-71
CNPY2	ENSG0000	165.673	75.695	1.12	4.93E-71
COX4I2	ENSG0000	3.1	0.33	1.624	4.93E-71
NENF	ENSG0000	81.311	29.561	1.429	4.92E-71
PDZD11	ENSG0000	30.37	11.065	1.379	4.14E-71
UBE2SP2	ENSG0000	3.54	0.085	2.065	3.79E-71
DPY30	ENSG0000	58.651	25.86	1.151	3.29E-71
NDUFA1	ENSG0000	183.101	75.371	1.269	2.97E-71
PIGC	ENSG0000	22.07	6.605	1.601	1.92E-71
CCNB1	ENSG0000	14.8	0.9	3.056	1.24E-71
NR2C2AP	ENSG0000	14.33	4.33	1.524	1.09E-71
SCNM1	ENSG0000	33.21	10.825	1.533	9.85E-72
PRKCSH	ENSG0000	198.376	98.155	1.008	8.08E-72
AP3B1	ENSG0000	16.49	6.325	1.256	6.45E-72
FTH1P7	ENSG0000	25.5	2.38	2.971	2.02E-72
BCAP31	ENSG0000	202.657	70.416	1.512	1.98E-72
ATP6AP1	ENSG0000	65.639	24.015	1.414	1.58E-72
TOMM6	ENSG0000	230.017	86.413	1.402	1.25E-72
PIGT	ENSG0000	65.05	22.78	1.474	1.20E-72
H3F3A	ENSG0000	397.496	139.034	1.509	9.56E-73
NELFE	ENSG0000	58.618	14.75	1.92	4.92E-73
FLAD1	ENSG0000	34.291	12.85	1.349	4.75E-73
AATF	ENSG0000	29.171	10.64	1.374	3.60E-73
ATP5J2	ENSG0000	584.111	238.425	1.289	2.71E-73
BLOC1S1	ENSG0000	213.441	94.87	1.161	2.56E-73
PFDN6	ENSG0000	118.569	41.236	1.501	1.97E-73
FAM65C	ENSG0000	0.33	7.74	-2.716	8.69E-74
UQCC2	ENSG0000	98.373	32.169	1.583	7.45E-74
CDKN3	ENSG0000	10.96	0.45	3.044	4.14E-74
ATP5G2	ENSG0000	428.751	189.499	1.174	3.40E-74
RPS28P7	ENSG0000	722.222	182.518	1.979	1.96E-74
MRPS21	ENSG0000	63.999	22.47	1.47	1.96E-74

CUTA	ENSG0000	288.894	108.548	1.404	1.34E-74
BOLA2B	ENSG0000	84.67	20.26	2.011	1.17E-74
UBE2T	ENSG0000	8.81	0.695	2.533	9.85E-75
ABHD12	ENSG0000	35.671	12.07	1.488	3.60E-75
CRHBP	ENSG0000	0.56	16.829	-3.515	3.43E-75
TRMT112	ENSG0000	215.358	83.786	1.352	3.43E-75
MRPL24	ENSG0000	161.735	54.922	1.541	2.07E-75
RPN2	ENSG0000	201.829	74.294	1.43	1.68E-75
STMN1	ENSG0000	56.668	6.28	2.986	1.65E-75
PPIA	ENSG0000	1363.405	530.68	1.36	1.65E-75
BCO2	ENSG0000	2.42	31.444	-3.246	8.54E-76
COX6B1P4	ENSG0000	0	1.055	-1.039	8.51E-76
UFC1	ENSG0000	97.497	33.145	1.528	7.85E-76
PRCC	ENSG0000	41.28	15.355	1.37	7.36E-76
RNASEH2A	ENSG0000	16.151	2.735	2.199	4.56E-77
NUDT1	ENSG0000	15.59	2.595	2.206	2.77E-77
CXCL14	ENSG0000	0.19	14.215	-3.676	7.94E-78
VPS72	ENSG0000	28.489	8.295	1.666	7.94E-78
RP11-40C	ENSG0000	389.91	0	8.611	3.30E-78
NTF3	ENSG0000	0.23	2.445	-1.486	1.37E-78
PPIAP22	ENSG0000	165.466	34.006	2.25	9.13E-79
PSMD4	ENSG0000	242.576	78.784	1.61	2.53E-79
POLR2K	ENSG0000	55.819	19.015	1.505	8.99E-80
PTTG1	ENSG0000	20.589	0.87	3.529	5.59E-80
SNRPC	ENSG0000	118.742	39.239	1.573	4.96E-80
DYNLRB1	ENSG0000	145.139	51.949	1.465	1.38E-80
MDK	ENSG0000	132.339	3.805	4.794	7.57E-81
SHFM1	ENSG0000	305.07	97.133	1.641	1.47E-81
PIGU	ENSG0000	16.75	4.875	1.595	1.06E-81
ADRA1A	ENSG0000	0.75	16.38	-3.312	2.62E-82
MTX1	ENSG0000	58.838	20.895	1.45	1.55E-82
SCAMP3	ENSG0000	85.793	25.355	1.72	5.81E-83
SNRPE	ENSG0000	137.548	38.494	1.811	5.11E-83
DRAP1	ENSG0000	101.391	38.361	1.379	4.29E-83
PSMB4	ENSG0000	347.073	134.526	1.361	3.11E-83
MEA1	ENSG0000	62.691	22.585	1.433	2.41E-84
FTH1P20	ENSG0000	13.07	1.48	2.504	1.81E-84
PRPF6	ENSG0000	40.029	14.02	1.45	6.06E-85
NSMCE2	ENSG0000	20.92	5.565	1.739	4.91E-85
LMNA	ENSG0000	175.52	45.859	1.913	4.12E-85
CCT3	ENSG0000	185.734	51.09	1.842	3.78E-87
LINC0109	ENSG0000	0.55	23.3	-3.971	3.78E-87
BMPER	ENSG0000	0.06	1.515	-1.246	1.97E-87
COA6	ENSG0000	53.471	13.715	1.888	2.18E-88
APOA1BP	ENSG0000	120.684	38.634	1.618	9.18E-90
VIPR1	ENSG0000	0.56	11.848	-3.042	4.67E-92
KRTCAP2	ENSG0000	342.651	102.454	1.732	1.10E-93
CD34	ENSG0000	14.17	1.625	2.531	8.03E-94
ANGPTL6	ENSG0000	1.67	11.6	-2.238	1.35E-96
COLEC10	ENSG0000	0.39	12.865	-3.318	1.21E-96
COMMD4	ENSG0000	110.17	35.546	1.605	1.73E-97
ADAMTS1	ENSG0000	2.32	14.475	-2.221	1.49E-97
CLEC1B	ENSG0000	0.03	8.658	-3.229	1.07E-97
DBH	ENSG0000	0.56	10.785	-2.917	5.04E-99
PTH1R	ENSG0000	1.32	23.575	-3.405	1.44E-102
FCN2	ENSG0000	0.28	30.658	-4.628	2.54E-103
GBA	ENSG0000	73.48	12.61	2.452	2.46E-103
STAB2	ENSG0000	0.08	3.505	-2.06	1.98E-103

CLEC4G	ENSG0000	0.12	23.634	-4.459	5.34E-114
GDF2	ENSG0000	0	4.84	-2.546	1.40E-115
PLVAP	ENSG0000	43.03	2.464	3.668	7.99E-127
CLEC4M	ENSG0000	0.02	18.949	-4.29	1.36E-146
