# Supplementary material for: Mitochondrial-Related Transcriptome Feature Correlates with Prognosis, Vascular Invasion, Tumor Microenvironment, and Treatment Response in Hepatocellular Carcinoma
Source: Oxid Med Cell Longev. 2022 Apr 30;2022:1592905. doi: 10.1155/2022/1592905 (PMC9078845; doi:10.1155/2022/1592905)
Supplement: Supplementary Materials — Supplementary Figure 1: the Kaplan-Meier curves between high- and low-risk patients, in VI group (A) or none-VI group (B). Supplementary Figure 2: the ROC curves of each involved NMRG and prognosis_score for overall survival (OS) at 1 (A), 3 (B), and 5 years (C). Supplementary Figure 3:the differentially expressed genes between high- and low-risk groups. Supplementary Figure 4: the differentially expressed genes between nonresponder and responder groups. Supplementary Figure 5: the intersections of significantly upregulated genes (A) and downregulated genes (B) between low-risk patients and responder patients. The upregulated pathways potentially targeted by sorafenib via HALLMARK (C) and KEGG (D) enrichment analysis. The downregulated pathways potentially targeted by sorafenib via HALLMARK (E) and KEGG (F) enrichment analysis. Supplementary Figure 6: the evaluation of chemodrug treatment response between non-VI, micro-VI, and macro-VI groups. Supplementary Figure 7: The evaluation of chemodrug treatment response between high- and low-risk patients in the non-VI group. Supplementary Figure 8: the evaluation of chemodrug treatment response between high- and low-risk patients in the micro-VI group. Supplementary Figure 9: the evaluation of chemodrug treatment response between high- and low-risk patients in the macro-VI group. Supplementary Table 1: differentially expressed genes between tumor and normal tissues. Supplementary Table 2: coefficients of each NMRG involved in the NMRG signature. Supplementary Table 3: Top 20 high prevalence of altered genes in the high-risk group. Supplementary Table 4: Top 20 high prevalence of altered genes in the low-risk group. Supplementary Table: 5. the prevalence of a total of 61 genes was significantly different between high- and low-risk groups. [file 1592905.f1.zip › supplementary table 1B.pdf]

Supplementary Table 1B. Differentially upregulated genes in tumor tissues.

Gene Syml	Gene ID	Median (T	Median (N	Log2(Fold	adjp
FKBP9	ENSG0000	17.64	8.315	1.001	5.14E-27
APIP	ENSG0000	18.439	8.705	1.002	1.29E-36
TMED9	ENSG0000	183.304	91.034	1.002	6.92E-43
RP11-138	ENSG0000	198.074	98.4	1.002	6.64E-21
RPL15	ENSG0000	755.139	377.046	1.002	1.90E-44
RRP15	ENSG0000	4.32	1.655	1.003	5.88E-26
ENY2	ENSG0000	127.009	62.88	1.003	1.36E-46
ADIPOR1	ENSG0000	66.791	32.8	1.004	1.88E-43
BTF3	ENSG0000	338.66	168.376	1.004	8.92E-46
CORO1B	ENSG0000	47.55	23.209	1.004	1.49E-42
HSPB11	ENSG0000	21.91	10.425	1.004	5.79E-49
SEC11C	ENSG0000	180.33	89.39	1.004	4.71E-25
FASN	ENSG0000	217.896	108.112	1.004	2.11E-08
ARHGEF3	ENSG0000	1.81	0.4	1.005	1.83E-36
CAPRIN1	ENSG0000	35.17	17.019	1.005	9.85E-33
LAPTM5	ENSG0000	28.489	13.69	1.005	3.00E-11
MAGED1	ENSG0000	64.44	31.6	1.005	1.20E-12
RPS3AP6	ENSG0000	12.82	5.885	1.005	1.09E-27
TPR	ENSG0000	20	9.455	1.006	7.29E-31
AIMP2	ENSG0000	27.651	13.26	1.007	1.33E-32
CTSD	ENSG0000	769.138	382.084	1.007	1.02E-31
HCFC1R1	ENSG0000	44.622	21.699	1.007	2.88E-40
RP11-977	ENSG0000	1.01	0	1.007	1.70E-11
GEMIN6	ENSG0000	11.22	5.08	1.007	7.82E-55
RUSC1	ENSG0000	10.76	4.85	1.007	2.44E-33
TRBC2	ENSG0000	11.13	5.035	1.007	3.92E-09
UGT1A10	ENSG0000	1.21	0.1	1.007	4.13E-17
ARF3	ENSG0000	30.759	14.79	1.008	1.65E-30
DPP3	ENSG0000	21.58	10.23	1.008	6.32E-37
PRKCSH	ENSG0000	198.376	98.155	1.008	8.08E-72
NUDT14	ENSG0000	19.94	9.41	1.008	7.68E-22
CSTB	ENSG0000	62.012	30.3	1.009	3.64E-26
EFCAB11	ENSG0000	4.05	1.51	1.009	6.94E-49
NDUFS8	ENSG0000	131.06	64.625	1.009	9.08E-47
RAB4A	ENSG0000	37.219	17.995	1.009	1.56E-41
CCL18	ENSG0000	2.04	0.51	1.01	1.72E-11
COPZ1	ENSG0000	81.882	40.161	1.01	1.86E-49
RPS13	ENSG0000	649.415	322.018	1.01	6.76E-48
HELLS	ENSG0000	1.83	0.405	1.01	1.27E-29
RPL23A	ENSG0000	690.213	342.33	1.01	5.01E-38
TAF9	ENSG0000	33.171	15.97	1.01	1.93E-35
RPP40	ENSG0000	7.11	3.025	1.011	2.19E-36
RPS6	ENSG0000	1117.066	553.925	1.011	2.75E-32
FAM103A	ENSG0000	15.7	7.285	1.011	9.60E-51
FIBP	ENSG0000	36.801	17.76	1.011	8.92E-57
NDUFA4	ENSG0000	120.508	59.309	1.011	1.15E-48
RP11-475	ENSG0000	15.42	7.145	1.011	2.34E-28
TMEM164	ENSG0000	2.59	0.78	1.012	5.00E-28
ARF1	ENSG0000	269.025	132.775	1.013	3.51E-42
BPGM	ENSG0000	8.15	3.535	1.013	3.91E-40
FTL	ENSG0000	11550.76	5723.571	1.013	5.51E-19
SNX15	ENSG0000	9.93	4.415	1.013	8.06E-43
SKA3	ENSG0000	1.16	0.07	1.013	1.35E-39
SOX18	ENSG0000	4.61	1.78	1.013	5.70E-29
PMS2P1	ENSG0000	5.08	2.01	1.014	4.84E-48
SYNGR1	ENSG0000	6.5	2.715	1.014	5.92E-08
PSMC3	ENSG0000	107.507	52.736	1.014	1.17E-59

BCAS4	ENSG0000	1.85	0.41	1.015	2.00E-31
EEF1G	ENSG0000	721.421	356.435	1.015	1.20E-44
IMMP2L	ENSG0000	17.52	8.165	1.015	8.77E-32
ACP6	ENSG0000	18.6	8.69	1.016	6.14E-32
ATP6V1H	ENSG0000	23.62	11.17	1.016	1.12E-44
BAG6	ENSG0000	179.805	88.398	1.016	1.81E-55
C1orf35	ENSG0000	12.7	5.775	1.016	1.91E-48
ATP6V1D	ENSG0000	38.409	18.475	1.017	3.53E-39
C14orf166	ENSG0000	107.94	52.815	1.017	2.38E-65
CENPK	ENSG0000	1.51	0.24	1.017	2.37E-30
PDAP1	ENSG0000	72.978	35.554	1.017	1.75E-36
VBP1	ENSG0000	18.29	8.53	1.017	3.50E-39
DHFRP1	ENSG0000	2.31	0.635	1.018	4.52E-10
TIMM10	ENSG0000	60.48	29.36	1.018	4.07E-50
TBCC	ENSG0000	11.02	4.93	1.019	3.26E-45
FUCA2	ENSG0000	34.951	16.73	1.02	1.28E-29
SAMM50	ENSG0000	30.89	14.73	1.02	2.94E-36
PSMB10	ENSG0000	59.112	28.645	1.02	1.14E-29
POLR2J2	ENSG0000	5.5	2.205	1.02	1.64E-14
AC093673	ENSG0000	5.26	2.085	1.021	1.12E-24
ATIC	ENSG0000	66.201	32.119	1.021	1.34E-34
DTNBP1	ENSG0000	8.48	3.67	1.021	1.58E-32
RHOA	ENSG0000	154.396	75.585	1.021	1.12E-41
SLC29A1	ENSG0000	46.571	22.445	1.021	4.68E-19
LSM7	ENSG0000	71.848	34.91	1.021	2.95E-46
GGPS1	ENSG0000	12.93	5.865	1.021	4.77E-50
CH17-340	ENSG0000	9.38	4.11	1.022	8.62E-42
EMP1	ENSG0000	7.72	3.295	1.022	2.12E-06
RP3-417G	ENSG0000	2.94	0.94	1.022	5.27E-29
CLN6	ENSG0000	19.69	9.18	1.023	2.95E-51
TMEM97	ENSG0000	38.62	18.5	1.023	7.48E-16
RFC3	ENSG0000	3.4	1.165	1.023	5.19E-32
UQCR10	ENSG0000	135.532	66.201	1.023	7.94E-53
TSEN15	ENSG0000	11.54	5.17	1.023	1.19E-41
ALKBH2	ENSG0000	25.259	11.915	1.024	4.09E-37
ERGIC3	ENSG0000	192.324	94.039	1.024	4.39E-64
PDIA6	ENSG0000	159.155	77.753	1.024	3.64E-27
THOC7	ENSG0000	41.271	19.789	1.024	1.66E-54
MORN2	ENSG0000	12.46	5.615	1.025	9.02E-40
GRK6	ENSG0000	10.62	4.71	1.025	1.04E-47
CD320	ENSG0000	20.33	9.475	1.026	1.42E-40
PHPT1	ENSG0000	99.787	48.509	1.026	2.25E-41
NIPAL2	ENSG0000	5.82	2.35	1.026	1.57E-22
SYNGR2	ENSG0000	83.169	40.321	1.026	2.46E-38
SNRPF	ENSG0000	27.971	13.225	1.026	4.38E-44
PTRH2	ENSG0000	27.851	13.168	1.026	6.31E-49
SNAPIN	ENSG0000	32.479	15.445	1.026	1.25E-54
LINC0046	ENSG0000	10.37	4.585	1.026	1.15E-42
TOR3A	ENSG0000	17.179	7.93	1.026	1.02E-27
AC016747	ENSG0000	4.32	1.61	1.027	9.53E-37
C10orf35	ENSG0000	3.84	1.375	1.027	1.80E-18
PDCD5	ENSG0000	85.241	41.33	1.027	2.56E-49
UCHL5	ENSG0000	15.85	7.27	1.027	2.61E-26
DCTN2	ENSG0000	55.341	26.62	1.028	6.03E-34
ZMAT2	ENSG0000	48.509	23.279	1.028	1.50E-47
PPIC	ENSG0000	23.64	11.08	1.028	2.60E-31
XRCC6	ENSG0000	110.598	53.725	1.028	4.22E-48
PNMA6A	ENSG0000	9.82	4.305	1.028	2.33E-07

ARRDC2	ENSG0000	15.5	7.085	1.029	1.11E-14
C11orf98	ENSG0000	63.592	30.65	1.029	1.59E-48
DHX9	ENSG0000	41.55	19.855	1.029	8.48E-40
TEAD2	ENSG0000	7.46	3.145	1.029	1.47E-13
SWAP70	ENSG0000	5.09	1.985	1.029	7.00E-23
TUBB2A	ENSG0000	60.17	28.979	1.029	8.62E-16
TIPRL	ENSG0000	13.95	6.325	1.029	5.64E-42
SEMA3G	ENSG0000	1.93	0.435	1.03	6.60E-27
SMOX	ENSG0000	4.76	1.82	1.03	1.40E-09
RPL12	ENSG0000	922.752	451.379	1.03	9.95E-36
EZH2	ENSG0000	3.65	1.275	1.031	7.56E-30
RP11-35N	ENSG0000	3.71	1.305	1.031	1.27E-21
ZNF581	ENSG0000	9.69	4.23	1.031	1.32E-44
C1QTNF5	ENSG0000	11.43	5.08	1.032	2.00E-19
DOLK	ENSG0000	10.08	4.42	1.032	8.50E-57
LRP11	ENSG0000	7.82	3.315	1.032	1.44E-31
PTGFRN	ENSG0000	10.95	4.845	1.032	4.96E-18
RPL4	ENSG0000	1141.566	557.933	1.032	2.02E-40
MARCKSL	ENSG0000	29.07	13.709	1.032	3.51E-18
C15orf40	ENSG0000	15.5	7.065	1.033	4.08E-49
CALM3	ENSG0000	104.213	50.399	1.033	1.35E-34
DUSP23	ENSG0000	131.497	63.76	1.033	3.93E-33
LAMP2	ENSG0000	125.756	60.959	1.033	4.88E-20
GPKOW	ENSG0000	14.82	6.73	1.033	2.64E-56
NDUFA12	ENSG0000	82.681	39.89	1.033	1.74E-63
RRP9	ENSG0000	12.34	5.515	1.034	3.72E-45
C1QA	ENSG0000	81.492	39.245	1.035	1.34E-09
COA3	ENSG0000	130.119	62.989	1.035	3.77E-45
DCK	ENSG0000	3.95	1.415	1.035	8.38E-27
DTD1	ENSG0000	10.72	4.72	1.035	4.28E-20
RPL5	ENSG0000	560.899	273.187	1.035	3.44E-40
TPP1	ENSG0000	67.568	32.47	1.035	2.90E-23
BAMBI	ENSG0000	13.81	6.22	1.036	1.44E-05
COCH	ENSG0000	1.42	0.18	1.036	1.03E-21
RPL37A	ENSG0000	2021.064	985.047	1.036	9.48E-37
GRAMD1A	ENSG0000	18.69	8.595	1.037	7.23E-16
RBBP4	ENSG0000	30.969	14.58	1.037	1.25E-12
THEM6	ENSG0000	35.921	16.975	1.038	1.03E-27
NFKBIL1	ENSG0000	18.87	8.68	1.038	1.09E-51
RP11-369	ENSG0000	1.73	0.33	1.038	8.97E-05
CTNNA1	ENSG0000	88.812	42.721	1.039	1.10E-43
FST	ENSG0000	51.929	24.758	1.039	7.56E-05
MRPL15	ENSG0000	50.699	24.155	1.039	2.07E-40
SECTM1	ENSG0000	5.34	2.085	1.039	4.35E-19
FN3K	ENSG0000	54.059	25.804	1.039	7.59E-25
HRAS	ENSG0000	32.271	15.19	1.039	3.00E-48
RPL31	ENSG0000	1529.028	743.015	1.04	2.85E-40
KLHDC3	ENSG0000	61.12	29.215	1.04	5.23E-54
UBA52	ENSG0000	852.821	414.275	1.04	8.44E-57
ABCB6	ENSG0000	39.371	18.615	1.041	8.28E-19
ATP6V0B	ENSG0000	142.339	68.669	1.041	8.52E-40
CHMP4B	ENSG0000	57.181	27.285	1.041	1.47E-42
PES1	ENSG0000	40.87	19.35	1.041	2.02E-45
NEDD8	ENSG0000	160.385	77.409	1.041	5.01E-71
STAP2	ENSG0000	66.68	31.891	1.041	2.07E-21
MRPL21	ENSG0000	61.23	29.245	1.041	5.43E-45
CPSF3	ENSG0000	13.29	5.94	1.042	1.43E-58
PDCD2L	ENSG0000	6.35	2.57	1.042	1.95E-47

RPS24	ENSG0000	1646.514	799.358	1.042	1.33E-38
VPS33A	ENSG0000	7.39	3.075	1.042	3.20E-40
UBE2A	ENSG0000	25.029	11.63	1.043	4.00E-40
MAP2	ENSG0000	3.05	0.965	1.043	8.47E-17
NDUFA8	ENSG0000	57.432	27.36	1.043	3.50E-55
HAUS1	ENSG0000	8.63	3.675	1.043	4.37E-37
UBXN1	ENSG0000	75.378	36.076	1.043	4.46E-62
ISG20	ENSG0000	19.96	9.17	1.043	7.63E-16
NDUFB6	ENSG0000	61.601	29.35	1.044	8.17E-52
TRIM16L	ENSG0000	7.1	2.925	1.045	6.52E-17
CDC123	ENSG0000	33.16	16.075	1.045	6.77E-45
BEX2	ENSG0000	2.48	0.685	1.046	1.26E-11
TSNAX	ENSG0000	14.6	6.555	1.046	5.62E-45
NUDCD2	ENSG0000	15.3	6.895	1.046	8.99E-32
POLR2L	ENSG0000	155.448	74.78	1.046	6.25E-51
YBEY	ENSG0000	17.97	8.19	1.046	9.18E-35
DEPTOR	ENSG0000	9.3	3.985	1.047	4.12E-28
FBLN1	ENSG0000	8.62	3.655	1.047	4.88E-07
IFT52	ENSG0000	7.39	3.06	1.047	2.12E-37
TIMM17B	ENSG0000	46.659	22.065	1.047	2.67E-61
MIS18A	ENSG0000	4.36	1.595	1.047	1.36E-38
C17orf89	ENSG0000	74.47	35.49	1.048	1.48E-45
TXNDC12	ENSG0000	31.04	14.5	1.048	4.23E-55
ARV1	ENSG0000	17.67	8.02	1.049	3.26E-45
SMS	ENSG0000	25.701	11.905	1.049	2.98E-37
SSR1	ENSG0000	43.609	20.554	1.049	4.69E-23
TPPP3	ENSG0000	4.09	1.46	1.049	3.36E-13
NSA2	ENSG0000	56.629	26.857	1.049	7.30E-45
FKBPL	ENSG0000	5.26	2.025	1.049	4.76E-60
RP11-680	ENSG0000	3.77	1.305	1.049	1.13E-16
AC092171	ENSG0000	2.23	0.56	1.05	2.10E-36
DERL1	ENSG0000	56.191	26.628	1.05	5.19E-37
PYGO2	ENSG0000	15.31	6.875	1.05	1.25E-47
NUDT2	ENSG0000	20.569	9.42	1.05	1.35E-43
SLC25A6	ENSG0000	110.063	52.654	1.05	5.00E-32
TATDN3	ENSG0000	7.83	3.265	1.05	3.12E-45
AGBL5	ENSG0000	8.21	3.445	1.051	2.57E-39
PLOD1	ENSG0000	76.388	36.346	1.051	2.95E-32
TRAIP	ENSG0000	1.87	0.385	1.051	1.16E-40
C12orf45	ENSG0000	19.5	8.885	1.052	3.14E-58
CRTAP	ENSG0000	47.31	22.299	1.052	1.27E-22
CYC1	ENSG0000	157.324	75.334	1.052	5.84E-48
RIPK2	ENSG0000	6.29	2.515	1.052	6.24E-25
NCAPG2	ENSG0000	2.93	0.895	1.052	3.71E-28
TMEM183	ENSG0000	27.02	12.51	1.052	5.56E-52
NSMCE1	ENSG0000	51.61	24.375	1.052	2.32E-56
WBP5	ENSG0000	20.89	9.55	1.053	1.10E-06
FAM127B	ENSG0000	33.109	15.435	1.053	8.59E-12
EMC4	ENSG0000	77.299	36.715	1.054	5.22E-67
MRPL47	ENSG0000	30.3	14.075	1.054	8.16E-49
RPL23AP8	ENSG0000	9.59	4.1	1.054	1.44E-41
CCDC28B	ENSG0000	3.8	1.31	1.055	2.88E-26
SLC25A39	ENSG0000	125.382	59.779	1.056	2.03E-60
PRR11	ENSG0000	1.37	0.14	1.056	2.78E-35
TMEM209	ENSG0000	5.27	2.015	1.056	2.42E-34
ZCCHC10	ENSG0000	18.15	8.21	1.056	1.41E-57
IGSF8	ENSG0000	46.529	21.859	1.056	1.58E-26
TEX264	ENSG0000	62.651	29.615	1.056	3.68E-67

SPON2	ENSG0000	94.851	45.069	1.057	1.97E-17
CANX	ENSG0000	179.83	85.843	1.058	6.23E-29
CH17-13I2	ENSG0000	1.3	0.105	1.058	2.82E-10
NDUFAF2	ENSG0000	25.06	11.515	1.058	6.33E-43
GNB2L1	ENSG0000	891.937	427.994	1.058	4.11E-47
ZSCAN16-	ENSG0000	8.45	3.54	1.058	4.19E-41
BROX	ENSG0000	10.95	4.735	1.059	1.51E-45
C9orf16	ENSG0000	50.801	23.865	1.059	2.63E-38
SCPEP1	ENSG0000	25.849	11.89	1.059	5.18E-11
KIF23	ENSG0000	1.48	0.19	1.059	7.83E-31
PTMA	ENSG0000	508.498	243.57	1.059	1.61E-41
ATP6V0E1	ENSG0000	207.691	99.105	1.06	2.92E-53
PSMD2	ENSG0000	81.48	38.55	1.06	4.01E-29
DCDC2	ENSG0000	2.38	0.62	1.061	3.39E-11
PEX11B	ENSG0000	23.079	10.54	1.061	3.73E-58
RPN1	ENSG0000	160.362	76.362	1.061	9.65E-46
FTH1P23	ENSG0000	2.4	0.63	1.061	2.57E-49
RP11-111	ENSG0000	4.52	1.645	1.061	4.45E-37
PTGES3	ENSG0000	137.758	65.443	1.062	8.02E-42
HGH1	ENSG0000	22.409	10.215	1.062	1.99E-38
DPCD	ENSG0000	7.67	3.15	1.063	3.19E-37
YIPF3	ENSG0000	100.002	47.349	1.063	3.77E-55
GAS5	ENSG0000	169.388	80.573	1.063	1.93E-27
CCNE1	ENSG0000	1.55	0.22	1.064	5.34E-30
DHRS7	ENSG0000	80.309	37.9	1.064	5.29E-22
MANBAL	ENSG0000	32.901	15.21	1.064	1.33E-61
RPS19	ENSG0000	1703.043	813.895	1.064	4.40E-40
SEC61G	ENSG0000	167.195	79.423	1.064	8.75E-38
BPNT1	ENSG0000	13.67	6.01	1.065	1.24E-31
CCDC64	ENSG0000	4.25	1.51	1.065	1.30E-13
CTSH	ENSG0000	163.46	77.63	1.065	5.46E-19
RPL6	ENSG0000	779.064	371.739	1.065	3.44E-53
NSL1	ENSG0000	13.84	6.095	1.065	1.05E-34
SLC6A8	ENSG0000	3.29	1.05	1.065	4.62E-12
SHC1	ENSG0000	58.189	27.29	1.065	5.31E-27
RPL41P2	ENSG0000	37.139	17.225	1.065	2.90E-18
CHCHD6	ENSG0000	8.61	3.59	1.066	5.59E-48
COX6A1	ENSG0000	639.1	304.795	1.066	4.56E-61
DBNDD2	ENSG0000	8.35	3.465	1.066	1.79E-24
PDE6D	ENSG0000	11.73	5.08	1.066	9.55E-46
RUVBL1	ENSG0000	17.349	7.765	1.066	1.18E-37
FXYD5	ENSG0000	29.699	13.652	1.067	1.43E-14
S100A11	ENSG0000	77.321	36.376	1.067	7.15E-13
RPL22L1	ENSG0000	61.469	28.814	1.067	3.95E-20
SOX12	ENSG0000	4.26	1.51	1.067	6.01E-28
TBCAP1	ENSG0000	1.64	0.26	1.067	8.15E-27
ALDH1A1	ENSG0000	458.284	217.994	1.068	2.39E-10
ATRAID	ENSG0000	74.109	34.835	1.068	2.28E-55
YIF1B	ENSG0000	47.891	22.32	1.068	4.91E-35
GLB1	ENSG0000	33.079	15.252	1.068	3.66E-39
AGPAT1	ENSG0000	46.821	21.794	1.069	7.90E-49
S100P	ENSG0000	6.05	2.36	1.069	1.30E-08
KCTD17	ENSG0000	4.51	1.625	1.07	2.73E-18
ABCC6P2	ENSG0000	8.95	3.735	1.071	3.34E-29
PSME2	ENSG0000	253.755	120.229	1.071	5.98E-45
MKKS	ENSG0000	22.899	10.375	1.071	4.69E-37
PARPBP	ENSG0000	1.5	0.19	1.071	4.70E-39
C12orf73	ENSG0000	7.32	2.955	1.073	9.38E-59

CENPL	ENSG0000	1.82	0.34	1.073	8.02E-41
RP11-128	ENSG0000	1.61	0.24	1.074	4.26E-37
TRIP13	ENSG0000	1.55	0.21	1.075	3.15E-33
RAD51AP1	ENSG0000	1.72	0.29	1.076	1.24E-31
SRP9	ENSG0000	168.685	79.5	1.076	2.63E-44
INHBB	ENSG0000	10.18	4.304	1.076	1.55E-09
PPP1R11	ENSG0000	42.579	19.67	1.076	8.00E-46
OST4	ENSG0000	273.879	129.4	1.076	7.70E-69
MRPS17	ENSG0000	16.48	7.29	1.076	5.73E-45
C6orf89	ENSG0000	20.281	9.085	1.077	1.24E-38
CTNNBL1	ENSG0000	45.379	20.99	1.077	2.39E-33
MAGOHB	ENSG0000	17.95	7.98	1.077	7.20E-53
PDGFRB	ENSG0000	10.1	4.26	1.077	1.10E-16
RAC1	ENSG0000	116.994	54.913	1.077	8.53E-51
NDUFS4	ENSG0000	82.081	38.374	1.077	4.28E-45
COX7A2	ENSG0000	317.364	149.805	1.078	3.75E-45
MYL6	ENSG0000	1006.338	476.208	1.078	8.54E-53
SPTSSA	ENSG0000	24.609	11.13	1.078	1.28E-21
OLFML2A	ENSG0000	1.48	0.175	1.078	1.57E-41
ADSL	ENSG0000	31.91	14.58	1.079	1.69E-44
ANKRD39	ENSG0000	14.36	6.27	1.079	4.26E-64
DDOST	ENSG0000	104.22	48.816	1.079	1.42E-35
SPCS1	ENSG0000	167.846	78.948	1.079	7.46E-50
COX8A	ENSG0000	396.56	187.065	1.08	2.35E-54
TOMM22	ENSG0000	38.84	17.85	1.08	5.91E-53
UBALD2	ENSG0000	27.02	12.254	1.08	6.24E-33
CGREF1	ENSG0000	9.8	4.105	1.081	7.12E-12
DRG1	ENSG0000	40.159	18.46	1.081	7.30E-57
IRF6	ENSG0000	21.24	9.51	1.081	3.04E-07
HMGN1	ENSG0000	69.42	32.281	1.081	3.30E-36
CD36	ENSG0000	16.32	7.178	1.083	7.68E-10
CANT1	ENSG0000	14.81	6.46	1.084	2.58E-39
CUEDC1	ENSG0000	7.88	3.19	1.084	6.86E-26
DNAJC19F	ENSG0000	2.02	0.425	1.084	5.55E-20
AC016292	ENSG0000	2.15	0.485	1.085	2.75E-23
APOE	ENSG0000	5880.405	2771.689	1.085	8.27E-14
ARSE	ENSG0000	49.9	23	1.085	9.66E-07
LINC0160	ENSG0000	8.22	3.345	1.085	3.98E-34
TSPAN15	ENSG0000	5.6	2.11	1.086	1.23E-19
RPS23	ENSG0000	1376.605	648.044	1.086	5.30E-45
SNRPD3	ENSG0000	99.401	46.269	1.087	1.58E-47
PSEN2	ENSG0000	14.1	6.11	1.087	1.08E-31
C6orf1	ENSG0000	20.87	9.29	1.088	3.18E-43
METTL5	ENSG0000	34.82	15.849	1.088	4.15E-61
BRIX1	ENSG0000	13	5.58	1.089	2.97E-39
CCT7	ENSG0000	105.829	49.213	1.089	1.59E-57
CLTA	ENSG0000	122.402	57.029	1.089	1.99E-50
DAD1	ENSG0000	207.245	96.907	1.089	1.94E-61
ZCRB1	ENSG0000	33.481	15.205	1.089	1.97E-48
LINC0066	ENSG0000	2.16	0.485	1.089	3.09E-18
NCAPH2	ENSG0000	23.71	10.6	1.091	1.53E-48
TCEB2	ENSG0000	295.517	138.164	1.091	2.37E-51
PDIA3	ENSG0000	370.671	173.452	1.091	2.37E-44
ZNF585A	ENSG0000	5.72	2.155	1.091	7.45E-34
ALG1	ENSG0000	19.83	8.77	1.092	3.96E-57
ELOVL1	ENSG0000	37.6	17.105	1.092	1.59E-39
RPLP0	ENSG0000	1398.049	655.112	1.092	1.85E-44
PSMA6	ENSG0000	123.974	57.615	1.092	8.22E-62

HDGFRP2	ENSG0000	27.739	12.485	1.092	3.29E-62
SLC43A2	ENSG0000	4.48	1.57	1.092	9.52E-20
UBE2Q2P6	ENSG0000	655.975	307.128	1.092	1.39E-15
RPS16	ENSG0000	1328.515	622.443	1.093	5.50E-47
SMARCA4	ENSG0000	29.769	13.425	1.093	2.20E-50
RAC3	ENSG0000	14.47	6.25	1.093	3.17E-22
SH3BGRL3	ENSG0000	70.629	32.565	1.094	7.00E-26
RP11-386	ENSG0000	69.622	32.095	1.094	7.95E-18
BZW2	ENSG0000	15.3	6.63	1.095	3.77E-34
CD151	ENSG0000	152.704	70.965	1.095	6.23E-29
CSTF2	ENSG0000	4.47	1.56	1.095	1.37E-34
WBSCR22	ENSG0000	56.441	25.895	1.095	4.57E-64
TNFRSF21	ENSG0000	5.45	2.02	1.095	5.04E-13
POC1A	ENSG0000	4.81	1.72	1.095	4.36E-44
CETN2	ENSG0000	20.93	9.26	1.096	3.54E-44
STAT1	ENSG0000	38.62	17.54	1.096	1.35E-16
TBCE	ENSG0000	16.95	7.4	1.096	1.50E-51
SKA2	ENSG0000	14.76	6.375	1.096	2.07E-41
TSPAN4	ENSG0000	40.52	18.425	1.096	2.62E-21
ARPC3	ENSG0000	174.634	81.089	1.097	8.44E-55
PIK3IP1	ENSG0000	7	2.74	1.097	1.30E-17
TRIM28	ENSG0000	143.419	66.495	1.097	4.58E-45
SND1	ENSG0000	82.521	38.05	1.097	2.44E-57
NDUFA2	ENSG0000	139.111	64.451	1.098	2.33E-63
HNRNPA3	ENSG0000	124.077	57.412	1.098	4.79E-50
RP11-556	ENSG0000	1.14	0	1.098	1.21E-22
DAXX	ENSG0000	23.62	10.495	1.099	5.71E-40
SAMD1	ENSG0000	17.89	7.82	1.099	1.55E-40
HTATSF1	ENSG0000	19.28	8.46	1.1	9.73E-37
P3H4	ENSG0000	3.91	1.29	1.1	3.48E-29
SUSD4	ENSG0000	3.92	1.295	1.1	1.23E-08
PMVK	ENSG0000	77.262	35.51	1.1	2.50E-58
PTENP1	ENSG0000	1.98	0.39	1.1	5.57E-32
EIF3M	ENSG0000	133.963	61.922	1.101	2.64E-48
PSMB5	ENSG0000	103.421	47.67	1.101	6.32E-71
RPL23	ENSG0000	944.625	439.386	1.102	1.17E-46
PAPSS1	ENSG0000	6.5	2.495	1.102	1.33E-25
UTP14A	ENSG0000	7.05	2.75	1.102	2.94E-44
NAP1L1	ENSG0000	87.462	40.215	1.102	1.73E-30
SUPT4H1	ENSG0000	66.192	30.304	1.102	4.01E-58
TRAF2	ENSG0000	10.5	4.355	1.103	2.86E-43
STYXL1	ENSG0000	31.679	14.21	1.103	1.62E-37
RPL29	ENSG0000	1049.145	487.919	1.103	1.66E-49
S100A4	ENSG0000	29.25	13.083	1.103	9.94E-12
PTMAP5	ENSG0000	2.79	0.765	1.103	9.23E-26
AIDA	ENSG0000	24.519	10.875	1.104	1.27E-46
CAV1	ENSG0000	11.49	4.81	1.104	7.21E-21
RPL26L1	ENSG0000	43.989	19.929	1.104	1.27E-65
MAP1S	ENSG0000	14.04	5.995	1.104	2.83E-44
TM4SF5	ENSG0000	200.31	92.641	1.104	3.90E-09
LCMT1	ENSG0000	12.62	5.335	1.104	5.15E-53
RP11-49K	ENSG0000	3.59	1.135	1.104	3.04E-08
ALMS1	ENSG0000	5	1.79	1.105	1.28E-57
CLEC14A	ENSG0000	7.72	3.055	1.105	1.10E-24
ACTN4	ENSG0000	102.749	47.2	1.106	1.82E-31
CRNKL1	ENSG0000	13.85	5.9	1.106	4.78E-63
MIF4GD	ENSG0000	16.58	7.17	1.106	5.98E-40
EFTUD2	ENSG0000	32.139	14.38	1.107	2.23E-57

TAX1BP3	ENSG0000	26.981	11.99	1.107	2.37E-17
CBX8	ENSG0000	4.54	1.57	1.108	1.07E-43
GTF2IRD1	ENSG0000	7.07	2.745	1.108	1.25E-33
TMEM14A	ENSG0000	50.499	22.89	1.108	1.53E-28
SSR3	ENSG0000	89.059	40.774	1.108	4.02E-38
OSGIN1	ENSG0000	89.492	40.993	1.108	1.48E-08
SLAMF8	ENSG0000	2.05	0.415	1.108	2.94E-20
LRPAP1	ENSG0000	95.259	43.646	1.108	2.91E-40
GPSM1	ENSG0000	3.67	1.165	1.109	1.63E-18
ALG3	ENSG0000	57.289	26.005	1.11	3.84E-51
PLAU	ENSG0000	2.82	0.77	1.11	1.07E-23
SMO	ENSG0000	16.5	7.11	1.11	2.25E-16
HIGD2A	ENSG0000	145.804	67.002	1.11	1.48E-68
TAP1	ENSG0000	22.079	9.69	1.11	1.05E-22
SLC35F6	ENSG0000	16.66	7.18	1.11	4.73E-44
COX20	ENSG0000	40.969	18.425	1.111	2.80E-44
PRDX5	ENSG0000	278.165	128.23	1.111	2.65E-66
LY96	ENSG0000	8.45	3.375	1.111	7.04E-14
TBC1D16	ENSG0000	5.07	1.81	1.111	6.32E-26
SUMO2P1	ENSG0000	1.16	0	1.111	1.18E-06
DNPH1	ENSG0000	125.6	57.579	1.112	3.92E-46
HMGN2	ENSG0000	225.736	103.877	1.112	3.38E-42
SUCO	ENSG0000	6.03	2.25	1.113	2.38E-32
TBCB	ENSG0000	58.351	26.444	1.113	4.50E-42
MYL12B	ENSG0000	310.295	142.938	1.113	5.47E-42
TMEM258	ENSG0000	160.841	73.84	1.113	4.55E-62
WDYHV1	ENSG0000	8.96	3.605	1.113	2.23E-42
POLR3C	ENSG0000	10.38	4.26	1.113	5.62E-43
FAM20B	ENSG0000	8.97	3.605	1.114	6.50E-49
RPS11	ENSG0000	1591.31	734.489	1.114	3.62E-54
IK	ENSG0000	56.141	25.39	1.115	5.67E-66
GJA1	ENSG0000	4.23	1.415	1.115	3.08E-18
BUD31	ENSG0000	64.382	29.16	1.116	6.44E-52
COPS6	ENSG0000	83.707	38.084	1.116	9.69E-69
PSMC4	ENSG0000	71.401	32.394	1.116	7.36E-60
BANF1	ENSG0000	152.165	69.61	1.117	1.06E-68
NDRG3	ENSG0000	7.09	2.73	1.117	1.16E-41
RBM3	ENSG0000	79.539	36.115	1.118	1.99E-23
ICAM2	ENSG0000	12.49	5.214	1.118	1.55E-25
RPSA	ENSG0000	680.38	312.941	1.118	2.42E-39
TRIM16	ENSG0000	6.27	2.35	1.118	7.03E-22
ADM2	ENSG0000	2.8	0.75	1.119	3.37E-20
ALDH3A1	ENSG0000	2.17	0.46	1.119	7.82E-12
DYNC1H1	ENSG0000	65.922	29.81	1.119	3.19E-53
TMEM132	ENSG0000	2.42	0.575	1.119	1.31E-17
NEDD4L	ENSG0000	25.941	11.405	1.119	1.58E-29
CNPY2	ENSG0000	165.673	75.695	1.12	4.93E-71
AKIP1	ENSG0000	12.78	5.335	1.121	7.69E-50
DPM3	ENSG0000	133.61	60.894	1.121	1.08E-40
METTL13	ENSG0000	18	7.735	1.121	6.04E-57
NUTF2	ENSG0000	55.861	25.134	1.121	1.43E-51
RPL14P1	ENSG0000	9.55	3.85	1.121	3.84E-40
KDELR3	ENSG0000	9.84	3.98	1.122	1.90E-13
PIK3R2	ENSG0000	16.35	6.97	1.122	1.80E-33
GNS	ENSG0000	30.759	13.59	1.122	3.96E-38
RPL13AP5	ENSG0000	68.802	31.064	1.122	1.31E-37
UBE2M	ENSG0000	40.19	17.905	1.123	1.37E-56
WBSCR27	ENSG0000	3.28	0.965	1.123	2.17E-25

CTD-2540	ENSG0000	125.4	57.014	1.124	2.20E-18
FKBP1A	ENSG0000	88.56	40.05	1.125	5.76E-48
OPTN	ENSG0000	50.569	22.641	1.125	2.32E-36
PA2G4	ENSG0000	52.57	23.56	1.125	6.70E-46
ZNF775	ENSG0000	8.49	3.35	1.125	1.01E-34
AC007318	ENSG0000	4.63	1.58	1.126	1.13E-24
CD248	ENSG0000	3.79	1.195	1.126	1.40E-25
JMJD4	ENSG0000	14.75	6.215	1.126	8.15E-59
RPL24	ENSG0000	884.733	404.767	1.126	5.84E-52
C15orf39	ENSG0000	4.46	1.5	1.127	5.00E-32
SNF8	ENSG0000	88.101	39.81	1.127	1.69E-58
TSTD1	ENSG0000	79.181	35.715	1.127	6.37E-20
NOMO1	ENSG0000	31.299	13.774	1.128	4.10E-47
SNRPB2	ENSG0000	28.699	12.59	1.128	1.39E-52
PYCR2	ENSG0000	40.649	18.06	1.128	1.26E-48
RPL28	ENSG0000	1238.089	565.741	1.129	5.32E-43
PHB	ENSG0000	120.25	54.451	1.129	2.57E-52
CMSS1	ENSG0000	13.65	5.695	1.13	5.50E-47
6-Sep	ENSG0000	22.06	9.535	1.13	9.56E-18
FAU	ENSG0000	796.206	363.206	1.13	2.00E-62
PRKDC	ENSG0000	22.389	9.685	1.13	4.97E-16
IGF2BP2	ENSG0000	2.68	0.68	1.131	2.89E-15
AFP	ENSG0000	11.46	4.685	1.132	2.19E-11
CEP131	ENSG0000	5.98	2.185	1.132	3.42E-49
CTB-25B1	ENSG0000	9.2	3.654	1.132	2.22E-49
NSUN5	ENSG0000	17.259	7.33	1.132	4.49E-59
ACP5	ENSG0000	22.77	9.84	1.133	2.02E-08
PSMD14	ENSG0000	27.46	11.975	1.133	1.07E-37
RPL36	ENSG0000	739.803	336.869	1.133	1.54E-46
MFSD5	ENSG0000	14.31	5.98	1.133	6.51E-58
EXOC4	ENSG0000	11.34	5.17	1.133	6.07E-48
SPA17	ENSG0000	2.84	0.75	1.134	2.96E-32
SPATS2	ENSG0000	5.64	2.025	1.134	7.78E-35
NREP	ENSG0000	17.25	7.315	1.134	3.45E-19
RP11-465	ENSG0000	4.77	1.63	1.134	3.17E-22
EIF3CL	ENSG0000	33.16	14.54	1.136	9.35E-38
EMC2	ENSG0000	31.99	14.01	1.136	6.04E-41
PSME1	ENSG0000	221.797	100.384	1.136	5.05E-56
COPG2	ENSG0000	6	2.18	1.138	8.90E-34
MGP	ENSG0000	34.7	15.225	1.138	2.51E-06
PHLDA3	ENSG0000	6.77	2.53	1.138	4.69E-10
ALDOA	ENSG0000	289.595	130.929	1.139	1.02E-26
EMCN	ENSG0000	3.89	1.22	1.139	2.32E-25
AC012146	ENSG0000	5.99	2.17	1.141	8.25E-30
CHMP2A	ENSG0000	166.352	74.886	1.141	3.08E-69
CKAP2	ENSG0000	3.09	0.855	1.141	2.09E-33
CAPN2	ENSG0000	26.33	11.385	1.142	1.95E-25
HNRNPA1	ENSG0000	7.86	3.015	1.142	1.06E-33
CHCHD3	ENSG0000	24.45	10.515	1.144	7.26E-34
EEF1D	ENSG0000	621.107	280.43	1.144	2.20E-43
TGFBI	ENSG0000	114.213	51.15	1.144	5.76E-22
HLA-DRB	ENSG0000	14.2	5.88	1.144	1.50E-05
C19orf48	ENSG0000	26.67	11.515	1.145	7.86E-38
CDKN2B	ENSG0000	2.03	0.37	1.145	2.24E-30
LASP1	ENSG0000	64.408	28.585	1.145	4.22E-38
AP000349	ENSG0000	1.28	0.03	1.146	1.78E-25
C20orf27	ENSG0000	22.11	9.443	1.146	4.31E-49
CASC5	ENSG0000	3.48	1.025	1.146	7.01E-22

DLL4	ENSG0000	3.79	1.165	1.146	6.49E-35
PLXND1	ENSG0000	36.579	15.975	1.147	2.23E-29
ARPC1A	ENSG0000	87.772	39.045	1.148	8.56E-59
DDAH2	ENSG0000	45.041	19.78	1.148	3.31E-31
TAX1BP1	ENSG0000	42.499	18.625	1.148	4.00E-42
TOMM40	ENSG0000	36.99	16.13	1.149	8.57E-43
RP11-579	ENSG0000	1.95	0.33	1.149	5.42E-28
C6orf47	ENSG0000	10.63	4.24	1.15	4.29E-35
HAUS4	ENSG0000	21.44	9.115	1.15	4.55E-25
UBE2Q2	ENSG0000	5.68	2.01	1.15	1.86E-21
TAP2	ENSG0000	17.289	7.24	1.15	1.36E-20
CCT4	ENSG0000	57.519	25.355	1.151	2.01E-54
DPY30	ENSG0000	58.651	25.86	1.151	3.29E-71
HCST	ENSG0000	12.42	5.039	1.152	2.51E-17
QARS	ENSG0000	101.447	45.099	1.152	6.28E-61
MRPL14	ENSG0000	79.71	35.33	1.152	9.91E-59
F5	ENSG0000	206.614	92.446	1.152	8.08E-13
SLC12A5	ENSG0000	2.37	0.515	1.153	6.52E-24
POLR2G	ENSG0000	53.772	23.625	1.153	1.79E-62
RNU4-2	ENSG0000	13.67	5.598	1.153	5.34E-06
HEXA	ENSG0000	76.68	33.93	1.153	2.55E-44
SPAG4	ENSG0000	6.14	2.21	1.154	1.70E-20
RUVBL2	ENSG0000	50.822	22.285	1.154	9.46E-65
PSMC1P1	ENSG0000	13.25	5.405	1.154	3.47E-19
B3GNT3	ENSG0000	2.33	0.495	1.155	1.36E-10
RAD51	ENSG0000	1.75	0.235	1.155	6.48E-39
UBL5	ENSG0000	409.522	183.329	1.155	1.28E-70
HLA-C	ENSG0000	499.658	223.828	1.155	7.73E-16
GGCT	ENSG0000	31.26	13.475	1.156	8.64E-56
MMP9	ENSG0000	3.01	0.8	1.156	1.18E-15
PDCL3	ENSG0000	11.29	4.515	1.156	1.68E-53
SRP14	ENSG0000	253.474	113.165	1.156	1.94E-66
ANXA11	ENSG0000	86.99	38.454	1.157	3.81E-43
ATP6V1C1	ENSG0000	16.8	6.985	1.157	5.23E-30
C1orf106	ENSG0000	1.81	0.26	1.157	1.60E-19
GNPAT	ENSG0000	27.72	11.88	1.157	9.80E-51
CD3D	ENSG0000	4.12	1.295	1.158	2.24E-16
RAP2A	ENSG0000	5.38	1.86	1.158	1.82E-31
C1orf43	ENSG0000	182.214	81.021	1.159	4.33E-62
CASK	ENSG0000	8.9	3.435	1.159	1.41E-26
ATP5H	ENSG0000	378.119	168.709	1.16	5.68E-70
GLUL	ENSG0000	323.024	144.037	1.16	5.79E-11
ZP3	ENSG0000	2.71	0.66	1.16	1.18E-29
RP11-452	ENSG0000	7.38	2.75	1.16	3.59E-14
BLOC1S1	ENSG0000	213.441	94.87	1.161	2.56E-73
PTK2	ENSG0000	22.721	9.61	1.161	3.37E-38
RABIF	ENSG0000	6.21	2.225	1.161	5.55E-69
DDX41	ENSG0000	59.511	26.05	1.162	1.64E-69
TRAPPC2L	ENSG0000	54.651	23.875	1.162	1.09E-53
NACA	ENSG0000	953.903	425.79	1.162	3.21E-62
RPL6P27	ENSG0000	12.27	4.93	1.162	9.28E-36
GOLPH3L	ENSG0000	9.31	3.605	1.163	6.65E-38
FAM49B	ENSG0000	17.579	7.295	1.163	1.50E-28
FAM195B	ENSG0000	68.721	30.14	1.163	3.78E-40
HLA-DQA	ENSG0000	2.19	0.425	1.163	1.62E-08
ALG8	ENSG0000	45.711	19.841	1.164	3.48E-66
BUB1B	ENSG0000	1.6	0.16	1.164	1.37E-34
SKA1	ENSG0000	1.33	0.04	1.164	7.50E-40

PPIB	ENSG0000	710.208	316.299	1.164	2.58E-57
ANO10	ENSG0000	10.95	4.33	1.165	1.20E-42
RPL14	ENSG0000	706.917	314.714	1.165	1.55E-49
PITPNA-A	ENSG0000	8.52	3.245	1.165	5.67E-37
PSMB3	ENSG0000	225.627	100.068	1.165	7.70E-57
LSM4	ENSG0000	102.757	45.219	1.167	4.90E-61
RP11-480	ENSG0000	3.39	0.955	1.167	3.16E-25
NDUFA7	ENSG0000	96.837	42.579	1.167	9.36E-70
CYTH2	ENSG0000	23.18	9.76	1.168	2.22E-48
HM13	ENSG0000	160.162	70.709	1.168	1.15E-45
MAPRE1	ENSG0000	14.98	6.11	1.168	5.40E-28
GSN	ENSG0000	85.882	37.655	1.168	6.32E-15
BOLA1	ENSG0000	26.79	11.355	1.169	3.78E-58
TFPT	ENSG0000	11.74	4.665	1.169	2.46E-54
EIF2D	ENSG0000	30.789	13.13	1.17	1.04E-57
MTHFD1L	ENSG0000	4.12	1.275	1.17	1.52E-27
SLC35B2	ENSG0000	32.739	13.99	1.17	4.34E-43
SOX9-AS1	ENSG0000	2.87	0.72	1.17	9.13E-19
C17orf49	ENSG0000	55.241	23.97	1.171	1.71E-42
C1orf54	ENSG0000	7.23	2.655	1.171	3.55E-24
C2orf54	ENSG0000	6.44	2.305	1.171	4.81E-06
DLGAP5	ENSG0000	1.41	0.07	1.171	3.76E-38
TGM3	ENSG0000	1.68	0.19	1.171	3.54E-22
DNAJB11	ENSG0000	145.411	63.964	1.172	4.51E-58
ZMAT5	ENSG0000	25.2	10.63	1.172	6.35E-63
AKR1C1	ENSG0000	481.068	212.636	1.174	9.13E-08
ATP5G2	ENSG0000	428.751	189.499	1.174	3.40E-74
C8orf76	ENSG0000	19.81	8.225	1.174	8.54E-53
KIFAP3	ENSG0000	9.63	3.71	1.174	1.70E-37
SRC	ENSG0000	8.85	3.365	1.174	1.05E-12
C7orf50	ENSG0000	47.619	20.535	1.175	3.79E-50
SMYD2	ENSG0000	30.199	12.82	1.175	1.71E-33
BCL2L12	ENSG0000	19.401	8.03	1.176	9.33E-50
NHP2L1	ENSG0000	81.379	35.463	1.176	1.60E-59
LAMTOR2	ENSG0000	162.263	71.28	1.176	2.33E-64
VRK1	ENSG0000	4.99	1.65	1.177	7.51E-52
PPM1G	ENSG0000	39.769	17.025	1.177	6.71E-53
RP4-706A	ENSG0000	22.42	9.355	1.177	2.60E-30
CSE1L	ENSG0000	21.24	8.83	1.178	3.31E-55
EHD4	ENSG0000	9.79	3.77	1.178	6.04E-22
HAX1	ENSG0000	107.328	46.864	1.178	6.03E-70
MRPS16	ENSG0000	81.452	35.44	1.178	4.09E-69
HMGN4	ENSG0000	13.39	5.36	1.178	1.88E-18
RASSF3	ENSG0000	6.27	2.21	1.179	1.48E-21
DCTPP1	ENSG0000	22.201	9.24	1.18	5.91E-53
TTC9	ENSG0000	2.24	0.43	1.18	1.69E-22
GINS1	ENSG0000	1.72	0.2	1.181	2.40E-37
RTP4	ENSG0000	5.18	1.725	1.181	3.68E-22
PSMD10	ENSG0000	20.941	8.67	1.182	5.29E-39
GTF2A2	ENSG0000	61.158	26.395	1.182	1.28E-61
TALDO1	ENSG0000	149.955	65.534	1.182	8.91E-51
PPP2R1A	ENSG0000	101.412	44.099	1.183	2.18E-57
NECAB3	ENSG0000	21.341	8.84	1.183	1.03E-53
TMEM14B	ENSG0000	75.572	32.725	1.183	2.85E-60
UBL7	ENSG0000	29.531	12.445	1.183	4.75E-68
SCARA3	ENSG0000	3.69	1.065	1.183	2.01E-20
RPS7	ENSG0000	895.903	394.121	1.183	1.42E-54
MYEOV2	ENSG0000	94.693	41.148	1.183	1.77E-53

RPL39	ENSG0000	1924.809	846.93	1.183	1.93E-44
ATP5E	ENSG0000	77.471	33.549	1.184	5.86E-58
CAPNS1	ENSG0000	328.51	144.006	1.184	3.25E-61
CSNK2B	ENSG0000	182.771	79.873	1.184	4.67E-67
RPS10	ENSG0000	973.069	427.846	1.184	1.26E-42
C17orf58	ENSG0000	10.87	4.22	1.185	4.39E-59
DSN1	ENSG0000	7.32	2.66	1.185	1.72E-48
IGBP1	ENSG0000	20.49	8.45	1.185	2.05E-47
RPL19	ENSG0000	1450.263	637.353	1.185	4.15E-61
H2AFY	ENSG0000	56.539	24.299	1.185	1.46E-44
SPNS1	ENSG0000	31.6	13.335	1.185	4.26E-64
RPL35A	ENSG0000	803.914	353.091	1.185	3.04E-53
TOPORS-A	ENSG0000	9.65	3.685	1.185	1.22E-48
B3GNT5	ENSG0000	2.3	0.45	1.186	1.39E-19
TMEM14C	ENSG0000	101.159	43.904	1.186	2.44E-59
PPIH	ENSG0000	16.36	6.63	1.186	8.15E-51
RTN2	ENSG0000	5.1	1.68	1.187	3.26E-19
PARP1	ENSG0000	36.99	15.685	1.187	1.12E-34
TBC1D7	ENSG0000	15.92	6.43	1.187	8.27E-60
RPS14	ENSG0000	955.822	419.331	1.187	1.83E-54
FOLR2	ENSG0000	10.76	4.165	1.187	2.82E-09
STIP1	ENSG0000	52.57	22.525	1.187	2.85E-33
C19orf53	ENSG0000	130.861	56.883	1.188	5.48E-67
IFI35	ENSG0000	35.901	15.2	1.188	3.78E-32
IMPDH2	ENSG0000	67.662	29.13	1.188	2.28E-39
ISG15	ENSG0000	71.55	30.819	1.189	9.07E-13
TARBP1	ENSG0000	14.1	5.62	1.19	5.14E-36
CMTM7	ENSG0000	8.1	3.55	1.19	1.06E-15
CHEK1	ENSG0000	2.38	0.48	1.191	1.77E-38
PMF1	ENSG0000	61.158	26.235	1.191	2.11E-61
TUBA1A	ENSG0000	15.45	6.205	1.191	8.89E-18
COX7B	ENSG0000	168.51	73.211	1.192	4.09E-56
RAD51C	ENSG0000	13.68	5.425	1.192	5.70E-52
LAMTOR4	ENSG0000	128.079	55.479	1.192	3.09E-57
THOC3	ENSG0000	22.43	9.25	1.193	6.88E-48
RPS27	ENSG0000	3269.149	1428.86	1.193	1.73E-47
LYRM4	ENSG0000	16	6.435	1.193	1.35E-52
CYB5R1	ENSG0000	47.698	20.285	1.194	2.90E-52
DNASE2	ENSG0000	32.07	13.445	1.195	5.48E-27
NDUFC2	ENSG0000	323.853	140.872	1.195	2.15E-56
MRPL55	ENSG0000	113.739	49.114	1.195	2.74E-56
ATP6V1E1	ENSG0000	67.568	28.939	1.196	3.07E-53
SERPINB1	ENSG0000	26.379	10.953	1.196	4.83E-24
NCSTN	ENSG0000	62.552	26.736	1.196	1.96E-34
PSENEN	ENSG0000	80.968	34.785	1.196	1.95E-59
HIST2H4A	ENSG0000	8.54	3.165	1.196	6.77E-22
RPL27	ENSG0000	1270.164	553.58	1.197	2.01E-54
UGGT1	ENSG0000	14.17	5.615	1.197	1.62E-43
CCDC80	ENSG0000	4.3	1.31	1.198	5.83E-10
RRAGD	ENSG0000	8.76	3.255	1.198	1.36E-28
LL22NC03	ENSG0000	1.96	0.29	1.198	1.07E-26
PRR34-AS	ENSG0000	7.78	3.39	1.198	3.51E-24
ITGB4	ENSG0000	5.13	1.67	1.199	9.14E-26
SERPINH1	ENSG0000	37.121	15.61	1.199	5.16E-20
RAD21	ENSG0000	33.499	14.025	1.199	1.17E-29
CBX1	ENSG0000	14.33	5.675	1.2	2.02E-35
CCDC167	ENSG0000	49.179	20.844	1.2	8.38E-48
RNF187	ENSG0000	55.749	23.695	1.2	3.40E-56

RNF181	ENSG0000	192.417	83.178	1.2	5.57E-67
PET100	ENSG0000	68.322	29.18	1.2	8.36E-63
CCDC86	ENSG0000	12.32	4.795	1.201	1.91E-38
FLVCR1-A	ENSG0000	2.93	0.71	1.201	1.73E-32
ITGB3BP	ENSG0000	8.56	3.155	1.202	6.97E-46
STT3A	ENSG0000	76.882	32.829	1.203	1.44E-42
GPATCH4	ENSG0000	18.181	7.33	1.203	2.74E-34
CCHCR1	ENSG0000	11.72	4.52	1.204	6.93E-41
RPL27A	ENSG0000	1599.05	693.57	1.204	5.94E-51
CENPN	ENSG0000	6.1	2.08	1.205	4.90E-38
LOXL2	ENSG0000	3.68	1.03	1.205	1.84E-25
NOMO2	ENSG0000	83.609	35.69	1.205	5.96E-48
B9D1	ENSG0000	10.26	3.88	1.206	3.33E-37
TIMM9	ENSG0000	33.88	14.115	1.206	4.79E-61
FANCD2	ENSG0000	2.68	0.595	1.206	8.55E-35
ZNF544	ENSG0000	9.53	3.565	1.206	2.66E-35
NPC2	ENSG0000	121.456	52.019	1.208	5.03E-45
RP11-73N	ENSG0000	1.31	0	1.208	1.45E-21
LMAN2	ENSG0000	226.866	97.544	1.209	4.84E-65
DBN1	ENSG0000	4.76	1.49	1.21	5.55E-22
KNSTRN	ENSG0000	7.78	2.795	1.21	1.60E-38
COPA	ENSG0000	47.009	19.744	1.211	6.92E-30
SRD5A3	ENSG0000	15.24	6.015	1.211	1.74E-50
SNHG6	ENSG0000	110.921	47.349	1.211	2.29E-36
EIF6	ENSG0000	180.305	77.275	1.212	5.07E-65
MRPL17	ENSG0000	33.91	14.065	1.212	5.09E-61
FHIT	ENSG0000	11.09	4.22	1.212	4.78E-39
TUBB	ENSG0000	207.015	88.763	1.212	3.10E-32
GGTA1P	ENSG0000	3.88	1.105	1.213	7.57E-28
KIAA0196	ENSG0000	15.47	6.095	1.215	2.18E-52
FABP4	ENSG0000	7.47	2.645	1.216	2.62E-10
PUF60	ENSG0000	112.259	47.77	1.216	3.24E-54
MAPK3	ENSG0000	24.54	9.99	1.217	2.16E-53
FKBP11	ENSG0000	70.438	29.725	1.217	5.20E-22
NABP2	ENSG0000	19.38	7.77	1.217	5.42E-57
FAM3B	ENSG0000	2.72	0.6	1.217	4.04E-11
NDUFA13	ENSG0000	306.702	131.338	1.217	6.06E-60
C16orf59	ENSG0000	2.19	0.37	1.219	1.35E-39
H1F0	ENSG0000	142.438	60.62	1.219	4.46E-28
SMARCE1	ENSG0000	49.05	20.464	1.221	1.03E-43
BTG3	ENSG0000	8.55	3.095	1.222	8.12E-29
WDR76	ENSG0000	2.02	0.295	1.222	2.31E-41
KIAA1429	ENSG0000	32.019	13.155	1.222	4.60E-66
C1orf198	ENSG0000	12.46	4.765	1.223	7.64E-27
C8orf33	ENSG0000	18.379	7.305	1.223	4.89E-48
TP53BP2	ENSG0000	12.89	4.945	1.224	1.25E-22
GJA5	ENSG0000	2.24	0.385	1.226	1.02E-31
AC009065	ENSG0000	1.34	0	1.227	9.02E-31
PARVB	ENSG0000	16.31	6.394	1.227	3.72E-13
CLDN7	ENSG0000	35.24	14.474	1.228	2.16E-08
TMEM38B	ENSG0000	8.39	3.005	1.229	1.28E-35
SLC39A7	ENSG0000	107.157	45.144	1.229	2.79E-43
MAF1	ENSG0000	56.469	23.51	1.229	1.43E-55
TRIM52-A	ENSG0000	9.2	3.35	1.229	7.07E-50
MRPL13	ENSG0000	47.431	19.64	1.23	8.48E-57
SLC39A3	ENSG0000	29.3	11.91	1.231	6.69E-58
SERPIND1	ENSG0000	344.389	146.027	1.232	4.45E-04
SERPINI1	ENSG0000	3.56	0.94	1.233	6.41E-31

TDRKH	ENSG0000	4.09	1.165	1.233	1.56E-35
ADCK2	ENSG0000	14.49	5.585	1.234	6.84E-62
APLN	ENSG0000	1.59	0.1	1.235	3.06E-34
GGH	ENSG0000	121.852	51.186	1.235	1.99E-17
COL1A2	ENSG0000	29.91	12.124	1.236	1.68E-09
PIR	ENSG0000	23.02	9.2	1.236	1.28E-27
HTATIP2	ENSG0000	77.831	32.464	1.236	1.48E-26
FBXO32	ENSG0000	4.23	1.22	1.236	3.93E-29
CCT5	ENSG0000	82.509	34.429	1.237	3.35E-42
RALY	ENSG0000	63.039	26.163	1.237	1.08E-57
FAM111B	ENSG0000	1.57	0.09	1.237	5.95E-33
CTC-246B	ENSG0000	5.76	1.865	1.238	2.29E-25
DUXAP8	ENSG0000	1.89	0.225	1.238	1.59E-27
POLA2	ENSG0000	7.17	2.465	1.238	1.69E-50
ASNA1	ENSG0000	43.761	17.964	1.239	1.73E-56
EEF1A1P5	ENSG0000	63.341	26.253	1.239	2.14E-14
RPL24P4	ENSG0000	9.02	3.245	1.239	7.20E-36
HLA-DRB1	ENSG0000	66.431	27.555	1.24	4.58E-10
RBM42	ENSG0000	52.83	21.78	1.241	5.56E-66
TWF2	ENSG0000	30.83	12.465	1.241	1.10E-36
CFL1	ENSG0000	437.093	184.272	1.242	1.62E-54
FBLN7	ENSG0000	6.2	2.045	1.242	1.89E-27
GARS	ENSG0000	59.231	24.455	1.243	2.68E-45
CCT6A	ENSG0000	83.169	34.536	1.244	1.14E-46
EIF3D	ENSG0000	84.829	35.248	1.244	9.48E-55
NPM1P27	ENSG0000	5.95	1.935	1.244	5.04E-35
ACBD6	ENSG0000	24.71	9.845	1.245	2.08E-59
UFD1L	ENSG0000	57.039	23.485	1.245	3.98E-58
CDK4	ENSG0000	47.33	19.38	1.246	1.22E-41
LIG1	ENSG0000	16.18	6.24	1.247	6.01E-44
MPZL1	ENSG0000	21.41	8.445	1.247	1.17E-30
DLGAP1-A	ENSG0000	7.94	2.765	1.248	3.17E-30
RNASET2	ENSG0000	42.86	17.46	1.248	9.02E-33
AKR1C2	ENSG0000	299.123	125.148	1.25	8.98E-05
HOMER3	ENSG0000	7.3	2.49	1.25	2.80E-23
AC005943	ENSG0000	1.38	0	1.251	8.76E-11
SOX4	ENSG0000	3.07	0.71	1.251	5.22E-16
NGFRAP1	ENSG0000	82.928	34.27	1.251	1.48E-06
GPANK1	ENSG0000	16.02	6.145	1.252	4.25E-59
TPGS2	ENSG0000	20.93	8.2	1.253	2.54E-30
CD2BP2	ENSG0000	24.97	9.89	1.254	4.12E-56
HACD3	ENSG0000	47.261	19.23	1.254	2.71E-30
S100A6	ENSG0000	69.07	28.38	1.254	2.49E-12
ECSCR	ENSG0000	6.8	2.27	1.254	1.22E-32
EFNA4	ENSG0000	8.27	2.885	1.255	2.00E-46
FAM50A	ENSG0000	61.922	25.359	1.255	1.46E-43
RPL32	ENSG0000	1338.312	560.083	1.255	1.99E-50
ACLY	ENSG0000	20.91	8.174	1.256	5.83E-28
AP3B1	ENSG0000	16.49	6.325	1.256	6.45E-72
DAP3	ENSG0000	95.378	39.351	1.256	2.69E-68
SHARPIN	ENSG0000	59.272	24.24	1.256	4.45E-61
ATP5J2-P1	ENSG0000	3.96	1.075	1.257	3.69E-31
LGALS9	ENSG0000	20.79	8.12	1.257	1.77E-17
CBR1	ENSG0000	181.056	75.123	1.258	1.46E-21
PFDN4	ENSG0000	21.78	8.525	1.258	3.44E-54
SPSB2	ENSG0000	6.75	2.24	1.258	4.55E-54
APOLD1	ENSG0000	4.3	1.215	1.259	1.19E-25
SCD	ENSG0000	205.457	85.176	1.26	1.12E-05

RPP21	ENSG0000	44.901	18.17	1.26	2.39E-62
ATP5L	ENSG0000	304.183	126.381	1.261	4.57E-59
ZIC2	ENSG0000	1.42	0.01	1.261	2.17E-31
EXOSC5	ENSG0000	22.461	8.788	1.261	1.15E-60
CPD	ENSG0000	16.511	6.295	1.263	8.17E-23
EIF3K	ENSG0000	205.429	85.005	1.263	5.18E-70
TMEM147	ENSG0000	69.05	28.196	1.263	1.04E-50
AIM1L	ENSG0000	1.81	0.17	1.264	1.32E-33
BYSL	ENSG0000	12.53	4.635	1.264	2.49E-42
TMEM101	ENSG0000	23.46	9.185	1.264	7.51E-62
RBM8A	ENSG0000	83.152	34.049	1.264	3.29E-57
PHF19	ENSG0000	6.2	1.995	1.265	3.53E-35
NES	ENSG0000	5.32	1.63	1.265	6.86E-33
ZNF687	ENSG0000	11.88	4.36	1.265	3.11E-45
SLC38A6	ENSG0000	6.79	2.24	1.266	5.55E-61
NOL7	ENSG0000	38.55	15.44	1.266	1.50E-59
AC016739	ENSG0000	12.83	4.745	1.267	4.16E-35
ATP1B3	ENSG0000	21.14	8.2	1.267	1.30E-18
CALR	ENSG0000	813.782	337.302	1.268	2.50E-47
NDUFA1	ENSG0000	183.101	75.371	1.269	2.97E-71
PFDN2	ENSG0000	104.959	42.964	1.269	3.23E-57
SLC39A1	ENSG0000	72.098	29.34	1.269	8.80E-45
GINS2	ENSG0000	2.4	0.41	1.27	1.15E-44
PYCR1	ENSG0000	6.32	2.035	1.27	4.77E-11
H2AFV	ENSG0000	78.302	31.871	1.271	2.69E-68
RPL15P3	ENSG0000	8.69	3.015	1.271	6.17E-38
FTH1	ENSG0000	3615.3	1496.627	1.272	3.64E-49
NMB	ENSG0000	3.43	0.835	1.272	8.27E-32
LYPLAL1	ENSG0000	36.221	14.405	1.273	2.19E-48
POLD1	ENSG0000	13.28	4.905	1.274	6.07E-49
TTC39A	ENSG0000	2.76	0.555	1.274	3.15E-21
UCP2	ENSG0000	14.85	5.555	1.274	9.18E-13
GSTA4	ENSG0000	18.01	6.855	1.275	2.86E-28
COX7C	ENSG0000	750.183	309.093	1.276	2.01E-63
RFXANK	ENSG0000	44.099	17.625	1.276	1.95E-58
SOWAHA	ENSG0000	2.79	0.565	1.276	1.14E-34
SEMA3F	ENSG0000	5.44	1.655	1.278	1.43E-50
UBE2Q1	ENSG0000	39.719	15.774	1.279	3.97E-40
NAA20	ENSG0000	71.858	29.014	1.279	1.48E-58
RP11-51C	ENSG0000	21.64	8.325	1.28	1.06E-44
ORMDL2	ENSG0000	48.691	19.455	1.281	9.75E-68
LSM8	ENSG0000	21.201	8.135	1.281	6.01E-51
OLFM2	ENSG0000	31.021	12.169	1.282	6.92E-10
SPCS2P4	ENSG0000	30.96	12.14	1.282	2.97E-41
CDK5	ENSG0000	12.8	4.67	1.283	5.06E-63
RFWD2	ENSG0000	30.789	12.06	1.283	9.26E-68
FAM103A1	ENSG0000	6.95	2.265	1.284	2.92E-28
CD163L1	ENSG0000	2.63	0.49	1.285	1.64E-30
PPT1	ENSG0000	19.291	7.325	1.285	1.05E-31
ST14	ENSG0000	8.05	2.715	1.285	2.33E-11
ADAM15	ENSG0000	42.241	16.735	1.286	1.78E-44
HSPG2	ENSG0000	22.611	8.685	1.286	9.63E-24
STK39	ENSG0000	2.72	0.525	1.286	7.44E-24
TRAC	ENSG0000	7.94	2.665	1.286	3.38E-12
C6orf48	ENSG0000	78.378	31.529	1.287	4.33E-40
ENSA	ENSG0000	109.949	44.47	1.287	1.18E-60
NME1-NM	ENSG0000	398.434	162.714	1.287	1.52E-54
ZNRD1	ENSG0000	25.93	10.03	1.288	1.05E-59

ITGB1BP1	ENSG0000	32.839	12.855	1.288	1.65E-48
ATP5J2	ENSG0000	584.111	238.425	1.289	2.71E-73
CTSC	ENSG0000	35.961	14.13	1.289	1.43E-20
COX6C	ENSG0000	582.17	237.502	1.29	3.06E-60
TMEM50A	ENSG0000	50.779	20.175	1.29	2.01E-54
RP11-161	ENSG0000	58.602	23.37	1.29	4.29E-30
ASPH	ENSG0000	41.04	16.18	1.291	4.24E-21
LMCD1	ENSG0000	10.45	3.68	1.291	2.04E-21
FOXRED2	ENSG0000	6.45	2.045	1.291	1.30E-29
OLA1	ENSG0000	40.941	16.146	1.291	2.43E-44
PRIM2	ENSG0000	4.04	1.06	1.291	2.40E-57
HSPA4	ENSG0000	68.029	27.19	1.292	1.13E-52
BUB1	ENSG0000	1.72	0.11	1.293	3.44E-40
VIM	ENSG0000	130.273	52.559	1.293	1.76E-19
LAMA4	ENSG0000	4.83	1.38	1.293	2.58E-36
RPS2P55	ENSG0000	2.26	0.33	1.293	2.92E-51
RPS18	ENSG0000	2144.461	874.278	1.293	5.72E-48
PDGFB	ENSG0000	4.05	1.06	1.294	4.44E-36
STC1	ENSG0000	2.25	0.325	1.294	2.19E-22
CDKN2AIF	ENSG0000	16.91	6.3	1.295	5.74E-71
AP1M2	ENSG0000	2.83	0.56	1.296	4.23E-15
TATDN1	ENSG0000	22.869	8.72	1.296	7.80E-54
GLA	ENSG0000	19.061	7.165	1.297	6.62E-36
CCDC107	ENSG0000	40.179	15.745	1.298	1.88E-61
CXorf36	ENSG0000	2.25	0.32	1.3	1.83E-66
OIP5	ENSG0000	1.88	0.17	1.3	3.71E-42
NDUFS6	ENSG0000	89.12	35.605	1.3	6.13E-68
CKB	ENSG0000	26.379	10.115	1.301	4.73E-13
ANXA13	ENSG0000	7.52	2.45	1.304	2.16E-08
IL3RA	ENSG0000	2.95	0.6	1.304	7.64E-59
BRMS1	ENSG0000	30.751	11.85	1.305	3.54E-69
HNRNPA1	ENSG0000	363.698	146.569	1.305	6.58E-44
FAM222A	ENSG0000	4.25	1.125	1.305	9.68E-36
DUSP9	ENSG0000	2.14	0.27	1.306	1.47E-24
IFI27L2	ENSG0000	12.91	4.625	1.306	1.37E-20
SLC52A2	ENSG0000	14.78	5.38	1.307	2.94E-44
MESP1	ENSG0000	3.86	0.96	1.31	6.00E-48
P4HA2	ENSG0000	15.899	5.81	1.311	1.88E-35
H2BFS	ENSG0000	7.35	2.365	1.311	1.07E-27
ECT2	ENSG0000	3	0.61	1.313	4.23E-34
RPLP1	ENSG0000	1491.242	599.659	1.313	5.50E-68
TM7SF2	ENSG0000	103.407	41.026	1.313	3.57E-16
FAM189B	ENSG0000	12.53	4.445	1.313	1.79E-47
VPS28	ENSG0000	192.071	76.699	1.313	3.18E-66
MRPL9	ENSG0000	49.261	19.215	1.314	1.94E-66
TYMSOS	ENSG0000	2.58	0.44	1.314	1.16E-36
SMIM11	ENSG0000	4.77	1.32	1.314	3.18E-14
DYNLL1	ENSG0000	246.304	98.267	1.317	1.33E-55
RPS4X	ENSG0000	486.365	194.576	1.317	6.51E-48
HEXB	ENSG0000	156.096	62.003	1.318	5.25E-66
PYGB	ENSG0000	14.47	5.205	1.318	6.43E-36
COMMD5	ENSG0000	24.38	9.165	1.32	5.36E-65
TXNRD1	ENSG0000	30.751	11.718	1.32	1.14E-22
SHISA4	ENSG0000	11.06	3.83	1.32	1.95E-21
RP11-452	ENSG0000	11.78	4.12	1.32	1.81E-47
CD63	ENSG0000	685.588	273.832	1.321	1.84E-54
SUB1	ENSG0000	184.144	73.124	1.321	2.45E-61
TCEB1	ENSG0000	67.911	26.575	1.321	2.54E-55

LRRC1	ENSG0000	2.35	0.34	1.322	2.45E-27
TRAPPC2E	ENSG0000	10.94	3.775	1.322	5.49E-35
AL158801.	ENSG0000	2.19	0.275	1.323	4.13E-33
DCAF13	ENSG0000	17.179	6.265	1.323	4.36E-45
MPC2	ENSG0000	279.053	110.929	1.323	4.12E-61
UHRF1	ENSG0000	1.79	0.115	1.323	6.25E-35
C8orf59	ENSG0000	87.069	34.17	1.324	3.10E-61
VARS	ENSG0000	36.521	13.985	1.324	3.20E-47
ANAPC11	ENSG0000	192.35	76.061	1.327	1.35E-65
GABRD	ENSG0000	1.66	0.06	1.327	1.49E-62
RPL23AP6	ENSG0000	2.83	0.525	1.329	2.76E-26
XRCC1	ENSG0000	13.98	4.955	1.331	6.31E-57
RPL7	ENSG0000	1178.47	467.589	1.332	4.79E-43
PSMG3	ENSG0000	31.519	11.92	1.332	7.20E-62
MANEAL	ENSG0000	9.16	3.035	1.332	1.01E-29
CDC6	ENSG0000	2.44	0.365	1.334	8.64E-29
ATRN	ENSG0000	27.481	10.28	1.336	3.10E-24
RAN	ENSG0000	208.441	81.905	1.337	2.40E-41
PLOD3	ENSG0000	60.601	23.364	1.338	1.88E-53
TECRP1	ENSG0000	6.04	1.785	1.338	4.63E-39
IL32	ENSG0000	748.417	295.25	1.339	1.34E-21
SAE1	ENSG0000	33.261	12.505	1.343	1.26E-52
EMC3	ENSG0000	74.279	28.66	1.344	1.83E-55
RPS20	ENSG0000	1550.372	610.077	1.344	9.99E-54
CENPH	ENSG0000	3.04	0.59	1.345	7.84E-51
DTYMK	ENSG0000	19.68	7.14	1.345	3.89E-55
NUP37	ENSG0000	10.3	3.445	1.346	1.67E-59
SDC2	ENSG0000	176.557	68.859	1.346	1.43E-24
TOMM7	ENSG0000	327.397	128.181	1.346	3.43E-57
TRNP1	ENSG0000	5.14	1.415	1.346	8.41E-12
ARPC1B	ENSG0000	99.181	38.33	1.349	1.13E-27
COL1A1	ENSG0000	28.3	10.504	1.349	2.14E-09
FLAD1	ENSG0000	34.291	12.85	1.349	4.75E-73
NFKBIE	ENSG0000	11.85	4.04	1.35	4.40E-28
BCAM	ENSG0000	57.46	21.915	1.351	2.50E-22
DAPK2	ENSG0000	4.37	1.105	1.351	2.77E-40
FBL	ENSG0000	123.391	47.738	1.352	7.33E-50
TRMT112	ENSG0000	215.358	83.786	1.352	3.43E-75
IRAK1	ENSG0000	36.75	13.79	1.352	1.22E-39
EXO1	ENSG0000	1.81	0.1	1.353	1.41E-45
CTSS	ENSG0000	37.281	13.98	1.354	5.87E-11
LAMA3	ENSG0000	3.81	0.88	1.355	1.87E-22
RPLP2	ENSG0000	879.597	343.329	1.355	2.02E-51
MSH2	ENSG0000	5.18	1.415	1.356	2.42E-46
RRM1	ENSG0000	13.68	4.735	1.356	1.14E-46
CHAF1A	ENSG0000	5.12	1.39	1.357	4.33E-53
AP1S1	ENSG0000	45.54	17.135	1.36	4.79E-61
PPIA	ENSG0000	1363.405	530.68	1.36	1.65E-75
PSMB4	ENSG0000	347.073	134.526	1.361	3.11E-83
EIF3H	ENSG0000	142.754	54.924	1.362	7.81E-48
RFX5	ENSG0000	10.55	3.49	1.363	4.80E-36
UXS1	ENSG0000	15.49	5.405	1.364	1.89E-47
TMCO3	ENSG0000	15.33	5.335	1.366	1.16E-24
UQCRH	ENSG0000	217.594	83.829	1.366	1.14E-57
H2AFJ	ENSG0000	110.208	42.105	1.367	5.74E-43
MCM7	ENSG0000	32.919	12.145	1.368	6.89E-41
ATP1B1	ENSG0000	97.951	37.295	1.37	1.12E-29
PRCC	ENSG0000	41.28	15.355	1.37	7.36E-76

HJURP	ENSG0000	2.22	0.245	1.371	2.04E-40
ARL2	ENSG0000	24.729	8.94	1.372	6.90E-39
LINC0051:	ENSG0000	1.86	0.105	1.372	2.19E-23
GMPS	ENSG0000	19.41	6.88	1.373	9.06E-52
HMGN1P3	ENSG0000	1.59	0	1.373	5.96E-38
AATF	ENSG0000	29.171	10.64	1.374	3.60E-73
CLDN15	ENSG0000	24.411	8.805	1.374	1.73E-16
RPL35P5	ENSG0000	5.17	1.38	1.374	7.81E-45
C15orf48	ENSG0000	3.02	0.55	1.375	7.15E-16
IFI6	ENSG0000	95.742	36.279	1.376	2.97E-10
ATP1A1	ENSG0000	136.342	51.872	1.377	1.61E-31
MAZ	ENSG0000	114.332	43.406	1.377	1.78E-68
NHP2	ENSG0000	99.332	37.635	1.377	6.68E-71
NDUFB9	ENSG0000	377.752	144.862	1.377	2.15E-64
PRAP1	ENSG0000	676.102	259.618	1.377	4.48E-05
FLVCR1	ENSG0000	3.43	0.705	1.378	3.91E-51
YWHAZ	ENSG0000	107.38	40.699	1.378	1.29E-36
TUBA1C	ENSG0000	72.028	27.1	1.378	5.52E-19
ALYREF	ENSG0000	37.749	13.9	1.379	1.14E-52
DRAP1	ENSG0000	101.391	38.361	1.379	4.29E-83
PLP2	ENSG0000	24.319	8.734	1.379	1.90E-17
PDZD11	ENSG0000	30.37	11.065	1.379	4.14E-71
FANCI	ENSG0000	3.08	0.565	1.382	1.22E-44
NRM	ENSG0000	8.47	2.63	1.383	1.91E-38
RPS21	ENSG0000	1270.692	486.517	1.383	9.69E-56
RPL39L	ENSG0000	5.42	1.46	1.384	1.23E-15
PSPH	ENSG0000	8.69	2.705	1.387	2.07E-35
EPRS	ENSG0000	37.111	13.56	1.388	2.11E-61
FLYWCH2	ENSG0000	14.31	4.85	1.388	1.74E-39
NME2	ENSG0000	15.899	5.455	1.389	5.15E-22
PPDPF	ENSG0000	190.982	72.223	1.391	1.61E-16
PXMP4	ENSG0000	10.38	3.335	1.392	3.71E-58
BOLA3	ENSG0000	36.339	13.22	1.393	4.46E-65
RP11-713.	ENSG0000	10.17	3.25	1.394	3.38E-32
BMS1P8	ENSG0000	1.92	0.11	1.395	1.45E-28
RPL37	ENSG0000	1566.576	594.465	1.396	2.08E-66
EXOSC4	ENSG0000	41.63	15.195	1.396	1.23E-55
HIST1H1C	ENSG0000	128.818	48.314	1.396	2.82E-24
MARCKS	ENSG0000	15.05	5.1	1.396	5.08E-28
TESC	ENSG0000	7.24	2.13	1.397	5.28E-16
ZNF706	ENSG0000	102.992	38.45	1.398	4.72E-59
FAM83H	ENSG0000	14.96	5.055	1.398	1.52E-39
SLC50A1	ENSG0000	65.289	24.135	1.399	1.38E-50
VAMP5	ENSG0000	90.88	33.767	1.402	1.87E-43
TOMM6	ENSG0000	230.017	86.413	1.402	1.25E-72
S100A13	ENSG0000	75.29	27.839	1.403	6.17E-19
CUTA	ENSG0000	288.894	108.548	1.404	1.34E-74
Metazoa_S	ENSG0000	343.769	129.247	1.404	1.20E-14
HLA-DMB	ENSG0000	14.22	4.74	1.407	5.56E-17
TPM3	ENSG0000	124.154	46.16	1.408	3.43E-50
SELM	ENSG0000	33.981	12.185	1.408	9.10E-14
LOXL4	ENSG0000	3.58	0.72	1.413	6.30E-08
ATP6AP1	ENSG0000	65.639	24.015	1.414	1.58E-72
CADM1	ENSG0000	40.07	14.41	1.414	1.01E-20
EHMT2	ENSG0000	23.36	8.135	1.415	1.27E-58
MMP14	ENSG0000	15	5	1.415	6.88E-21
SNRPGP2	ENSG0000	9.32	2.87	1.415	5.66E-38
FDPS	ENSG0000	291.63	108.698	1.416	2.85E-38

ATP5G1	ENSG0000	239.967	89.216	1.417	2.90E-65
H2AFX	ENSG0000	14.94	4.97	1.417	8.39E-40
RP11-464	ENSG0000	2.61	0.35	1.419	1.55E-43
UQCRB	ENSG0000	349.269	129.912	1.42	4.17E-69
LSM2	ENSG0000	38.869	13.895	1.42	2.80E-64
PAQR4	ENSG0000	3.82	0.8	1.421	7.08E-32
KIAA1522	ENSG0000	11.99	3.85	1.421	4.79E-36
KIF4A	ENSG0000	1.87	0.07	1.423	4.49E-43
SPC25	ENSG0000	2.11	0.16	1.423	3.18E-47
RPL38	ENSG0000	1230.73	457.934	1.424	8.63E-66
SLC41A3	ENSG0000	18.379	6.215	1.425	5.16E-66
HLA-DRB	ENSG0000	4.28	0.965	1.426	1.57E-14
FAT1	ENSG0000	33.42	11.8	1.427	3.61E-20
HIGD1B	ENSG0000	2.2	0.19	1.427	6.54E-69
TUBG1	ENSG0000	24.47	8.475	1.427	1.90E-43
CACYBP	ENSG0000	43.419	15.51	1.428	6.64E-39
CTHRC1	ENSG0000	2.35	0.245	1.428	3.20E-24
CDC25C	ENSG0000	1.84	0.055	1.429	3.83E-53
NENF	ENSG0000	81.311	29.561	1.429	4.92E-71
IFI30	ENSG0000	140.769	51.639	1.429	1.31E-30
RPN2	ENSG0000	201.829	74.294	1.43	1.68E-75
GTF2IP4	ENSG0000	23.86	8.225	1.43	1.57E-28
RAMP2	ENSG0000	17.27	5.77	1.432	1.02E-32
RPL23AP4	ENSG0000	644.751	238.359	1.432	1.41E-52
MEA1	ENSG0000	62.691	22.585	1.433	2.41E-84
PLK1	ENSG0000	3.77	0.765	1.434	6.79E-36
SAC3D1	ENSG0000	13.78	4.47	1.434	1.33E-70
RBM34	ENSG0000	44.711	15.919	1.434	4.00E-70
GDF15	ENSG0000	44.51	15.795	1.438	1.80E-15
PKM	ENSG0000	38.791	13.675	1.439	7.36E-21
TMEM9	ENSG0000	78.102	28.145	1.44	6.19E-64
PBXIP1	ENSG0000	38.369	13.51	1.44	5.51E-53
LMNB1	ENSG0000	7.73	2.21	1.443	2.22E-27
MRPS23	ENSG0000	28.859	9.985	1.443	1.07E-47
RNF157	ENSG0000	3.3	0.58	1.444	5.21E-26
CCNA2	ENSG0000	2.96	0.455	1.445	1.85E-36
PRIM1	ENSG0000	5.97	1.56	1.445	1.73E-42
TACC3	ENSG0000	10.26	3.13	1.447	2.48E-40
LYPD1	ENSG0000	2.34	0.225	1.447	3.30E-24
RP11-443	ENSG0000	1.78	0.02	1.447	1.02E-27
COX6B1	ENSG0000	407.285	148.543	1.449	4.41E-65
SNRPD2	ENSG0000	193.165	70.121	1.449	8.88E-60
PRPF6	ENSG0000	40.029	14.02	1.45	6.06E-85
MTX1	ENSG0000	58.838	20.895	1.45	1.55E-82
SERF1B	ENSG0000	18.65	6.19	1.45	1.62E-60
SRXN1	ENSG0000	30.141	10.395	1.45	1.06E-28
RHOC	ENSG0000	174.755	63.284	1.451	4.75E-51
APOBEC3	ENSG0000	2.34	0.22	1.453	2.35E-29
TRIM31	ENSG0000	2.55	0.295	1.455	7.90E-21
RP1-241P	ENSG0000	3.77	0.74	1.455	9.14E-15
KIF2C	ENSG0000	2.21	0.17	1.456	6.33E-43
UCK2	ENSG0000	18.22	6	1.457	5.06E-48
RFC4	ENSG0000	12.42	3.89	1.457	1.42E-48
PHLDA2	ENSG0000	4.54	1.015	1.459	2.33E-12
RPLP0P6	ENSG0000	13.89	4.415	1.459	7.08E-44
ATP6V1F	ENSG0000	102.047	36.464	1.46	5.33E-67
NPM3	ENSG0000	17.14	5.595	1.46	2.79E-27
ZNF28	ENSG0000	3.54	0.65	1.46	1.30E-20

SNRPD1	ENSG0000	42.22	14.7	1.461	1.13E-60
RHNO1	ENSG0000	6.63	1.77	1.462	2.76E-52
CENPA	ENSG0000	2.09	0.12	1.464	6.70E-44
DYNLRB1	ENSG0000	145.139	51.949	1.465	1.38E-80
CKAP4	ENSG0000	32.45	11.11	1.466	9.61E-39
NOTCH3	ENSG0000	4.32	0.925	1.467	5.02E-30
EIF3E	ENSG0000	194.253	69.545	1.469	1.00E-45
ATAD2	ENSG0000	6.91	1.855	1.47	1.16E-38
MRPS21	ENSG0000	63.999	22.47	1.47	1.96E-74
ANLN	ENSG0000	2.26	0.175	1.472	2.75E-40
DBP	ENSG0000	18.81	6.13	1.474	2.74E-34
PIGT	ENSG0000	65.05	22.78	1.474	1.20E-72
ANKRD29	ENSG0000	3.48	0.61	1.476	6.70E-29
PLA2G4C	ENSG0000	16.41	5.25	1.478	3.54E-30
RAB11FIP4	ENSG0000	5.22	1.225	1.483	3.08E-36
DARS2	ENSG0000	10.48	3.105	1.484	3.52E-50
RELB	ENSG0000	13.58	4.209	1.485	2.92E-31
MYL6B	ENSG0000	27.219	9.079	1.485	1.42E-52
HLA-B	ENSG0000	788.298	281.004	1.485	1.15E-16
PSMB9	ENSG0000	50.011	17.194	1.487	6.35E-29
ABHD12	ENSG0000	35.671	12.07	1.488	3.60E-75
MAGED2	ENSG0000	63.711	22.045	1.49	1.27E-57
G6PD	ENSG0000	9.9	2.875	1.492	1.33E-28
IFI27L1	ENSG0000	14.55	4.515	1.495	1.39E-60
TMED3	ENSG0000	20.71	6.7	1.495	1.13E-09
PBK	ENSG0000	2.1	0.1	1.495	8.04E-41
DTL	ENSG0000	2.23	0.145	1.496	1.04E-41
RP11-284	ENSG0000	1.99	0.06	1.496	3.11E-29
HLA-DPA	ENSG0000	49.969	17.045	1.498	2.45E-11
TM4SF1	ENSG0000	41.469	14.024	1.499	5.03E-16
LTB	ENSG0000	7.99	2.18	1.499	1.25E-15
RPL41	ENSG0000	2353.512	831.227	1.5	7.63E-52
HIST1H2B	ENSG0000	106.042	36.82	1.501	4.03E-23
PFDN6	ENSG0000	118.569	41.236	1.501	1.97E-73
CKS1B	ENSG0000	71.878	24.695	1.504	3.76E-61
TXN	ENSG0000	347.458	121.873	1.504	1.17E-50
KIAA1462	ENSG0000	3.31	0.52	1.504	9.40E-51
POLR2K	ENSG0000	55.819	19.015	1.505	8.99E-80
NPM1	ENSG0000	485.355	170.378	1.505	2.36E-47
RP11-215	ENSG0000	2.32	0.17	1.505	1.69E-56
ROMO1	ENSG0000	249.154	86.933	1.508	3.79E-69
PODXL	ENSG0000	5.7	1.355	1.508	8.20E-32
LEF1	ENSG0000	2.67	0.29	1.508	8.05E-29
H3F3A	ENSG0000	397.496	139.034	1.509	9.56E-73
ARPC5	ENSG0000	86.762	29.82	1.51	3.85E-56
ZFAS1	ENSG0000	81.002	27.78	1.511	7.16E-40
GM2A	ENSG0000	16	4.965	1.511	1.23E-64
BCAP31	ENSG0000	202.657	70.416	1.512	1.98E-72
LINC0129	ENSG0000	2.28	0.15	1.512	2.92E-34
EEF1A1P6	ENSG0000	11.7	3.45	1.513	1.01E-30
HSP90AB1	ENSG0000	400.622	139.516	1.515	4.29E-45
CDCA8	ENSG0000	2.69	0.29	1.516	7.21E-44
SNCG	ENSG0000	6.78	1.72	1.516	1.21E-23
RP11-61N	ENSG0000	6.12	1.49	1.516	1.21E-19
RPL39P3	ENSG0000	319.151	110.798	1.518	2.15E-18
HMGB2	ENSG0000	34.871	11.52	1.519	1.95E-43
ITPKA	ENSG0000	3.46	0.555	1.52	3.00E-30
GPAA1	ENSG0000	115.511	39.639	1.52	7.85E-66

RP11-641	ENSG0000	174.126	60.058	1.52	2.02E-49
APOA2	ENSG0000	31278.85	10880.07	1.523	9.24E-04
NT5DC2	ENSG0000	11.39	3.31	1.523	4.62E-26
NR2C2AP	ENSG0000	14.33	4.33	1.524	1.09E-71
GBP2	ENSG0000	45.972	15.32	1.525	3.30E-36
SCAMP5	ENSG0000	3.87	0.69	1.527	2.60E-24
UFC1	ENSG0000	97.497	33.145	1.528	7.85E-76
BOP1	ENSG0000	28.041	9.04	1.532	7.46E-50
HLA-DMA	ENSG0000	37.32	12.255	1.532	1.92E-20
PECAM1	ENSG0000	11.39	3.285	1.532	2.92E-39
TNFRSF12	ENSG0000	46.019	15.247	1.533	8.77E-15
VAT1	ENSG0000	49.37	16.41	1.533	1.56E-44
RPL7P9	ENSG0000	28.391	9.16	1.533	1.79E-48
SCNM1	ENSG0000	33.21	10.825	1.533	9.85E-72
XXbac-BP	ENSG0000	5.75	1.324	1.538	9.66E-05
RNASE1	ENSG0000	56.531	18.8	1.539	4.55E-29
MRPL24	ENSG0000	161.735	54.922	1.541	2.07E-75
PTP4A3	ENSG0000	8.63	2.31	1.541	1.56E-32
SNX8	ENSG0000	17.73	5.43	1.542	3.64E-55
TLCD1	ENSG0000	16.11	4.87	1.543	4.12E-47
TOMM20	ENSG0000	65.871	21.944	1.543	3.13E-69
HIST2H2A	ENSG0000	29.59	9.48	1.545	1.62E-12
LGALS3BP	ENSG0000	160.385	54.264	1.546	2.76E-08
ILF2	ENSG0000	80.991	27.02	1.549	3.65E-67
ITIH2	ENSG0000	623.739	212.342	1.55	1.81E-09
CXCL9	ENSG0000	4.18	0.765	1.553	5.77E-14
MELK	ENSG0000	2.23	0.1	1.554	1.08E-44
GNAZ	ENSG0000	2.38	0.15	1.555	1.68E-35
CLN3	ENSG0000	58.809	19.355	1.555	2.78E-68
SUMO2	ENSG0000	146.859	49.28	1.556	7.57E-57
CPE	ENSG0000	15.74	4.685	1.558	2.12E-24
HMMR	ENSG0000	2.54	0.2	1.561	4.19E-43
RGS10	ENSG0000	7.6	1.915	1.561	4.08E-31
CCL15	ENSG0000	29.421	9.305	1.562	1.09E-23
BSG	ENSG0000	301.329	101.335	1.563	1.20E-64
RP11-620	ENSG0000	5.08	1.055	1.565	8.54E-53
U91328.1	ENSG0000	1.96	0	1.566	1.88E-03
SLC44A3	ENSG0000	6.79	1.63	1.567	4.44E-26
FAM83D	ENSG0000	4.5	0.855	1.568	1.65E-36
OLFML2B	ENSG0000	2.78	0.275	1.568	3.12E-45
UGT2B11	ENSG0000	3.8	0.615	1.572	4.59E-20
SNRPC	ENSG0000	118.742	39.239	1.573	4.96E-80
SOX9	ENSG0000	4.95	1	1.573	3.90E-17
RPL36A	ENSG0000	883.691	296.337	1.573	5.82E-59
RP11-345	ENSG0000	18.04	5.385	1.576	5.76E-58
H2AFZ	ENSG0000	99.808	32.785	1.577	4.21E-53
RMI2	ENSG0000	3.39	0.47	1.578	1.38E-41
TMCO1	ENSG0000	124.94	41.106	1.581	1.55E-60
IQGAP3	ENSG0000	2.35	0.12	1.581	4.48E-45
HLA-DQA	ENSG0000	12.49	3.505	1.582	1.17E-09
UQCC2	ENSG0000	98.373	32.169	1.583	7.45E-74
SQSTM1	ENSG0000	460.225	152.9	1.583	1.68E-40
RPL30	ENSG0000	1964.708	654.091	1.585	1.70E-64
ITGA6	ENSG0000	10.25	2.74	1.589	1.72E-41
TNFRSF4	ENSG0000	2.97	0.32	1.589	4.05E-65
CENPU	ENSG0000	4.69	0.885	1.594	4.48E-42
PIGU	ENSG0000	16.75	4.875	1.595	1.06E-81
ATOX1	ENSG0000	347.29	114.178	1.596	6.63E-63

MAN2B1	ENSG0000	55.591	17.704	1.597	1.74E-60
HLA-F	ENSG0000	95.709	30.939	1.598	5.53E-21
EPHX1	ENSG0000	1532.848	505.37	1.599	2.23E-17
RPL37P6	ENSG0000	2.03	0	1.599	8.18E-40
HMGN2P5	ENSG0000	42.659	13.404	1.6	2.32E-34
NCAPH	ENSG0000	2.55	0.17	1.601	2.29E-45
PIGC	ENSG0000	22.07	6.605	1.601	1.92E-71
CCDC34	ENSG0000	5.74	1.22	1.602	1.03E-51
GPNMB	ENSG0000	12.74	3.525	1.602	4.77E-17
SORT1	ENSG0000	7.08	1.66	1.603	2.69E-34
COMMD4	ENSG0000	110.17	35.546	1.605	1.73E-97
CKLF	ENSG0000	29.829	9.115	1.608	2.26E-62
PSMD4	ENSG0000	242.576	78.784	1.61	2.53E-79
MND1	ENSG0000	2.58	0.17	1.613	4.58E-64
AP003391.	ENSG0000	2.06	0	1.614	1.08E-03
ENAH	ENSG0000	10.65	2.805	1.614	2.54E-29
HDAC11	ENSG0000	8.49	2.1	1.614	4.48E-54
STXBP6	ENSG0000	6.17	1.34	1.615	9.91E-40
PYCARD	ENSG0000	16.68	4.764	1.617	1.09E-21
APOA1BP	ENSG0000	120.684	38.634	1.618	9.18E-90
CNIH4	ENSG0000	32.81	10.014	1.618	1.20E-64
NUF2	ENSG0000	2.48	0.13	1.623	8.54E-46
CDC45	ENSG0000	3.07	0.32	1.624	7.92E-45
COX4I2	ENSG0000	3.1	0.33	1.624	4.93E-71
ANXA2P2	ENSG0000	4.42	0.755	1.627	1.14E-37
RP11-316	ENSG0000	78.699	24.778	1.628	2.61E-19
SSR2	ENSG0000	259.267	82.939	1.633	6.01E-67
RRS1	ENSG0000	16	4.47	1.636	1.32E-34
GRN	ENSG0000	187.857	59.719	1.637	6.44E-71
RP3-461F.	ENSG0000	7.65	1.78	1.638	3.70E-34
SHFM1	ENSG0000	305.07	97.133	1.641	1.47E-81
RP11-345.	ENSG0000	2.12	0	1.642	1.56E-04
SF3B4	ENSG0000	35.839	10.79	1.644	1.44E-68
TKT	ENSG0000	113.921	35.77	1.644	3.14E-33
RP11-756	ENSG0000	2.13	0	1.646	2.60E-20
LAGE3	ENSG0000	36.59	11.005	1.647	2.33E-67
VIL1	ENSG0000	14.91	4.055	1.654	1.06E-07
CPQ	ENSG0000	47.94	14.525	1.656	4.21E-58
NTPCR	ENSG0000	54.161	16.505	1.656	2.67E-68
BOLA2	ENSG0000	10.8	2.74	1.658	3.31E-53
C12orf75	ENSG0000	6.37	1.335	1.658	2.87E-18
BAX	ENSG0000	78.71	24.245	1.659	1.38E-59
MCAM	ENSG0000	14.76	3.97	1.665	3.84E-36
VPS72	ENSG0000	28.489	8.295	1.666	7.94E-78
KRT23	ENSG0000	2.85	0.21	1.67	2.75E-10
MAD2L1	ENSG0000	3.9	0.535	1.675	1.61E-47
UGT1A6	ENSG0000	70.37	21.275	1.68	2.54E-05
DPP4	ENSG0000	19.36	5.345	1.682	3.61E-16
ARSEP1	ENSG0000	2.5	0.09	1.683	1.24E-10
PKN1	ENSG0000	44.609	13.205	1.683	1.92E-45
TUBA1B	ENSG0000	234.996	72.433	1.684	8.76E-30
CDH13	ENSG0000	3.25	0.32	1.687	2.95E-66
NCAPG	ENSG0000	2.59	0.115	1.687	1.63E-46
SEZ6L2	ENSG0000	2.88	0.205	1.687	3.02E-23
RAMP1	ENSG0000	103.679	31.471	1.689	1.78E-15
AC005943	ENSG0000	2.24	0	1.696	5.01E-03
HN1	ENSG0000	64.208	19.065	1.7	8.00E-54
RP11-295	ENSG0000	20.34	5.555	1.703	9.96E-37

SPARC	ENSG0000	214.881	65.26	1.704	8.74E-29
CLIC1	ENSG0000	143.867	43.331	1.708	1.91E-47
CYSTM1	ENSG0000	72.781	21.57	1.709	6.22E-58
MCM4	ENSG0000	11.24	2.725	1.716	1.41E-37
TAGLN2	ENSG0000	252.545	76.164	1.716	3.34E-43
RPL8	ENSG0000	1876.722	569.775	1.718	6.14E-59
RACGAP1	ENSG0000	4.61	0.705	1.718	1.73E-50
SCAMP3	ENSG0000	85.793	25.355	1.72	5.81E-83
HLA-DPB1	ENSG0000	59.821	17.4	1.725	2.74E-14
NDC80	ENSG0000	2.91	0.18	1.728	3.06E-52
KRTCAP2	ENSG0000	342.651	102.454	1.732	1.10E-93
NME1	ENSG0000	100.099	29.344	1.736	3.98E-60
MKI67	ENSG0000	2.9	0.17	1.737	6.18E-46
ASF1B	ENSG0000	3.27	0.28	1.738	7.91E-48
PCNA	ENSG0000	48.09	13.71	1.739	1.99E-57
NSMCE2	ENSG0000	20.92	5.565	1.739	4.91E-85
MCM3	ENSG0000	20.53	5.435	1.742	9.00E-46
GLMP	ENSG0000	123.023	36.071	1.742	4.49E-67
CTSA	ENSG0000	181.974	53.563	1.746	1.71E-70
KIF20A	ENSG0000	2.79	0.13	1.746	9.12E-45
CKS2	ENSG0000	31.349	8.62	1.75	3.56E-45
RNF185-A	ENSG0000	3.09	0.212	1.755	6.30E-05
TMEM45B	ENSG0000	29.9	8.1	1.764	7.97E-11
PDGFA	ENSG0000	9.08	1.965	1.765	1.02E-27
NEU1	ENSG0000	55.299	15.57	1.765	3.02E-68
ETV4	ENSG0000	3.97	0.46	1.767	9.97E-26
SPATC1L	ENSG0000	3.81	0.41	1.77	3.21E-38
CRIP1	ENSG0000	26.359	7.01	1.772	1.06E-23
TP53I3	ENSG0000	20.09	5.15	1.778	2.13E-35
SNRPEP2	ENSG0000	7.97	1.61	1.781	2.82E-64
SPARCL1	ENSG0000	19.1	4.845	1.782	1.87E-22
SLC51B	ENSG0000	3.87	0.415	1.783	6.74E-27
COL15A1	ENSG0000	3.03	0.17	1.784	4.85E-52
PVT1	ENSG0000	6.01	1.035	1.784	7.25E-20
SNRPB	ENSG0000	126.473	35.895	1.789	1.23E-70
SMIM4	ENSG0000	34.9	9.335	1.796	1.83E-59
SMYD3	ENSG0000	13.12	3.06	1.798	2.36E-59
NUSAP1	ENSG0000	8.31	1.675	1.799	2.72E-40
FEN1	ENSG0000	12.59	2.9	1.801	7.26E-62
FABP5P7	ENSG0000	8.22	1.645	1.801	1.41E-32
ELOVL2	ENSG0000	17.6	4.335	1.802	7.73E-15
PEA15	ENSG0000	45.819	12.42	1.803	3.81E-58
GOLM1	ENSG0000	22.4	5.695	1.805	7.37E-18
SQLE	ENSG0000	36.309	9.635	1.811	2.09E-25
SNRPE	ENSG0000	137.548	38.494	1.811	5.11E-83
ENPP2	ENSG0000	14.99	3.555	1.812	3.56E-22
FOXM1	ENSG0000	5.72	0.91	1.815	2.70E-41
ASPM	ENSG0000	2.96	0.125	1.816	8.72E-54
TMEM106	ENSG0000	44.05	11.765	1.819	6.54E-59
MCM6	ENSG0000	7.88	1.485	1.837	2.31E-49
CCT3	ENSG0000	185.734	51.09	1.842	3.78E-87
DTNA	ENSG0000	6.14	0.99	1.843	4.13E-27
PABPC1	ENSG0000	653.57	181.313	1.844	9.41E-59
RPL7P1	ENSG0000	20.549	5	1.845	1.49E-45
HULC	ENSG0000	127.221	34.625	1.848	6.45E-12
HRCT1	ENSG0000	4.34	0.48	1.851	2.27E-36
TREM2	ENSG0000	3.51	0.245	1.857	9.01E-33
PEG10	ENSG0000	5.21	0.71	1.861	3.12E-13

TCF19	ENSG0000	4.27	0.44	1.872	6.76E-47
CLGN	ENSG0000	2.2	0.6	1.874	5.64E-11
CDKN2C	ENSG0000	7.91	1.415	1.883	4.49E-54
CDT1	ENSG0000	3.68	0.265	1.887	1.30E-49
COA6	ENSG0000	53.471	13.715	1.888	2.18E-88
TMEM98	ENSG0000	30.38	7.44	1.895	2.53E-21
PAFAH1B3	ENSG0000	14.81	3.225	1.904	2.54E-43
NDUFA4L2	ENSG0000	14.62	3.17	1.905	4.81E-41
CRNDE	ENSG0000	4.62	0.5	1.906	2.52E-27
LMNA	ENSG0000	175.52	45.859	1.913	4.12E-85
SULT1C2	ENSG0000	4.09	0.35	1.915	3.66E-19
NELFE	ENSG0000	58.618	14.75	1.92	4.92E-73
NEK2	ENSG0000	3.21	0.11	1.923	2.38E-51
RGS5	ENSG0000	18.01	4.01	1.924	2.20E-34
HSPB1	ENSG0000	644.572	168.996	1.925	2.04E-43
CDCA5	ENSG0000	3.89	0.275	1.939	6.90E-56
HLA-DQB	ENSG0000	22.92	5.189	1.95	3.59E-16
ESM1	ENSG0000	3.22	0.09	1.953	1.59E-52
AKR1C3	ENSG0000	351.236	89.588	1.959	3.03E-46
COL4A2	ENSG0000	30.359	7.015	1.968	1.19E-37
RPS28P7	ENSG0000	722.222	182.518	1.979	1.96E-74
HLA-DRA	ENSG0000	171.468	42.669	1.982	3.47E-16
CDCA3	ENSG0000	5.67	0.685	1.985	4.65E-50
CYP7A1	ENSG0000	10.65	1.939	1.987	7.80E-10
PKMYT1	ENSG0000	5.72	0.695	1.987	1.95E-56
RECQL4	ENSG0000	8.65	1.435	1.987	6.01E-51
TPX2	ENSG0000	6	0.765	1.988	5.60E-45
DBNDD1	ENSG0000	11.41	2.12	1.992	7.68E-36
LGALS3	ENSG0000	30.231	6.845	1.993	1.84E-25
RP11-10G	ENSG0000	2.99	0	1.996	3.34E-66
ANXA2	ENSG0000	254.866	63.065	1.998	1.96E-44
KPNA2	ENSG0000	28.43	6.31	2.009	1.03E-62
BOLA2B	ENSG0000	84.67	20.26	2.011	1.17E-74
AC239868	ENSG0000	22.92	4.91	2.017	4.33E-23
AC239868	ENSG0000	22.92	4.91	2.017	4.33E-23
CD74	ENSG0000	753.727	185.354	2.018	1.25E-25
THBS4	ENSG0000	3.68	0.15	2.025	6.22E-40
AURKB	ENSG0000	5.25	0.53	2.03	3.98E-50
TMSB10	ENSG0000	859.646	208.556	2.038	1.53E-37
LAMC1	ENSG0000	26.829	5.775	2.038	2.21E-43
H3F3AP4	ENSG0000	144.256	34.374	2.038	4.70E-16
LAPTM4B	ENSG0000	34.179	7.56	2.039	7.12E-31
CPVL	ENSG0000	14.5	2.755	2.045	3.69E-20
RGCC	ENSG0000	11.91	2.12	2.049	1.35E-36
UBE2SP2	ENSG0000	3.54	0.085	2.065	3.79E-71
AURKA	ENSG0000	7.35	0.99	2.069	2.38E-56
CAP2	ENSG0000	8.86	1.345	2.072	3.05E-52
UBE2S	ENSG0000	19.39	3.845	2.073	5.29E-67
MYBL2	ENSG0000	4.07	0.19	2.091	3.56E-43
EIF5AP4	ENSG0000	3.95	0.16	2.093	9.78E-37
TYMS	ENSG0000	16.4	3.05	2.103	2.21E-48
HLA-A	ENSG0000	714.504	165.214	2.106	3.22E-46
KIAA0101	ENSG0000	9.86	1.51	2.113	9.70E-50
CENPW	ENSG0000	8.1	1.1	2.115	3.78E-62
CKS1BP3	ENSG0000	5.44	0.475	2.126	1.92E-51
S100A10	ENSG0000	241.168	54.345	2.129	2.01E-41
RP11-667	ENSG0000	6.33	0.655	2.147	5.25E-22
AC005255	ENSG0000	3.45	0	2.154	6.77E-37

TM4SF4	ENSG0000	430.001	95.683	2.156	2.86E-18
KIFC1	ENSG0000	4.37	0.205	2.156	2.31E-55
MCM2	ENSG0000	6.75	0.735	2.159	1.21E-49
GMNN	ENSG0000	31.111	6.19	2.159	2.77E-59
MCM5	ENSG0000	21.45	4.025	2.16	2.99E-56
ERICH5	ENSG0000	18.92	3.41	2.175	1.43E-14
TMEM150	ENSG0000	6.44	0.645	2.177	3.44E-36
HKDC1	ENSG0000	8.11	1.01	2.18	3.33E-23
FABP5	ENSG0000	16.66	2.88	2.186	1.76E-44
CCNB2	ENSG0000	4.81	0.27	2.194	3.46E-57
CTB-63M2	ENSG0000	136.882	29.105	2.195	1.57E-20
ACSM1	ENSG0000	8.56	1.085	2.197	2.43E-16
RNASEH2	ENSG0000	16.151	2.735	2.199	4.56E-77
NUDT1	ENSG0000	15.59	2.595	2.206	2.77E-77
MMP11	ENSG0000	5.24	0.35	2.209	9.60E-39
SPC24	ENSG0000	5.15	0.33	2.209	5.18E-66
RP11-114	ENSG0000	17.64	3.005	2.219	4.02E-19
FTH1P8	ENSG0000	9.88	1.33	2.223	4.15E-60
HMGA1	ENSG0000	36.319	6.9	2.24	8.96E-51
HNRNPCF	ENSG0000	7.96	0.895	2.241	1.00E-63
APOC2	ENSG0000	2260.615	475.779	2.246	3.11E-13
PPIAP22	ENSG0000	165.466	34.006	2.25	9.13E-79
LGALS4	ENSG0000	224.27	46.12	2.257	2.48E-06
AC104534	ENSG0000	5.81	0.42	2.262	6.03E-12
ST8SIA6-A	ENSG0000	3.81	0	2.266	2.87E-27
ROBO1	ENSG0000	9.19	1.07	2.299	1.09E-32
BLVRA	ENSG0000	19.46	3.145	2.303	3.46E-46
EIF4BP7	ENSG0000	1.68	0.34	2.305	3.21E-40
COL4A1	ENSG0000	26.299	4.465	2.321	3.62E-43
RBP7	ENSG0000	17.259	2.635	2.329	3.32E-59
E2F1	ENSG0000	5.49	0.28	2.342	2.90E-57
EPS8L3	ENSG0000	4.76	0.13	2.35	2.06E-30
BIRC5	ENSG0000	6.81	0.505	2.376	1.57E-54
CENPM	ENSG0000	5.97	0.33	2.39	8.75E-58
HULC	ENSG0000	307.319	57.466	2.399	1.54E-20
HLA-H	ENSG0000	36.931	6.175	2.402	1.66E-34
IFI27	ENSG0000	145.209	26.64	2.403	3.73E-19
RP5-890E	ENSG0000	4.32	0	2.411	2.20E-14
RP11-334	ENSG0000	6.87	0.46	2.43	1.24E-44
TK1	ENSG0000	17.25	2.335	2.452	2.99E-58
GBA	ENSG0000	73.48	12.61	2.452	#######
CDK1	ENSG0000	6.32	0.33	2.46	7.38E-61
PRC1	ENSG0000	10.68	1.12	2.462	3.32E-64
CENPF	ENSG0000	6.14	0.295	2.463	1.12E-53
RP11-452	ENSG0000	37.569	5.96	2.47	9.36E-52
NQO1	ENSG0000	11.72	1.285	2.477	2.02E-29
EEF1A2	ENSG0000	7.09	0.45	2.48	9.75E-14
MUC13	ENSG0000	5.82	0.22	2.483	6.99E-25
FTH1P20	ENSG0000	13.07	1.48	2.504	1.81E-84
VWF	ENSG0000	14.41	1.715	2.505	7.16E-57
ZWINT	ENSG0000	9.69	0.865	2.519	7.03E-58
CD34	ENSG0000	14.17	1.625	2.531	8.03E-94
UBE2T	ENSG0000	8.81	0.695	2.533	9.85E-75
TROAP	ENSG0000	6.69	0.325	2.537	3.39E-57
CAPG	ENSG0000	26.6	3.725	2.546	7.53E-36
CXCL10	ENSG0000	11.72	1.145	2.568	3.36E-23
LCN2	ENSG0000	38.101	5.525	2.583	1.58E-13
ALG1L	ENSG0000	7.27	0.325	2.642	9.46E-43

MIR4435-.	ENSG0000	33.1	4.45	2.645	4.40E-53
HSPB1P1	ENSG0000	5.31	0	2.658	1.66E-49
RRM2	ENSG0000	9.26	0.595	2.685	1.36E-50
TSPAN8	ENSG0000	52.008	7.16	2.7	2.31E-22
LINC0015.	ENSG0000	35.26	4.54	2.711	1.71E-59
PDZK1IP1	ENSG0000	15.74	1.505	2.74	1.12E-16
SFN	ENSG0000	9.74	0.605	2.742	1.61E-31
CD24	ENSG0000	50.85	6.745	2.743	5.43E-13
RP5-940J.	ENSG0000	20.359	2.015	2.825	1.22E-06
THY1	ENSG0000	20.42	1.995	2.838	1.97E-62
TOP2A	ENSG0000	9.54	0.405	2.907	3.35E-65
SPINK1	ENSG0000	187.155	23.855	2.92	1.47E-06
FTH1P7	ENSG0000	25.5	2.38	2.971	2.02E-72
LYZ	ENSG0000	81.413	9.505	2.972	1.14E-24
CDC20	ENSG0000	9.32	0.31	2.978	3.93E-63
STMN1	ENSG0000	56.668	6.28	2.986	1.65E-75
SPP1	ENSG0000	75.378	8.524	3.004	2.34E-15
CDKN3	ENSG0000	10.96	0.45	3.044	4.14E-74
CCNB1	ENSG0000	14.8	0.9	3.056	1.24E-71
UBE2C	ENSG0000	13.24	0.6	3.154	1.56E-68
CDKN2A	ENSG0000	10.79	0.315	3.164	9.92E-63
CCL20	ENSG0000	22.42	1.5	3.228	7.05E-19
PIGY	ENSG0000	8.94	0.005	3.306	6.41E-13
ACSL4	ENSG0000	33.02	2.355	3.342	4.63E-30
PTTG1	ENSG0000	20.589	0.87	3.529	5.59E-80
OR2I1P	ENSG0000	25.71	1.305	3.535	2.25E-34
PLVAP	ENSG0000	43.03	2.464	3.668	#######
MTND4P1	ENSG0000	39.65	0.81	4.489	5.49E-17
MDK	ENSG0000	132.339	3.805	4.794	7.57E-81
UBD	ENSG0000	334.949	7.468	5.31	1.60E-66
AKR1B10	ENSG0000	177.158	3.015	5.472	9.84E-30
GPC3	ENSG0000	148.158	0.92	6.28	6.38E-59
RP11-40C	ENSG0000	389.91	0	8.611	3.30E-78
