# Supplementary material for: Mitochondrial-Related Transcriptome Feature Correlates with Prognosis, Vascular Invasion, Tumor Microenvironment, and Treatment Response in Hepatocellular Carcinoma
Source: Oxid Med Cell Longev. 2022 Apr 30;2022:1592905. doi: 10.1155/2022/1592905 (PMC9078845; doi:10.1155/2022/1592905)
Supplement: Supplementary Materials — Supplementary Figure 1: the Kaplan-Meier curves between high- and low-risk patients, in VI group (A) or none-VI group (B). Supplementary Figure 2: the ROC curves of each involved NMRG and prognosis_score for overall survival (OS) at 1 (A), 3 (B), and 5 years (C). Supplementary Figure 3:the differentially expressed genes between high- and low-risk groups. Supplementary Figure 4: the differentially expressed genes between nonresponder and responder groups. Supplementary Figure 5: the intersections of significantly upregulated genes (A) and downregulated genes (B) between low-risk patients and responder patients. The upregulated pathways potentially targeted by sorafenib via HALLMARK (C) and KEGG (D) enrichment analysis. The downregulated pathways potentially targeted by sorafenib via HALLMARK (E) and KEGG (F) enrichment analysis. Supplementary Figure 6: the evaluation of chemodrug treatment response between non-VI, micro-VI, and macro-VI groups. Supplementary Figure 7: The evaluation of chemodrug treatment response between high- and low-risk patients in the non-VI group. Supplementary Figure 8: the evaluation of chemodrug treatment response between high- and low-risk patients in the micro-VI group. Supplementary Figure 9: the evaluation of chemodrug treatment response between high- and low-risk patients in the macro-VI group. Supplementary Table 1: differentially expressed genes between tumor and normal tissues. Supplementary Table 2: coefficients of each NMRG involved in the NMRG signature. Supplementary Table 3: Top 20 high prevalence of altered genes in the high-risk group. Supplementary Table 4: Top 20 high prevalence of altered genes in the low-risk group. Supplementary Table: 5. the prevalence of a total of 61 genes was significantly different between high- and low-risk groups. [file 1592905.f1.zip › supplementary table 1C.pdf]

Supplementary Table 1C. Differentially downregulated genes in tumor tissues.

Gene Syml	Gene ID	Median (Tu	Median (N	Log2(Fold	adjp
SLC27A5	ENSG0000	248.413	497.947	-1.003	5.86E-10
AGAP5	ENSG0000	1.11	3.235	-1.005	3.29E-11
DTX4	ENSG0000	3.77	8.57	-1.005	1.15E-10
CD5L	ENSG0000	1.35	3.725	-1.008	2.12E-11
HPX	ENSG0000	1194.589	2403.131	-1.008	3.35E-11
AP000355.	ENSG0000	0.63	2.28	-1.009	1.62E-16
KCNK5	ENSG0000	1.67	4.375	-1.009	3.47E-14
TAF1C	ENSG0000	6.94	14.985	-1.01	1.46E-07
SIK1	ENSG0000	1.57	4.18	-1.011	6.29E-07
RP11-510.	ENSG0000	0.27	1.56	-1.011	8.48E-13
MYO7A	ENSG0000	4.79	10.68	-1.012	2.15E-13
FAM150B	ENSG0000	0.13	1.28	-1.013	5.92E-11
IGKC	ENSG0000	145.713	295.005	-1.013	2.58E-03
RP11-715.	ENSG0000	0	1.02	-1.014	1.02E-18
AGAP4	ENSG0000	2.28	5.63	-1.015	5.30E-08
LINC0096	ENSG0000	15.97	33.294	-1.015	1.63E-03
UBAP1L	ENSG0000	0.45	1.93	-1.015	5.99E-24
RUFY3	ENSG0000	6.88	14.95	-1.017	7.58E-15
SLC10A1	ENSG0000	33.819	69.567	-1.019	2.91E-07
RPS12P21	ENSG0000	0.07	1.17	-1.02	2.74E-46
RP11-394.	ENSG0000	0.23	1.495	-1.02	2.61E-32
PODN	ENSG0000	1.39	3.865	-1.025	7.70E-11
MT-CO2	ENSG0000	13276.62	27014.57	-1.025	1.28E-24
SULT1B1	ENSG0000	1.05	3.175	-1.026	2.81E-14
LY6G5B	ENSG0000	1.49	4.07	-1.026	3.60E-05
IGKV3-11	ENSG0000	8.89	19.145	-1.026	2.15E-03
ACOT12	ENSG0000	13.96	29.518	-1.029	2.17E-12
RP11-458.	ENSG0000	1.31	3.715	-1.029	1.74E-12
RP11-286.	ENSG0000	0.06	1.165	-1.03	5.25E-29
ZNF160	ENSG0000	1.91	4.945	-1.031	1.05E-15
ADRA2B	ENSG0000	0.41	1.885	-1.033	4.15E-50
DPH1	ENSG0000	10.36	22.265	-1.034	1.87E-17
TPT1-AS1	ENSG0000	3.42	8.05	-1.034	1.23E-12
GBP7	ENSG0000	6.71	14.798	-1.035	7.89E-07
DDX3Y	ENSG0000	3.68	8.605	-1.037	3.27E-10
TENM1	ENSG0000	0.12	1.3	-1.038	5.58E-26
IL4R	ENSG0000	17.09	36.155	-1.038	2.59E-17
FOSB	ENSG0000	1.62	4.38	-1.038	3.59E-11
CACNB2	ENSG0000	0.74	2.575	-1.039	1.27E-15
COX6B1P4	ENSG0000	0	1.055	-1.039	8.51E-76
PTGIS	ENSG0000	0.37	1.815	-1.039	1.91E-16
SMUG1P1	ENSG0000	0	1.055	-1.039	1.88E-58
CROCCP2	ENSG0000	2.91	7.045	-1.041	5.78E-21
CTB-89H1	ENSG0000	1.4	3.94	-1.041	5.23E-13
SLC39A5	ENSG0000	52.399	108.891	-1.041	3.77E-11
CDA	ENSG0000	15.42	32.819	-1.042	2.27E-14
FNBP4	ENSG0000	9.22	20.065	-1.043	2.32E-05
CCBE1	ENSG0000	0.06	1.185	-1.044	3.49E-54
ACAP3	ENSG0000	7.86	17.3	-1.046	7.95E-04
IGHV3-15	ENSG0000	0.45	1.995	-1.046	6.26E-03
RP11-34P.	ENSG0000	0.85	2.82	-1.046	5.09E-08
MT-ND1	ENSG0000	5574.782	11534.36	-1.049	3.52E-24
CES3	ENSG0000	7.32	16.224	-1.05	1.23E-13
IGLC3	ENSG0000	24.69	52.18	-1.05	1.49E-03
FANCC	ENSG0000	2.86	7.004	-1.052	1.14E-24
TMEM184.	ENSG0000	5.88	13.27	-1.052	3.29E-07
EVL	ENSG0000	25.86	54.749	-1.054	8.84E-15

COX6A2	ENSG0000	2.46	6.19	-1.055	4.48E-09
CPED1	ENSG0000	3.01	7.33	-1.055	2.93E-23
ETFDH	ENSG0000	27.81	58.846	-1.055	2.86E-33
MT-ND3	ENSG0000	9254.247	19245.72	-1.056	5.56E-24
MTND2P2	ENSG0000	211.541	441.922	-1.059	2.46E-12
SPAG5-AS	ENSG0000	0.5	2.125	-1.059	1.73E-24
RP11-423	ENSG0000	0.19	1.485	-1.062	2.96E-15
LINC0132	ENSG0000	0.46	2.05	-1.063	1.73E-09
LINC0010	ENSG0000	0.56	2.26	-1.063	1.09E-12
SLC46A3	ENSG0000	11.35	23.705	-1.063	1.20E-15
ASPDH	ENSG0000	33.751	71.634	-1.064	4.55E-11
RSRP1	ENSG0000	16.95	36.53	-1.064	9.46E-03
SCARNA1	ENSG0000	0.37	1.865	-1.064	5.67E-19
SLC3A1	ENSG0000	0.66	2.48	-1.068	4.67E-03
SLC11A1	ENSG0000	0.59	2.335	-1.069	5.08E-20
IGKV1-27	ENSG0000	0.4	1.94	-1.07	5.39E-03
ADAT2	ENSG0000	1.23	3.685	-1.071	1.97E-11
MRC1	ENSG0000	1.92	5.135	-1.071	2.95E-07
FGB	ENSG0000	3258.516	6850.767	-1.072	1.51E-09
ACCS	ENSG0000	6.22	14.189	-1.073	6.11E-12
AGER	ENSG0000	1.41	4.07	-1.073	5.63E-10
RP4-568C	ENSG0000	1.02	3.25	-1.073	3.07E-10
NDRG2	ENSG0000	83.239	176.349	-1.074	4.03E-23
AMN	ENSG0000	9.84	21.844	-1.075	1.73E-06
TPRG1	ENSG0000	1.7	4.69	-1.075	2.30E-23
IGHV5-51	ENSG0000	0.64	2.455	-1.075	1.92E-03
ENGASE	ENSG0000	5.9	13.544	-1.076	1.47E-04
RP11-119	ENSG0000	4.61	10.829	-1.076	2.11E-05
VNN1	ENSG0000	17.96	39.013	-1.077	3.43E-09
RP11-347	ENSG0000	0.37	1.89	-1.077	4.93E-25
CYP3A5	ENSG0000	100.148	212.607	-1.078	1.23E-05
C1R	ENSG0000	414.692	877.009	-1.079	1.34E-22
PNN	ENSG0000	14.91	32.62	-1.079	5.10E-11
LILRB5	ENSG0000	1.45	4.175	-1.079	5.91E-27
LTB4R	ENSG0000	2.01	5.365	-1.08	3.40E-08
RP11-384	ENSG0000	1.46	4.205	-1.081	2.87E-23
SORL1	ENSG0000	6.55	15	-1.084	1.74E-23
ADAMTS1	ENSG0000	3.15	7.81	-1.086	8.91E-16
CDK11A	ENSG0000	11.85	26.269	-1.086	7.63E-07
EBF4	ENSG0000	2.55	6.54	-1.087	8.38E-07
SLC25A18	ENSG0000	21.47	46.775	-1.088	1.48E-13
ARGLU1	ENSG0000	17.771	38.953	-1.09	4.10E-06
SH3BP5-A	ENSG0000	0.9	3.05	-1.092	1.24E-18
FGL1	ENSG0000	714.652	1526.116	-1.093	5.42E-08
SLX1B-SU	ENSG0000	0.99	3.245	-1.093	4.32E-11
AUTS2	ENSG0000	3.86	9.375	-1.094	2.28E-16
TGM2	ENSG0000	49.931	107.727	-1.094	3.91E-12
AP006285	ENSG0000	0.06	1.265	-1.095	2.02E-45
GRAMD1C	ENSG0000	2.24	5.925	-1.096	1.36E-19
IGKV1-9	ENSG0000	0.57	2.357	-1.097	1.74E-03
SRRM2	ENSG0000	123.442	265.349	-1.098	9.48E-06
PLXNB1	ENSG0000	30.89	67.29	-1.099	9.51E-09
AMDHD1	ENSG0000	23.48	51.479	-1.1	6.39E-15
AZGP1	ENSG0000	569.518	1221.805	-1.1	5.97E-16
HAPLN4	ENSG0000	0.31	1.81	-1.101	1.90E-25
RP13-516	ENSG0000	0.38	1.96	-1.101	1.50E-25
CITED2	ENSG0000	16.549	36.75	-1.105	3.51E-15
FPR1	ENSG0000	0.53	2.29	-1.105	7.81E-18

IGHV3-33	ENSG0000	0.74	2.745	-1.106	4.21E-03
TMEM25	ENSG0000	2.85	7.29	-1.107	1.37E-21
IL1RL1	ENSG0000	0.08	1.33	-1.109	2.80E-34
RP11-258	ENSG0000	0.23	1.655	-1.11	9.13E-37
RNF152	ENSG0000	2.87	7.37	-1.113	7.43E-30
ARHGAP1	ENSG0000	1.86	5.19	-1.114	4.07E-22
CCL21	ENSG0000	7.73	17.895	-1.114	1.21E-05
TRPV4	ENSG0000	0.77	2.83	-1.114	2.01E-14
APOA5	ENSG0000	97.267	211.834	-1.115	5.78E-13
SERPINE1	ENSG0000	20.831	46.29	-1.115	3.42E-05
VEGFA	ENSG0000	53.05	116.073	-1.115	3.43E-07
PRSS1	ENSG0000	0	1.17	-1.118	6.84E-25
KAZN	ENSG0000	0.34	1.91	-1.119	1.53E-33
MT-ND5	ENSG0000	1410.997	3065.91	-1.119	9.28E-17
MCC	ENSG0000	1.22	3.825	-1.12	2.64E-38
OGDHL	ENSG0000	18.45	41.286	-1.12	1.77E-17
AKR1C8P	ENSG0000	0.26	1.74	-1.121	2.75E-20
C1RL-AS1	ENSG0000	1.81	5.113	-1.121	6.51E-12
CLASRP	ENSG0000	20.34	45.469	-1.123	2.99E-03
BCL6	ENSG0000	10.78	24.681	-1.124	5.88E-13
BCHE	ENSG0000	17.321	38.95	-1.125	8.65E-13
CIDEB	ENSG0000	62.721	137.997	-1.125	1.22E-23
RNF165	ENSG0000	0.16	1.53	-1.125	1.29E-60
GABBR1	ENSG0000	0.68	2.665	-1.125	3.18E-12
RP11-496	ENSG0000	0.65	2.6	-1.125	9.34E-08
SPTBN2	ENSG0000	8.04	18.735	-1.126	2.26E-16
WASH5P	ENSG0000	5.2	12.53	-1.126	3.52E-05
PRSS2	ENSG0000	0.03	1.25	-1.127	1.60E-22
C14orf180	ENSG0000	0.01	1.21	-1.13	2.10E-53
MAT1A	ENSG0000	220.907	484.649	-1.13	7.57E-19
TRPM8	ENSG0000	4.06	10.085	-1.131	5.06E-12
RP11-42C	ENSG0000	4.66	11.395	-1.131	6.70E-13
EPHX2	ENSG0000	47.891	106.244	-1.133	3.89E-18
SLAIN1	ENSG0000	0.63	2.575	-1.133	7.08E-22
AFG3L1P	ENSG0000	3.2	8.22	-1.134	2.33E-10
ADRA1B	ENSG0000	1.21	3.855	-1.135	8.39E-31
N4BP2L1	ENSG0000	12.22	28.05	-1.136	5.56E-34
CYP21A1P	ENSG0000	2.25	6.15	-1.137	7.39E-04
HEPACAM	ENSG0000	0.07	1.355	-1.138	2.67E-04
NDUFA6-	ENSG0000	5.28	12.819	-1.138	9.18E-15
PPP1R3B	ENSG0000	10.71	24.785	-1.139	9.18E-19
ANK3	ENSG0000	1.01	3.435	-1.142	6.39E-15
PALM3	ENSG0000	7.61	18.025	-1.144	2.18E-17
RASGEF1B	ENSG0000	4.63	11.45	-1.145	8.14E-25
SNRPGP1	ENSG0000	0.25	1.765	-1.145	4.66E-19
RP11-609	ENSG0000	1.08	3.6	-1.145	9.69E-08
CYP3A43	ENSG0000	0.75	2.875	-1.147	1.02E-13
PLSCR4	ENSG0000	5.39	13.155	-1.147	8.18E-31
AC009120	ENSG0000	1.99	5.625	-1.148	4.51E-07
IGHV2-5	ENSG0000	0.23	1.725	-1.148	3.21E-04
MALAT1	ENSG0000	13.79	31.885	-1.153	4.83E-04
RP11-203	ENSG0000	1.67	4.945	-1.155	4.29E-11
KRT17P4	ENSG0000	0.05	1.34	-1.156	1.80E-35
PLCH2	ENSG0000	0.57	2.5	-1.157	6.00E-03
CPEB3	ENSG0000	1.11	3.71	-1.158	7.09E-40
NAMPT	ENSG0000	38.001	86.288	-1.162	1.32E-14
RND3	ENSG0000	9.27	21.98	-1.162	3.53E-24
SKAP1	ENSG0000	7.37	17.729	-1.162	9.64E-12

SYT9	ENSG0000	0.01	1.26	-1.162	8.39E-44
LRP5L	ENSG0000	2.81	7.535	-1.164	3.77E-13
IGKV2-24	ENSG0000	0.37	2.074	-1.166	5.29E-08
NEIL1	ENSG0000	8.76	20.924	-1.168	1.85E-11
MT-ND2	ENSG0000	6531.958	14680.36	-1.168	2.13E-23
SATB1	ENSG0000	1.65	4.965	-1.17	2.49E-22
CBFA2T3	ENSG0000	0.57	2.535	-1.171	1.29E-39
RP11-164.	ENSG0000	0.77	2.985	-1.171	1.98E-22
GOLGA8N	ENSG0000	1.07	3.665	-1.172	2.69E-20
RP11-394	ENSG0000	0.48	2.335	-1.172	1.21E-17
FAM160B2	ENSG0000	9.28	22.195	-1.174	1.12E-07
FAM151A	ENSG0000	0.45	2.274	-1.175	1.09E-05
PNPLA7	ENSG0000	1.7	5.1	-1.176	7.03E-45
PHYHD1	ENSG0000	14.71	34.515	-1.177	9.58E-19
PHYKPL	ENSG0000	20.569	47.765	-1.177	1.10E-27
LINC0017	ENSG0000	1.05	3.635	-1.177	4.56E-19
PAMR1	ENSG0000	0.7	2.855	-1.181	6.04E-24
AC004540	ENSG0000	0.17	1.654	-1.182	1.94E-25
FNDC4	ENSG0000	22.841	53.096	-1.182	9.26E-18
MT-CYB	ENSG0000	6751.535	15334.04	-1.183	2.38E-28
STAG3L5P	ENSG0000	5.22	13.125	-1.183	5.08E-08
TRIM66	ENSG0000	0.84	3.18	-1.184	1.30E-17
ADGRA3	ENSG0000	8.9	21.525	-1.186	8.54E-24
GGT5	ENSG0000	8.17	19.87	-1.186	5.36E-16
GPM6A	ENSG0000	0.16	1.64	-1.186	9.84E-45
SEC14L4	ENSG0000	2.04	5.92	-1.187	1.11E-14
RP4-669L.	ENSG0000	3.09	8.316	-1.188	1.19E-09
GADD45B	ENSG0000	66.472	152.783	-1.189	2.93E-20
YJEFN3	ENSG0000	0.55	2.535	-1.189	4.56E-10
RP11-817.	ENSG0000	0.04	1.375	-1.191	3.70E-21
UNC13D	ENSG0000	1.63	5.01	-1.192	1.65E-13
MST1	ENSG0000	205.486	472.132	-1.196	7.93E-24
DOCK5	ENSG0000	2.04	5.98	-1.199	5.82E-19
EGR1	ENSG0000	18.64	44.085	-1.199	1.97E-11
RP11-274	ENSG0000	4.96	12.679	-1.199	4.58E-10
ADAMTS2	ENSG0000	1.37	4.445	-1.2	2.95E-16
RP11-830	ENSG0000	0.04	1.39	-1.2	2.41E-51
PBLD	ENSG0000	29.59	69.384	-1.202	6.28E-19
KCND3	ENSG0000	0.84	3.235	-1.203	7.35E-28
CCDC84	ENSG0000	6.33	15.905	-1.206	2.85E-05
UGT2B17	ENSG0000	0.75	3.04	-1.207	1.79E-03
HK3	ENSG0000	1.21	4.105	-1.208	1.17E-20
GOLGA6L	ENSG0000	0.93	3.465	-1.21	1.80E-18
SPRR3	ENSG0000	0	1.315	-1.211	5.13E-29
NPY1R	ENSG0000	0.4	2.24	-1.211	1.79E-37
EPOR	ENSG0000	2.57	7.27	-1.212	1.33E-17
PLA2G4B	ENSG0000	1.46	4.7	-1.212	7.46E-13
RP11-250	ENSG0000	0.5	2.48	-1.214	3.11E-40
BGN	ENSG0000	56.79	133.176	-1.215	1.42E-13
C19orf66	ENSG0000	42.679	100.513	-1.217	3.13E-30
IGLC2	ENSG0000	39.769	93.769	-1.217	5.45E-03
PTOV1-AS	ENSG0000	2.39	6.895	-1.22	4.51E-08
CYP21A2	ENSG0000	5.8	14.85	-1.221	6.93E-07
TSLP	ENSG0000	0.33	2.1	-1.221	2.45E-42
HHIP	ENSG0000	0.06	1.475	-1.223	2.21E-43
IGF1	ENSG0000	4.77	12.475	-1.224	9.57E-14
MTHFD2L	ENSG0000	3.57	9.69	-1.226	7.44E-37
MUC3A	ENSG0000	1.62	5.13	-1.226	5.18E-04

ALDH8A1	ENSG0000	27.029	64.679	-1.229	3.10E-22
ZFP36	ENSG0000	53.742	127.304	-1.229	2.91E-21
SAMD5	ENSG0000	0.48	2.47	-1.229	5.79E-26
GOLGA8B	ENSG0000	7.7	19.393	-1.229	3.83E-03
DEFA3	ENSG0000	0	1.345	-1.23	2.14E-31
PEMT	ENSG0000	50.53	119.908	-1.23	5.18E-26
C8A	ENSG0000	73.338	173.446	-1.231	4.02E-19
ERRFI1	ENSG0000	67.672	160.379	-1.233	7.82E-19
FAM229A	ENSG0000	1.52	4.93	-1.235	6.43E-12
RP11-256	ENSG0000	0.83	3.32	-1.239	5.22E-20
PRELP	ENSG0000	1.25	4.32	-1.241	4.30E-16
GCH1	ENSG0000	11.76	29.19	-1.242	1.20E-26
RP11-676	ENSG0000	0.11	1.625	-1.242	6.65E-29
uc_338	ENSG0000	0	1.365	-1.242	4.08E-27
AF064858.	ENSG0000	0.81	3.285	-1.243	3.50E-13
BCL3	ENSG0000	31.12	75.011	-1.243	2.34E-15
B3GAT1	ENSG0000	0.11	1.63	-1.245	5.52E-11
AKAP17A	ENSG0000	10.16	25.474	-1.246	3.02E-09
ATF3	ENSG0000	14.94	36.796	-1.246	2.39E-11
BMPER	ENSG0000	0.06	1.515	-1.246	1.97E-87
JCHAIN	ENSG0000	2.83	8.089	-1.247	1.53E-07
RP11-166	ENSG0000	0.87	3.453	-1.252	1.57E-17
SNRPGP1	ENSG0000	1.81	5.694	-1.252	3.22E-15
MT-ND4	ENSG0000	9684.084	23104.72	-1.254	1.08E-34
EXOC3L4	ENSG0000	17.59	43.365	-1.255	2.97E-16
LINC0065	ENSG0000	1.39	4.703	-1.255	2.20E-14
MZF1	ENSG0000	4.09	11.175	-1.258	1.24E-10
SERPINA3	ENSG0000	370.748	888.913	-1.259	9.53E-13
ACADVL	ENSG0000	359.313	861.913	-1.26	2.35E-22
AP006285.	ENSG0000	0.47	2.525	-1.262	4.77E-13
NEU4	ENSG0000	9.57	24.385	-1.264	4.71E-12
RP11-390	ENSG0000	1.26	4.439	-1.267	5.45E-28
ABCC9	ENSG0000	2.25	6.825	-1.268	3.07E-26
CPT1B	ENSG0000	5.21	13.954	-1.268	5.88E-07
MT1P3	ENSG0000	0	1.41	-1.269	1.22E-09
LCN12	ENSG0000	6.83	17.9	-1.271	7.33E-14
AQP3	ENSG0000	24.599	60.915	-1.274	4.29E-21
OLFML3	ENSG0000	2.83	8.29	-1.278	3.70E-14
RSPO3	ENSG0000	0.06	1.57	-1.278	2.81E-43
PTPRS	ENSG0000	0.58	2.84	-1.281	8.57E-09
RP11-228	ENSG0000	0.38	2.354	-1.281	1.39E-25
GSDMB	ENSG0000	9.1	23.562	-1.282	5.33E-06
RP11-175	ENSG0000	1.41	4.86	-1.282	6.52E-12
LENG8	ENSG0000	19.07	47.91	-1.285	1.03E-05
IGLV2-8	ENSG0000	0.55	2.785	-1.288	3.27E-06
RP11-575	ENSG0000	0.44	2.52	-1.289	9.72E-23
LHX2	ENSG0000	0.36	2.33	-1.292	3.44E-33
ORM2	ENSG0000	1430.198	3502.573	-1.292	8.12E-14
RP1-232P.	ENSG0000	0.06	1.595	-1.292	1.26E-34
ACADL	ENSG0000	2.9	8.555	-1.293	3.27E-23
SLX1A-SU	ENSG0000	2.04	6.45	-1.293	6.72E-12
ACADS	ENSG0000	39.17	97.48	-1.294	1.55E-37
ADH1B	ENSG0000	360.135	884.396	-1.294	5.15E-11
DTX1	ENSG0000	4.03	11.335	-1.294	1.17E-21
PLGLB1	ENSG0000	29.8	74.586	-1.295	1.09E-20
WASH7P	ENSG0000	1.2	4.41	-1.298	1.11E-15
HSD11B1	ENSG0000	76.531	189.827	-1.299	4.75E-11
AGXT2	ENSG0000	13.63	35.03	-1.3	1.27E-23

WDR27	ENSG0000	2.94	8.715	-1.302	8.39E-10
RP11-159	ENSG0000	1	3.94	-1.305	8.45E-12
LSMEM1	ENSG0000	0.64	3.055	-1.306	5.49E-22
REC8	ENSG0000	2.28	7.14	-1.311	1.43E-14
IGHG4	ENSG0000	4.9	13.65	-1.312	2.88E-03
C8orf46	ENSG0000	1.62	5.51	-1.313	2.86E-14
BX842568.	ENSG0000	0.4	2.485	-1.316	3.16E-30
IGKV3-20	ENSG0000	6.35	17.302	-1.316	2.63E-03
CTC-529I	ENSG0000	0.66	3.14	-1.318	2.44E-37
RP11-43N	ENSG0000	0.69	3.215	-1.318	1.67E-22
IP6K3	ENSG0000	0.36	2.394	-1.319	3.14E-09
MAN1C1	ENSG0000	4.64	13.085	-1.32	1.14E-21
RP4-631H	ENSG0000	0	1.5	-1.322	1.76E-07
C6	ENSG0000	47.422	120.137	-1.323	5.41E-20
HOGA1	ENSG0000	7.79	21.02	-1.325	6.56E-23
CHST4	ENSG0000	0.03	1.585	-1.327	1.28E-18
CLDN10	ENSG0000	0.1	1.76	-1.327	6.13E-06
CACNA1H	ENSG0000	1.51	5.3	-1.328	2.70E-03
SMG1P7	ENSG0000	1.05	4.145	-1.328	1.69E-23
SPG20	ENSG0000	1.55	5.41	-1.33	4.11E-21
TIAF1	ENSG0000	0.48	2.72	-1.33	2.07E-39
COL27A1	ENSG0000	6.03	16.685	-1.331	4.27E-10
RP11-125	ENSG0000	0.32	2.325	-1.333	1.81E-26
CTD-2619	ENSG0000	1.95	6.439	-1.334	7.86E-19
PNISR	ENSG0000	11.81	31.325	-1.335	2.01E-08
ATHL1	ENSG0000	9.89	26.485	-1.336	7.22E-03
PDGFRA	ENSG0000	0.92	3.855	-1.338	3.43E-19
MUC6	ENSG0000	0.02	1.58	-1.339	2.73E-09
RP11-295	ENSG0000	3.58	10.585	-1.339	6.20E-08
COX7CP1	ENSG0000	0	1.532	-1.34	6.12E-13
RP11-196	ENSG0000	0.57	2.975	-1.34	2.77E-47
USP9Y	ENSG0000	0.4	2.55	-1.342	1.78E-14
FBP1	ENSG0000	116.403	296.902	-1.343	4.11E-20
NPC1L1	ENSG0000	4.68	13.435	-1.346	1.66E-15
LIPG	ENSG0000	8.01	21.904	-1.346	2.07E-20
RP11-632	ENSG0000	1.68	5.815	-1.346	2.31E-26
NKTR	ENSG0000	4.61	13.3	-1.35	1.96E-10
CYP2B7P	ENSG0000	5.24	14.939	-1.353	1.85E-03
TPPP2	ENSG0000	0.76	3.525	-1.362	1.01E-29
PGA4	ENSG0000	0.05	1.7	-1.362	3.57E-43
C1orf228	ENSG0000	1.79	6.188	-1.365	4.05E-27
AL161668.	ENSG0000	1.63	5.785	-1.367	3.53E-22
SLC25A37	ENSG0000	4.4	12.924	-1.367	8.04E-34
PPP1R3C	ENSG0000	10.36	28.32	-1.368	1.33E-15
KMO	ENSG0000	7.17	20.115	-1.37	7.00E-22
CCL23	ENSG0000	0.22	2.155	-1.371	1.11E-67
CTC-524C	ENSG0000	0.71	3.425	-1.372	4.47E-21
OAT	ENSG0000	9.78	26.915	-1.373	7.28E-09
HOOK2	ENSG0000	8.22	22.89	-1.374	5.02E-07
RIC3	ENSG0000	0.07	1.775	-1.375	1.08E-35
LPIN3	ENSG0000	4.75	13.924	-1.376	5.67E-11
HAL	ENSG0000	20.48	54.784	-1.377	3.89E-14
ACACB	ENSG0000	8.47	23.61	-1.378	2.85E-25
ABCA9	ENSG0000	0.63	3.27	-1.389	1.21E-33
RP11-115	ENSG0000	0.56	3.09	-1.391	1.34E-09
NPIPB3	ENSG0000	8.82	24.769	-1.392	1.05E-07
IGKV1D-3	ENSG0000	3.94	11.967	-1.392	4.79E-03
RP4-583P	ENSG0000	2.09	7.12	-1.394	1.02E-32

F11-AS1	ENSG0000	2.75	8.87	-1.396	2.92E-32
TNS2	ENSG0000	24.931	67.367	-1.399	2.42E-23
LIFR	ENSG0000	0.71	3.515	-1.401	4.82E-49
DIRAS3	ENSG0000	0.25	2.305	-1.403	1.29E-38
FAM193B	ENSG0000	17.13	46.951	-1.403	3.06E-04
IGLV2-11	ENSG0000	1.12	4.605	-1.403	9.43E-05
RP11-404	ENSG0000	0.35	2.57	-1.403	1.16E-22
SRSF11	ENSG0000	25.31	68.614	-1.404	8.39E-11
SLC13A5	ENSG0000	60.4	161.954	-1.408	6.58E-20
MLXIPL	ENSG0000	98.7	263.982	-1.41	2.76E-10
AC004538	ENSG0000	0.14	2.04	-1.415	9.74E-43
HPGD	ENSG0000	14.55	40.456	-1.415	6.59E-12
RP11-238	ENSG0000	0.14	2.04	-1.415	1.53E-04
CAMK2B	ENSG0000	0.24	2.31	-1.416	1.36E-26
NRG1	ENSG0000	0.45	2.87	-1.416	1.20E-10
CCL19	ENSG0000	2.7	8.89	-1.418	1.65E-06
GSTZ1	ENSG0000	29.67	81.055	-1.42	1.10E-26
IGHG2	ENSG0000	10.09	28.674	-1.42	3.72E-04
MTATP6P	ENSG0000	1387.526	3715.008	-1.42	1.04E-23
TMEM45A	ENSG0000	11.38	32.179	-1.422	4.51E-14
KIAA0895	ENSG0000	2.04	7.16	-1.424	1.66E-10
MFAP3L	ENSG0000	1.98	7.005	-1.426	9.41E-28
RP11-524	ENSG0000	0.37	2.685	-1.427	1.64E-41
ACSM5	ENSG0000	28.829	79.263	-1.428	1.12E-22
RP11-69E	ENSG0000	0.83	3.935	-1.431	4.93E-20
HERC2P2	ENSG0000	4.35	13.429	-1.431	8.98E-06
CCNL1	ENSG0000	21.84	60.633	-1.432	8.69E-22
GSTT2B	ENSG0000	3.38	10.82	-1.432	6.07E-07
IGLV3-25	ENSG0000	0.84	3.975	-1.435	9.68E-04
RCAN1	ENSG0000	17.179	48.371	-1.441	5.50E-43
TBX15	ENSG0000	2.27	7.885	-1.442	1.26E-14
IGKV1-5	ENSG0000	1.45	5.679	-1.447	3.65E-03
AZGP1P1	ENSG0000	6.43	19.275	-1.448	8.77E-28
STARD5	ENSG0000	6.45	19.359	-1.45	3.13E-42
STAG3L5P	ENSG0000	14.78	42.15	-1.451	4.67E-08
RP3-508I1	ENSG0000	0	1.745	-1.457	4.80E-18
CYP4A11	ENSG0000	108.217	299.559	-1.46	1.21E-15
RP1-102E	ENSG0000	0.27	2.495	-1.46	2.12E-46
CLDN2	ENSG0000	5.48	16.855	-1.462	1.92E-05
SULT1E1	ENSG0000	1.41	5.65	-1.464	1.08E-08
RP11-415	ENSG0000	0.74	3.8	-1.464	3.31E-31
CTD-2240	ENSG0000	0.3	2.59	-1.465	3.83E-54
COLEC11	ENSG0000	18.71	53.509	-1.468	2.49E-20
DUSP1	ENSG0000	76.452	213.619	-1.47	6.33E-21
SEC31B	ENSG0000	1.22	5.16	-1.472	4.60E-23
TDO2	ENSG0000	76.478	213.996	-1.472	1.71E-17
AKR7L	ENSG0000	4.43	14.072	-1.473	1.15E-28
PRSS8	ENSG0000	3	10.11	-1.474	3.16E-09
LRG1	ENSG0000	121.229	339.166	-1.477	1.29E-15
ABLIM3	ENSG0000	12.69	37.135	-1.478	3.05E-29
AC138035	ENSG0000	0.6	3.465	-1.481	7.14E-22
NR4A1	ENSG0000	8.57	25.707	-1.481	1.62E-17
IGLV1-51	ENSG0000	1.47	5.9	-1.482	1.29E-04
SOCS3	ENSG0000	6.84	20.916	-1.483	7.50E-13
TTTY14	ENSG0000	0.41	2.95	-1.486	2.34E-05
NTF3	ENSG0000	0.23	2.445	-1.486	1.37E-78
MBL2	ENSG0000	12.81	37.715	-1.487	4.36E-18
AFM	ENSG0000	50.38	143.334	-1.49	1.15E-19

IGKV2-28	ENSG0000	2.52	8.89	-1.49	2.38E-04
CNTN3	ENSG0000	0.08	2.035	-1.491	2.99E-40
ZC3H13	ENSG0000	4.81	15.4	-1.497	1.80E-30
RP11-499	ENSG0000	2.1	7.765	-1.499	3.20E-16
CD1D	ENSG0000	1.31	5.54	-1.501	6.84E-32
RP11-66N	ENSG0000	1.31	5.55	-1.504	6.60E-14
SMIM24	ENSG0000	1.55	6.257	-1.509	9.69E-03
MT-ATP6	ENSG0000	11214.67	31942.73	-1.51	1.16E-41
G0S2	ENSG0000	46.051	133.107	-1.511	9.21E-13
ASMTL-AS	ENSG0000	3.23	11.1	-1.516	3.17E-06
AASS	ENSG0000	3.24	11.16	-1.52	9.03E-20
SYT7	ENSG0000	6.73	21.18	-1.521	2.42E-13
CSAD	ENSG0000	14.78	44.387	-1.524	5.27E-29
DNAJC12	ENSG0000	6.66	21.085	-1.528	1.18E-18
GPD1	ENSG0000	15.39	46.359	-1.531	1.03E-17
KBTBD11	ENSG0000	0.26	2.645	-1.532	7.57E-57
MPPED1	ENSG0000	1.34	5.769	-1.532	5.30E-26
ID1	ENSG0000	15.63	47.12	-1.533	3.47E-20
CYP1A1	ENSG0000	0.97	4.71	-1.535	1.72E-04
MT-ND4L	ENSG0000	5341.391	15483.03	-1.535	6.04E-27
EFHD1	ENSG0000	5.26	17.155	-1.536	2.61E-09
SLC39A14	ENSG0000	54.681	160.457	-1.536	6.14E-37
GNMT	ENSG0000	30.38	90.336	-1.541	8.24E-10
IGHA2	ENSG0000	2.83	10.167	-1.544	5.71E-12
CTB-50L1	ENSG0000	1.12	5.205	-1.549	6.55E-24
HAND2-A	ENSG0000	0.12	2.285	-1.552	3.44E-58
IGHM	ENSG0000	8.76	27.634	-1.553	1.81E-10
TUBE1	ENSG0000	4.17	14.245	-1.56	2.56E-55
RASD1	ENSG0000	14.23	43.902	-1.56	8.63E-11
CTD-2619	ENSG0000	1.41	6.11	-1.561	1.97E-20
HPN-AS1	ENSG0000	0.59	3.69	-1.561	1.59E-26
C1orf168	ENSG0000	3.31	11.729	-1.562	6.25E-32
CTD-3092	ENSG0000	2.47	9.255	-1.563	2.53E-27
STEAP4	ENSG0000	0.77	4.24	-1.566	4.38E-36
PLGLB2	ENSG0000	65.348	195.78	-1.568	4.11E-23
FXYD1	ENSG0000	79.34	237.14	-1.568	2.63E-12
CBS	ENSG0000	4.02	13.905	-1.57	8.68E-05
SLC17A9	ENSG0000	29.769	90.333	-1.57	4.91E-07
RRN3P1	ENSG0000	1.24	5.655	-1.571	1.25E-33
ADAMTSL	ENSG0000	3.15	11.359	-1.574	3.48E-25
MAPK8IP3	ENSG0000	5.62	18.72	-1.575	5.30E-08
ALOX12P2	ENSG0000	1.39	6.128	-1.577	2.52E-25
CFP	ENSG0000	2.32	8.934	-1.581	3.12E-36
CCNL2	ENSG0000	33.029	101.006	-1.584	1.99E-10
IGKV1-33	ENSG0000	1.17	5.51	-1.585	1.25E-03
RP11-115	ENSG0000	1.93	7.789	-1.585	9.26E-04
CSRNP1	ENSG0000	7.19	23.779	-1.597	3.50E-31
MYOM2	ENSG0000	0.86	4.635	-1.599	2.62E-37
MT1XP1	ENSG0000	0	2.03	-1.599	2.90E-28
ACSM3	ENSG0000	18.45	58.215	-1.606	1.69E-24
C14orf105	ENSG0000	5.67	19.307	-1.606	5.36E-20
ACAD11	ENSG0000	12.74	40.844	-1.607	7.07E-44
AGAP6	ENSG0000	3.38	12.338	-1.607	1.98E-13
ANXA10	ENSG0000	10.59	34.34	-1.608	2.59E-18
NR1I2	ENSG0000	9.26	30.295	-1.609	2.22E-27
FAM134B	ENSG0000	2.32	9.124	-1.609	1.44E-17
STEAP3	ENSG0000	19.849	62.63	-1.61	3.11E-40
SLC7A2	ENSG0000	14.44	46.174	-1.611	3.88E-24

CNTFR	ENSG0000	0.49	3.555	-1.612	1.21E-06
SAA4	ENSG0000	251.479	770.846	-1.612	7.75E-21
LINC0101ε	ENSG0000	5.58	19.171	-1.616	8.20E-12
ECHDC2	ENSG0000	148.549	458.109	-1.618	1.73E-42
DNHD1	ENSG0000	1.45	6.529	-1.62	1.38E-17
RP11-361.	ENSG0000	20.53	65.36	-1.624	1.61E-03
GLYATL1	ENSG0000	44.609	140.035	-1.629	8.95E-25
SNX29P2	ENSG0000	0.59	3.925	-1.631	4.19E-40
KRTCAP3	ENSG0000	1.41	6.48	-1.634	1.47E-04
HP	ENSG0000	8497.954	26421.96	-1.636	2.98E-17
ACSL1	ENSG0000	129.984	406.58	-1.638	4.55E-31
CLRN3	ENSG0000	4	14.575	-1.639	1.60E-12
DES	ENSG0000	0.19	2.725	-1.646	3.52E-34
CTC-505C	ENSG0000	2.9	11.22	-1.648	1.94E-11
FAM163B	ENSG0000	0.09	2.415	-1.648	2.16E-47
CTB-79E8.	ENSG0000	0	2.14	-1.651	3.13E-21
ITIH4-AS1	ENSG0000	0.58	3.97	-1.653	4.22E-33
ADGRG7	ENSG0000	0.72	4.425	-1.657	4.94E-46
PILRB	ENSG0000	7.6	26.129	-1.657	3.78E-11
C4A-AS1	ENSG0000	2.72	10.738	-1.658	2.36E-24
C4B-AS1	ENSG0000	2.72	10.738	-1.658	2.36E-24
PDXDC2P	ENSG0000	2.91	11.375	-1.662	3.44E-20
LINC0100.	ENSG0000	3.89	14.567	-1.671	1.84E-11
IDO2	ENSG0000	0.11	2.54	-1.673	3.37E-33
F9	ENSG0000	42.659	138.457	-1.675	2.08E-23
LPAL2	ENSG0000	1.31	6.39	-1.678	4.47E-39
SLC28A1	ENSG0000	8.97	30.939	-1.68	7.70E-22
RP4-763G	ENSG0000	5.4	19.574	-1.685	9.76E-20
CYP4A22	ENSG0000	23.29	77.329	-1.689	1.47E-19
AC009963	ENSG0000	12.75	43.499	-1.694	7.81E-18
ABCA8	ENSG0000	3.33	13.07	-1.7	4.52E-35
CSF3R	ENSG0000	1.43	6.905	-1.702	3.81E-27
NPIPB4	ENSG0000	4.31	16.34	-1.707	4.23E-13
PGA3	ENSG0000	0.06	2.469	-1.71	8.52E-49
GHR	ENSG0000	7.3	26.178	-1.711	1.09E-29
IGFBP3	ENSG0000	49.428	164.471	-1.714	1.52E-24
IGLV1-40	ENSG0000	1.33	6.649	-1.715	1.26E-03
MT1DP	ENSG0000	0.59	4.235	-1.719	2.76E-18
ORM1	ENSG0000	4575.773	15121.36	-1.724	3.35E-19
PAN2	ENSG0000	13.25	46.264	-1.73	5.37E-17
WDR72	ENSG0000	1.77	8.21	-1.733	1.72E-23
BHMT	ENSG0000	58.582	197.176	-1.734	1.51E-11
CTD-3080	ENSG0000	0.44	3.79	-1.734	1.40E-29
CYP2D7	ENSG0000	19.04	65.65	-1.734	1.53E-12
CAPN3	ENSG0000	3.25	13.15	-1.735	2.72E-26
MBL1P	ENSG0000	0.22	3.06	-1.735	3.31E-49
DEFA1B	ENSG0000	0	2.35	-1.744	8.92E-47
BBOX1	ENSG0000	2.69	11.375	-1.746	1.48E-11
DHODH	ENSG0000	14.04	49.561	-1.749	3.99E-44
MFAP4	ENSG0000	3.46	14.005	-1.75	2.29E-16
IGSF9	ENSG0000	1.64	7.91	-1.755	1.31E-18
GYS2	ENSG0000	7.64	28.294	-1.761	1.57E-25
MAMDC4	ENSG0000	2.66	11.405	-1.761	4.27E-16
MT-ATP8	ENSG0000	11020.48	37358.53	-1.761	1.62E-29
INMT	ENSG0000	1.57	7.73	-1.764	6.84E-36
C1QTNF1	ENSG0000	4.54	17.865	-1.768	5.91E-25
IFITM10	ENSG0000	1.82	8.635	-1.773	3.62E-14
PROZ	ENSG0000	8.74	32.433	-1.779	3.48E-29

PLAC8	ENSG0000	1.03	5.975	-1.781	4.43E-28
DEFA1	ENSG0000	0.09	2.755	-1.784	7.86E-44
CYP2C9	ENSG0000	134.596	470.368	-1.798	1.33E-17
AKR1D1	ENSG0000	10.96	40.685	-1.801	1.01E-11
TAT	ENSG0000	98.25	345.525	-1.804	4.06E-16
GBA3	ENSG0000	8.22	31.277	-1.808	1.37E-18
SRD5A2	ENSG0000	1.17	6.605	-1.809	1.59E-20
PLIN5	ENSG0000	18.75	68.379	-1.813	8.12E-20
RP11-419	ENSG0000	2.46	11.16	-1.813	2.07E-18
EPO	ENSG0000	0.19	3.185	-1.814	6.95E-16
PLIN1	ENSG0000	1.84	8.99	-1.815	7.43E-29
C11orf96	ENSG0000	6.79	26.472	-1.818	1.04E-24
FITM1	ENSG0000	1.31	7.18	-1.824	3.99E-55
ADH4	ENSG0000	154.032	549.147	-1.827	2.35E-13
SRPX	ENSG0000	0.84	5.53	-1.827	1.85E-35
AC099668	ENSG0000	2.33	10.819	-1.828	3.09E-21
ALPL	ENSG0000	10.67	40.58	-1.833	1.71E-26
ESR1	ENSG0000	0.53	4.47	-1.838	3.35E-35
DBH-AS1	ENSG0000	5.1	20.855	-1.841	1.48E-21
FOLH1B	ENSG0000	0.43	4.13	-1.843	2.53E-28
IL1RAP	ENSG0000	6.41	25.615	-1.845	5.13E-43
LINC0023	ENSG0000	0.19	3.295	-1.852	2.37E-34
CYP8B1	ENSG0000	34.489	127.326	-1.854	2.61E-21
CYR61	ENSG0000	14.11	53.815	-1.859	8.70E-30
FAM180A	ENSG0000	0.22	3.43	-1.86	4.66E-57
TMEM82	ENSG0000	5.08	21.11	-1.863	2.33E-21
FAM198A	ENSG0000	0.36	3.95	-1.864	4.73E-34
RP11-328	ENSG0000	3.02	13.645	-1.865	2.86E-17
RP11-231	ENSG0000	6.94	27.937	-1.866	3.68E-16
ADCY1	ENSG0000	0.4	4.115	-1.869	5.43E-29
ATOH8	ENSG0000	4.42	18.95	-1.88	2.47E-30
AGAP9	ENSG0000	2.16	10.75	-1.895	3.89E-19
S100A12	ENSG0000	0.19	3.425	-1.895	2.35E-62
SLC25A27	ENSG0000	2.23	11.03	-1.897	7.15E-23
AC006128	ENSG0000	1.15	7.035	-1.902	1.11E-20
KRT13	ENSG0000	0	2.74	-1.903	6.91E-36
NBPF8	ENSG0000	2.65	12.719	-1.91	1.69E-33
RP11-622	ENSG0000	5.56	23.667	-1.911	1.49E-28
LINC0159	ENSG0000	0.99	6.505	-1.915	1.21E-14
XAF1	ENSG0000	7.13	29.705	-1.917	1.11E-22
CES4A	ENSG0000	1.93	10.081	-1.919	1.34E-22
DIO3OS	ENSG0000	0.41	4.344	-1.922	2.75E-26
GSTM2	ENSG0000	4.74	20.76	-1.923	4.67E-22
RP11-132	ENSG0000	1.1	6.998	-1.929	2.20E-45
CTD-2619	ENSG0000	1.39	8.145	-1.936	1.05E-25
FCGR2B	ENSG0000	1.64	9.1	-1.936	7.25E-22
GREM2	ENSG0000	0.93	6.41	-1.941	4.38E-16
GLYAT	ENSG0000	35.37	138.764	-1.942	3.66E-18
NEAT1	ENSG0000	124.507	481.719	-1.943	1.13E-11
MAGI2-A	ENSG0000	1.53	8.76	-1.948	1.65E-24
NGFR	ENSG0000	1.25	7.695	-1.95	2.04E-36
S100A9	ENSG0000	11.29	46.889	-1.962	1.32E-14
SLC6A13	ENSG0000	2.29	11.89	-1.97	1.19E-28
FAM99B	ENSG0000	0.23	3.835	-1.975	1.97E-44
CTD-2537	ENSG0000	1.46	8.73	-1.984	1.04E-19
FAM13A	ENSG0000	3.1	15.24	-1.986	1.03E-33
IGLV2-14	ENSG0000	2.14	11.483	-1.991	2.77E-06
NFKBIZ	ENSG0000	3.85	18.334	-1.995	1.07E-34

AC068535	ENSG0000	1.62	9.54	-2.008	3.12E-22
TMEM27	ENSG0000	1.03	7.24	-2.021	5.51E-33
OR10J6P	ENSG0000	0.62	5.62	-2.031	6.99E-38
TNFSF14	ENSG0000	2.84	14.71	-2.032	5.52E-16
FAM83A-A	ENSG0000	0.45	4.95	-2.037	2.15E-08
FLJ22763	ENSG0000	0.76	6.25	-2.042	3.65E-27
RP11-326	ENSG0000	0.96	7.133	-2.053	1.31E-07
STAB2	ENSG0000	0.08	3.505	-2.06	#######
AC104809	ENSG0000	0.04	3.375	-2.073	1.11E-34
C3P1	ENSG0000	11.36	51.089	-2.075	5.67E-42
LPA	ENSG0000	1.21	8.365	-2.083	6.63E-44
HGF	ENSG0000	0.79	6.615	-2.089	6.68E-35
MME	ENSG0000	0.91	7.135	-2.091	2.01E-06
KCNN2	ENSG0000	0.25	4.35	-2.098	1.91E-53
HAND2	ENSG0000	0.4	5.02	-2.104	1.16E-45
GCKR	ENSG0000	22.119	98.499	-2.106	1.31E-37
C8orf4	ENSG0000	12.81	58.63	-2.11	1.62E-36
CYP39A1	ENSG0000	3.25	17.415	-2.115	3.87E-28
LRCOL1	ENSG0000	1.04	7.855	-2.118	2.13E-29
AVPR1A	ENSG0000	0.47	5.405	-2.123	1.46E-24
LINC0134	ENSG0000	1.2	8.645	-2.132	5.14E-42
OIT3	ENSG0000	2.66	15.075	-2.135	1.80E-52
LYVE1	ENSG0000	0.83	7.055	-2.138	1.12E-41
AADAT	ENSG0000	1.83	11.5	-2.143	8.81E-52
CYP2A6	ENSG0000	132.862	592.285	-2.148	3.83E-11
RP4-564F	ENSG0000	0.54	5.865	-2.156	5.41E-43
PLIN4	ENSG0000	4.27	22.554	-2.16	1.51E-25
PLGLA	ENSG0000	4.32	22.95	-2.171	6.68E-46
RP3-342P	ENSG0000	0.1	3.96	-2.173	5.87E-47
PRSS53	ENSG0000	0.23	4.55	-2.174	2.46E-52
DPT	ENSG0000	0.55	6.09	-2.194	5.77E-25
NPIPB5	ENSG0000	9.71	48.205	-2.2	8.53E-16
LCAT	ENSG0000	32.421	153.649	-2.21	2.18E-42
CYP26A1	ENSG0000	0.28	4.96	-2.219	5.10E-42
ADAMTS1	ENSG0000	2.32	14.475	-2.221	1.49E-97
LY6E	ENSG0000	54.53	259.294	-2.229	6.44E-29
ANGPTL6	ENSG0000	1.67	11.6	-2.238	1.35E-96
IGHA1	ENSG0000	21.08	103.5	-2.243	3.01E-16
PCK1	ENSG0000	84.799	405.426	-2.244	1.54E-23
CYP2B6	ENSG0000	15.26	76.219	-2.248	3.21E-25
ENO3	ENSG0000	11.36	58.1	-2.258	4.36E-29
SFRP5	ENSG0000	0.05	4.03	-2.26	1.01E-24
RDH16	ENSG0000	39.609	193.762	-2.262	4.68E-32
SAA2-SAA	ENSG0000	2.75	17.052	-2.267	1.56E-11
CXCL12	ENSG0000	11.66	60.325	-2.276	7.53E-40
FOS	ENSG0000	15.56	80.208	-2.294	5.31E-30
RP11-6B4	ENSG0000	0.17	4.765	-2.301	2.21E-48
LINC0137	ENSG0000	0.53	6.555	-2.304	1.99E-14
INS-IGF2	ENSG0000	0	4.03	-2.331	9.78E-65
CYP2E1	ENSG0000	500.525	2526.544	-2.333	3.98E-21
CFHR3	ENSG0000	33.541	174.664	-2.346	3.33E-15
AC005077	ENSG0000	0.52	6.74	-2.348	2.36E-51
MT1A	ENSG0000	15.19	81.693	-2.353	1.74E-18
PZP	ENSG0000	0.18	5.14	-2.379	1.20E-55
HEPN1	ENSG0000	0.21	5.295	-2.379	5.40E-09
GNAO1	ENSG0000	0.68	7.76	-2.382	4.76E-52
RP11-290	ENSG0000	3.19	20.87	-2.384	1.44E-36
ATF5	ENSG0000	145.946	767.753	-2.387	6.51E-30

MTND4P2	ENSG0000	4	25.358	-2.398	3.83E-27
DNASE1L3	ENSG0000	6.64	39.306	-2.399	3.21E-36
ECM1	ENSG0000	2.51	17.755	-2.418	1.30E-63
MT1JP	ENSG0000	0.05	4.62	-2.42	1.61E-49
OXT	ENSG0000	0.13	5.293	-2.477	6.22E-25
RP11-434	ENSG0000	1.32	11.964	-2.482	2.64E-30
MST1L	ENSG0000	10.36	64.444	-2.526	5.43E-21
CCL14	ENSG0000	7.25	46.812	-2.535	1.04E-34
SDS	ENSG0000	58.318	343.496	-2.538	1.03E-11
GDF2	ENSG0000	0	4.84	-2.546	#######
CYP2C19	ENSG0000	0.33	6.81	-2.554	3.42E-45
LINC0084	ENSG0000	5.59	37.984	-2.565	5.09E-17
CYP2C8	ENSG0000	85.017	509.168	-2.568	5.79E-27
CDHR2	ENSG0000	1.82	16.165	-2.606	1.02E-28
APOF	ENSG0000	17.701	113.668	-2.616	2.62E-38
CXCL2	ENSG0000	18.181	119.784	-2.655	1.51E-45
DCN	ENSG0000	14.87	101.289	-2.688	1.21E-29
FAM65C	ENSG0000	0.33	7.74	-2.716	8.69E-74
NNMT	ENSG0000	269.342	1779.553	-2.719	8.31E-23
APOA4	ENSG0000	3.03	26.19	-2.754	1.79E-07
S100A8	ENSG0000	3.42	29.025	-2.764	1.36E-39
MST1P2	ENSG0000	9.95	74.23	-2.78	5.13E-30
CHRD	ENSG0000	9.55	72.098	-2.793	1.16E-30
KDM8	ENSG0000	5.66	45.724	-2.811	9.08E-45
HBA2	ENSG0000	22.15	162.612	-2.821	4.20E-38
MEG3	ENSG0000	2.21	22.203	-2.854	6.21E-17
MT1L	ENSG0000	5.34	45.263	-2.867	1.56E-33
C7	ENSG0000	5.88	49.485	-2.875	7.22E-31
DBH	ENSG0000	0.56	10.785	-2.917	5.04E-99
GLS2	ENSG0000	7.55	64.115	-2.929	2.16E-23
MOGAT2	ENSG0000	5.55	49.036	-2.933	1.71E-41
IGF2	ENSG0000	10.29	89.75	-3.007	3.53E-10
UROC1	ENSG0000	3.89	38.638	-3.019	9.88E-33
MT2P1	ENSG0000	0	7.11	-3.02	1.10E-64
VIPR1	ENSG0000	0.56	11.848	-3.042	4.67E-92
RNA5SP2	ENSG0000	0	7.302	-3.054	1.59E-19
PLA2G2A	ENSG0000	23.811	207.166	-3.069	5.77E-07
RP5-966N	ENSG0000	6.86	66.16	-3.095	4.32E-35
NAT2	ENSG0000	2.28	27.324	-3.11	1.07E-60
SLC22A1	ENSG0000	56.102	495.588	-3.12	2.77E-29
ASPG	ENSG0000	7.77	75.475	-3.124	8.33E-41
MT2A	ENSG0000	261.977	2324.572	-3.145	5.54E-41
HBA1	ENSG0000	10	98.975	-3.184	2.99E-47
MT1E	ENSG0000	86.228	797.532	-3.194	1.59E-32
HAO2	ENSG0000	11.13	111.712	-3.216	4.03E-33
CLEC1B	ENSG0000	0.03	8.658	-3.229	1.07E-97
BCO2	ENSG0000	2.42	31.444	-3.246	8.54E-76
HGFAC	ENSG0000	11.26	117.783	-3.276	5.22E-35
HSD17B13	ENSG0000	6.73	74.827	-3.294	2.68E-16
CHRNA4	ENSG0000	0.33	12.095	-3.3	9.74E-29
ADRA1A	ENSG0000	0.75	16.38	-3.312	2.62E-82
COLEC10	ENSG0000	0.39	12.865	-3.318	1.21E-96
HBB	ENSG0000	15.86	167.277	-3.319	2.55E-49
THRSP	ENSG0000	6.27	72.531	-3.338	4.00E-20
CYP2A7	ENSG0000	1.97	29.83	-3.376	7.48E-14
PTH1R	ENSG0000	1.32	23.575	-3.405	#######
GCGR	ENSG0000	4.47	57.661	-3.423	2.38E-39
SLCO1B3	ENSG0000	1.89	30.546	-3.448	4.57E-31

IGFALS	ENSG0000	2.83	40.84	-3.449	2.19E-47
MT1X	ENSG0000	145.29	1597.997	-3.45	8.62E-43
C9	ENSG0000	23.051	263.854	-3.461	3.80E-35
CRP	ENSG0000	251.671	2792.323	-3.467	1.62E-13
PVALB	ENSG0000	0.11	1.22	-3.471	2.77E-41
CRHBP	ENSG0000	0.56	16.829	-3.515	3.43E-75
CYP3A4	ENSG0000	31.139	386.278	-3.591	1.22E-16
CNDP1	ENSG0000	0.49	17.989	-3.672	1.82E-56
CXCL14	ENSG0000	0.19	14.215	-3.676	7.94E-78
TTC36	ENSG0000	4	63.26	-3.684	2.66E-42
SLC25A47	ENSG0000	13.85	213.567	-3.853	3.91E-45
MARCO	ENSG0000	0.39	19.3	-3.868	4.03E-61
SAA1	ENSG0000	287.157	4468.423	-3.955	3.44E-23
LINC0109[illegible]	ENSG0000	0.55	23.3	-3.971	3.78E-87
MFSD2A	ENSG0000	3.81	77.691	-4.032	5.52E-45
AC132217	ENSG0000	21.11	365.062	-4.049	3.36E-12
FAM99A	ENSG0000	1.1	37.31	-4.189	1.13E-52
CLEC4M	ENSG0000	0.02	18.949	-4.29	#######
FCN3	ENSG0000	2.32	67.3	-4.363	1.37E-68
CLEC4G	ENSG0000	0.12	23.634	-4.459	#######
SAA2	ENSG0000	105.36	2489.516	-4.549	4.99E-25
MT1F	ENSG0000	7	191.386	-4.588	6.81E-49
FCN2	ENSG0000	0.28	30.658	-4.628	#######
LINC0155[illegible]	ENSG0000	1.42	69.249	-4.859	4.99E-26
CYP1A2	ENSG0000	0.48	44.13	-4.93	3.50E-37
GSTM1	ENSG0000	1.28	70.601	-4.973	2.84E-03
MT1M	ENSG0000	1.78	112.018	-5.345	8.93E-60
MT1G	ENSG0000	40.209	2587.601	-5.973	3.31E-51
HAMP	ENSG0000	6.75	669.968	-6.436	3.63E-66
MT1H	ENSG0000	2.16	332.67	-6.722	2.39E-61
