# Supplementary material for: Mitochondrial-Related Transcriptome Feature Correlates with Prognosis, Vascular Invasion, Tumor Microenvironment, and Treatment Response in Hepatocellular Carcinoma
Source: Oxid Med Cell Longev. 2022 Apr 30;2022:1592905. doi: 10.1155/2022/1592905 (PMC9078845; doi:10.1155/2022/1592905)
Supplement: Supplementary Materials — Supplementary Figure 1: the Kaplan-Meier curves between high- and low-risk patients, in VI group (A) or none-VI group (B). Supplementary Figure 2: the ROC curves of each involved NMRG and prognosis_score for overall survival (OS) at 1 (A), 3 (B), and 5 years (C). Supplementary Figure 3:the differentially expressed genes between high- and low-risk groups. Supplementary Figure 4: the differentially expressed genes between nonresponder and responder groups. Supplementary Figure 5: the intersections of significantly upregulated genes (A) and downregulated genes (B) between low-risk patients and responder patients. The upregulated pathways potentially targeted by sorafenib via HALLMARK (C) and KEGG (D) enrichment analysis. The downregulated pathways potentially targeted by sorafenib via HALLMARK (E) and KEGG (F) enrichment analysis. Supplementary Figure 6: the evaluation of chemodrug treatment response between non-VI, micro-VI, and macro-VI groups. Supplementary Figure 7: The evaluation of chemodrug treatment response between high- and low-risk patients in the non-VI group. Supplementary Figure 8: the evaluation of chemodrug treatment response between high- and low-risk patients in the micro-VI group. Supplementary Figure 9: the evaluation of chemodrug treatment response between high- and low-risk patients in the macro-VI group. Supplementary Table 1: differentially expressed genes between tumor and normal tissues. Supplementary Table 2: coefficients of each NMRG involved in the NMRG signature. Supplementary Table 3: Top 20 high prevalence of altered genes in the high-risk group. Supplementary Table 4: Top 20 high prevalence of altered genes in the low-risk group. Supplementary Table: 5. the prevalence of a total of 61 genes was significantly different between high- and low-risk groups. [file 1592905.f1.zip › Supplementary Table 4.pdf]

Supplementary Table 4. Top 20 high prevalence of altered genes in the low-risk group

	Hugo_Sym	Frame_Shi	Frame_Shi	In_Frame_I	In_Frame_I	Missense_I	Nonsense_	Nonstop_I	Splice_Site	Translatior	total	MutatedSa	AlteredSar	Frequency
1	CTNNB1	0	0	2	0	57	0	0	0	0	59	57	57	32%
2	TTN	5	0	0	2	51	2	0	0	0	60	48	48	27%
3	TP53	3	1	1	0	16	2	0	4	0	27	26	26	15%
4	ALB	6	3	4	0	6	0	0	10	0	29	23	23	13%
5	PCLO	1	1	0	0	25	2	0	0	0	29	22	22	12%
6	MUC16	0	1	0	0	24	2	0	0	0	27	21	21	12%
7	APOB	6	5	1	0	13	0	0	0	0	25	21	21	12%
8	MT-ND5	0	0	0	0	18	2	0	0	0	20	18	18	10%
9	XIRP2	2	0	1	0	14	0	0	0	0	17	16	16	9%
10	CACNA1E	1	0	0	0	14	0	0	1	0	16	15	15	8%
11	USH2A	1	0	0	1	14	0	0	0	0	16	15	15	8%
12	RYR1	1	1	0	1	12	0	0	0	0	15	15	15	8%
13	KMT2D	4	0	0	0	11	1	0	0	0	16	14	14	8%
14	RYR2	1	0	0	0	14	1	0	0	0	16	14	14	8%
15	AHNAK2	0	0	0	0	15	0	0	0	0	15	14	14	8%
16	ARID2	2	1	0	0	6	4	0	2	0	15	14	14	8%
17	BAP1	1	0	1	0	6	5	0	2	0	15	13	13	7%
18	HMCN1	0	1	0	0	11	3	0	0	0	15	13	13	7%
19	OBSCN	0	0	0	0	15	0	0	0	0	15	13	13	7%
20	COL11A1	2	0	0	0	11	0	0	1	0	14	13	13	7%
21	MUC2	1	1	2	0	10	0	0	0	0	14	13	13	7%
22	ABCA13	0	0	0	0	12	0	0	1	0	13	13	13	7%
23	MT-CYB	0	0	0	0	13	0	0	0	0	13	13	13	7%
