# Supplementary material for: Mitochondrial-Related Transcriptome Feature Correlates with Prognosis, Vascular Invasion, Tumor Microenvironment, and Treatment Response in Hepatocellular Carcinoma
Source: Oxid Med Cell Longev. 2022 Apr 30;2022:1592905. doi: 10.1155/2022/1592905 (PMC9078845; doi:10.1155/2022/1592905)
Supplement: Supplementary Materials — Supplementary Figure 1: the Kaplan-Meier curves between high- and low-risk patients, in VI group (A) or none-VI group (B). Supplementary Figure 2: the ROC curves of each involved NMRG and prognosis_score for overall survival (OS) at 1 (A), 3 (B), and 5 years (C). Supplementary Figure 3:the differentially expressed genes between high- and low-risk groups. Supplementary Figure 4: the differentially expressed genes between nonresponder and responder groups. Supplementary Figure 5: the intersections of significantly upregulated genes (A) and downregulated genes (B) between low-risk patients and responder patients. The upregulated pathways potentially targeted by sorafenib via HALLMARK (C) and KEGG (D) enrichment analysis. The downregulated pathways potentially targeted by sorafenib via HALLMARK (E) and KEGG (F) enrichment analysis. Supplementary Figure 6: the evaluation of chemodrug treatment response between non-VI, micro-VI, and macro-VI groups. Supplementary Figure 7: The evaluation of chemodrug treatment response between high- and low-risk patients in the non-VI group. Supplementary Figure 8: the evaluation of chemodrug treatment response between high- and low-risk patients in the micro-VI group. Supplementary Figure 9: the evaluation of chemodrug treatment response between high- and low-risk patients in the macro-VI group. Supplementary Table 1: differentially expressed genes between tumor and normal tissues. Supplementary Table 2: coefficients of each NMRG involved in the NMRG signature. Supplementary Table 3: Top 20 high prevalence of altered genes in the high-risk group. Supplementary Table 4: Top 20 high prevalence of altered genes in the low-risk group. Supplementary Table: 5. the prevalence of a total of 61 genes was significantly different between high- and low-risk groups. [file 1592905.f1.zip › Supplementary Table 5.pdf]

Supplementary Table 5. The prevalence of a total of 61 genes were significantly different between high- and low- risk groups

	Hugo_Sym	High-risk (	Low-risk g	ci.up	ci.low	P value
1	TP53	83	26	8.536022	2.896458	7.91E-11
2	FAT3	20	5	14.94167	1.512559	0.002978
3	DNAH7	18	4	19.83139	1.532072	0.003319
4	MCTP2	8	0	Infinite	1.720825	0.007232
5	NLRP2	8	0	Infinite	1.720825	0.007232
6	SH3TC2	8	0	Infinite	1.720825	0.007232
7	MT-CO3	1	9	0.770499	0.002373	0.010168
8	DNAH3	10	1	450.6532	1.438091	0.010583
9	DNAH17	12	2	57.97021	1.35483	0.011364
10	MYO18B	12	2	57.97021	1.35483	0.011364
11	TENM4	12	2	57.97021	1.35483	0.011364
12	MAGEL2	0	6	0.822452	0	0.014238
13	PIK3C2G	0	6	0.822452	0	0.014238
14	C1orf173	7	0	Infinite	1.445844	0.014748
15	CSPG4	7	0	Infinite	1.445844	0.014748
16	DAO	7	0	Infinite	1.445844	0.014748
17	DMXL2	7	0	Infinite	1.445844	0.014748
18	ITGB2	7	0	Infinite	1.445844	0.014748
19	STK32B	7	0	Infinite	1.445844	0.014748
20	HSPG2	15	4	16.53531	1.214741	0.016307
21	KIAA1731	1	8	0.900342	0.002652	0.019179
22	NUP214	1	8	0.900342	0.002652	0.019179
23	SPHKAP	1	8	0.900342	0.002652	0.019179
24	TG	11	2	53.36261	1.211287	0.020238
25	CTNNB1	38	57	0.933158	0.340177	0.022905
26	LRP1B	22	9	6.590773	1.106331	0.023008
27	COL1A1	0	5	1.061601	0	0.02913
28	CREB3L3	0	5	1.061601	0	0.02913
29	DUOX2	0	5	1.061601	0	0.02913
30	FURIN	0	5	1.061601	0	0.02913
31	IL12A	0	5	1.061601	0	0.02913
32	KIAA1211	0	5	1.061601	0	0.02913
33	MCM2	0	5	1.061601	0	0.02913
34	QRFPR	0	5	1.061601	0	0.02913
35	SEL1L	0	5	1.061601	0	0.02913
36	SLK	0	5	1.061601	0	0.02913
37	TSHZ3	0	5	1.061601	0	0.02913
38	UMODL1	0	5	1.061601	0	0.02913
39	WNK1	0	5	1.061601	0	0.02913
40	ZNF800	0	5	1.061601	0	0.02913
41	CACNA1I	6	0	Infinite	1.175579	0.029991
42	CEP44	6	0	Infinite	1.175579	0.029991
43	EPHA6	6	0	Infinite	1.175579	0.029991
44	HHIPL2	6	0	Infinite	1.175579	0.029991
45	MAP4K5	6	0	Infinite	1.175579	0.029991
46	NCOA7	6	0	Infinite	1.175579	0.029991
47	PDE10A	6	0	Infinite	1.175579	0.029991
48	POTEH	6	0	Infinite	1.175579	0.029991
49	QRICH2	6	0	Infinite	1.175579	0.029991
50	SLC4A10	6	0	Infinite	1.175579	0.029991
51	SLCO6A1	6	0	Infinite	1.175579	0.029991
52	TGFBRAP1	6	0	Infinite	1.175579	0.029991
53	TSNARE1	6	0	Infinite	1.175579	0.029991
54	WIF1	6	0	Infinite	1.175579	0.029991
55	ACVR2A	2	9	1.038442	0.021849	0.034531
56	FCGBP	10	2	48.77841	1.069866	0.035557
57	PLXNA4	10	2	48.77841	1.069866	0.035557

58	ADAMTS9	1	7	1.07898	0.003001	0.03577
59	ZFHX4	15	5	11.20996	1.047027	0.036083
60	MRC2	8	1	364.5262	1.07355	0.036799
61	FBN1	4	12	1.061165	0.072315	0.042781
